# Modular and practical diamination of allenes

**DOI:** 10.1038/s41467-023-37345-8

**Published:** 2023-03-30

**Authors:** Jian-Jun Dai, Xianglin Yin, Lei Li, Mario E. Rivera, Ye-Cheng Wang, Mingji Dai

**Affiliations:** 1 Department of Chemistry and Center for Cancer Research, Purdue University, West Lafayette, IN 47907 USA; 2 Department of Chemistry, Emory University, Atlanta, GA 30322 USA; 3 Present Address: School of Food and Biological Engineering, Hefei University of Technology, Hefei, 230009 China

**Keywords:** Synthetic chemistry methodology, Synthetic chemistry methodology

## Abstract

Vicinal diamines are privileged scaffolds in medicine, agrochemicals, catalysis, and other fields. While significant advancements have been made in diamination of olefins, diamination of allenes is only sporadically explored. Furthermore, direct incorporation of acyclic and cyclic alkyl amines onto unsaturated π systems is highly desirable and important, but problematic for many previously reported amination reactions including the diamination of olefins. Herein, we report a modular and practical diamination of allenes, which offers efficient syntheses of β,γ-diamino carboxylates and sulfones. This reaction features broad substrate scope, excellent functional group tolerability, and scalability. Experimental and computational studies support an ionic reaction pathway initiated with a nucleophilic addition of the in situ formed iodoamine to the electron deficient allene substrate. An iodoamine activation mode via a halogen bond with a chloride ion was revealed to substantially increase the nucleophilicity of the iodoamine and lower the activation energy barrier for the nucleophilic addition step.

## Abstract (web summary)

Diamination of allenes with two different acyclic and cyclic alkyl amines remains a daunting challenge. Here, the authors report a modular and practical diamination of allenes, which offers efficient syntheses of β,γ-diamino carboxylates and sulfones.

## Introduction

**Fig. 1:**
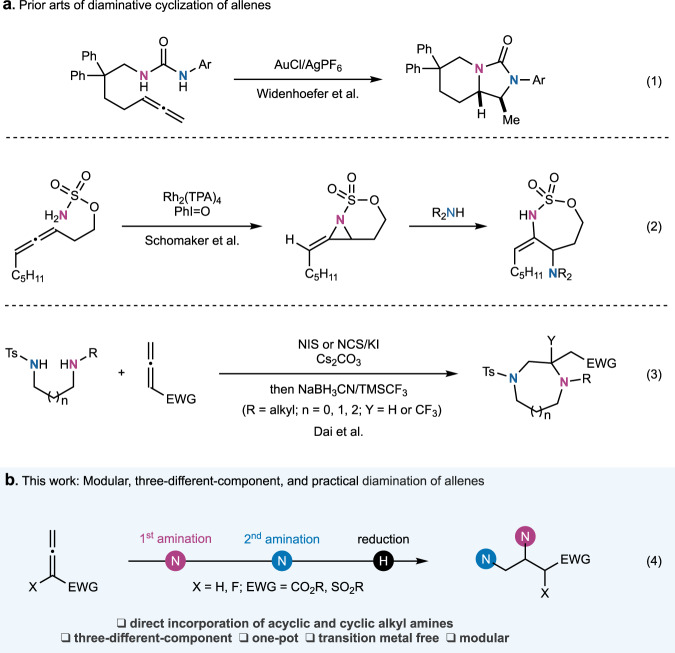
Diamination of allenes. **a** Previous diaminative cyclization of allenes. **b** Our method: Modular and practical diamination of allenes with two different amines.

Vicinal diamines are important scaffolds frequently identified in pharmaceuticals, agrochemicals, and natural products^1,2^. They are also often used as catalysts or ligands for transition metal catalysis^3^. Direct diamination of unsaturated carbon−carbon bonds particularly carbon−carbon double bonds presents an attractive but challenging strategy to prepare vicinal diamines. Remarkable progress has been made in diamination of olefins^4–8^. Various intramolecular^9–33^ and intermolecular^34–50^ olefin diamination reactions have been developed. However, direct diamination of allenes is extremely rare despite the fact that allenes are close relatives of olefins. In 2009, Widenhoefer et al. reported a gold-catalyzed intramolecular dihydroamination of allenes to synthesize bicyclic products with an imidazolidin-2-one moiety (Fig. 1a, eq. 1)^51^. In 2012, Schomaker et al. developed an elegant Rh-catalyzed intramolecular allene aziridination followed by nucleophilic aziridine ring opening with an aniline or amine to synthesize cyclic sulfamates (Fig. 1a, eq. 2)^52^. In 2018, we reported a transition-metal-free diamination of electron deficient allenes with 1,2-, 1,3- or 1,4-diamine derivatives to synthesize saturated 1,4-diazo heterocycles including piperazines, 1,4-diazepanes, and 1,4-diazocanes (Fig. 1a, eq. 3)^53^. In our case, unsymmetrical 1,2-, 1,3- or 1,4-diamines were used to provide orthogonally functionalized or protected 1,4-diazo heterocycles. However, similar to most of the reported olefin diaminations, in these allene diamination cases, a tether between the allene and one or both of the amino groups or a linker between the two amino groups is required to ensure the occurrence of the corresponding C−N bond formation. Furthermore, one or both amino groups need to be activated and/or protected in the forms of sulfonamide, carbamate, or others. The direct use of alkyl amines is rare.

Diamination of unsaturated carbon−carbon bonds with two different amines would offer a modular strategy to synthesize vicinal diamines. However, even for the heavily studied diamination of olefins, including diazidations^54–61^, two identical aminating reagents are often required to avoid regioselectivity issues, which consequently creates problems in differentiating the two nitrogen-containing groups for further modification. Olefin diaminations with two different aminating reagents are very limited^62–67^. So far, there is no report on intermolecular diamination of allenes with two different aminating reagents. Furthermore, the use of prominent acyclic and cyclic alkyl amines in diamination reactions remains a daunting challenge despite many advantages it could offer. For example, saturated *N*-heterocycles are indispensable in drug discovery^68^, but their direct incorporation via diamination method is extremely difficult.

Herein, we report a modular, transition-metal-free, and practical diamination of allenes, which directly incorporates two different and readily available acyclic or cyclic alkylamines into the product under mild conditions with cheap reagents (Fig. 1b, eq. 4). Our mechanistic studies support an ionic reaction pathway involving a nucleophilic addition of an in situ formed iodoamine to the electron deficient allene substrate. An iodoamine activation mode via a halogen bond with a chloride ion was revealed to substantially increase the nucleophilicity of the iodoamine and lower the activation energy barrier for the nucleophilic addition step.

## Results and discussion

### Reaction discovery

Our investigation began with benzyl allenoate **2** and two different cyclic amines: piperidine **1** and 1-phenylpiperazine **3** (Table 1). The desired diamination product **4** (X-ray, CCDC 2004609) was obtained in good isolated yield (70%) under our optimized standard conditions (entry 1). The nature of the halogenation reagent for in situ generation of the haloamine exerted a strong influence on the reaction outcome. For example, when *tert*-butyl hypochlorite (*t*-BuOCl) was replaced with NCS, the yield of product **4** dropped to 47% yield (entry 2). This is partially due to the competition between the succinimide derived from NCS and amine **3** for the second amination step. In comparison to NCS, the byproduct of *t*-BuOCl is *tert*-butanol, a bulky and relatively inert alcohol for nucleophilic substitution reactions^69^. The use of iodide salt is another key to the success of this reaction. Additionally, NIS or NBS alone gave much lower yields (31% and 28% respectively, entries 3 and 4). Only trace amount of product was observed with NCS alone (entry 5). When the amount of tetra-*n*-butylammonium iodide (TBAI) was reduced from 1.5 equiv. to 0.5 equiv., the yield dropped to 41% (entry 6). Without TBAI, the yield was lower than 5% (entry 7). It was further found that TBAI was more effective than inorganic iodide sources including KI (entry 8), presumably due to its better solubility in acetonitrile. The use of Cs_2_CO_3_ was proved to be superior to other bases including K_2_CO_3_ (entry 9), and THF was less effective than acetonitrile as a solvent (entry 10). Furthermore, when the reaction was conducted at 5 mmol scale, product **4** was obtained in even higher yield (entry 11, 78%, 1.64 g). Additionally, when NaBD_3_CN was used as the reducing reagent, a deuterium atom can be introduced at the β-position of **4** directly (entry 12).

**Table 1:** Optimization of the reaction conditions


Entry	Variation from standard conditions	Yield (%)^a^
1	none	70
2	NCS instead of *t*-BuOCl	47
3	NIS (no TBAI)	31
4	NBS (no TBAI)	28
5	NCS (no TBAI)	trace
6	TBAI (50 mol %)	41
7	no TBAI	<5
8	KI instead of TBAI	45
9	K_2_CO_3_ instead of Cs_2_CO_3_	57
10	THF instead of CH_3_CN	26
11	5 mmol of **2**	78 (1.64 g)
12	NaBD_3_CN instead of NaBH_3_CN	57 (X = D)

Reactions were carried out on a 0.2 or 5 mmol scale.

*NCS* *N*-chlorosuccinimide. *NIS N*-iodosuccinimide. *NBS N*-bromosuccinimide. *TBAI* tetra-*n*-butylammonium iodide. *THF* tetrahydrofuran. CH_3_CN acetonitrile.

^a^Isolated yields.

### Substrate scope

**Fig. 2:**
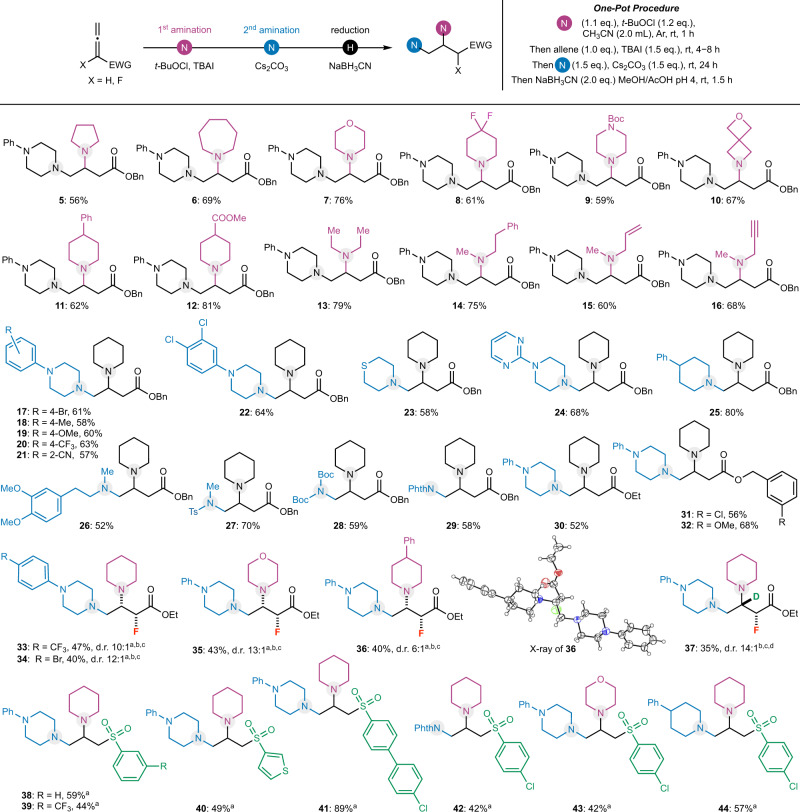
Substrate scope. ^a^NaBH_3_CN (2.0 eq.), EtOH/AcOH, pH 4, −20 °C, 1.5 h. ^b^Ratio was determined by ^19^F NMR of the crude reaction mixture. ^c^Purified *N*-chloroamine was used. ^d^NaBD_3_CN (2.0 eq.), EtOH/AcOH, pH 4, −20 °C, 1.5 h. Bn, benzyl. Ph phenyl. Ts, tosyl. Boc *tert*-butyloxycarbonyl. Phth phthaloyl. d.r. diastereomeric ratio.

With the optimized reaction conditions in hand, the substrate scope of this allene diamination reaction was investigated (Fig. 2). Both cyclic and acyclic aliphatic amines can be used as the aminating reagents for the first C−N bond formation. The cyclic amines include a wide range of saturated *N*-heterocycles such as pyrrolidine (**5**), piperidine derivatives (**8**, **11**, **12**), piperazine (**9**), azepane (**6**), morpholine (**7**), and azetidine (**10**), which are often found in U.S. FDA-approved drug molecules. The acyclic amines are represented by diethyl amine (**13**), *N*-methyl-2-phenylethan-1-amine (**14**), an allylic amine (**15**), and a propargylic amine (**16**). The survival of both a terminal olefin and a terminal alkyne testifies the mildness of the reaction conditions. The amine scope for the second amination is even broader. In addition to cyclic and acyclic aliphatic amines (**5**−**26** and **30**−**40**), sulfonamide (**27**), carbamate (**28**), and imide (**29**) worked smoothly as well. The reaction features excellent functional group tolerability. For example, Boc-carbamate (**28**), ester, olefin (**15**), alkyne (**16**), nitrile (**21**), sulfide (**23**), and pyrimidine (**24**) are all compatible with the mild reaction conditions. In addition to benzyl allenoate, other alkyl groups are tolerated on the carboxylate (**30**−**32**).

We further learned that the reaction is very sensitive to substituents on the α-carbon and γ-carbon of the allenoates. We were delighted to discover that small fluorine atom is tolerated at the α-position of the allenoate, which led to the formation of α-fluoro-β,γ-diamino acid derivatives with modest reaction yield and excellent diastereoselectivity (Fig. 2, **33**−**37**). For these cases, the use of the corresponding purified *N*-chloroamines gave better reaction results than the in situ generation of the *N*-chloroamines. Conducting the NaBH_3_CN reduction at a lower temperature (−20 °C) in a mixture of EtOH and AcOH is important for obtaining high diastereoselectivity. The relative stereochemistry of **36** was determined by X-ray crystallographic analysis (CCDC 2004611). The stereochemistry of the reduction step could be explained by the polar Felkin-Anh model^70^. When NaBD_3_CN was used as the reducing reagent, a remarkably functionalized α-fluoro-β-deutero-β,γ-diamino carboxylate could be produced in just one step (**37**)^71^. The successful use of α-fluoroallenoates provides an efficient method to prepare fluorine-containing molecules, which are important in drug discovery^72^.

To further expand the allene substrate scope, other electron-withdrawing groups such as sulfone and nitrile on the allene were investigated. This effort led to the discovery that allenyl sulfones are effective substrates for this three-different-component diamination (Fig. 2, **38**−**44**). Various products equipped with an aryl (**38**, **39**, **41**−**43**) or heteroaryl (thiophene, **40**) sulfones can be obtained in modest to high yield. Again, the use of *N*-chloroamine directly is beneficial.

### Synthetic applications

**Fig. 3:**
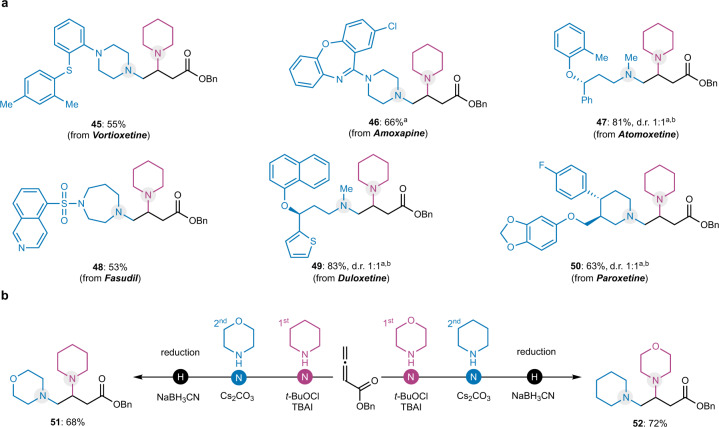
Synthetic applications. **a** Derivatization of FDA-approved drug molecules. **b** Modular synthesis. ^a^Purified *N*-chloroamine was used. ^b^Ratio was determined by ^1^H NMR. d.r. Diastereomeric ratio.

We then explored the usefulness of this diamination reaction as an efficient method for the derivatization of drug molecules (Fig. 3a). Indeed, complex U.S. FDA-approved secondary amine-containing drugs such as vortioxetine (**45**), amoxapine (**46**), atomoxetine (**47**), fasudil (**48**), duloxetine (**49**), and paroxetine (**50**) can be used, which provides an efficient way to incorporate these lifesaving drug molecules into more complex structures to tune or repurpose their function. For the cases of **46**, **47**, **49**, and **50**, the use of *N*-chloropiperidine directly gave slightly higher yields than its in situ generation. More importantly, the position of the amines in the final product could be switched by simply alternating the addition order of the two amines (Fig. 3b). For example, both **51** and **52** could be prepared in good yield by changing the addition order of piperidine and morpholine. This modular feature would enable rapid synthesis of analogs without inventing another synthetic approach and is believed to be useful for structure-activity relationship studies.

### Mechanistic studies

**Fig. 4:**
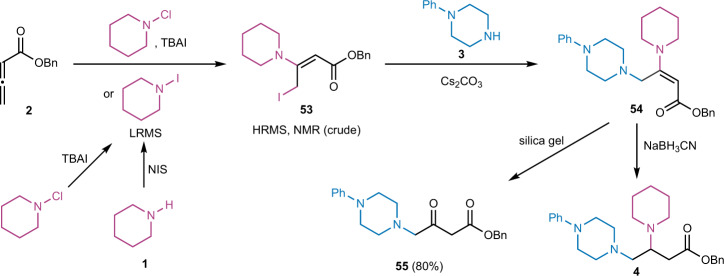
Identification of key intermediates. TBAI tetra-*n*-butylammonium iodide. NIS *N*-iodosuccinimide.

We then started to probe the reaction mechanism. First, investigations were conducted to identify and characterize key intermediates involved in the reaction process (Fig. 4). We prepared and purified *N*-chloropiperidine, then reacted it with allenoate **2** in the presence of TBAI. The formation of an unstable allylic iodide **53** was observed and characterized using HRMS and NMR. The same allylic iodide was obtained when **2** was treated with *N*-iodopiperidine generated in situ from piperidine and NIS. These results indicate the possibility of forming *N*-iodopiperidine from *N*-chloropiperidine and TBAI via a chloride-iodide exchange process. While such a process was not detected by Mass Spec study when the *N*-chloro-1,2-diamine derivative in our previous study^53^ was treated with KI, interestingly, the formation of *N*-iodopiperidine from *N*-chloropiperidine and TBAI was observed by Mass Spec (for details, see Supplementary Fig. 3). After adding 1-phenylpiperazine **3**, substitution product **54** was detected using HRMS and crude NMR, but **54** was not stable and underwent hydrolysis during silica gel column purification to produce γ-amino-β-ketoester **55**. Therefore, the one-pot NaBH_3_CN reduction was carried out to produce β,γ-diamino acid derivative **4**.

**Fig. 5:**
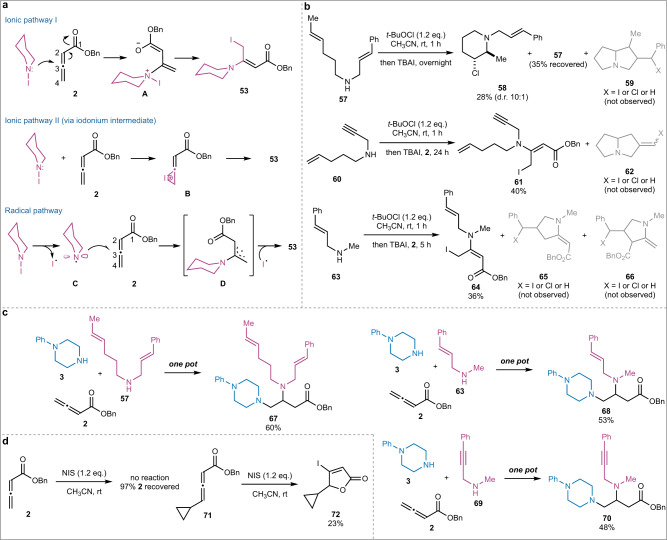
Mechanistic studies. **a** Proposed reaction mechanism. **b** Radical trapping experiments with tethered alkenes and alkynes. **c** Results of substrates **57**, **63**, and **69** under standard diamination conditions. **d** Reactions of allenoates **2** and **71** with NIS. TBAI tetra-*n*-butylammonium iodide. NIS *N*-iodosuccinimide.

The next question was how **53** was formed from treating **2** with *N*-iodopiperidine or a combination of *N*-chloropiperidine and TBAI. Three plausible pathways, a radical and two ionic processes, to its formation are proposed and shown in Fig. 5a. In the first ionic process, *N*-iodopiperidine could attack the sp carbon (C3) of allenoate **2** by using its lone pair electrons to form intermediate **A**, then to **53** via an intramolecular iodine transfer. In the second ionic process, an iodonium ion intermediate could be involved. In the radical process, an amino radical (**C**) could be derived from *N*-iodopiperidine, which would add on allenoate **2** to form a stabilized radical intermediate **D**. Trapping **D** with an iodine radical would form **53**. To differentiate these potential pathways, radical trap experiments with (2,2,6,6-tetramethylpiperidin-1-yl)oxidanyl (TEMPO) and butylated hydroxytoluene (BHT) were then conducted (Supplementary Fig. 6). When BHT was added to the reaction mixture of *N*-chloropiperidine and TBAI, 2,6-di-*tert*-butyl-4-(piperidin-1-ylmethyl)phenol (**56**) was formed in 43% yield. Its formation was not observed without TBAI. Both TEMPO and BHT inhibited the formation of **53** in presence of allenoate **2**. The formation of **56** was again observed when BHT was used in the presence allenoate **2**. These results indicate the amino radical process, but cannot rule out the ionic processes. To further explore the reaction mechanism, alkyl amines **57**, **60**, and **63** were prepared. For **57** and **60**, if an amino radical is formed, cascade radical cyclization products such as **59** and **62** could be formed^73^. After **57** was treated with *t-*BuOCl, then TBAI, product **58** was formed in 28% yield (Fig. 5b). The high stereoselectivity for the formation of **58** suggests a halonium ion intermediate and is against the amino radical process^74^. When **60** was treated with *t-*BuOCl, then TBAI and allenoate **2** was added, allylic iodide **61** was formed in 40% yield (Fig. 5b). Meanwhile, the absence of cascade cyclization products **59** and **62** also disapproves the formation of an amino radical. Alkyl amine **63** was next used to detect whether radical intermediate like **D** is formed during the reaction process (Fig. 5b). After treating **63** with *t-*BuOCl and TBAI, allenoate **2** was added. In this case, we were also able to isolate allylic iodide **64** and fully characterize it. No radical cyclization products **65** and/or **66** were observed, which again disapproves the proposed radical mechanism. When **57**, **63**, and **69** were subjected to the standard one-pot reaction conditions, desired products **67**, **68**, and **70** were obtained in good yields, respectively (Fig. 5c). We then tried to differentiate the two ionic processes. Ma et al. have shown that if iodonium ion intermediate like **B** is formed, a 4-iodofuran-2(5*H*)-one could be formed via an intramolecular iodolactonization process^75^. When allenoate **2** was treated with NIS, no reaction happened and **2** was recovered in 97% yield (Fig. 5d). However, when allenoate **71** was treated with NIS, 4-iodofuran-2(5*H*)-one product **72** was observed (Fig. 5d). Allenoate **71** was originally prepared as a radical clock substrate, but it was not effective because all the other reactions we have tried on **71** led to complex mixtures.

**Fig. 6:**
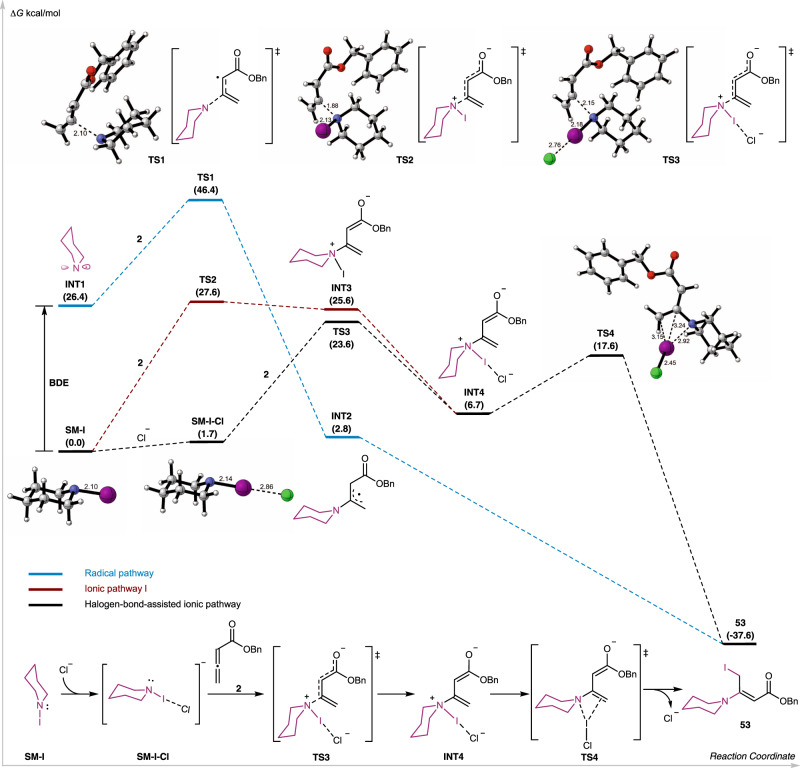
Computational studies. Free energy profiles (kcal/mol) of the radical and ionic processes for the formation of **53** at the Mo6-2x-D3/def2-TZVPP (SMD18, acetonitrile) //M06-2x-D3/def2-SVP (SMD18, acetonitrile) level.

After the radical pathway and the ionic pathway II via iodonium ion intermediate **B** were ruled out, we started to study the ionic pathway I via intermediate **A** using DFT calculation (Fig. 6). The radical pathway was calculated as well to shed light on why it was not operative under the current reaction conditions. As shown in Fig. 6, the radical pathway would require a very high energy barrier of the radical Michael addition process (**TS1**, 46.4 kcal/mol), while the rest of the radical processes were energetically favored. For the ionic pathway I, the free energy barrier of direct Michael addition between **SM-I** (*N*-iodopiperidine) and **2** was calculated as 27.6 kcal/mol (**TS2**), which is inconsistent with the mild room temperature reaction condition. Thus, other factors need to be considered here to either increase the nucleophilicity of **SM-I** or the electrophilicity of allenoate **2**. We proposed that the free chloride ion in the system would have weak interaction with the σ hole of the iodine atom on **SM-I** through halogen bond^76^ to form **SM-I-Cl** complex, which could make the nitrogen atom more nucleophilic, thus lowering the energy barrier for the nucleophilic addition step. Indeed, stronger nucleophilicity of the nitrogen atom of **SM-I-Cl** was predicted based on comparison of N−I distance in **SM-I** (2.10 Å) and **SM-I-Cl** (2.14 Å). Then a halogen bond assisted Michael addition was calculated to form **INT4** via transition state **TS3** (23.6 kcal/mol) with about 4 kcal/mol lower free energy than **TS3** (27.6 kcal/mol). Notably, the free iodide ion could activate **SM-I** via the **SM-I-I** complex, but computationally the effect was not as significant as the chloride ion. Finally, product **53** was generated from **INT4** through an iodine monochloride mediated 4-membered ring transition state **TS4** (17.8 kcal/mol). The finalized mechanism for the formation of **53** from **SM-I** and allenoate **2** is summarized at the bottom of Fig. 6.

In summary, a one-pot, transition-metal-free, modular, and practical intermolecular diamination of electron deficient allenes (allenoates and allenyl sulfones) was developed to allow efficient synthesis of (α-fluoro-)β,γ-diamino acid/sulfone derivatives. This reaction features mild reaction conditions, readily available and cheap reagents, and excellent functional group tolerability. Both cyclic and acyclic aliphatic amines can be used. In addition, amide, imide, and sulfonamide can be used in the second nucleophilic amination step. Small fluorine atom is tolerated at the α-carbon of the allenoates, which offers a method to prepare α-fluorine-containing molecules for novel therapeutic development. Experimental and computational mechanism studies supported the ionic pathway via a nucleophilic addition of the corresponding iodoamine to the electron deficient allene substrate. Furthermore, an iodoamine activation mode via a halogen bond with a chloride ion was revealed to substantially increase the nucleophilicity of the iodoamine, which can be explored to discover new reactions.

## Methods

### General procedures for modular three-different-component diamination of allenes general procedure A

To an oven-dried 10 mL vial wrapped with aluminum foil and equipped with a stir bar was added the indicated amine (0.11 mmol, 1.1 equiv), *t-*BuOCl (14.0 µL, 0.12 mmol, 1.2 equiv), and CH_3_CN (2.0 mL) under argon atmosphere. The reaction mixture was stirred for 1 h at room temperature. Then, tetra-*n*-butylammonium iodide (TBAI, 55.4 mg, 0.15 mmol, 1.5 equiv) and allenoate (0.1 mmol, 1.0 equiv) were added. The reaction mixture was stirred for 4−8 h (TLC monitored the conversion of allenoate). After the indicated time, Cs_2_CO_3_ (48.9 mg, 0.15 mmol, 1.5 equiv) and the indicated amine (0.15 mmol, 1.5 equiv) were added. The reaction mixture was stirred for 24 h at room temperature. Subsequently, NaBH_3_CN (0.2 mmol) and a co-solvent of MeOH/AcOH (pH = 4, 1.0 mL) were added to the reaction mixture. After 1.5 h, the reaction was quenched with a saturated aqueous solution of NaHCO_3_ and extracted with CH_2_Cl_2_ for three times. The combined organic extracts were dried over Na_2_SO_4_, filtered, and concentrated under reduced pressure. The resulting crude mixture was purified by flash column chromatography on silica gel (hexanes/EtOAc/Et_3_N) to afford the desired compound.

### General procedure B

To an oven-dried 10 mL vial wrapped with aluminum foil and equipped with a stir bar was added allene (0.1 mmol, 1.0 equiv), *N*-chloroamine (0.11 mmol, 1.1 equiv), tetra-*n*-butylammonium iodide (TBAI, 55.4 mg, 0.15 mmol, 1.5 equiv) and CH_3_CN (2.0 mL) under argon atmosphere. The reaction mixture was stirred for 2−8 h (TLC monitored the conversion of allenoate). After the indicated time, Cs_2_CO_3_ (48.9 mg, 0.15 mmol, 1.5 equiv) and the indicated amine were added. The reaction mixture was then stirred for 24 h at room temperature. Subsequently, NaBH_3_CN (0.2 mmol) and a co-solvent of MeOH/AcOH (pH = 4, 1.0 mL) were added to the reaction mixture. After 1.5 h, the reaction was quenched with a saturated aqueous solution of NaHCO_3_ and extracted with CH_2_Cl_2_ for three times. The combined organic extracts were dried over Na_2_SO_4_, filtered, and concentrated under reduced pressure. The resulting crude mixture was purified by flash column chromatography on silica gel (hexanes/EtOAc/Et_3_N) to afford the desired compound.

## Supplementary information

Supplementary Information

Description of Additional Supplementary Files

Supplementary Data 1

## Data Availability

The authors declare that the data supporting the findings of this study are available within the article and its supplementary information files or from the corresponding author upon request. ^1^H, ^13^C, ^19^F NMR spectra, and high resolution mass spectrometry data are available in the Supplementary Information. The Cartesian coordinates data generated in this study are provided in the Supplementary Data 1. The crystallographic data generated in this study have been deposited in the Cambridge Crystallographic Data Center database under accession code CCDC 2004609 (**4**) and CCDC 2004611 (**36**) (www.ccdc.cam.ac.uk/data_request/cif).
