# Supplementary material for: Modular and practical diamination of allenes
Source: Nat Commun. 2023 Mar 30;14:1774. doi: 10.1038/s41467-023-37345-8 (PMC10063549; doi:10.1038/s41467-023-37345-8)
Supplement: Supplementary file 1 — Supplementary Information [file 41467_2023_37345_MOESM1_ESM.pdf]

Supplementary Information

Modular and Practical Diamination of Allenes

Jian-Jun Dai[1‡†], Xianglin Yin[1†], Lei Li[1,2†], Mario E. Rivera[1,2], Ye-Cheng Wang[1*], Mingji Dai[1,2*]

[1]Department of Chemistry and Center for Cancer Research, Purdue University, West Lafayette, IN 47907, United States.

[2]Department of Chemistry, Emory University, Atlanta, GA 30322, United States.

[‡]Present address: School of Food and Biological Engineering, Hefei University of Technology, Hefei 230009, China.

[†]These authors contributed equally: Jian-Jun Dai, Xianglin Yin, Lei Li.

*E-mail: wang3737@purdue.edu; mingji.dai@emory.edu

Table of Contents

I. Supplementary Notes S3

1. General Information S3

II. Supplementary Methods S3

2. Preparation and Characterization of Starting Materials S3

3. General Procedures for Diamination of Allenes S6

4. Characterization Data for Diamination Products S7

III. Supplementary Discussion S31

5. X-Ray Crystallography Data for Compounds **4** and **36** S31

6. Mechanistic Study Experiments S35

7. DFT Calculation S44

IV. Supplementary Figures S49

8. Copies of NMR Spectra S49

V. Supplementary References S121

I. Supplementary Notes

1. General Information

All reagents were used as received from commercial sources unless specified otherwise or prepared as described in the literature. Anhydrous THF and toluene were distilled over sodium and diphenylketone under Argon. Anhydrous CH_2Cl_2 and CH_3CN was distilled over calcium hydride under Argon. Analytical TLC was done on pre-coated silica gel plates. Organic solutions were concentrated under reduced pressure on a Büchi rotary evaporator. Oil bath was used for reaction heating. Product purification was performed by flash column chromatography with silica gel (200−300 mesh) unless otherwise stated. ^1H, ^{13}C, ^{19}F NMR spectra were recorded on a 500 MHz or a 600 MHz instrument. Data are reported in the following format: chemical shift in ppm, multiplicity (s = singlet, d = doublet, t = triplet, q = quartet, bs = broad singlet, m = multiplet, dd = doublet of doublets, etc.), coupling constant *J* in Hz, and integration. $CDCl_3$ was used as received. NMR chemical shifts are reported in ppm relative to $CDCl_3$ (7.26 ppm ^1H and 77.2 ppm ^{13}C). Partial IR spectra are reported. GC-MS analyses were performed on a GC-MS with an EI mode. High-resolution mass spectra (HRMS) were obtained using electrospray ionization (ESI) on a time of flight (TOF) mass spectrometer.

II. Supplementary Methods

2. Preparation and Characterization of Starting Materials

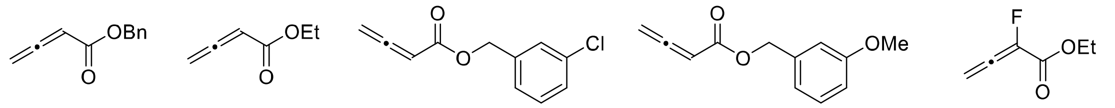

2 **A1** **A2** **A3** **A4**

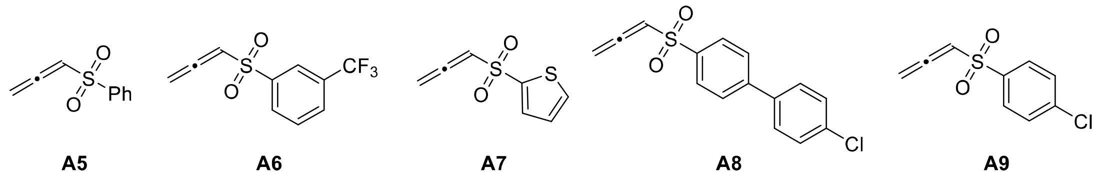

A5 **A6** **A7** **A8** **A9**

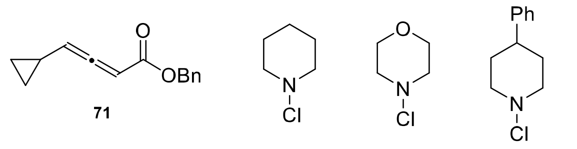

N-chloroamines

Allenoates **2**, **A1**, **A2**, **A3** were each synthesized according to literature procedures[1,2].

Allenoate **A4** was synthesized according to the following procedure.

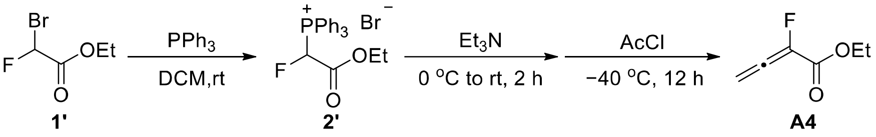

In a 20.0 mL flame dried vial, ethyl 2-bromo-2-fluoroacetate **1'** (1.8 g, 9.7 mmol) and PPh_3 (2.81 g, 10.7 mmol) were dissolved in 7.0 mL anhydrous DCM. The vial was capped and sealed by parafilm and wrapped by aluminum foil. The reaction was stirred for 5 days and diluted with 30.0 mL anhydrous DCM. At 0 °C Et_3N was added and the reaction was stirred for 2 h at room temperature. The reaction was cooled to −40 °C and AcCl (917 mg, 11.7 mmol) was added dropwise and the reaction was stirred at −40 °C for 12 h. The solution was directly passed through a silica gel plug and washed down by 10% Et_2O in pentane. Then, the crude solution was concentrated under vacuum in an ice bath and purified by column chromatography (1−2% Et_2O in pentane) to afford allene **A4** as a colorless oil.

Allene **A5** was prepared according to literature procedure[3].

Allenes **A6**, **A7**, **A8** and **A9** synthesized according to the following procedure.

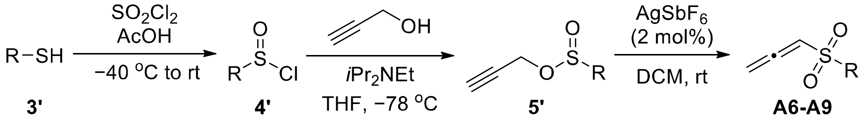

The thiophenol **3'** (1.0 equiv) and acetic acid (1.0 equiv) were mixed in an 8 mL vial with stir bar and the solution was cooled to −40 °C sulfuryl chloride (2.0 equiv) was added dropwise at this temperature to the frozen mixture. After completing addition, the reaction mixture was stirred at −40 °C for 30 min and slowly increase to room temperature and then stir for further 3 h. The solution was concentrated to give product **4'** in quantitative yield as yellow oil. The freshly prepared **4'** was dissolved in THF and cooled to −78 °C. Propargylic alcohol (1.0 equiv) was added followed by diisopropylethylamine (1.2 equiv). The reaction was stirred at −78 °C for 2 h and quenched with water. The organic layer was extracted by EtOAc and dried over Na_2SO_4. After filtration, the organic layer was concentrated and purified by column chromatography to afford sulfinic ester **5'** as colorless oil. **5'** was dissolved in anhydrous DCM and catalytic amount of $AgSbF_6$ (2 mol%) was added. The reaction was monitored by TLC. After reaction is completed, solvent was removed, and the crude product was purified by column chromatography to afford allenyl sulfone **A6**−**A9** as white solid.

Allene **71** synthesized according to the following procedure.

To a solution of triphenylphosphorane (0.82 g, 2 mmol) in DCM (5 mL) was added Et_3N (0.3 mL, 2.2 mmol) and then a solution of Acyl chloride (0.2 mL, 2 mmol) in 2 mL DCM was added slowly in about 20 min. The reaction mixture was stirred overnight and concentrated. Pentane was added and then insoluble triphenylphosphine oxide was filtered off. The filtrate was concentrated and the crude product was purified by flash column chromatography on silica gel (eluted with PE/Ethyl ether 20:1) to afford product **71** (201 mg, 47%).

N-chloroamines were synthesized according to literature procedure[4].

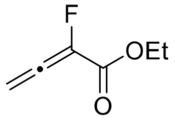

Ethyl 2-fluorobuta-2,3-dienoate (A4)

^{1}H NMR (500 MHz, $CDCl_3$) δ 5.91 (s, 2H), 4.30 (q, *J* = 7.1 Hz, 2H), 1.32 (t, *J* = 7.1 Hz, 3H); **^{13}C NMR** (126 MHz, $CDCl_3$) δ 201.6 (d, *J* = 22.8 Hz), 161.6 (d, *J* = 38.5 Hz), 131.7 (d, *J* = 238.3 Hz), 96.0 (d, *J* = 8.6 Hz), 62.2, 14.3; **^{19}F NMR** (471 MHz, $CDCl_3$) δ −157.61; **GCMS** (EI) *m/z* 130 $[M]^+$; **FTIR** (neat) ν_{max} 2985, 2919, 1768, 1723, 1372, 1312, 1180, 1094, 1017, 945, 771 cm^{-1}. *(Note: we tried different ionization and detection methods, but were not able to obtain the mass spectrum of allenoate **A4**).*

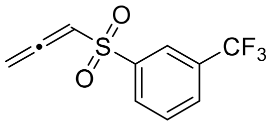

1-(Propa-1,2-dien-1-ylsulfonyl)-3-(trifluoromethyl)benzene (A6)

^{1}H NMR (500 MHz, $CDCl_3$) δ 8.19 (s, 1H), 8.12 (d, *J* = 7.9 Hz, 1H), 7.90 (d, *J* = 7.8 Hz, 1H), 7.72 (t, *J* = 7.8 Hz, 1H), 6.27 (t, *J* = 6.3 Hz, 1H), 5.52 (d, *J* = 6.4 Hz, 2H); **^{13}C NMR** (126 MHz, $CDCl_3$) δ 210.2, 142.6, 132.2 (q, *J* = 33.4 Hz), 131.1, 130.4 (d, *J* = 2.6 Hz), 130.3, 125.0 (d, *J* = 3.6 Hz), 123.3 (q, *J* = 272.1 Hz), 100.8, 84.8; **^{19}F NMR** (470 MHz, $CDCl_3$) δ −64.03; **HRMS** (ESI/$[M+H]^+$) calcd. for $C_{10}H_8F_3O_2S^+$: 249.0192, found $[M+H]^+$: 249.0192; **FTIR** (neat) 1966, 1326, 1148, 692, 613 cm^{-1}.

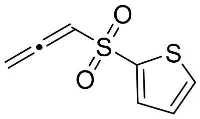

2-(Propa-1,2-dien-1-ylsulfonyl)thiophene (A7)

^{1}H NMR (500 MHz, $CDCl_3$) δ 7.77 – 7.56 (m, 2H), 7.14 (dd, *J* = 4.9, 3.9 Hz, 1H), 6.35 (t, *J* = 6.3 Hz, 1H), 5.48 (d, *J* = 6.3 Hz, 2H); **^{13}C NMR** (126 MHz, $CDCl_3$) δ 209.3, 142.6, 134.1, 133.8, 128.0, 102.1, 84.8; **HRMS** (ESI/$[M+H]^+$) calcd. for $C_7H_8O_2S_2^+$: 208.9701, found $[M+H]^+$: 208.9702; **FTIR** (neat) ν_{max} 1965, 1401, 1323, 1141, 1015, 609 cm^{-1}.

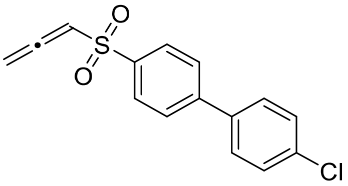

4-Chloro-4'-(propa-1,2-dien-1-ylsulfonyl)-1,1'-biphenyl (A8)

^{1}H NMR (500 MHz, $CDCl_3$) δ 8.03 – 7.91 (m, 2H), 7.77 – 7.65 (m, 2H), 7.60 – 7.51 (m, 2H), 7.51 – 7.41 (m, 2H), 6.28 (t, *J* = 6.4 Hz, 1H), 5.48 (d, *J* = 6.3 Hz, 2H); **^{13}C NMR** (126 MHz, $CDCl_3$) δ 209.7, 145.4, 140.3, 137.7, 135.1, 129.5, 128.8, 128.4, 127.9, 101.2, 84.4; **HRMS** (ESI/[M+H]$^+$) calcd. for $C_{15}H_{12}ClO_2S^+$: 291.0241, found [M+H]$^+$:291.0241; **FTIR** (neat) ν_{max} 1965, 1595, 1478, 1320, 1146, 1088, 628 cm^{-1}.

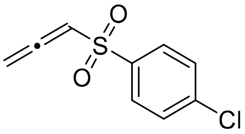

1-Chloro-4-(propa-1,2-dien-1-ylsulfonyl)benzene (A9)

^{1}H NMR (500 MHz, $CDCl_3$) δ 7.84 (d, *J* = 8.7 Hz, 2H), 7.57 – 7.46 (m, 2H), 6.23 (t, *J* = 6.3 Hz, 1H), 5.46 (d, *J* = 6.3 Hz, 2H); **^{13}C NMR** (126 MHz, $CDCl_3$) δ 209.7, 140.4, 139.8, 129.7, 129.3, 101.0, 84.5; **HRMS** (ESI/[M+H]$^+$) calcd. for $C_9H_8ClO_2S^+$: 214.9929, found [M+H]$^+$: 214.9928; **FTIR** (neat) ν_{max} 1966, 1579, 1476, 1394, 1322, 1148, 1086 cm^{-1}.

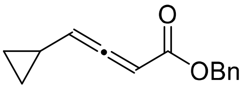

Benzyl 4-cyclopropylbuta-2,3-dienoate (71)

^{1}H NMR (400 MHz, $CDCl_3$) δ 7.48 – 7.28 (m, 5H), 5.70 (dd, *J* = 6.1, 1.3 Hz, 1H), 5.51 (dd, *J* = 7.7, 6.1 Hz, 1H), 5.18 (m, 2H), 1.42 – 1.30 (m, 1H), 0.80 (dd, *J* = 8.1, 1.7 Hz, 2H), 0.46 (m, 2H); **^{13}C NMR** (100 MHz, $CDCl_3$) δ 213.0, 165.8, 136.0, 128.5, 128.1, 128.1, 100.2, 89.5, 66.5, 8.4, 7.3, 7.0; **HRMS** (ESI/[M+H]$^+$) calcd. for $C_{14}H_{15}O_2^+$: 215.1067, found [M+H]$^+$: 215.1069; **FTIR** (neat) ν_{max} 2921, 2850, 1957, 1715, 1455, 1250, 1148, 735, 696 cm^{-1}.

3. General Procedures for Diamination of Allenes

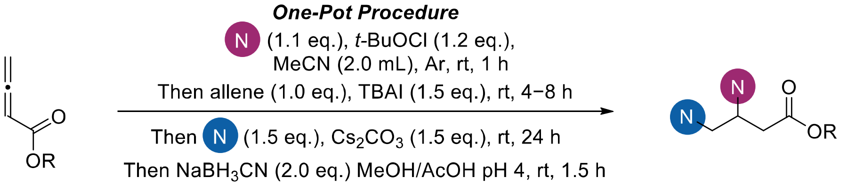

General Procedure A: To an oven-dried 10 mL vial wrapped with aluminum foil and equipped with a stir bar was added the indicated amine (0.11 mmol, 1.1 equiv), *t*-BuOCl (14.0 μL, 0.12 mmol, 1.2

equiv), and CH_3CN (2.0 mL) under argon atmosphere. The reaction mixture was stirred for 1 h at room temperature. Then, tetra-*n*-butylammonium iodide (TBAI, 55.4 mg, 0.15 mmol, 1.5 equiv), allenoate (0.1 mmol, 1.0 equiv) was added and the reaction mixture was stirred for 4−8 h (TLC monitored the conversion of allenoate). After the indicated time, Cs_2CO_3 (48.9 mg, 0.15 mmol, 1.5 equiv) and the indicated amine (0.15 mmol, 1.5 equiv) were added. The reaction mixture was stirred for 24 h at room temperature. Subsequently, $NaBH_3CN$ (0.2 mmol) and a co-solvent of MeOH/AcOH (pH = 4, 1.0 mL) were added to the reaction mixture. After 1.5 h, the reaction was quenched with a saturated aqueous solution of $NaHCO_3$, extracted with CH_2Cl_2 for three times. The combined organic extracts were dried over Na_2SO_4, filtered, and concentrated under reduced pressure. The resulting crude mixture was purified by flash column chromatography on silica gel (hexanes/EtOAc/Et_3N) to afford the desired compound.

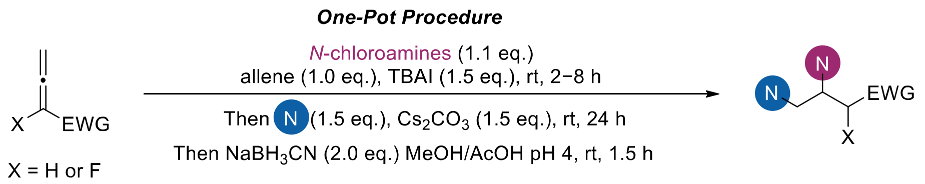

General Procedure B: To an oven-dried 10 mL vial wrapped with aluminum foil and equipped with a stir bar was added allene (0.1 mmol, 1.0 equiv), *N*-chloroamine (0.11 mmol, 1.1 equiv), tetra-n-butylammonium iodide (TBAI, 55.4 mg, 0.15 mmol, 1.5 equiv) and CH_3CN (2.0 mL) under argon atmosphere. The reaction mixture was stirred for 2−8 h (TLC monitored the conversion of allenoate). After the indicated time, Cs_2CO_3 (48.9 mg, 0.15 mmol, 1.5 equiv) and the indicated amine were added. The reaction mixture was then stirred for 24 h at room temperature. Subsequently, $NaBH_3CN$ (0.2 mmol) and a co-solvent of MeOH/AcOH (pH = 4, 1.0 mL) were added to the reaction mixture. After 1.5 h, the reaction was quenched with a saturated aqueous solution of $NaHCO_3$, extracted with CH_2Cl_2 for three times. The combined organic extracts were dried over Na_2SO_4, filtered, and concentrated under reduced pressure. The resulting crude mixture was purified by flash column chromatography on silica gel (hexanes/EtOAc/Et_3N) to afford the desired compound.

4. Characterization Data for Diamination Products

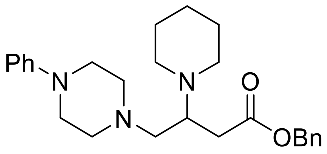

Benzyl 4-(4-phenylpiperazin-1-yl)-3-(piperidin-1-yl)butanoate (4)

Following the general procedure A, using allenoate **2** (17.4 mg, 0.1 mmol, 1.0 equiv), piperidine (11.0 μL, 0.11 mmol, 1.1 equiv) and 1-phenylpiperazine (24.3 mg, 0.15 mmol, 1.5 equiv). The reaction time of the first amination step was 4 h. The product was isolated by silica gel

chromatography as a white solid (0.1 mmol scale 29.5 mg, 70% yield, m.p. 55−58 °C; **5 mmol scale 1.64 g, 78%**). **^{1}H NMR** (500 MHz, $CDCl_3$) δ 7.38 – 7.22 (m, 7H), 6.94 – 6.87 (m, 2H), 6.84 (t, *J* = 7.3 Hz, 1H), 5.17 – 5.04 (m, 2H), 3.36 – 3.26 (m, 1H), 3.17 – 3.04 (m, 4H), 2.76 – 2.69 (m, 2H), 2.62 – 2.35 (m, 10H), 1.57 – 1.46 (m, 4H), 1.44 – 1.36 (m, 2H); **^{13}C NMR** (126 MHz, $CDCl_3$) δ 173.1, 151.5, 136.3, 129.2, 128.6, 128.3, 128.2, 119.6, 116.1, 66.2, 60.0, 59.4, 53.8, 50.2, 49.3, 35.4, 26.6, 24.9; **HRMS** (ESI/[M+H]$^+$) calcd. for $C_{26}H_{36}N_3O_2^+$: 422.2802, found [M+H]$^+$: 422.2802; **FTIR** (neat) v_{max} 2933, 2810, 1733, 1599, 1495, 1453, 1233, 1010, 757, 695 cm^{-1}.

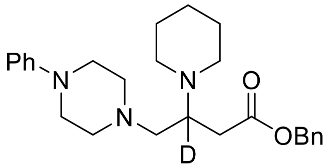

Benzyl 4-(4-phenylpiperazin-1-yl)-3-(piperidin-1-yl)butanoate-3-*d* (4D)

Following the general procedure A, using allenoate **2** (17.4 mg, 0.1 mmol, 1.0 equiv), piperidine (11.0 μL, 0.11 mmol, 1.1 equiv) and 1-phenylpiperazine (24.3 mg, 0.15 mmol, 1.5 equiv). The reaction time of the first amination step was 4 h. $NaBD_3CN$ was used instead of $NaBH_3CN$. The product was isolated by silica gel chromatography as a colorless oil (24.2 mg, 57% yield). **^{1}H NMR** (500 MHz, $CDCl_3$) δ 7.40 – 7.29 (m, 5H), 7.28 – 7.23 (m, 2H), 6.90 (d, *J* = 7.9 Hz, 2H), 6.84 (t, *J* = 7.3 Hz, 1H), 5.16 – 5.06 (m, 2H), 3.16 – 3.03 (m, 4H), 2.78 – 2.67 (m, 2H), 2.61 – 2.33 (m, 10H), 1.55 – 1.48 (m, 4H), 1.43 – 1.37 (m, 2H); **^{13}C NMR** (126 MHz, $CDCl_3$) δ 173.1, 151.5, 136.3, 129.2, 128.6, 128.3, 128.2, 119.7, 116.1, 66.3, 59.9, 53.8, 50.1, 49.3, 35.3, 26.6, 24.9. The quaternary carbon connected with deuterium was omitted; **HRMS** (ESI/[M+H]$^+$) calcd. for $C_{26}H_{35}DN_3O_2^+$: 423.2865, found [M+H]$^+$: 423.2864; **FTIR** (neat) v_{max} 2934, 2817, 1730, 1599, 1497, 1454, 1233, 1007, 753 cm^{-1}.

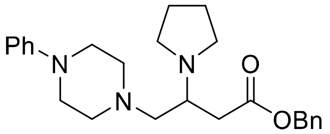

Benzyl 4-(4-phenylpiperazin-1-yl)-3-(pyrrolidin-1-yl)butanoate (5)

Following the general procedure A, using allenoate **2** (17.4 mg, 0.1 mmol, 1.0 equiv), pyrrolidine (9.0 μL, 0.11 mmol, 1.1 equiv) and 1-phenylpiperazine (24.3 mg, 0.15 mmol, 1.5 equiv). The reaction time of the first amination step was 4 h. The product was isolated by silica gel chromatography as a colorless oil (22.8 mg, 56% yield). **^{1}H NMR** (500 MHz, $CDCl_3$) δ 7.38 – 7.22 (m, 7H), 6.90 (d, *J* = 8.1 Hz, 2H), 6.85 (t, *J* = 7.3 Hz, 1H), 5.18 – 5.05 (m, 2H), 3.29 – 3.19 (m, 1H), 3.16 – 3.06 (m, 4H), 2.75 – 2.69 (m, 2H), 2.67 – 2.56 (m, 7H), 2.55 – 2.47 (m, 1H), 2.43 (dd, *J* = 12.3, 9.8 Hz, 1H), 1.74 (bs, 4H); **^{13}C NMR** (126 MHz, $CDCl_3$) δ 173.0, 151.5, 136.2, 129.2, 128.6, 128.32, 128.25, 119.6, 116.1, 66.3, 61.8, 57.0, 53.9, 50.3, 49.3, 36.8, 23.5; **HRMS** (ESI/[M+H]$^+$) calcd. for $C_{25}H_{34}N_3O_2^+$: 408.2646, found [M+H]$^+$: 408.2635; **FTIR** (neat) v_{max} 2959, 2817, 1731, 1599, 1500, 1454, 1232, 1135, 1009, 756, 691 cm^{-1}.

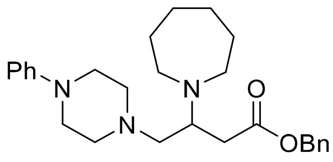

Benzyl 3-(azepan-1-yl)-4-(4-phenylpiperazin-1-yl)butanoate (6)

Following the general procedure A, using allenoate **2** (17.4 mg, 0.1 mmol, 1.0 equiv), azepane (12.0 μL, 0.11 mmol, 1.1 equiv) and 1-phenylpiperazine (24.3 mg, 0.15 mmol, 1.5 equiv). The reaction time of the first amination step was 7 h. The product was isolated by silica gel chromatography as a colorless oil (30.1 mg, 69% yield). **^{1}H NMR** (500 MHz, $CDCl_3$) δ 7.41 – 7.30 (m, 5H), 7.29 – 7.24 (m, 2H), 6.94 – 6.90 (m, 1H), 6.88 – 6.83 (m, 1H), 5.18 – 5.07 (m, 2H), 3.44 – 3.35 (m, 1H), 3.20 – 3.06 (m, 4H), 2.77 – 2.66 (m, 4H), 2.66 – 2.59 (m, 2H), 2.57 – 2.46 (m, 4H), 2.40 – 2.28 (m, 1H), 1.66 – 1.46 (m, 8H); **^{13}C NMR** (126 MHz, $CDCl_3$) δ 173.1, 151.5, 136.3, 129.2, 128.6, 128.4, 128.2, 119.6, 116.0, 66.3, 60.4, 59.8, 53.8, 51.6, 49.3, 36.7, 30.0, 27.0; **HRMS** (ESI/[M+H]$^+$) calcd. for $C_{27}H_{38}N_3O_2^+$: 436.2959, found [M+H]$^+$: 436.2950; **FTIR** (neat) ν_{max} 2924, 2818, 1731, 1599, 1500, 1453, 1231, 1135, 1008, 756, 694 cm^{-1}.

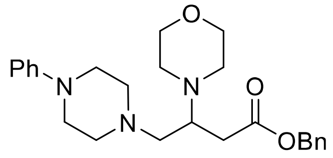

Benzyl 3-morpholino-4-(4-phenylpiperazin-1-yl)butanoate (7)

Following the general procedure A, using allenoate **2** (17.4 mg, 0.1 mmol, 1.0 equiv), morpholine (10.0 μL, 0.11 mmol, 1.1 equiv) and 1-phenylpiperazine (24.3 mg, 0.15 mmol, 1.5 equiv). The reaction time of the first amination step was 7 h. The product was isolated by silica gel chromatography as a colorless oil (32.2 mg, 76% yield). **^{1}H NMR** (500 MHz, $CDCl_3$) δ 7.39 – 7.29 (m, 5H), 7.29 – 7.23 (m, 2H), 6.93 – 6.89 (m, 2H), 6.85 (t, *J* = 7.3 Hz, 1H), 5.22 – 5.06 (m, 2H), 3.62 (t, *J* = 4.6 Hz, 4H), 3.35 – 3.23 (m, 1H), 3.19 – 3.08 (m, 4H), 2.76 – 2.62 (m, 4H), 2.61 – 2.48 (m, 7H), 2.41 – 2.31 (m, 1H); **^{13}C NMR** (126 MHz, $CDCl_3$) δ 172.7, 151.4, 136.2, 129.2, 128.7, 128.4, 128.3, 119.7, 116.1, 67.5, 66.4, 59.3, 58.9, 53.7, 49.3, 35.7, 29.8; **HRMS** (ESI/[M+H]$^+$) calcd. for $C_{25}H_{34}N_3O_3^+$: 424.2595, found [M+H]$^+$: 424.2596; **FTIR** (neat) ν_{max} 2949, 2818, 1731, 1599, 1500, 1497, 1454, 1308, 1232, 1135, 1115, 1009, 756, 695 cm^{-1}.

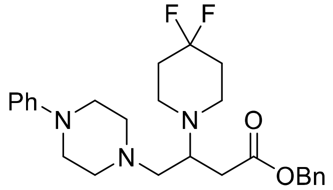

Benzyl 3-(4,4-difluoropiperidin-1-yl)-4-(4-phenylpiperazin-1-yl)butanoate (8)

Following the general procedure A, using allenoate **2** (17.4 mg, 0.1 mmol, 1.0 equiv), 4,4-difluoropiperidine (13.3 mg, 0.11 mmol, 1.1 equiv) and 1-phenylpiperazine (24.3 mg, 0.15 mmol, 1.5 equiv). The reaction time of the first amination step was 7 h. The product was isolated by silica

gel chromatography as a white solid (28.1 mg, 61% yield, m.p. 87−89 °C). **^{1}H NMR** (500 MHz, $CDCl_3$) δ 7.40 – 7.30 (m, 5H), 7.29 – 7.23 (m, 2H), 6.93 – 6.89 (m, 2H), 6.86 (tt, *J* = 7.3, 1.1 Hz, 1H), 5.18 – 5.07 (m, 2H), 3.41 (p, *J* = 7.0 Hz, 1H), 3.13 (td, *J* = 4.6, 4.2, 2.1 Hz, 4H), 2.76 (dt, *J* = 11.5, 5.7 Hz, 2H), 2.68 (dt, *J* = 10.4, 5.0 Hz, 2H), 2.61 (dt, *J* = 11.5, 5.6 Hz, 2H), 2.58 – 2.44 (m, 5H), 2.34 (dd, *J* = 12.4, 8.9 Hz, 1H), 1.93 – 1.80 (m, 4H); **^{13}C NMR** (126 MHz, $CDCl_3$) δ 172.5, 151.4, 136.2, 129.2, 128.7, 128.5, 128.4, 122.3 (t, *J* = 241.4 Hz), 119.8, 116.1, 66.4, 59.3, 58.5, 53.7, 49.3, 45.6 (t, *J* = 4.7 Hz), 36.3, 34.7 (t, *J* = 22.4 Hz); **^{19}F NMR** (471 MHz, $CDCl_3$) δ −98.7; **HRMS** (ESI/[M+H]$^+$) calcd. for $C_{26}H_{34}F_2N_3O_2^+$: 458.2614, found [M+H]$^+$: 458.2608; **FTIR** (neat) ν_{max} 2941, 2821, 1731, 1599, 1497, 1454, 1360, 1231, 1135, 1091, 1010, 947, 927, 756, 696 cm^{-1}.

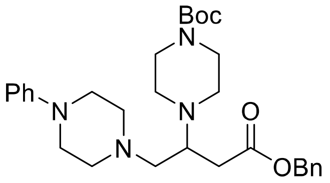

tert-Butyl 4-(4-(benzyloxy)-4-oxo-1-(4-phenylpiperazin-1-yl)butan-2-yl)piperazine-1-carboxylate (9)

Following the general procedure A, using allenoate **2** (17.4 mg, 0.1 mmol, 1.0 equiv), *tert*-butyl piperazine-1-carboxylate (20.5 mg, 0.11 mmol, 1.1 equiv) and 1-phenylpiperazine (24.3 mg, 0.15 mmol, 1.5 equiv). The reaction time of the first amination step was 8 h. The product was isolated by silica gel chromatography as a colorless oil (30.7 mg, 59% yield). **^{1}H NMR** (500 MHz, $CDCl_3$) δ 7.42 – 7.28 (m, 5H), 7.28 – 7.22 (m, 2H), 6.94 – 6.88 (m, 2H), 6.85 (tt, *J* = 7.3, 1.1 Hz, 1H), 5.21 – 5.01 (m, 2H), 3.42 – 3.26 (m, 5H), 3.21 – 3.02 (m, 4H), 2.74 – 2.65 (m, 2H), 2.63 – 2.57 (m, 2H), 2.56 – 2.44 (m, 7H), 2.41 – 2.29 (m, 1H), 1.45 (s, 9H); **^{13}C NMR** (126 MHz, $CDCl_3$) δ 172.6, 154.9, 151.4, 136.1, 129.2, 128.7, 128.4, 128.3, 119.7, 116.1, 79.7, 66.4, 59.4, 58.8, 53.7, 49.3, 35.9, 28.6; **HRMS** (ESI/[M+H]$^+$) calcd. for $C_{30}H_{43}N_4O_4^+$: 523.3279, found [M+H]$^+$: 523.3271; **FTIR** (neat) ν_{max} 2917, 1733, 1693, 1599, 1423, 1246, 1166, 1136, 1080, 757 cm^{-1}.

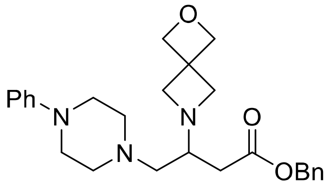

Benzyl 4-(4-phenylpiperazin-1-yl)-3-(2-oxa-6-azaspiro[3.3]heptan-6-yl)butanoate (10)

Following the general procedure A, using allenoate **2** (17.4 mg, 0.1 mmol, 1.0 equiv), 2-oxa-6-azaspiro[3.3]heptane (11.0 mg, 0.11 mmol, 1.1 equiv) and 1-phenylpiperazine (24.3 mg, 0.15 mmol, 1.5 equiv). The reaction time of the first amination step was 8 h. The product was isolated by silica gel chromatography as a colorless oil (29.2 mg, 67% yield). **^{1}H NMR** (500 MHz, $CDCl_3$) 7.38 – 7.28 (m, 5H), 7.29 – 7.22 (m, 2H), 6.93 – 6.87 (m, 2H), 6.85 (t, *J* = 7.3 Hz, 1H), 5.18 – 5.05 (m, 2H), 4.70 (s, 4H), 3.44 – 3.26 (m, 5H), 3.16 – 3.03 (m, 4H), 2.87 – 2.77 (m, 1H), 2.66 – 2.49 (m, 4H), 2.45 – 2.30 (m, 3H), 2.26 – 2.13 (m, 1H); **^{13}C NMR** (126 MHz, $CDCl_3$) δ 172.2, 151.4, 136.0, 129.2,

128.7, 128.5, 128.4, 119.7, 116.1, 81.3, 66.5, 62.6, 61.4, 60.6, 54.0, 49.3, 38.9, 36.7; **HRMS** (ESI/[M+H]$^+$) calcd. for $C_{26}H_{34}N_3O_3^+$: 436.2595, found [M+H]$^+$: 436.2591; **FTIR** (neat) ν_{max} 2943, 2820, 1731, 1599, 1497, 1454, 1384, 1292, 1233, 1150, 1009, 971, 757, 695 cm^{-1}.

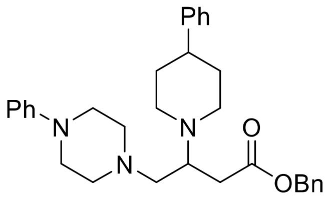

Benzyl 4-(4-phenylpiperazin-1-yl)-3-(4-phenylpiperidin-1-yl)butanoate (11)

Following the general procedure A, using allenoate **2** (17.4 mg, 0.1 mmol, 1.0 equiv), 4-phenylpiperidine (17.7 mg, 0.11 mmol, 1.1 equiv) and 1-phenylpiperazine (24.3 mg, 0.15 mmol, 1.5 equiv). The reaction time of the first amination step was 6 h. The product was isolated by silica gel chromatography as a colorless oil (30.8 mg, 62% yield). **^{1}H NMR** (500 MHz, $CDCl_3$) δ 7.41 – 7.34 (m, 4H), 7.33 – 7.25 (m, 5H), 7.24 – 7.18 (m, 3H), 6.93 (d, *J* = 8.1 Hz, 2H), 6.87 (t, *J* = 7.3 Hz, 1H), 5.20 – 5.09 (m, 2H), 3.49 – 3.38 (m, 1H), 3.22 – 3.08 (m, 4H), 2.93 (dd, *J* = 31.2, 10.9 Hz, 2H), 2.81 – 2.71 (m, 2H), 2.67 – 2.58 (m, 2H), 2.58 – 2.50 (m, 4H), 2.49 – 2.39 (m, 3H), 1.87 – 1.78 (m, 2H), 1.75 – 1.62 (m, 2H); **^{13}C NMR** (126 MHz, $CDCl_3$) δ 173.0, 151.5, 146.5, 136.3, 129.2, 128.6, 128.5, 128.4, 128.3, 127.0, 126.2, 119.7, 116.1, 66.3, 60.0, 59.1, 53.8, 49.9, 49.8, 49.3, 43.1, 35.6, 34.2, 34.0; **HRMS** (ESI/[M+H]$^+$) calcd. for $C_{32}H_{40}N_3O_2^+$: 498.3115, found [M+H]$^+$: 498.3103; **FTIR** (neat) ν_{max} 2934, 2817, 1731, 1599, 1495, 1452, 1381, 1234, 1136, 1007, 756, 696 cm^{-1}.

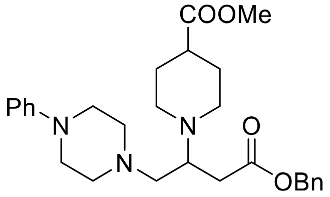

Methyl 1-(4-(benzyloxy)-4-oxo-1-(4-phenylpiperazin-1-yl)butan-2-yl)piperidine-4-carboxylate (12)

Following the general procedure A, using allenoate **2** (17.4 mg, 0.1 mmol, 1.0 equiv), methyl piperidine-4-carboxylate (15.7 mg, 0.11 mmol, 1.1 equiv) and 1-phenylpiperazine (24.3 mg, 0.15 mmol, 1.5 equiv). The reaction time of the first amination step was 6 h. The product was isolated by silica gel chromatography as a colorless oil (38.8 mg, 81% yield). **^{1}H NMR** (500 MHz, $CDCl_3$) δ 7.42 – 7.29 (m, 5H), 7.28 (t, *J* = 7.9 Hz, 2H), 6.92 (d, *J* = 8.2 Hz, 2H), 6.87 (t, *J* = 7.3 Hz, 1H), 5.18 – 5.09 (m, 2H), 3.69 (s, 3H), 3.42 – 3.31 (m, 1H), 3.19 – 3.05 (m, 4H), 2.90 – 2.68 (m, 4H), 2.59 – 2.42 (m, 6H), 2.40 – 2.22 (m, 3H), 1.92 – 1.81 (m, 2H), 1.74 – 1.60 (m, 2H); **^{13}C NMR** (126 MHz, $CDCl_3$) δ 175.7, 172.8, 151.4, 136.2, 129.2, 128.6, 128.4, 128.2, 119.7, 116.0, 66.3, 59.5, 59.0, 53.7, 51.7, 49.3, 48.9, 48.2, 41.5, 35.7, 28.9, 28.8; **HRMS** (ESI/[M+H]$^+$) calcd. for $C_{28}H_{38}N_3O_4^+$: 480.2857, found [M+H]$^+$: 480.2857; **FTIR** (neat) ν_{max} 2947, 2817, 1731, 1599, 1497, 1448, 1380, 1337, 1294, 1233, 1194, 1135, 1045, 1009, 971, 757, 695 cm^{-1}.

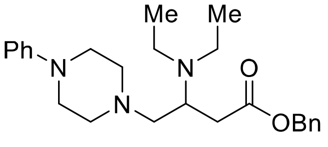

Benzyl 3-(diethylamino)-4-(4-phenylpiperazin-1-yl)butanoate (13)

Following the general procedure A, using allenoate **2** (17.4 mg, 0.1 mmol, 1.0 equiv), diethylamine (9.0 μL, 0.11 mmol, 1.1 equiv) and 1-phenylpiperazine (24.3 mg, 0.15 mmol, 1.5 equiv). The reaction time of the first amination step was 4 h. The product was isolated by silica gel chromatography as a colorless oil (32.4 mg, 79% yield). **^{1}H NMR** (500 MHz, $CDCl_3$) δ 7.41 – 7.22 (m, 7H), 6.94 – 6.89 (m, 1H), 6.85 (tt, *J* = 7.2, 0.9 Hz, 1H), 5.17 – 5.04 (m, 2H), 3.57 – 3.46 (m, 1H), 3.17 – 3.06 (m, 4H), 2.77 – 2.69 (m, 2H), 2.58 – 2.34 (m, 10H), 1.04 (t, *J* = 7.1 Hz, 6H); **^{13}C NMR** (126 MHz, $CDCl_3$) δ 173.1, 151.5, 136.3, 129.2, 128.6, 128.3, 128.2, 119.6, 116.0, 66.2, 60.4, 54.6, 53.9, 49.3, 44.1, 36.2, 14.7; **HRMS** (ESI/[M+H]$^+$) calcd. for $C_{25}H_{36}N_3O_2^+$: 410.2802, found [M+H]$^+$: 410.2802; **FTIR** (neat) ν_{max} 2969, 2817, 1732, 1599, 1500, 1453, 1381, 1294, 1232, 1136, 1009, 756, 693 cm^{-1}.

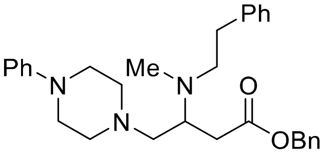

Benzyl 3-(methyl(phenethyl)amino)-4-(4-phenylpiperazin-1-yl)butanoate (14)

Following the general procedure A, using allenoate **2** (17.4 mg, 0.1 mmol, 1.0 equiv), *N*-methyl-2-phenylethan-1-amine (16.0 μL, 0.11 mmol, 1.1 equiv) and 1-phenylpiperazine (24.3 mg, 0.15 mmol, 1.5 equiv). The reaction time of the first amination step was 8 h. The product was isolated by silica gel chromatography as a colorless oil (35.4 mg, 75% yield). **^{1}H NMR** (500 MHz, $CDCl_3$) δ 7.37 – 7.32 (m, 4H), 7.32 – 7.24 (m, 5H), 7.23 – 7.18 (m, 3H), 6.94 – 6.89 (m, 2H), 6.88 – 6.83 (m, 1H), 5.16 – 5.04 (m, 2H), 3.50 – 3.42 (m, 1H), 3.16 – 3.06 (m, 4H), 2.80 – 2.65 (m, 6H), 2.57 – 2.45 (m, 5H), 2.35 (s, 3H); **^{13}C NMR** (126 MHz, $CDCl_3$) δ 172.9, 151.4, 140.6, 136.2, 129.2, 128.9, 128.6, 128.4, 128.34, 128.26, 126.1, 119.7, 116.1, 66.3, 59.8, 57.9, 56.2, 53.8, 49.3, 37.4, 35.41, 35.35; **HRMS** (ESI/[M+H]$^+$) calcd. for $C_{30}H_{38}N_3O_2^+$: 472.2959, found [M+H]$^+$: 472.2968; **FTIR** (neat) ν_{max} 2943, 2820, 1731, 1599, 1496, 1454, 1384, 1337, 1294, 1231, 1136, 1009, 925, 753, 696 cm^{-1}.

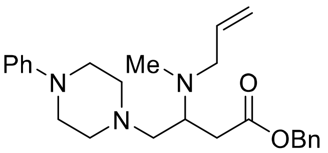

Benzyl 3-(allyl(methyl)amino)-4-(4-phenylpiperazin-1-yl)butanoate (15)

Following the general procedure A, using allenoate **2** (17.4 mg, 0.1 mmol, 1.0 equiv), *N*-allylmethylamine (11.0 μL, 0.11 mmol, 1.1 equiv) and 1-phenylpiperazine (24.3 mg, 0.15 mmol, 1.5 equiv). The reaction time of the first amination step was 6 h. The product was isolated by silica gel chromatography as a colorless oil (24.5 mg, 60% yield). **^{1}H NMR** (500 MHz, $CDCl_3$) δ 7.40 – 7.28

(m, 5H), 7.29 – 7.23 (m, 2H), 6.91 (d, *J* = 7.9 Hz, 2H), 6.85 (t, *J* = 7.3 Hz, 1H), 5.79 (ddt, *J* = 16.6, 10.1, 6.3 Hz, 1H), 5.22 – 5.00 (m, 4H), 3.50 – 3.41 (m, 1H), 3.22 – 3.03 (m, 6H), 2.69 (dq, *J* = 10.3, 5.1, 4.5 Hz, 2H), 2.58 – 2.45 (m, 5H), 2.35 (dd, *J* = 12.4, 9.2 Hz, 1H), 2.24 (s, 3H); **^{13}C NMR** (126 MHz, $CDCl_3$) δ 172.8, 151.5, 136.8, 136.2, 129.2, 128.6, 128.33, 128.25, 119.7, 117.0, 116.1, 66.3, 59.7, 57.6, 56.7, 53.8, 49.3, 36.9, 35.4; **HRMS** (ESI/[M+H]$^+$) calcd. for $C_{25}H_{34}N_3O_2^+$: 408.2646, found [M+H]$^+$: 408.2648; **FTIR** (neat) ν_{max} 3361, 2941, 2817, 1731, 1599, 1497, 1454, 1382, 1337, 1295, 1232, 1135, 1030, 1009, 923, 755, 693 cm^{-1}.

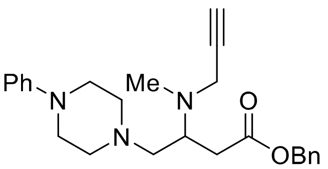

Benzyl 3-(methyl(prop-2-yn-1-yl)amino)-4-(4-phenylpiperazin-1-yl)butanoate (16)

Following the general procedure A, using allenoate **2** (17.4 mg, 0.1 mmol, 1.0 equiv), *N*-methylprop-2-yn-1-amine (7.6 mg, 0.11 mmol, 1.1 equiv) and 1-phenylpiperazine (24.3 mg, 0.15 mmol, 1.5 equiv). The reaction time of the first amination step was 6 h. The product was isolated by silica gel chromatography as a colorless oil (27.6 mg, 68% yield). **^{1}H NMR** (500 MHz, $CDCl_3$) δ 7.38 – 7.31 (m, 4H), 7.26 (t, *J* = 8.0 Hz, 3H), 6.95 – 6.87 (m, 2H), 6.89 – 6.81 (m, 1H), 5.17 – 5.07 (m, 2H), 3.57 – 3.48 (m, 1H), 3.49 – 3.37 (m, 2H), 3.22 – 3.04 (m, 5H), 2.76 – 2.47 (m, 8H), 2.37 (s, 3H); **^{13}C NMR** (126 MHz, $CDCl_3$) δ 172.5, 151.4, 136.2, 129.2, 128.6, 128.4, 128.3, 119.7, 116.1, 80.8, 72.8, 66.4, 60.1, 56.9, 53.8, 49.3, 43.8, 36.9, 35.8; **HRMS** (ESI/[M+H]$^+$) calcd. for $C_{25}H_{32}N_3O_2^+$: 406.2489, found [M+H]$^+$: 406.2486; **FTIR** (neat) ν_{max} 3294, 2818, 1731, 1599, 1499, 1453, 1383, 1232, 1136, 1009, 757, 695 cm^{-1}.

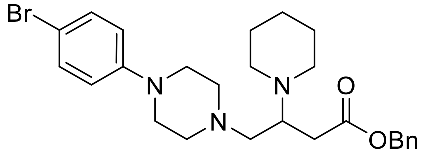

Benzyl 4-(4-(4-bromophenyl)piperazin-1-yl)-3-(piperidin-1-yl)butanoate (17)

Following the general procedure A, using allenoate **2** (17.4 mg, 0.1 mmol, 1.0 equiv), piperidine (11.0 µL, 0.11 mmol, 1.1 equiv) and 1-(4-bromophenyl)piperazine (36.2 mg, 0.15 mmol, 1.5 equiv). The reaction time of the first amination step was 4 h. The product was isolated by silica gel chromatography as a colorless oil (30.5 mg, 61% yield). **^{1}H NMR** (500 MHz, $CDCl_3$) δ 7.39 – 7.27 (m, 7H), 6.79 – 6.71 (m, 2H), 5.25 – 4.96 (m, 2H), 3.38 – 3.20 (m, 1H), 3.14 – 2.96 (m, 4H), 2.71 (dq, J = 10.5, 5.9, 4.5 Hz, 2H), 2.62 – 2.31 (m, 10H), 1.50 (q, *J* = 5.5 Hz, 4H), 1.40 (q, *J* = 5.7 Hz, 2H); **^{13}C NMR** (126 MHz, $CDCl_3$) δ 173.2, 150.5, 136.3, 132.0, 128.7, 128.35, 128.28, 117.6, 111.7, 66.3, 60.0, 59.5, 53.6, 50.2, 49.2, 35.4, 26.7, 24.9; **HRMS** (ESI/[M+H]$^+$) calcd. for $C_{26}H_{35}BrN_3O_2^+$: 500.1907, found [M+H]$^+$: 500.1905; **FTIR** (neat) ν_{max} 2932, 1733, 1493, 1453, 1235, 1151, 1136, 891, 808 cm^{-1}.

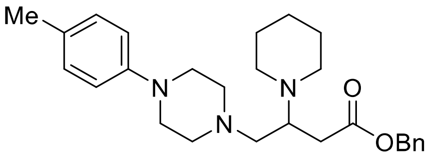

Benzyl 3-(piperidin-1-yl)-4-(4-(*p*-tolyl)piperazin-1-yl)butanoate (18)

Following the general procedure A, using allenoate **2** (17.4 mg, 0.1 mmol, 1.0 equiv), piperidine (11.0 μL, 0.11 mmol, 1.1 equiv) and 1-(*p*-tolyl)piperazine (26.4 mg, 0.15 mmol, 1.5 equiv). The reaction time of the first amination step was 4 h. The product was isolated by silica gel chromatography as a colorless oil (25.3 mg, 58% yield). **^{1}H NMR** (500 MHz, $CDCl_3$) δ 7.45 – 7.26 (m, 5H), 7.07 (d, *J* = 8.4 Hz, 2H), 6.85 – 6.81 (m, 2H), 5.18 – 5.05 (m, 2H), 3.37 – 3.26 (m, 1H), 3.01 – 3.11 (m, 4H), 2.76 – 2.68 (m, 2H), 2.68 – 2.32 (m, 10H), 2.27 (s, 3H), 1.51 (q, *J* = 5.6 Hz, 4H), 1.41 (q, *J* = 5.5 Hz, 2H); **^{13}C NMR** (126 MHz, $CDCl_3$) δ 173.1, 149.4, 136.3, 129.7, 129.1, 128.6, 128.3, 128.2, 116.4, 66.2, 60.0, 59.4, 53.8, 50.2, 49.9, 35.4, 26.7, 24.9, 20.5; **HRMS** (ESI/[M+H]$^+$) calcd. for $C_{27}H_{38}N_3O_2^+$: 436.2959, found [M+H]$^+$: 436.2964; **FTIR** (neat) v_{max} 2932, 2817, 1733, 1515, 1453, 1238, 1151, 1008, 808 cm^{-1}.

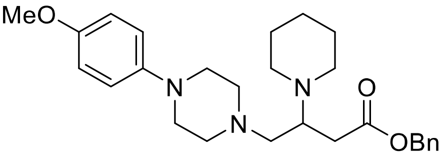

Benzyl 4-(4-(4-methoxyphenyl)piperazin-1-yl)-3-(piperidin-1-yl)butanoate (19)

Following the general procedure A, using allenoate **2** (17.4 mg, 0.1 mmol, 1.0 equiv), piperidine (11.0 μL, 0.11 mmol, 1.1 equiv) and 1-(4-methoxyphenyl)piperazine (28.8 mg, 0.15 mmol, 1.5 equiv). The reaction time of the first amination step was 4 h. The product was isolated by silica gel chromatography as a colorless oil (27.2 mg, 60% yield). **^{1}H NMR** (500 MHz, $CDCl_3$) δ 7.40 – 7.27 (m, 5H), 6.91 – 6.80 (m, 4H), 5.24 – 5.03 (m, 2H), 3.76 (s, 3H), 3.38 – 3.24 (m, 1H), 3.05 – 2.95 (m, 4H), 2.78 – 2.69 (m, 2H), 2.61 – 2.36 (m, 10H), 1.50 (q, *J* = 5.6 Hz, 4H), 1.40 (q, *J* = 5.7 Hz, 2H); **^{13}C NMR** (126 MHz, $CDCl_3$) δ 173.1, 153.8, 145.9, 136.3, 128.6, 128.3, 128.2, 118.1, 114.5, 66.2, 59.9, 59.4, 55.7, 53.9, 50.8, 50.2, 35.4, 26.6, 24.9; **HRMS** (ESI/[M+H]$^+$) calcd. for $C_{27}H_{38}N_3O_3^+$: 452.2908, found [M+H]$^+$: 452.2911; **FTIR** (neat) v_{max} 2932, 2815, 1731, 1510, 1497, 1454, 1379, 1293, 1241, 1151, 1135, 1036, 1012, 822, 742, 698 cm^{-1}.

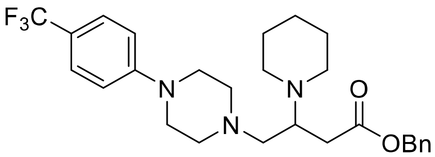

Benzyl 3-(piperidin-1-yl)-4-(4-(4-(trifluoromethyl)phenyl)piperazin-1-yl)butanoate (20)

Following the general procedure A, using allenoate **2** (17.4 mg, 0.1 mmol, 1.0 equiv), piperidine (11.0 μL, 0.11 mmol, 1.1 equiv) and 1-(4-(trifluoromethyl)phenyl)piperazine (34.5 mg, 0.15 mmol, 1.5 equiv). The reaction time of the first amination step was 4 h. The product was isolated by silica gel chromatography as a colorless oil (30.8 mg, 63% yield). **^{1}H NMR** (500 MHz, $CDCl_3$) δ 7.47 (d,

J = 8.7 Hz, 2H), 7.39 – 7.26 (m, 5H), 6.88 (d, J = 8.8 Hz, 2H), 5.32 – 4.90 (m, 2H), 3.36 – 3.24 (m, 1H), 3.23 – 3.07 (m, 4H), 2.75 – 2.68 (m, 2H), 2.65 – 2.34 (m, 10H), 1.58 – 1.47 (m, 4H), 1.41 (q, J = 5.9 Hz, 2H); **^{13}C NMR** (126 MHz, $CDCl_3$) δ 173.1, 153.5, 136.3, 128.6, 128.3, 126.5 (q, J = 3.8 Hz), 124.9 (q, J = 270.9 Hz), 120.4 (q, J = 32.7 Hz), 114.5, 66.3, 59.9, 59.4, 53.5, 50.2, 48.1, 35.3, 26.6, 24.9; **^{19}F NMR** (471 MHz, $CDCl_3$) δ −62.44; **HRMS** (ESI/[M+H]$^+$) calcd. for $C_{27}H_{35}F_3N_3O_2^+$: 490.2676, found [M+H]$^+$: 490.2674; **FTIR** (neat) ν_{max} 2933, 2818, 1732, 1614, 1524, 1454, 1386, 1329, 1236, 1160, 1111, 1071, 1001, 823, 742, 697 cm^{-1}.

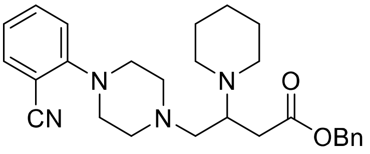

Benzyl 4-(4-(2-cyanophenyl)piperazin-1-yl)-3-(piperidin-1-yl)butanoate (21)

Following the general procedure A, using allenoate **2** (17.4 mg, 0.1 mmol, 1.0 equiv), piperidine (11.0 μL, 0.11 mmol, 1.1 equiv) and 4-(piperazin-1-yl)benzonitrile (28.1 mg, 0.15 mmol, 1.5 equiv). The reaction time of the first amination step was 4 h. The product was isolated by silica gel chromatography as a colorless oil (25.7 mg, 57% yield). **^{1}H NMR** (500 MHz, $CDCl_3$) δ 7.54 (dd, J = 7.7, 1.7 Hz, 1H), 7.49 – 7.42 (m, 1H), 7.40 – 7.26 (m, 5H), 7.02 – 6.92 (m, 2H), 5.17 – 5.08 (m, 2H), 3.34 – 3.23 (m, 1H), 3.13 (t, J = 4.8 Hz, 4H), 2.82 – 2.71 (m, 2H), 2.61 – 2.34 (m, 10H), 1.58 – 1.44 (m, 4H), 1.44 – 1.34 (m, 2H); **^{13}C NMR** (126 MHz, $CDCl_3$) δ 173.1, 155.8, 136.3, 134.5, 133.8, 128.6, 128.3, 128.2, 121.6, 118.7, 118.6, 105.9, 66.2, 59.5, 59.5, 53.7, 51.7, 50.2, 35.5, 26.6, 24.9; **HRMS** (ESI/[M+H]$^+$) calcd. for $C_{27}H_{35}N_4O_2^+$: 447.2755, found [M+H]$^+$: 447.2750; **FTIR** (neat) ν_{max} 2932, 2818, 1731, 1595, 1488, 1446, 1378, 1347, 1293, 1230, 1150, 1134, 1009, 757, 698 cm^{-1}.

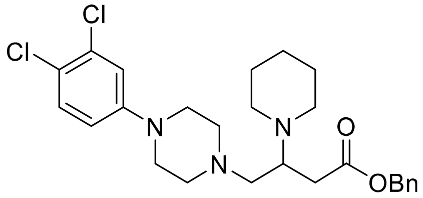

Benzyl 4-(4-(3,4-dichlorophenyl)piperazin-1-yl)-3-(piperidin-1-yl)butanoate (22)

Following the general procedure A, using allenoate **2** (17.4 mg, 0.1 mmol, 1.0 equiv), piperidine (11.0 μL, 0.11 mmol, 1.1 equiv) and 1-(3,4-dichlorophenyl)piperazine (34.7 mg, 0.15 mmol, 1.5 equiv). The reaction time of the first amination step was 4 h. The product was isolated by silica gel chromatography as a colorless oil (31.7 mg, 64% yield). **^{1}H NMR** (500 MHz, $CDCl_3$) δ 7.39 – 7.25 (m, 5H), 7.25 (d, J = 8.9 Hz, 1H), 6.90 (d, J = 2.9 Hz, 1H), 6.69 (dd, J = 8.9, 2.9 Hz, 1H), 5.15 – 5.06 (m, 2H), 3.34 – 3.24 (m, 1H), 3.11 – 2.98 (m, 4H), 2.73 – 2.64 (m, 2H), 2.62 – 2.34 (m, 10H), 1.58 – 1.46 (m, 4H), 1.44 – 1.36 (m, 2H); **^{13}C NMR** (126 MHz, $CDCl_3$) δ 173.1, 150.8, 136.3, 132.8, 130.5, 128.6, 128.32, 128.26, 122.0, 117.2, 115.3, 66.3, 59.9, 59.4, 53.4, 50.2, 48.8, 35.3, 26.6, 24.9; **HRMS** (ESI/[M+H]$^+$) calcd. for $C_{26}H_{34}Cl_2N_3O_2^+$: 490.2023, found [M+H]$^+$: 490.2027; **FTIR** (neat) ν_{max} 2932, 2818, 1732, 1593, 1484, 1453, 1382, 1237, 1151, 1137, 1009, 951, 699 cm^{-1}.

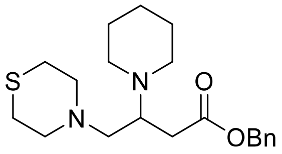

Benzyl 3-(piperidin-1-yl)-4-thiomorpholinobutanoate (23)

Following the general procedure A, using allenoate **2** (17.4 mg, 0.1 mmol, 1.0 equiv), piperidine (11.0 μL, 0.11 mmol, 1.1 equiv) and thiomorpholine (15.5 mg, 0.15 mmol, 1.5 equiv). The reaction time of the first amination step was 4 h. The product was isolated by silica gel chromatography as a colorless oil (21.0 mg, 58% yield). **^{1}H NMR** (500 MHz, $CDCl_3$) δ 7.44 – 7.27 (m, 5H), 5.17 – 5.07 (m, 2H), 3.34 – 3.20 (m, 1H), 2.87 – 2.73 (m, 1H), 2.70 – 2.33 (m, 11H), 2.33 – 2.15 (m, 2H), 1.50 (bs, 5H), 1.44 – 1.32 (m, 3H); **^{13}C NMR** (126 MHz, $CDCl_3$) δ 173.1, 136.3, 128.7, 128.3, 128.2, 66.3, 60.7, 59.5, 55.8, 50.2, 35.3, 28.1, 26.6, 24.9; **HRMS** (ESI/[M+H]$^+$) calcd. for $C_{20}H_{31}N_2O_2S^+$: 363.2101, found [M+H]$^+$: 363.2100; **FTIR** (neat) ν_{max} 2933, 2851, 2808, 1731, 1455, 1378, 1218, 1154, 1008, 1001, 957 cm^{-1}.

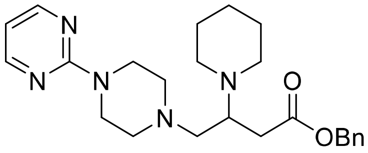

Benzyl 3-(piperidin-1-yl)-4-(4-(pyrimidin-2-yl)piperazin-1-yl)butanoate (24)

Following the general procedure A, using allenoate **2** (17.4 mg, 0.1 mmol, 1.0 equiv), piperidine (11.0 μL, 0.11 mmol, 1.1 equiv) and 2-(piperazin-1-yl)pyrimidine (24.6 mg, 0.15 mmol, 1.5 equiv). The reaction time of the first amination step was 4 h. The product was isolated by silica gel chromatography as a colorless oil (28.8 mg, 68% yield). **^{1}H NMR** (500 MHz, $CDCl_3$) δ 8.28 (d, *J* = 4.7 Hz, 2H), 7.39 – 7.25 (m, 5H), 6.45 (t, *J* = 4.7 Hz, 1H), 5.19 – 5.06 (m, 2H), 3.77 – 3.65 (m, 4H), 3.35 – 3.26 (m, 1H), 2.65 – 2.43 (m, 9H), 2.42 – 2.34 (m, 3H), 1.49 (q, *J* = 5.5 Hz, 4H), 1.39 (q, *J* = 5.9 Hz, 2H); **^{13}C NMR** (126 MHz, $CDCl_3$) δ 173.1, 161.8, 157.8, 136.3, 128.6, 128.3, 128.2, 109.8, 66.3, 60.0, 59.5, 53.7, 50.2, 43.9, 35.4, 26.6, 24.9; **HRMS** (ESI/[M+H]$^+$) calcd. for $C_{24}H_{34}N_5O_2^+$: 424.2707, found [M+H]$^+$: 424.2706; **FTIR** (neat) ν_{max} 2932, 1733, 1585, 1546, 1500, 1447, 1359, 1259, 983 cm^{-1}.

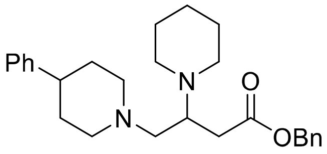

Benzyl 4-(4-phenylpiperidin-1-yl)-3-(piperidin-1-yl)butanoate (25)

Following the general procedure A, using allenoate **2** (17.4 mg, 0.1 mmol, 1.0 equiv), piperidine (11.0 μL, 0.11 mmol, 1.1 equiv) and 4-phenylpiperidine (24.2 mg, 0.15 mmol, 1.5 equiv). The reaction time of the first amination step was 4 h. The product was isolated by silica gel chromatography as a colorless oil (33.6 mg, 80% yield). **^{1}H NMR** (500 MHz, $CDCl_3$) δ 7.40 – 7.27 (m, 7H), 7.22 – 7.17 (m, 3H), 5.19 – 5.10 (m, 2H), 3.33 (s, 1H), 3.11 (d, *J* = 11.2 Hz, 1H), 2.91 (d,

J = 11.3 Hz, 1H), 2.64 – 2.22 (m, 10H), 2.01 (s, 1H), 1.84 – 1.64 (m, 4H), 1.58 – 1.48 (m, 4H), 1.41 (q, J = 6.0 Hz, 2H); **^{13}C NMR** (126 MHz, $CDCl_3$) δ 173.1, 146.6, 136.4, 128.6, 128.5, 128.3, 128.2, 126.9, 126.2, 66.3, 59.7, 56.1, 53.8, 50.2, 42.6, 33.8, 33.5, 26.5, 24.9; **HRMS** (ESI/[M+H]$^+$) calcd. for $C_{27}H_{37}N_2O_2^+$: 421.2850, found [M+H]$^+$: 421.2840; **FTIR** (neat) ν_{max} 2932, 2849, 2796, 1732, 1494, 1452, 1378, 1346, 1260, 1216, 1153, 1135, 1008, 993, 751, 698 cm^{-1}.

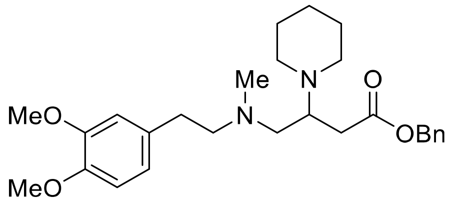

Benzyl 4-((3,4-dimethoxyphenethyl)(methyl)amino)-3-(piperidin-1-yl)butanoate (26)

Following the general procedure A, using allenoate **2** (17.4 mg, 0.1 mmol, 1.0 equiv), piperidine (11.0 µL, 0.11 mmol, 1.1 equiv) and 2-(3,4-dimethoxyphenyl)-*N*-methylethan-1-amine (29.3 mg, 0.15 mmol, 1.5 equiv). The reaction time of the first amination step was 4 h. The product was isolated by silica gel chromatography as a colorless oil (23.7 mg, 52% yield). **^{1}H NMR** (500 MHz, $CDCl_3$) δ 7.40 – 7.27 (m, 5H), 6.77 (d, J = 8.5 Hz, 1H), 6.74 – 6.67 (m, 2H), 5.22 – 5.02 (m, 2H), 3.85 (s, 3H), 3.84 (s, 3H), 3.27 – 3.18 (m, 1H), 2.70 – 2.58 (m, 3H), 2.57 – 2.48 (m, 5H), 2.48 – 2.42 (m, 3H), 2.40 – 2.33 (m, 1H), 2.27 (s, 3H), 1.53 – 1.45 (m, 4H), 1.41 – 1.34 (m, 2H); **^{13}C NMR** (126 MHz, $CDCl_3$) δ 173.2, 148.9, 147.3, 136.4, 133.3, 128.6, 128.3, 128.1, 120.6, 112.1, 111.3, 66.1, 60.5, 60.4, 58.5, 56.0, 55.9, 50.1, 42.8, 35.0, 33.5, 26.6, 24.9; **HRMS** (ESI/[M+H]$^+$) calcd. for $C_{27}H_{39}N_2O_4^+$: 455.2904, found [M+H]$^+$: 455.2901; **FTIR** (neat) ν_{max} 2932, 2848, 1732, 155, 1454, 1262, 1236, 1153, 1031, 807, 700 cm^{-1}.

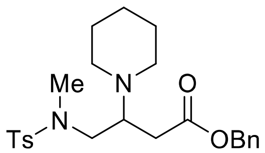

Benzyl 4-((*N*,4-dimethylphenyl)sulfonamido)-3-(piperidin-1-yl)butanoate (27)

Following the general procedure A, using allenoate **2** (17.4 mg, 0.1 mmol, 1.0 equiv), piperidine (11.0 µL, 0.11 mmol, 1.1 equiv) and *N*,4-dimethylbenzenesulfonamide (27.8 mg, 0.15 mmol, 1.5 equiv). The reaction time of the first amination step was 4 h. The reaction temperature of the second amination step was 60 °C. The product was isolated by silica gel chromatography as a colorless oil (31.2 mg, 70% yield). **^{1}H NMR** (500 MHz, $CDCl_3$) δ 7.65 (d, J = 8.3 Hz, 2H), 7.41 – 7.28 (m, 7H), 5.23 – 5.04 (m, 2H), 3.36 – 3.27 (m, 1H), 3.19 (dd, J = 13.1, 8.4 Hz, 1H), 2.87 (dd, J = 13.0, 6.0 Hz, 1H), 2.71 (s, 3H), 2.61 (dd, J = 15.0, 6.7 Hz, 1H), 2.53 – 2.46 (m, 5H), 2.42 (s, 3H), 1.52 – 1.43 (m, 4H), 1.41 – 1.34 (m, 2H); **^{13}C NMR** (126 MHz, $CDCl_3$) δ 172.7, 143.4, 136.1, 134.6, 129.8, 128.6, 128.5, 128.3, 127.6, 66.6, 59.9, 50.9, 50.0, 35.6, 33.7, 26.5, 24.8, 21.6; **HRMS** (ESI/[M+H]$^+$) calcd. for $C_{24}H_{33}N_2O_4S^+$: 445.2156, found [M+H]$^+$: 445.2154; **FTIR** (neat) ν_{max} 2932, 1732, 1454, 1341, 1161, 1110, 968, 741 cm^{-1}.

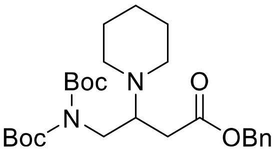

Benzyl 4-(bis(*tert*-butoxycarbonyl)amino)-3-(piperidin-1-yl)butanoate (28)

Following the general procedure A, using allenoate **2** (17.4 mg, 0.1 mmol, 1.0 equiv), piperidine (11.0 μL, 0.11 mmol, 1.1 equiv) and di-*tert*-butyl iminodicarbonate (32.6 mg, 0.15 mmol, 1.5 equiv). The reaction time of the first amination step was 4 h. The reaction temperature of the second amination step was 60 °C. The product was isolated by silica gel chromatography as a colorless oil (28.2 mg, 59% yield). ^{1}**H NMR** (500 MHz, $CDCl_3$) δ 7.39 – 7.29 (m, 5H), 5.12 (s, 2H), 3.81 (dd, *J* = 13.9, 7.1 Hz, 1H), 3.50 (dd, *J* = 13.9, 7.4 Hz, 1H), 3.40 – 3.33 (m, 1H), 2.57 (dd, *J* = 14.3, 7.9 Hz, 1H), 2.50 (s, 4H), 2.30 (dd, *J* = 14.3, 6.6 Hz, 1H), 1.49 (s, 18H), 1.47 – 1.43 (m, 4H), 1.38 – 1.33 (m, 2H); ^{13}**C NMR** (126 MHz, $CDCl_3$) δ 172.6, 152.9, 136.2, 128.6, 128.4, 128.2, 82.4, 66.4, 61.3, 49.9, 45.8, 34.2, 28.3, 26.6, 24.9; **HRMS** (ESI/[M+H]$^+$) calcd. for $C_{26}H_{41}N_2O_6^+$: 477.2959, found [M+H]$^+$: 477.2960; **FTIR** (neat) ν_{max} 2932, 2820, 1737, 1367, 1162, 1122, 755 cm^{-1}.

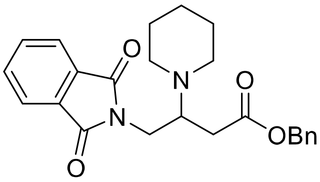

Benzyl 4-(1,3-dioxoisoindolin-2-yl)-3-(piperidin-1-yl)butanoate (29)

Following the general procedure A, using allenoate **2** (17.4 mg, 0.1 mmol, 1.0 equiv), piperidine (11.0 μL, 0.11 mmol, 1.1 equiv) and phthalimide (22.1 mg, 0.15 mmol, 1.5 equiv). The reaction time of the first amination step was 4 h. The reaction temperature of the second amination step was 60 °C. The product was isolated by silica gel chromatography as a colorless oil (23.6 mg, 58% yield). ^{1}**H NMR** (500 MHz, $CDCl_3$) δ 7.83 (dd, *J* = 5.4, 3.0 Hz, 2H), 7.70 (dd, *J* = 5.5, 3.0 Hz, 2H), 7.39 – 7.27 (m, 5H), 5.10 – 5.01 (m, 2H), 3.88 (dd, *J* = 13.7, 8.1 Hz, 1H), 3.65 – 3.57 (m, 1H), 3.56 – 3.47 (m, 1H), 2.68 (dd, *J* = 14.7, 6.8 Hz, 1H), 2.59 (dt, *J* = 10.5, 4.8 Hz, 2H), 2.44 (dt, *J* = 10.6, 5.1 Hz, 2H), 2.33 (dd, *J* = 14.7, 7.2 Hz, 1H), 1.41 – 1.29 (m, 6H); ^{13}**C NMR** (126 MHz, $CDCl_3$) δ 172.2, 168.4, 136.0, 134.0, 132.3, 128.6, 128.4, 128.3, 123.3, 66.5, 59.9, 49.8, 38.7, 34.0, 26.6, 24.8; **HRMS** (ESI/[M+H]$^+$) calcd. for $C_{24}H_{27}N_2O_4^+$: 407.1965, found [M+H]$^+$: 407.1960; **FTIR** (neat) ν_{max} 2933, 1712, 1398, 1171, 1099, 1000, 723, 698 cm^{-1}.

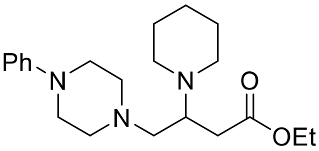

Ethyl 4-(4-phenylpiperazin-1-yl)-3-(piperidin-1-yl)butanoate (30)

Following the general procedure A, using allenoate **A1** (11.2 mg, 0.1 mmol, 1.0 equiv), piperidine (11.0 μL, 0.11 mmol, 1.1 equiv) and 1-phenylpiperazine (24.3 mg, 0.15 mmol, 1.5 equiv). The

reaction time of the first amination step was 4 h. The product was isolated by silica gel chromatography as a colorless oil (18.7 mg, 52% yield). **^{1}H NMR** (500 MHz, $CDCl_3$) δ 7.28 – 7.23 (m, 2H), 6.94 – 6.89 (m, 2H), 6.88 – 6.81 (m, 1H), 4.16 – 4.05 (m, 2H), 3.28 (dt, *J* = 13.9, 6.7 Hz, 1H), 3.17 – 3.08 (m, 4H), 2.77 – 2.71 (m, 2H), 2.60 – 2.47 (m, 8H), 2.43 – 2.35 (m, 2H), 1.53 (q, *J* = 5.4 Hz, 4H), 1.42 (q, *J* = 5.7 Hz, 2H), 1.25 (t, *J* = 7.1 Hz, 3H); **^{13}C NMR** (126 MHz, $CDCl_3$) δ 173.3, 151.5, 129.2, 119.7, 116.1, 60.3, 60.1, 59.4, 53.8, 50.2, 49.4, 35.4, 26.7, 24.9, 14.4; **HRMS** (ESI/[M+H]$^+$) calcd. for $C_{21}H_{34}N_3O_2^+$: 360.2646, found [M+H]$^+$: 360.2631; **FTIR** (neat) ν_{max} 2932, 2817, 1730, 1599, 1501, 1452, 1385, 1296, 1231, 1152, 1136, 1009, 927, 757, 690 cm^{-1}.

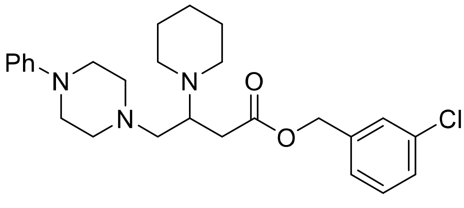

3-Chlorobenzyl 4-(4-phenylpiperazin-1-yl)-3-(piperidin-1-yl)butanoate (31)

Following the general procedure A, using allenoate **A2** (20.8 mg, 0.1 mmol, 1.0 equiv), piperidine (11.0 µL, 0.11 mmol, 1.1 equiv) and 1-phenylpiperazine (24.3 mg, 0.15 mmol, 1.5 equiv). The reaction time of the first amination step was 4 h. The product was isolated by silica gel chromatography as a colorless oil (25.8 mg, 56% yield). **^{1}H NMR** (500 MHz, $CDCl_3$) δ 7.33 – 7.23 (m, 6H), 6.90 (dd, *J* = 8.8, 0.9 Hz, 2H), 6.87 – 6.83 (m, 1H), 5.11 – 5.02 (m, 2H), 3.34 – 3.25 (m, 1H), 3.17 – 3.03 (m, 4H), 2.77 – 2.68 (m, 2H), 2.61 – 2.51 (m, 4H), 2.51 – 2.43 (m, 5H), 2.42 – 2.35 (m, 1H), 1.56 – 1.46 (m, 4H), 1.45 – 1.36 (m, 2H); **^{13}C NMR** (126 MHz, $CDCl_3$) δ 173.0, 151.5, 134.9, 134.1, 129.7, 129.2, 128.8, 119.7, 116.0, 65.4, 59.8, 59.5, 53.8, 50.2, 49.3, 35.5, 26.6, 24.9; **HRMS** (ESI/[M+H]$^+$) calcd. for $C_{26}H_{35}ClN_3O_2^+$: 456.2412, found [M+H]$^+$: 456.2404; **FTIR** (neat) ν_{max} 2932, 2817, 1732, 1599, 1494, 1452, 1231, 1151, 1135, 1010, 807, 757 cm^{-1}.

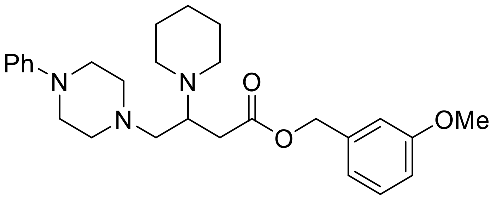

3-Methoxybenzyl 4-(4-phenylpiperazin-1-yl)-3-(piperidin-1-yl)butanoate (32)

Following the general procedure A, using allenoate **A3** (20.4 mg, 0.1 mmol, 1.0 equiv), piperidine (11.0 µL, 0.11 mmol, 1.1 equiv) and 1-phenylpiperazine (24.3 mg, 0.15 mmol, 1.5 equiv). The reaction time of the first amination step was 4 h. The product was isolated by silica gel chromatography as a colorless oil (30.7 mg, 68% yield). **^{1}H NMR** (500 MHz, $CDCl_3$) δ 7.32 – 7.22 (m, 3H), 6.99 – 6.89 (m, 4H), 6.86 (t, *J* = 7.2 Hz, 2H), 5.14 – 5.05 (m, 2H), 3.80 (s, 3H), 3.34 (bs, 1H), 3.17 – 3.08 (m, 4H), 2.80 – 2.70 (m, 2H), 2.67 – 2.40 (m, 10H), 1.55 (bs, 4H), 1.43 (q, *J* = 6.0 Hz, 2H); **^{13}C NMR** (126 MHz, $CDCl_3$) δ 173.1, 159.8, 151.5, 137.8, 129.7, 129.2, 120.5, 119.7, 116.1, 113.8, 113.7, 66.2, 59.4, 55.3, 53.8, 50.2, 49.3, 35.4, 29.8, 26.6, 24.9; **HRMS** (ESI/[M+H]$^+$)

calcd. for $C_{27}H_{38}N_3O_3^+$: 452.2908, found $[M+H]^+$: 452.2902; **FTIR** (neat) ν_{max} 2931, 1731, 1599, 1494, 1453, 1267, 1231, 1152, 1136, 1008, 757, 689 cm^{-1}.

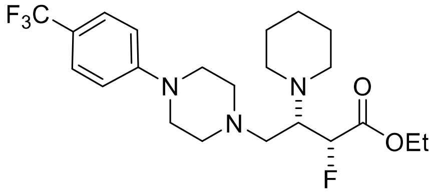

Ethyl 2-fluoro-3-(piperidin-1-yl)-4-(4-(4-(trifluoromethyl)phenyl)piperazin-1-yl)butanoate (33)

Following the general procedure B, using allenoate **A1** (11.2 mg, 0.1 mmol, 1.0 equiv), *N*-chloropiperidine (13.1 mg, 0.11 mmol, 1.1 equiv) and 1-(4-(trifluoromethyl)phenyl)piperazine (34.5 mg, 0.15 mmol, 1.5 equiv). The reaction time of the first amination step was 4 h. The enamine reduction was conducted under EtOH/AcOH, pH 4 at −20 °C. The product was isolated by silica gel chromatography as a colorless oil (21.0 mg, 47% yield, 10:1 d.r.). Major diastereomer: **^{1}H NMR** (500 MHz, $CDCl_3$) δ 7.47 (d, *J* = 8.6 Hz, 2H), 6.91 (d, *J* = 8.7 Hz, 2H), 5.23 (dd, *J* = 48.8, 3.6 Hz, 1H), 4.39 – 4.18 (m, 2H), 3.31 – 3.21 (m, 4H), 3.19 – 3.05 (m, 1H), 2.84 (dt, *J* = 10.6, 5.2 Hz, 2H), 2.72 (dt, *J* = 9.3, 7.6 Hz, 3H), 2.64 – 2.56 (m, 3H), 2.48 (dt, *J* = 10.6, 5.2 Hz, 2H), 1.51 – 1.42 (m, 4H), 1.40 – 1.29 (m, 5H); **^{13}C NMR** (126 MHz, $CDCl_3$) δ 169.8 (d, *J* = 24.3 Hz), 153.4, 126.5 (d, *J* = 3.4 Hz), 124.9 (q, *J* = 270.5 Hz), 120.6 (q, *J* = 32.7 Hz), 114.61, 92.0 (d, *J* = 190.7 Hz), 63.4 (d, *J* = 18.2 Hz), 61.2, 53.5, 52.6 (d, *J* = 4.2 Hz), 51.6, 48.3, 26.9, 24.6, 14.5; **^{19}F NMR** (471 MHz, $CDCl_3$) δ −62.52, −203.37 (dd, *J* = 48.8, 32.0 Hz); **HRMS** (ESI/$[M+H]^+$) calcd. for $C_{22}H_{32}F_4N_3O_2^+$: 446.2425, found $[M+H]^+$: 446.2425; **FTIR** (neat) ν_{max} 2932, 2850, 1762, 1737, 1615, 1524, 1453, 1387, 1329, 1298, 1235, 1200, 1149, 1113, 1071, 826, 755 cm^{-1}.

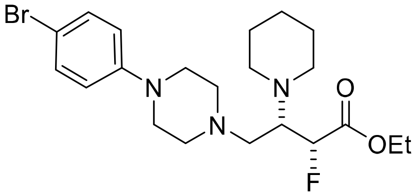

Ethyl 4-(4-(4-bromophenyl)piperazin-1-yl)-2-fluoro-3-(piperidin-1-yl)butanoate (34)

Following the general procedure B, using allenoate **A1** (11.2 mg, 0.1 mmol, 1.0 equiv), *N*-chloropiperidine (13.1 mg, 0.11 mmol, 1.1 equiv) and 1-(4-bromophenyl)piperazine (36.2 mg, 0.15 mmol, 1.5 equiv). The reaction time of the first amination step was 4 h. The enamine reduction was conducted under EtOH/AcOH, pH 4 at −20 °C. The product was isolated by silica gel chromatography as a colorless oil (18.3 mg, 40% yield, 12:1 d.r.). Major diastereomer: **^{1}H NMR** (500 MHz, $CDCl_3$) δ 7.37 – 7.30 (m, 2H), 6.81 – 6.74 (m, 2H), 5.22 (dd, *J* = 48.8, 3.5 Hz, 1H), 4.37 – 4.17 (m, 2H), 3.21 – 3.04 (m, 5H), 2.83 (dt, *J* = 10.5, 5.2 Hz, 2H), 2.76 – 2.66 (m, 3H), 2.58 (dt, *J* = 15.6, 5.3 Hz, 3H), 2.47 (dt, *J* = 10.5, 5.1 Hz, 2H), 1.52 – 1.42 (m, 4H), 1.40 – 1.31 (m, 5H); **^{13}C NMR** (126 MHz, $CDCl_3$) δ 169.8 (d, *J* = 24.2 Hz), 150.5, 132.0, 117.7, 111.9, 92.0 (d, *J* = 190.6 Hz), 63.4 (d, *J* = 18.3 Hz), 61.2 (s), 53.6, 52.6 (d, *J* = 4.2 Hz), 51.6, 49.3, 27.0, 24.6, 14.5; **^{19}F NMR** (471 MHz, $CDCl_3$) δ −203.38 (dd, *J* = 48.8, 32.0 Hz); **HRMS** (ESI/$[M+H]^+$) calcd. for $C_{21}H_{32}BrFN_3O_2^+$:

456.1656, found $[M+H]^+$: 456.1655; **FTIR** (neat) v_{max} 2931, 2818, 1761, 1736, 1589, 1494, 1452, 1297, 1233, 1146, 1127, 1094, 1033, 1010, 814 cm^{-1}.

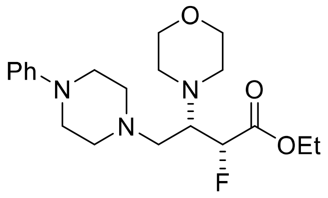

Ethyl 2-fluoro-3-morpholino-4-(4-phenylpiperazin-1-yl)butanoate (35)

Following the general procedure B, using allenoate **A1** (11.2 mg, 0.1 mmol, 1.0 equiv), *N*-chloromorpholine (13.3 mg, 0.11 mmol, 1.1 equiv) and 1-phenylpiperazine (24.3 mg, 0.15 mmol, 1.5 equiv). The reaction time of the first amination step was 7 h. The enamine reduction was conducted under EtOH/AcOH, pH 4 at −20 °C. The product was isolated by silica gel chromatography as a colorless oil (16.3 mg, 43% yield, 13:1 d.r.). Major diastereomer: **^{1}H NMR** (500 MHz, $CDCl_3$) δ 7.31 – 7.21 (m, 2H), 6.95 – 6.89 (m, 2H), 6.86 (t, *J* = 7.3 Hz, 1H), 5.24 (dd, *J* = 48.5, 3.2 Hz, 1H), 4.40 – 4.23 (m, 2H), 3.60 (t, *J* = 4.6 Hz, 4H), 3.27 – 3.09 (m, 5H), 2.99 – 2.89 (m, 2H), 2.79 – 2.67 (m, 4H), 2.67 – 2.60 (m, 2H), 2.60 – 2.52 (m, 2H), 1.34 (t, *J* = 7.1 Hz, 3H); **^{13}C NMR** (126 MHz, $CDCl_3$) δ 169.5 (d, *J* = 23.9 Hz), 151.4 (s), 129.3, 119.9, 116.2, 92.0 (d, *J* = 190.8 Hz), 67.8, 62.72 (d, *J* = 18.2 Hz), 61.3, 53.8, 52.64 (d, *J* = 4.0 Hz), 50.7, 49.5, 14.6; **^{19}F NMR** (471 MHz, $CDCl_3$) δ −204.04 (dd, *J* = 48.5, 32.2 Hz); **HRMS** (ESI/$[M+H]^+$) calcd. for $C_{20}H_{31}FN_3O_3^+$: 380.2344, found $[M+H]^+$: 380.2345; **FTIR** (neat) v_{max} 2956, 2823, 1761, 1737, 1600, 1496, 1454, 1372, 1291, 1231, 1215, 1154, 1117, 1009, 754 cm^{-1}.

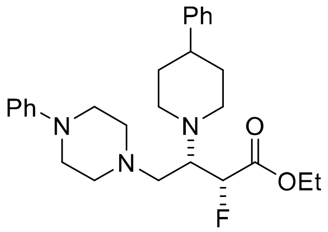

Ethyl 2-fluoro-4-(4-phenylpiperazin-1-yl)-3-(4-phenylpiperidin-1-yl)butanoate (36)

Following the general procedure B, using allenoate **A1** (11.2 mg, 0.1 mmol, 1.0 equiv), *N*-chloro-4-phenylpiperidine (21.5 mg, 0.11 mmol, 1.1 equiv) and 1-phenylpiperazine (24.3 mg, 0.15 mmol, 1.5 equiv). The reaction time of the first amination step was 6 h. The enamine reduction was conducted under EtOH/AcOH, pH 4 at −20 °C. The product was isolated by silica gel chromatography as a white solid (18.2 mg, 40% yield, 6:1 d.r., m.p. 97−100 °C). Major diastereomer: **^{1}H NMR** (500 MHz, $CDCl_3$) δ 7.31 – 7.25 (m, 4H), 7.23 – 7.15 (m, 3H), 6.94 (d, *J* = 7.9 Hz, 2H), 6.87 (t, *J* = 7.3 Hz, 1H), 5.26 (dd, *J* = 48.8, 3.5 Hz, 1H), 4.45 – 4.19 (m, 2H), 3.32 – 3.10 (m, 6H), 2.90 – 2.72 (m, 5H), 2.70 – 2.61 (m, 3H), 2.52 (td, *J* = 11.4, 2.1 Hz, 1H), 2.42 (tt, *J* = 12.1, 3.6 Hz, 1H), 1.83 – 1.73 (m, 2H), 1.70 – 1.57 (m, 2H), 1.36 (t, *J* = 7.1 Hz, 3H); **^{13}C NMR** (126 MHz, $CDCl_3$) δ 169.8 (d, *J* = 24.3 Hz), 151.4, 146.7, 129.3, 128.5, 126.9, 126.2, 119.9, 116.2, 92.0 (d, *J* = 190.5 Hz), 62.95 (d, *J* = 18.2 Hz), 61.3, 53.8, 53.6, 52.93 (d, *J* = 3.8 Hz), 49.5, 49.0 (d, *J* = 3.9 Hz), 42.7, 34.9, 33.9, 14.6; **^{19}F**

NMR (471 MHz, $CDCl_3$) δ −203.49 (dd, *J* = 48.7, 31.9 Hz); **HRMS** (ESI/[M+H]$^+$) calcd. for $C_{27}H_{37}FN_3O_3^+$: 454.2864, found [M+H]$^+$: 454.2865; **FTIR** (neat) ν_{max} 2932, 2817, 1763, 1731, 1599, 1494, 1452, 1371, 1297, 1231, 1147, 1094, 1029, 1011, 924, 757, 698 cm^{-1}.

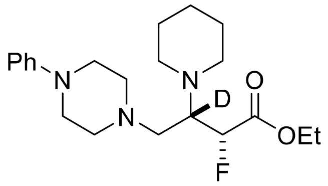

Ethyl 2-fluoro-4-(4-phenylpiperazin-1-yl)-3-(piperidin-1-yl)butanoate-3-*d* (37)

Following the general procedure A, using allenoate **A1** (11.2 mg, 0.1 mmol, 1.0 equiv), *N*-chloropiperidine (13.1 mg, 0.11 mmol, 1.1 equiv) and 1-phenylpiperazine (24.3 mg, 0.15 mmol, 1.5 equiv). The reaction time of the first amination step was 4 h. $NaBD_3CN$ was used instead of $NaBH_3CN$. The enamine reduction was conducted under EtOH/AcOH, pH 4 at −20 °C. The product was isolated by silica gel chromatography as a colorless oil (13.3 mg, 35% yield, 14:1 d.r.). Major diastereomer: **^{1}H NMR** (500 MHz, $CDCl_3$) δ 7.32 – 7.18 (m, 2H), 6.92 (dd, *J* = 8.7, 0.8 Hz, 2H), 6.85 (t, *J* = 7.3 Hz, 1H), 5.23 (d, *J* = 48.8 Hz, 1H), 4.39 – 4.14 (m, 2H), 3.24 – 3.07 (m, 4H), 2.84 (dt, *J* = 10.6, 5.2 Hz, 2H), 2.72 (dd, *J* = 11.5, 8.2 Hz, 3H), 2.65 – 2.54 (m, 3H), 2.48 (dt, *J* = 10.6, 5.2 Hz, 2H), 1.51 – 1.43 (m, 4H), 1.40 – 1.30 (m, 5H); **^{13}C NMR** (126 MHz, $CDCl_3$) δ 169.8 (d, *J* = 24.2 Hz), 151.5, 129.2, 119.8, 116.2, 92.0 (d, *J* = 190.2 Hz), 61.2, 53.8, 52.6 (d, *J* = 4.2 Hz), 51.5, 49.5, 27.0, 24.6, 14.5. The quaternary carbon connected with deuterium was omitted; **^{19}F NMR** (471 MHz, $CDCl_3$) δ −203.67 (d, *J* = 48.8 Hz); **HRMS** (ESI/[M+H]$^+$) calcd. for $C_{21}H_{32}DFN_3O_2^+$: 379.2614, found [M+H]$^+$: 379.2613; **FTIR** (neat) ν_{max} 2931, 2816, 1761, 1738, 1600, 1489, 1456, 1299, 1233, 1098, 1007, 755 cm^{-1}.

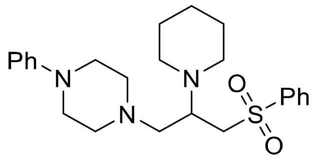

1-Phenyl-4-(3-(phenylsulfonyl)-2-(piperidin-1-yl)propyl)piperazine (38)

Following the general procedure B, using allene **A5** (18.0 mg, 0.1 mmol, 1.0 equiv), *N*-chloropiperidine (13.1 mg, 0.11 mmol, 1.1 equiv) and 1-phenylpiperazine (24.3 mg, 0.15 mmol, 1.5 equiv). The reaction time of the first amination step was 2 h. The product was isolated by silica gel chromatography as a colorless oil (25.2 mg, 59% yield). **^{1}H NMR** (500 MHz, $CDCl_3$) δ 8.00 – 7.85 (m, 2H), 7.70 – 7.56 (m, 1H), 7.58 – 7.41 (m, 2H), 7.34 – 7.18 (m, 2H), 6.92 (m, 2H), 6.85 (m, 1H), 3.43 (m, 1H), 3.39 – 3.30 (m, 2H), 3.15 (t, *J* = 5.0 Hz, 3H), 2.77 – 2.63 (m, 2H), 2.57 – 2.35 (m, 5H), 2.40 – 2.20 (m, 3H), 1.31 – 1.13 (m, 5H), 1.05 (m, 2H); **^{13}C NMR** (126 MHz, $CDCl_3$) δ 151.4, 141.2, 133.2, 129.3, 129.0, 128.2, 119.8, 116.2, 57.9, 56.4, 55.5, 53.6, 49.5, 49.4, 25.9, 24.6; **HRMS** (ESI/[M+H]$^+$) calcd. for $C_{24}H_{34}N_3O_2S^+$: 428.2366, found [M+H]$^+$: 428.2365; **FTIR** (neat) ν_{max} 2932, 2917, 1599, 1497, 1446, 1300, 1141, 752, 688 cm^{-1}.

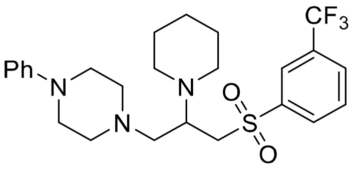

1-Phenyl-4-(2-(piperidin-1-yl)-3-((3-(trifluoromethyl)phenyl)sulfonyl)propyl)piperazine (39)

Following the general procedure B, using allene **A6** (24.8 mg, 0.1 mmol, 1.0 equiv), *N*-chloropiperidine (13.1 mg, 0.11 mmol, 1.1 equiv) and 1-phenylpiperazine (24.3 mg, 0.15 mmol, 1.5 equiv). The reaction time of the first amination step was 2 h. The product was isolated by silica gel chromatography as a colorless oil (21.8 mg, 44% yield). **^{1}H NMR** (500 MHz, $CDCl_3$) δ 8.20 (s, 1H), 8.11 (d, *J* = 7.7 Hz, 1H), 7.88 (d, *J* = 7.7 Hz, 1H), 7.69 (t, *J* = 7.8 Hz, 1H), 7.27 (t, *J* = 7.8 Hz, 2H), 6.92 (d, *J* = 8.1 Hz, 2H), 6.86 (t, *J* = 7.2 Hz, 1H), 3.57 (d, *J* = 12.5 Hz, 1H), 3.46 – 3.30 (m, 2H), 3.17 (t, *J* = 4.8 Hz, 3H), 2.83 – 2.63 (m, 2H), 2.60 – 2.37 (m, 4H), 2.36 – 2.16 (m, 3H), 1.35 – 1.07 (m, 6H), 0.91 (s, 2H); **^{13}C NMR** (126 MHz, $CDCl_3$) δ 151.4, 142.7, 131.7 (q, *J* = 33.4 Hz), 131.5, 129.8 (d, *J* = 3.1 Hz), 129.8, 129.3, 125.5 (d, *J* = 3.4 Hz), 123.4 (q, *J* = 273.1 Hz), 119.9, 116.3, 58.0, 55.7, 55.5, 53.6, 49.4, 49.3, 25.7, 24.4; **^{19}F NMR** (471 MHz, $CDCl_3$) δ −63.92; **HRMS** (ESI/[M+H]$^+$) calcd. for $C_{25}H_{33}F_3N_3O_2S^+$: 496.2240, found [M+H]$^+$: 496.2240; **FTIR** (neat) ν_{max} 2935, 2817, 1599, 1501, 1453, 1326, 1300, 1140, 769, 694 cm^{-1}.

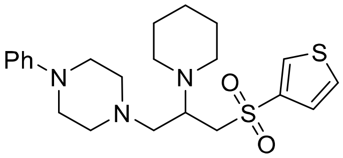

1-Phenyl-4-(2-(piperidin-1-yl)-3-(thiophen-2-ylsulfonyl)propyl)piperazine (40)

Following the general procedure B, using allene **A7** (18.6 mg, 0.1 mmol, 1.0 equiv), *N*-chloropiperidine (13.1 mg, 0.11 mmol, 1.1 equiv) and 1-phenylpiperazine (24.3 mg, 0.15 mmol, 1.5 equiv). The reaction time of the first amination step was 2 h. The product was isolated by silica gel chromatography as a colorless oil (21.2 mg, 49% yield). **^{1}H NMR** (500 MHz, $CDCl_3$) δ 7.70 (m, 2H), 7.43 – 7.20 (m, 2H), 7.16 (dd, *J* = 5.0, 3.8 Hz, 1H), 7.06 – 6.91 (m, 2H), 6.88 (tt, *J* = 7.3, 1.1 Hz, 1H), 3.59 – 3.50 (m, 1H), 3.52 – 3.33 (m, 2H), 3.18 (t, *J* = 5.0 Hz, 3H), 2.76 (m, 2H), 2.54 (dh, *J* = 18.3, 5.1 Hz, 5H), 2.45 – 2.20 (m, 4H), 1.41 – 1.14 (m, 6H); **^{13}C NMR** (126 MHz, $CDCl_3$) 151.5, 142.3, 133.7, 133.4, 129.3, 127.7, 119.9, 116.2, 58.1, 57.0, 56.6, 53.6, 49.7, 49.4, 26.3, 24.7; **HRMS** (ESI/[M+H]$^+$) calcd. for $C_{22}H_{32}N_3O_2S_2^+$: 434.1930, found [M+H]$^+$: 434.1928; **FTIR** (neat) ν_{max} 2931, 2817, 1599, 1305, 1282, 1137, 1010, 763 cm^{-1}.

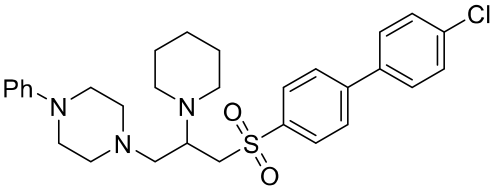

1-(3-((4'-Chloro-[1,1'-biphenyl]-4-yl)sulfonyl)-2-(piperidin-1-yl)propyl)-4-phenylpiperazine (41)

Following the general procedure B, using allene **A8** (29.0 mg, 0.1 mmol, 1.0 equiv), *N*-chloropiperidine (13.1 mg, 0.11 mmol, 1.1 equiv) and 1-phenylpiperazine (24.3 mg, 0.15 mmol, 1.5 equiv). The reaction time of the first amination step was 2 h. The product was isolated by silica gel chromatography as a colorless oil (47.9 mg, 89% yield). **^{1}H NMR** (500 MHz, $CDCl_3$) δ 7.98 (d, *J* = 8.3 Hz, 2H), 7.69 (d, *J* = 8.3 Hz, 2H), 7.55 – 7.48 (m, 2H), 7.48 – 7.42 (m, 2H), 7.32 – 7.22 (m, 2H), 6.91 (d, *J* = 7.9 Hz, 2H), 6.86 (t, *J* = 7.3 Hz, 1H), 3.48 (q, *J* = 7.3 Hz, 1H), 3.42 – 3.30 (m, 2H), 3.15 (s, 3H), 2.81 – 2.66 (m, 2H), 2.59 – 2.43 (m, 4H), 2.34 (dd, *J* = 19.0, 8.6 Hz, 3H), 1.25 (d, *J* = 6.6 Hz, 6H), 1.09 (s, 2H); **^{13}C NMR** (126 MHz, $CDCl_3$) δ 151.4, 145.0, 140.2, 138.1, 135.0, 129.5, 129.3, 128.9, 128.8, 127.6, 119.9, 116.2, 57.9, 56.4, 55.7, 53.6, 49.6, 49.4, 26.0, 24.6; **HRMS** (ESI/[M+H]$^+$) calcd. for $C_{30}H_{37}ClN_3O_2S^+$: 538.2289, found [M+H]$^+$: 538.2284; **FTIR** (neat) ν_{max} 2939, 2819, 1598, 1502, 1453, 1387, 1297, 1237, 1142, 1089, 820, 762 cm^{-1}.

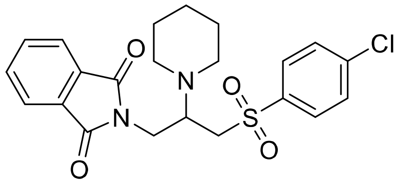

2-(3-((4-Chlorophenyl)sulfonyl)-2-(piperidin-1-yl)propyl)isoindoline-1,3-dione (42)

Following the general procedure B, using allene **A8** (21.5 mg, 0.1 mmol, 1.0 equiv), *N*-chloropiperidine (13.1 mg, 0.11 mmol, 1.1 equiv) and phthalimide (22.1 mg, 0.15 mmol, 1.5 equiv). The reaction time of the first amination step was 2 h. The product was isolated by silica gel chromatography as a colorless oil (18.8 mg, 42% yield). **^{1}H NMR** (500 MHz, $CDCl_3$) δ 7.89 (d, *J* = 8.5 Hz, 2H), 7.84 (dd, *J* = 5.4, 3.1 Hz, 2H), 7.72 (dd, *J* = 5.4, 3.0 Hz, 2H), 7.53 (d, *J* = 8.6 Hz, 2H), 3.89 (dd, *J* = 13.8, 7.5 Hz, 1H), 3.79 (dd, *J* = 13.9, 7.0 Hz, 1H), 3.53 – 3.37 (m, 2H), 3.06 (dd, *J* = 15.9, 8.0 Hz, 1H), 2.52 – 2.43 (m, 2H), 2.27 – 2.16 (m, 2H), 1.27 (d, *J* = 14.4 Hz, 6H); **^{13}C NMR** (126 MHz, $CDCl_3$) δ 168.4, 140.6, 138.0, 134.2, 132.2, 130.0, 129.7, 123.4, 58.2, 54.5, 49.5, 38.1, 26.0, 24.5; **HRMS** (ESI/[M+H]$^+$) calcd. for $C_{22}H_{24}ClN_2O_4S^+$: 447.1140, found [M+H]$^+$: 447.1138; **FTIR** (neat) ν_{max} 2933, 2953, 1713, 1396, 1304, 1145, 1087, 774, 715 cm^{-1}.

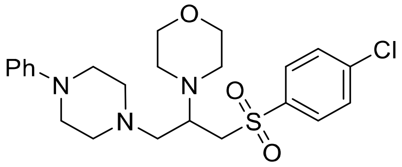

4-(1-((4-Chlorophenyl)sulfonyl)-3-(4-phenylpiperazin-1-yl)propan-2-yl)morpholine (43)

Following the general procedure B, using allene **A9** (21.5 mg, 0.1 mmol, 1.0 equiv), *N*-4-chloromorpholine (13.3 mg, 0.11 mmol, 1.1 equiv) and 1-phenylpiperazine (24.3 mg, 0.15 mmol, 1.5 equiv). The reaction time of the first amination step was 2 h. The product was isolated by silica gel chromatography as a colorless oil (19.5 mg, 42% yield). **^{1}H NMR** (500 MHz, $CDCl_3$) δ 7.90 –

7.82 (m, 2H), 7.58 – 7.49 (m, 2H), 7.30 – 7.23 (m, 2H), 6.92 (d, *J* = 7.8 Hz, 2H), 6.86 (t, *J* = 7.3 Hz, 1H), 3.47 (dd, *J* = 14.1, 2.6 Hz, 1H), 3.44 – 3.36 (m, 3H), 3.32 (dd, *J* = 14.1, 8.9 Hz, 1H), 3.30 – 3.22 (m, 2H), 3.16 (t, *J* = 4.9 Hz, 4H), 2.76 – 2.67 (m, 2H), 2.60 – 2.47 (m, 5H), 2.47 – 2.38 (m, 2H), 2.30 (dd, *J* = 12.5, 9.1 Hz, 1H); **^{13}C NMR** (126 MHz, $CDCl_3$) δ 151.3, 140.2, 139.7, 129.7, 129.5, 129.3, 120.0, 116.3, 66.9, 57.4, 56.3, 55.7, 53.6, 49.4, 48.7; **HRMS** (ESI/[M+H]$^+$) calcd. for $C_{23}H_{31}ClN_3O_3S^+$: 464.1769, found [M+H]$^+$: 464.1766; **FTIR** (neat) v_{max} 2923, 2822, 1599, 1496, 1303, 1147, 1087, 1004, 759 cm^{-1}.

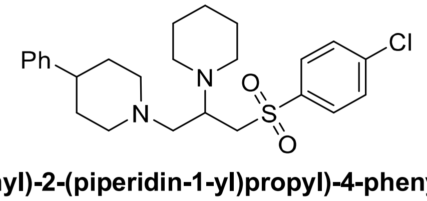

1-(3-((4-Chlorophenyl)sulfonyl)-2-(piperidin-1-yl)propyl)-4-phenylpiperidine (44)

Following the general procedure B, using allene **A9** (21.5 mg, 0.1 mmol, 1.0 equiv), *N*-chloropiperidine (13.1 mg, 0.11 mmol, 1.1 equiv) and 1-chloro-4-phenylpiperidine (29.2 mg, 0.15 mmol, 1.5 equiv). The reaction time of the first amination step was 2 h. The product was isolated by silica gel chromatography as a colorless oil (26.2 mg, 57% yield). **^{1}H NMR** (500 MHz, $CDCl_3$) δ 7.90 – 7.82 (m, 2H), 7.53 – 7.46 (m, 2H), 7.34 – 7.27 (m, 2H), 7.24 – 7.14 (m, 3H), 3.49 (t, *J* = 10.7 Hz, 1H), 3.40 – 3.24 (m, 2H), 3.04 (d, *J* = 11.1 Hz, 1H), 2.82 (d, *J* = 11.2 Hz, 1H), 2.53 – 2.40 (m, 4H), 2.35 – 2.27 (m, 2H), 2.26 – 2.16 (m, 2H), 1.97 (td, *J* = 11.7, 2.4 Hz, 1H), 1.79 (dd, *J* = 13.0, 2.0 Hz, 2H), 1.76 – 1.64 (m, 2H), 1.34 – 1.15 (m, 4H), 1.08 – 0.97 (m, 2H); **^{13}C NMR** (126 MHz, $CDCl_3$) δ 146.4, 139.9, 139.7, 129.8, 129.2, 128.6, 127.0, 126.3, 58.2, 56.21, 56.16, 55.9, 53.5, 49.4, 42.7, 34.0, 33.5, 25.9, 24.6; **HRMS** (ESI/[M+H]$^+$) calcd. for $C_{25}H_{34}ClN_2O_2S^+$: 461.2024, found [M+H]$^+$: 461.2020; **FTIR** (neat) v_{max} 2933, 2850, 1582, 1306, 1143, 1087, 777, 699 cm^{-1}.

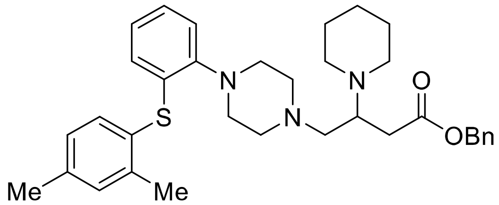

Benzyl 4-(4-(2-((2,4-dimethylphenyl)thio)phenyl)piperazin-1-yl)-3-(piperidin-1-yl)butanoate (45)

Following the general procedure A, using allenoate **2** (17.4 mg, 0.1 mmol, 1.0 equiv), piperidine (11.0 μL, 0.11 mmol, 1.1 equiv) and Vortioxetine (44.7 mg, 0.15 mmol, 1.5 equiv). The reaction time of the first amination step was 4 h. The product was isolated by silica gel chromatography as a colorless oil (30.7 mg, 55% yield). **^{1}H NMR** (500 MHz, $CDCl_3$) δ 7.41 – 7.35 (m, 5H), 7.34 – 7.29 (m, 1H), 7.16 – 7.14 (m, 1H), 7.09 – 6.99 (m, 3H), 6.89 – 6.80 (m, 1H), 6.50 (dd, *J* = 7.9, 1.4 Hz, 1H), 5.25 – 5.09 (m, 2H), 3.39 – 3.24 (m, 1H), 3.02 (bs, 4H), 2.77 (bs, 2H), 2.63 – 2.45 (m, 8H), 2.36 (s, 1H), 2.32 (s, 1H), 1.56 – 1.48 (m, 4H), 1.46 – 1.35 (m, 2H); **^{13}C NMR** (126 MHz, $CDCl_3$) δ 173.2, 149.5, 142.6, 139.3, 136.4, 136.3, 134.6, 131.8, 128.6, 128.4, 128.3, 128.2, 127.9, 126.3, 125.5, 124.3, 119.8, 66.2, 59.7, 59.6, 54.2, 51.9, 50.2, 35.6, 26.7, 24.9, 21.3, 20.7; **HRMS**

(ESI/[M+H]+) calcd. for $C_{34}H_{44}N_3O_2S^+$: 558.3149, found [M+H]+: 558.3122; **FTIR** (neat) v_{max} 2933, 2815, 1733, 1579, 1470, 1440, 1376, 1224, 1150, 1010, 750 cm−1.

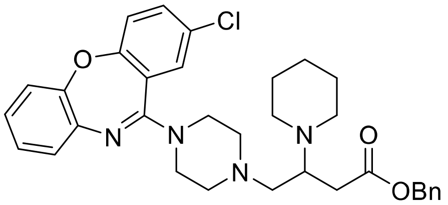

Benzyl 4-(4-(2-chlorodibenzo[*b,f*][1,4]oxazepin-11-yl)piperazin-1-yl)-3-(piperidin-1-yl)butanoate (46)

Following the general procedure B, using allenoate **2** (17.4 mg, 0.1 mmol, 1.0 equiv), *N*-chloropiperidine (13.1 mg, 0.11 mmol, 1.1 equiv) and Amoxapine (46.7 mg, 0.15 mmol, 1.5 equiv). The reaction time of the first amination step was 4 h. The product was isolated by silica gel chromatography as a colorless oil (37.8 mg, 66% yield). **^{1}H NMR** (500 MHz, $CDCl_3$) δ 7.40 – 7.29 (m, 6H), 7.28 (d, *J* = 2.6 Hz, 1H), 7.18 (d, *J* = 8.6 Hz, 1H), 7.13 (dd, *J* = 7.8, 1.7 Hz, 1H), 7.11 – 7.05 (m, 2H), 7.01 – 6.95 (m, 1H), 5.24 – 5.03 (m, 2H), 3.59 – 3.12 (m, 5H), 2.80 – 2.28 (m, 12H), 1.58 – 1.45 (m, 4H), 1.42 – 1.31 (m, 2H); **^{13}C NMR** (126 MHz, $CDCl_3$) δ 173.1, 159.4, 159.0, 151.9, 140.4, 136.3, 132.54, 130.3, 129.3, 128.7, 128.4, 128.3, 127.2, 125.9, 125.2, 124.5, 122.8, 120.2, 66.3, 59.8, 59.4, 53.6, 50.2, 35.4, 26.6, 24.9; **HRMS** (ESI/[M+H]+) calcd. for $C_{33}H_{38}ClN_4O_3^+$: 573.2626, found [M+H]+: 573.2623; **FTIR** (neat) v_{max} 2932, 2850, 2805, 1730, 1600, 1587, 1556, 1453, 1304, 1241, 1215, 1154, 1109, 1004, 752 cm−1.

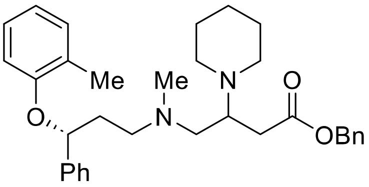

Benzyl 4-(methyl((*R*)-3-phenyl-3-(*o*-tolyloxy)propyl)amino)-3-(piperidin-1-yl)butanoate (47)

Following the general procedure B, using allenoate **2** (17.4 mg, 0.1 mmol, 1.0 equiv), *N*-chloropiperidine (13.1 mg, 0.11 mmol, 1.1 equiv) and Atomoxetine (38.3 mg, 0.15 mmol, 1.5 equiv). The reaction time of the first amination step was 4 h. The product was isolated by silica gel chromatography as a colorless oil (41.7 mg, 81% yield, 1:1 dr, characterized as an inseparable mixture). **^{1}H NMR** (500 MHz, $CDCl_3$) δ 7.42 – 7.30 (m, 8H), 7.30 – 7.23 (m, 2H), 7.12 (d, *J* = 7.2 Hz, 1H), 7.00 – 6.92 (m, 1H), 6.78 (t, *J* = 7.3 Hz, 1H), 6.62 (dd, *J* = 8.1, 2.7 Hz, 1H), 5.23 (dd, *J* = 8.3, 4.4 Hz, 1H), 5.15 – 5.08 (m, 2H), 3.24 (bs, 1H), 2.72 – 2.41 (m, 9H), 2.38 – 2.30 (m, 4H), 2.24 (s, 3H), 2.17 – 2.12 (m, 1H), 2.01 – 1.96 (m, 1H), 1.50 (bs, 4H), 1.40 – 1.32 (m, 2H); **^{13}C NMR** (126 MHz, $CDCl_3$) δ [173.1, 172.9], 156.2, [142.44, 142.38], 136.3, [130.67, 130.66], 128.7, 128.6, 128.4, 128.2, 127.5, 127.0, 126.7, [125.94, 125.90], 120.3, 112.9, [77.91, 77.85], 66.2, [60.3, 60.1], 58.6, 55.0, 50.1, 42.6, 36.6, 29.8, 26.5, 24.8, 16.7; **HRMS** (ESI/[M+H]+) calcd. for $C_{33}H_{43}N_2O_3^+$: 515.3268, found [M+H]+: 515.3268; **FTIR** (neat) v_{max} 2932, 2849, 2797, 1731, 1491, 1454, 1238, 1119, 752, 699 cm−1.

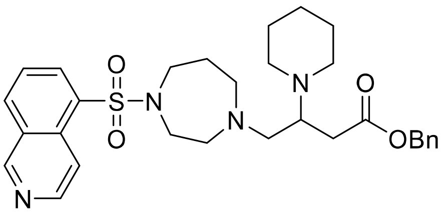

Benzyl 4-(4-(isoquinolin-5-ylsulfonyl)-1,4-diazepan-1-yl)-3-(piperidin-1-yl)butanoate (48)

Following the general procedure A, using allenoate **2** (17.4 mg, 0.1 mmol, 1.0 equiv), piperidine (11.0 μL, 0.11 mmol, 1.1 equiv) and Fasudil (43.6 mg, 0.15 mmol, 1.5 equiv). The reaction time of the first amination step was 4 h. The product was isolated by silica gel chromatography as a light-yellow oil (29.2 mg, 53% yield). **^{1}H NMR** (500 MHz, $CDCl_3$) δ 9.32 (d, *J* = 1.0 Hz, 1H), 8.67 (d, *J* = 6.2 Hz, 1H), 8.44 (d, *J* = 6.2 Hz, 1H), 8.31 (dd, *J* = 7.4, 1.2 Hz, 1H), 8.16 (d, *J* = 8.2 Hz, 1H), 7.73 – 7.63 (m, 1H), 7.37 – 7.25 (m, 5H), 5.07 – 5.00 (m, 2H), 3.47 – 3.27 (m, 5H), 3.16 – 3.06 (m, 1H), 2.78 – 2.56 (m, 5H), 2.53 – 2.26 (m, 7H), 1.84 – 1.66 (m, 2H), 1.51 – 1.41 (m, 4H), 1.36 (q, *J* = 5.4 Hz, 2H); **^{13}C NMR** (126 MHz, $CDCl_3$) δ 173.1, 153.3, 145.2, 136.2, 134.7, 133.4, 133.1, 131.8, 129.3, 128.6, 128.4, 128.2, 126.0, 117.8, 66.2, 60.5, 58.4, 56.6, 55.0, 50.2, 48.4, 46.2, 34.9, 28.4, 26.5, 24.8; **HRMS** (ESI/[M+H]$^+$) calcd. for $C_{30}H_{39}N_4O_4S^+$: 551.2687, found [M+H]$^+$: 551.2691; **FTIR** (neat) v_{max} 2932, 1728, 1615, 1453, 1327, 1155, 1135, 709 cm^{-1}.

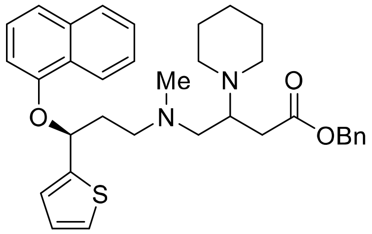

Benzyl 4-(methyl((*S*)-3-(naphthalen-1-yloxy)-3-(thiophen-2-yl)propyl)amino)-3-(piperidin-1-yl)butanoate (49)

Following the general procedure B, using allenoate **2** (17.4 mg, 0.1 mmol, 1.0 equiv), *N*-chloropiperidine (13.1 mg, 0.11 mmol, 1.1 equiv) and Duloxetine (44.6 mg, 0.15 mmol, 1.5 equiv). The reaction time of the first amination step was 4 h. The product was isolated by silica gel chromatography as a colorless oil (46.2 mg, 83% yield, 1:1 dr, characterized as an inseparable mixture). **^{1}H NMR** (500 MHz, $CDCl_3$) δ 8.41 – 8.31 (m, 1H), 7.82 – 7.73 (m, 1H), 7.51 – 7.45 (m, 2H), 7.41 – 7.24 (m, 7H), 7.20 (dt, *J* = 5.0, 1.2 Hz, 1H), 7.07 (t, *J* = 3.5 Hz, 1H), 6.96 – 6.91 (m, 1H), 6.88 (dd, *J* = 7.5, 5.2 Hz, 1H), 5.76 (t, *J* = 6.5 Hz, 1H), 5.21 – 5.01 (m, 2H), 3.28 – 3.16 (m, 1H), 2.72 – 2.33 (m, 11H), 2.29 – 2.23 (m, 1H), 2.21 (s, 3H), 2.19 – 2.10 (m, 1H), 1.45 (d, *J* = 4.6 Hz, 4H), 1.39 – 1.30 (m, 2H); **^{13}C NMR** (126 MHz, $CDCl_3$) δ [173.1, 173.0], [153.7, 153.6], 145.6, 136.3, 134.7, 128.6, 128.4, 128.2, 127.6, 126.7, 126.4, 126.3, 125.9, 125.3, 124.8, 122.3, 120.6, 107.2, 107.1, [74.64, 74.57], 66.2, [60.3, 60.1], [58.9, 58.8], 54.8, [50.09, 50.06], 42.6, [36.9, 36.8], [35.2, 34.9], 26.5, 24.8; **HRMS** (ESI/[M+H]$^+$) calcd. for $C_{34}H_{41}N_2O_3S^+$: 557.2832, found [M+H]$^+$: 557.2832; **FTIR** (neat) v_{max} 2931, 2849, 2796, 1731, 1595, 1507, 1456, 1396, 1263, 1235, 1095, 757 cm^{-1}.

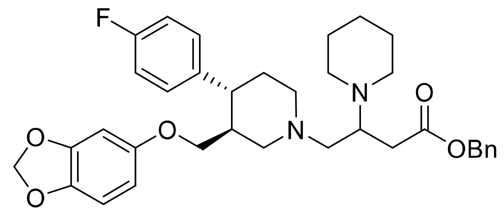

Benzyl 4-((3*S*,4*R*)-3-((benzo[d][1,3]dioxol-5-yloxy)methyl)-4-(4-fluorophenyl)piperidin-1-yl)-3-(piperidin-1-yl)butanoate (50)

Following the general procedure B, using allenoate **2** (17.4 mg, 0.1 mmol, 1.0 equiv), *N*-chloropiperidine (13.1 mg, 0.11 mmol, 1.1 equiv) and Duloxetine (44.6 mg, 0.15 mmol, 1.5 equiv). The reaction time of the first amination step was 4 h. The product was isolated by silica gel chromatography as a colorless oil (37.1 mg, 63% yield, 1:1 dr, characterized as an inseparable mixture). ^{1}**H NMR** (500 MHz, $CDCl_3$) δ 7.44 – 7.29 (m, 5H), 7.14 – 7.06 (m, 2H), 6.94 (td, *J* = 8.6, 1.0 Hz, 2H), 6.62 (d, *J* = 8.5 Hz, 1H), 6.33 (t, *J* = 2.2 Hz, 1H), 6.12 (dt, *J* = 8.5, 2.2 Hz, 1H), 5.87 (s, 2H), 5.16 (d, *J* = 1.9 Hz, 2H), 3.55 – 3.50 (m, 1H), 3.45 – 2.87 (m, 4H), 2.64 – 2.38 (m, 9H), 2.28 – 1.97 (m, 3H), 1.76 (s, 2H), 1.53 (s, 4H), 1.41 (d, *J* = 4.5 Hz, 2H); ^{13}**C NMR** (126 MHz, $CDCl_3$) δ [173.14, 173.07], 161.6 (d, *J* = 244.4 Hz), [154.6, 154.5], 148.3, 141.7, 139.9, 136.3, 128.91 (d, *J* = 10.6 Hz), 128.90, 128.7, 128.2, [115.48 (d, *J* = 20.9 Hz), 115.45 (d, *J* = 20.9 Hz)], 108.0, [105.7, 105.6], 101.2, [98.12, 98.06], 69.7, 66.2, [59.8, 59.7], 57.2, 55.8, 53.6, 50.2, [44.2, 44.0], [42.3, 42.1], 34.5, 29.8, 26.5, 24.9; ^{19}**F NMR** (471 MHz, $CDCl_3$) δ −117.80; **HRMS** (ESI/[M+H]$^+$) calcd. for $C_{35}H_{42}FN_2O_5^+$: 589.3072, found [M+H]$^+$: 589.3073; **FTIR** (neat) ν_{max} 2931, 2850, 2805, 1731, 1508, 1488, 1467, 1221, 1184, 1135, 1037, 830 cm^{-1}.

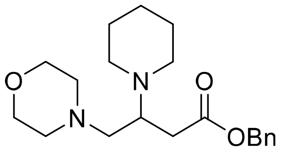

Benzyl 4-morpholino-3-(piperidin-1-yl)butanoate (51)

Following the general procedure A, using allenoate **2** (17.4 mg, 0.1 mmol, 1.0 equiv), piperidine (11.0 µL, 0.11 mmol, 1.1 equiv) and morpholine (14.0 µL, 0.15 mmol, 1.5 equiv). The reaction time of the first amination step was 4 h. The product was isolated by silica gel chromatography as a colorless oil (23.6 mg, 68% yield). ^{1}**H NMR** (500 MHz, $CDCl_3$) δ 7.41 – 7.27 (m, 5H), 5.15 – 5.09 (m, 2H), 3.66 – 3.53 (m, 4H), 3.29 – 3.21 (m, 1H), 2.59 – 2.40 (m, 9H), 2.36 – 2.25 (m, 3H), 1.54 – 1.43 (m, 4H), 1.41 – 1.36 (m, 2H); ^{13}**C NMR** (126 MHz, $CDCl_3$) δ 173.1, 136.3, 128.6, 128.3, 128.2, 67.2, 66.2, 60.4, 59.1, 54.2, 50.1, 35.3, 26.6, 24.9; **HRMS** (ESI/[M+H]$^+$) calcd. for $C_{20}H_{31}N_2O_3^+$: 347.2329, found [M+H]$^+$: 347.2325; **FTIR** (neat) ν_{max} 2932, 2852, 2804, 1732, 1454, 1289, 1137, 1117, 1009, 863, 743 cm^{-1}.

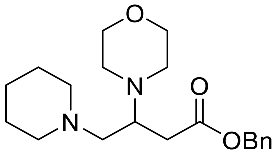

Benzyl 3-morpholino-4-(piperidin-1-yl)butanoate (52)

Following the general procedure A, using allenoate **2** (17.4 mg, 0.1 mmol, 1.0 equiv), morpholine (10.0 µL, 0.11 mmol, 1.1 equiv) and piperidine (15.0 µL, 0.15 mmol, 1.5 equiv). The reaction time of the first amination step was 7 h. The product was isolated by silica gel chromatography as a colorless oil (25.0 mg, 72% yield). ^{1}**H NMR** (500 MHz, $CDCl_3$) δ 7.41 – 7.27 (m, 5H), 5.15 – 5.09 (m, 2H), 3.66 – 3.53 (m, 4H), 3.29 – 3.21 (m, 1H), 2.59 – 2.40 (m, 9H), 2.36 – 2.25 (m, 3H), 1.54 – 1.43 (m, 4H), 1.41 – 1.36 (m, 2H); ^{13}**C NMR** (126 MHz, $CDCl_3$) δ 173.1, 136.3, 128.6, 128.3, 128.2, 67.2, 66.2, 60.4, 59.1, 54.2, 50.1, 35.3, 26.6, 24.9; **HRMS** (ESI/[M+H]$^+$) calcd. for $C_{20}H_{31}N_2O_3^+$: 347.2329, found [M+H]$^+$: 347.2320; **FTIR** (neat) v_{max} 2931, 2818, 1732, 1600, 1452, 1280, 1150, 1110, 757, 698 cm^{-1}.

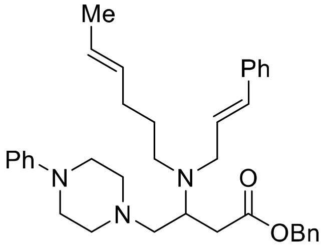

Benzyl 3-(cinnamyl((*E*)-hex-4-en-1-yl)amino)-4-(4-phenylpiperazin-1-yl)butanoate (67)

Following the general procedure A, using allenoate **2** (17.4 mg, 0.1 mmol, 1.0 equiv), amine **57** (23.7 mg, 0.11 mmol, 1.1 equiv) and 1-phenylpiperazine (24.3 mg, 0.15 mmol, 1.5 equiv). The reaction time of the first amination step was 8 h. The product was isolated by silica gel chromatography as a colorless oil (33.1 mg, 60% yield). ^{1}**H NMR** (500 MHz, $CDCl_3$) *δ* 7.44 – 7.11 (m, 13H), 6.91 (d, *J* = 7.7 Hz, 2H), 6.86 (t, *J* = 7.3 Hz, 1H), 6.51 (d, *J* = 15.9 Hz, 1H), 6.18 (dt, *J* = 15.9, 6.4 Hz, 1H), 5.47 – 5.36 (m, 2H), 5.22 – 5.02 (m, 2H), 3.56 (dtd, *J* = 9.4, 7.2, 5.1 Hz, 1H), 3.29 (d, *J* = 6.0 Hz, 2H), 3.11 (dt, *J* = 6.5, 3.4 Hz, 4H), 2.69 (td, *J* = 6.4, 5.7, 3.3 Hz, 2H), 2.59 – 2.45 (m, 7H), 2.36 (dd, *J* = 12.4, 9.4 Hz, 1H), 2.03 – 1.91 (m, 2H), 1.67 – 1.57 (m, 3H), 1.56 – 1.47 (m, 2H); ^{13}**C NMR** (125 MHz, $CDCl_3$) *δ* 172.78, 151.4, 137.3, 136.1, 131.3, 131.2, 129.5, 129.1, 128.6, 128.5, 128.3, 128.1, 127.2, 126.3, 125.0, 119.6, 116.0, 66.2, 59.8, 54.7, 53.8, 53.0, 49.6, 49.2, 36.5, 30.2, 28.7, 18.0; **HRMS** (ESI/[M+H$^+$]) *m/z* calcd. for $C_{36}H_{46}N_3O_2^+$: 552.3585, found [M+H]$^+$: 552.3604; **FTIR** (neat) v_{max} 2936, 2818, 1732, 1600, 1496, 1453, 1379, 1295, 1232, 1137, 1009, 967, 757, 692 cm^{-1}.

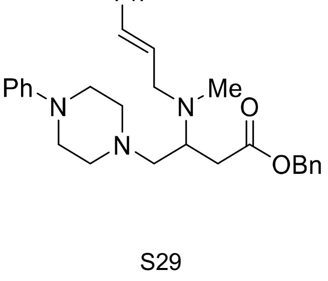

Benzyl 3-(cinnamyl(methyl)amino)-4-(4-phenylpiperazin-1-yl)butanoate (68)

Following the general procedure A, using allenoate **2** (17.4 mg, 0.1 mmol, 1.0 equiv), amine **63** (16.2 mg, 0.11 mmol, 1.1 equiv) and 1-phenylpiperazine (24.3 mg, 0.15 mmol, 1.5 equiv). The reaction time of the first amination step was 6 h. The product was isolated by silica gel chromatography as a yellowish oil (25.6 mg, 53% yield). **^{1}H NMR** (500 MHz, $CDCl_3$) δ 7.41 – 7.19 (m, 13H), 6.91 (d, J = 7.7 Hz, 2H), 6.85 (t, J = 7.3 Hz, 1H), 6.51 (d, J = 15.8 Hz, 1H), 6.19 (dt, J = 15.9, 6.5 Hz, 1H), 5.19 – 5.06 (m, 2H), 3.56 – 3.46 (m, 1H), 3.30 (dd, J = 6.6, 1.4 Hz, 2H), 3.19 – 3.06 (m, 4H), 2.69 (dt, J = 10.4, 5.5 Hz, 2H), 2.63 – 2.56 (m, 2H), 2.56 – 2.48 (m, 3H), 2.38 (dd, J = 12.4, 9.0 Hz, 1H), 2.29 (s, 3H); **^{13}C NMR** (125 MHz, $CDCl_3$) δ 172.7, 151.3, 137.1, 136.1, 132.0, 129.1, 128.6, 128.5, 128.3, 128.23, 128.16, 127.4, 126.3, 119.6, 116.0, 66.3, 59.6, 56.9, 56.9, 53.7, 49.2, 36.9, 35.4; **HRMS** (ESI/[M+H]$^+$) *m/z* calcd. for $C_{31}H_{38}N_3O_2^+$: 484.2959, found [M+H]$^+$: 484.2976; **FTIR** (neat) ν_{max} 2942, 2816, 1732, 1600, 1497, 1449, 1383, 1295, 1233, 1138, 1010, 754, 693 cm^{-1}.

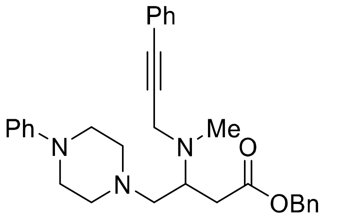

Benzyl 3-(methyl(3-phenylprop-2-yn-1-yl)amino)-4-(4-phenylpiperazin-1-yl)butanoate (70)

Following the general procedure A, using allenoate **2** (17.4 mg, 0.1 mmol, 1.0 equiv), amine **69** (16.0 mg, 0.11 mmol, 1.1 equiv) and 1-phenylpiperazine (24.3 mg, 0.15 mmol, 1.5 equiv). The reaction time of the first amination step was 6 h. The product was isolated by silica gel chromatography as a yellowish oil (23.1 mg, 48% yield). **^{1}H NMR** (500 MHz, $CDCl_3$) δ 7.46 – 7.38 (m, 2H), 7.38 – 7.22 (m, 10H), 6.90 (d, J = 8.5 Hz, 2H), 6.85 (t, J = 7.3 Hz, 1H), 5.18 – 5.05 (m, 2H), 3.66 (d, J = 8.3 Hz, 2H), 3.62 – 3.57 (m, 1H), 3.18 – 3.06 (m, 4H), 2.77 – 2.65 (m, 4H), 2.61 – 2.51 (m, 3H), 2.43 (s, 3H), 2.42 – 2.35 (m, 1H); **^{13}C NMR** (125 MHz, $CDCl_3$) δ 172.5, 151.3, 136.1, 131.6, 129.1, 128.5, 128.3, 128.22, 128.15, 128.1, 123.2, 119.6, 116.0, 86.2, 84.8, 66.3, 60.1, 56.7, 53.7, 49.1, 44.6, 37.0, 35.8; **HRMS** (ESI/[M+H]$^+$) *m/z* calcd. for $C_{31}H_{36}N_3O_2^+$: 482.2802, found [M+H]$^+$: 482.2818; **FTIR** (neat) ν_{max} 2943, 2818, 1731, 1599, 1490, 1454, 1383, 1233, 1139, 1009, 757, 692 cm^{-1}.

III. Supplementary Discussion

5. X-Ray Crystallography Data for Compounds 4 and 36

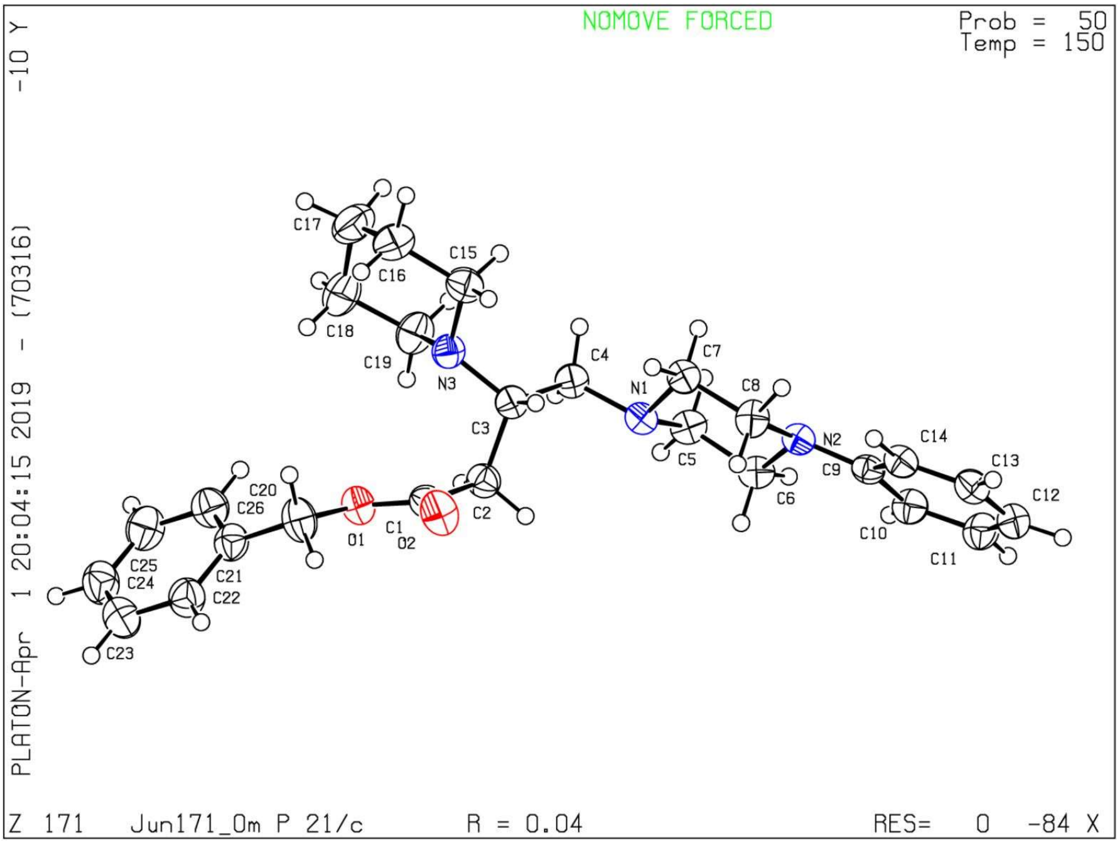

Supplementary Fig. 1. X-ray structure of **4**.

A colorless plate shaped crystal of **4** for X-ray diffraction was obtained by slow evaporation of a methanol solution of **4**. The data were collected at 150(2) K on a Bruker AXS D8 Quest CMOS diffractometer with Mo sealed tube and curved triumph monochromator with a 10 cm × 10 cm Photon-100 detector and fixed chi angle. The supplementary crystallographic data was deposited in The Cambridge Crystallographic Data Centre. CCDC 2004609.

Supplementary Table 1. X-ray analysis data of **4**.

Bond precision:		C−C = 0.0021 Å	Wavelength=1.54178
Cell:	a=24.8055(8)	b=5.7378(2)	c=16.5566(7)
	alpha=90	beta=98.9700(17)	gamma=90
Temperature:150 K			

	Calculated	Reported
Volume	2327.67(15)	2327.66(15)
Space group	P 2l/c	P 2l/c
Hall group	−P 2ybc	−P 2ybc
Moiety formula	$C_{26}H_{35}N_3O_2$	?
Sum formula	$C_{26}H_{35}N_3O_2$	$C_{26}H_{35}N_3O_2$
Mr	421.57	421.57
Dx, g cm^{-3}	1.203	1.203
Z	4	4
Mu (mm^{-1})	0.599	0.599
F000	912.0	912.0
F000’	914.49	
h, k, l max	31, 7, 21	31, 7, 21
Nref	5161	4933
Tmin, Tmax	0.891, 0.982	0.562, 0.754
Tmin’	0.841	

Correction method= # Reported T Limits: Tmin=0.562
Tmax=0.754 AbsCorr = MULTI-SCAN

Data completeness= 0.956 Theta(max)= 81.697

R(reflections)= 0.0429(4356) wR2(reflections)= 0.1218(4933)

S = 1.077 Npar= 282

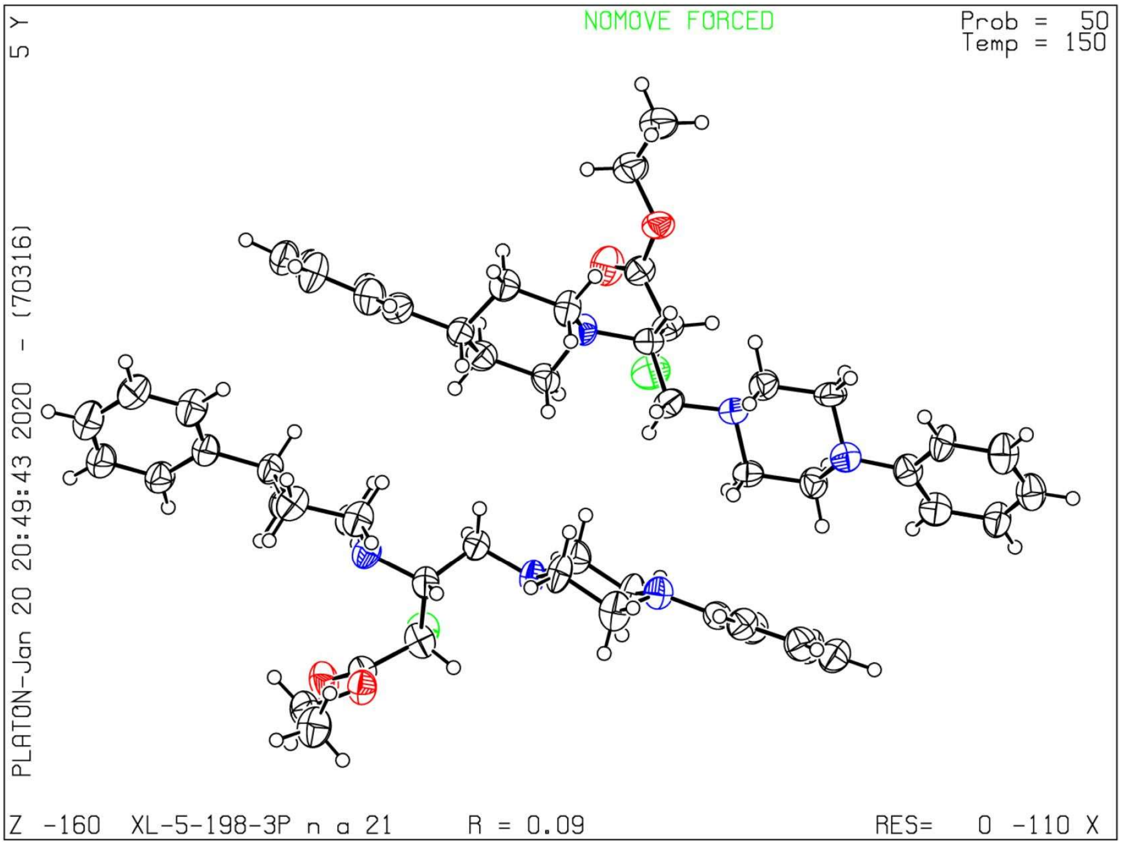

Supplementary Fig. 2. X-ray Structure of **36**.

A colorless plate shaped crystal of **36** for X-ray diffraction was obtained by slow evaporation of a methanol solution of **36**. The data were collected at 150(2) K on a Bruker AXS D8 Quest CMOS diffractometer with Mo sealed tube and curved triumph monochromator with a 10 cm × 10 cm Photon-100 detector and fixed chi angle. The supplementary crystallographic data was deposited in The Cambridge Crystallographic Data Centre. CCDC 2004611.

Supplementary Table 2. X-ray analysis data of **36**.

Bond precision:	C−C = 0.0139 Å		Wavelength=1.54178
Cell:	a=48.437(5)	b=6.0513(5)	c=16.8568(9)
	alpha=90	beta=90	gamma=90
Temperature:150 K			
	Calculated		Reported
Volume	4940.8(7)		4940.9(7)
Space group	P n a 2l		P n a 2l
Hall group	P 2c -2n		P 2c -2n
Moiety formula	$C_{27}H_{36}FN_3O_2$		?
Sum formula	$C_{27}H_{36}FN_3O_2$		$C_{27}H_{36}FN_3O_2$
Mr	453.59		453.59
Dx, g cm^{-3}	1.220		1.220
Z	8		8
Mu (mm^{-1})	0.660		0.660
F000	1952.0		1952.0
F000'	1957.70		
h, k, l max	59, 7, 20		58, 7, 20
Nref	9459 [4902]		8976
Tmin, Tmax	0.977, 0.987		0.532, 0.753
Tmin'	0.841		

Correction method= # Reported T Limits: Tmin=0.532
Tmax=0.753 AbsCorr = MULTI-SCAN
Data completeness= 1.83/0.95 Theta(max)= 70.335
R(reflections)= 0.0871(4026) wR2(reflections)= 0.2423(8976)
S = 0.914 Npar= 598

Alert level B
PLAT340_ALERT_3_B Low Bond Precision on C-C Bonds .... 0.01391 Ang.
Author Response: Data were collected on a very small needle shaped crystal with dimensions 0.15 x 0.03 x 0.02 mm.

6. Mechanistic Study Experiments

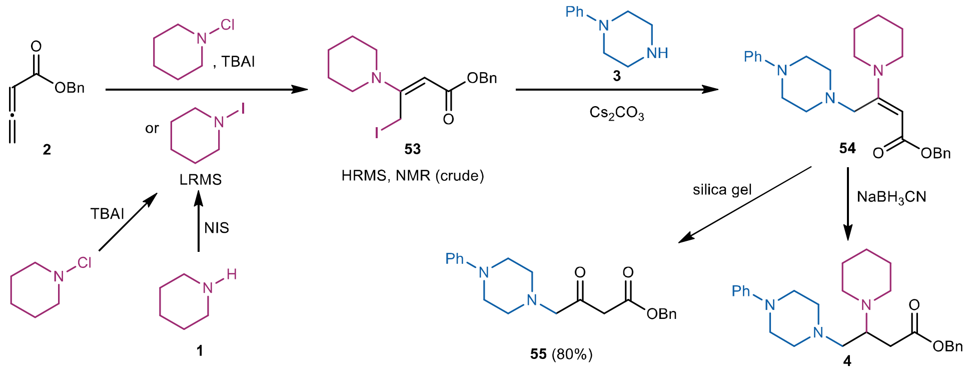

a. LRMS analyses for the formation of *N*-iodopiperidine from *N*-chloropiperidine and TBAI

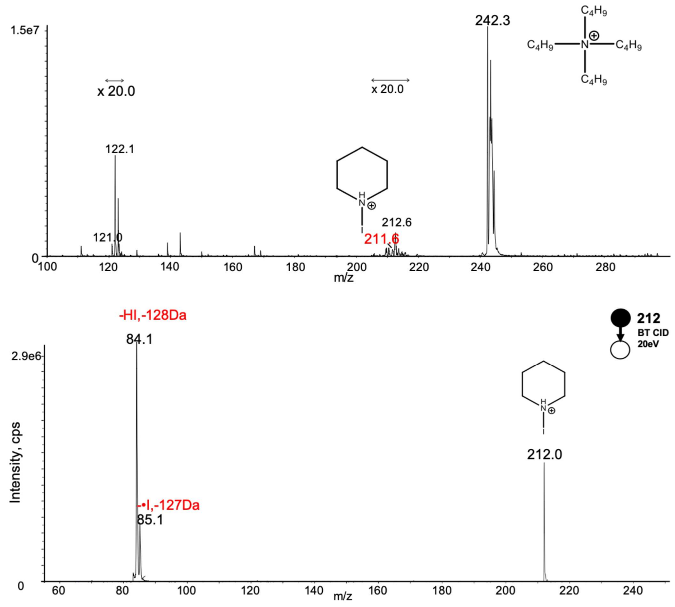

Supplementary Fig. 3. LRMS spectra of *N*-iodopiperidine.

b. HRMS analyses for the intermediates 53 and 54

Intermediate 53: To an oven-dried 10 mL vial wrapped with aluminum foil and equipped with a stir bar was added allenoate **2** (17.4 mg, 0.1 mmol, 1.0 equiv), *N*-chloropiperidine (13.1 mg, 0.11 mmol, 1.1 equiv), tetrabutylammonium iodide (TBAI, 55.4 mg, 0.15 mmol, 1.5 equiv) and CH_3CN (2.0 mL) under argon atmosphere. The reaction mixture was stirred for 4 h at room temperature before passing through a celite plug to remove solid. The solvent was removed under vacuum and the crude product was dissolved in 0.5 mL CD_3CN for NMR studies. The identity of allylic iodide **53** was confirmed by NMR (crude) and HRMS analyses of the reaction mixture. *(Note: The same allylic*

*iodide was obtained when **2** was treated with N-iodopiperidine generated in situ from piperidine **1** and NIS).*

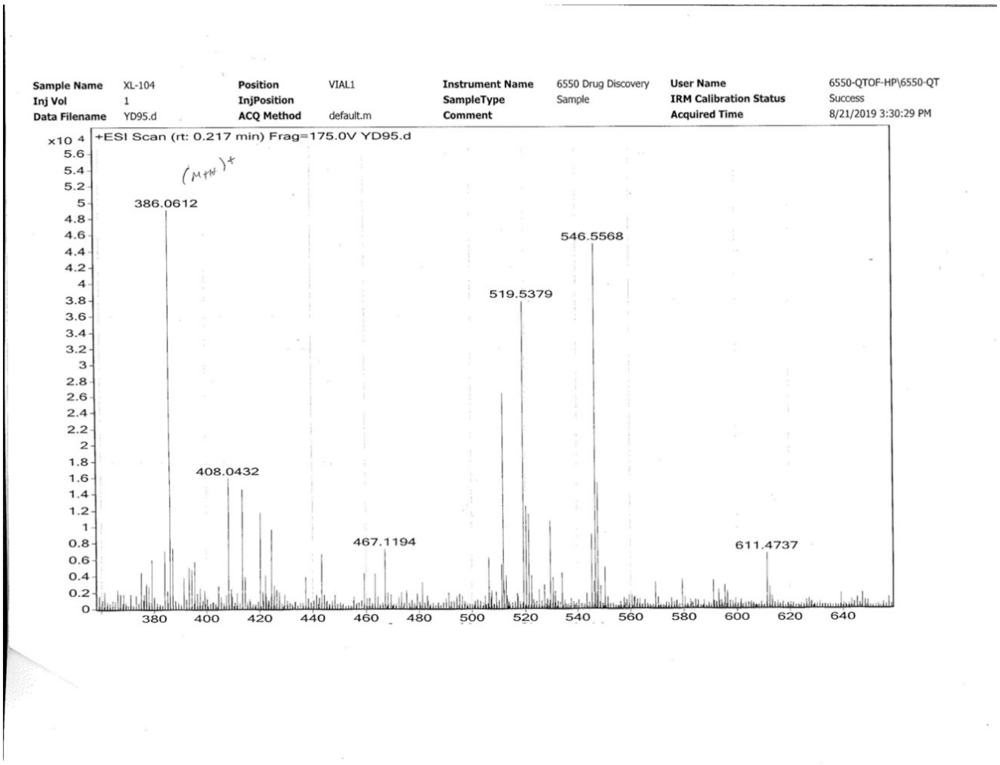

Supplementary Fig. 4. HRMS spectra of crude reaction mixture containing **53**. HRMS spectrum of **53** (ESI/[M+H]$^+$) calcd. for $C_{16}H_{21}INO_2^+$: 386.0611, found [M+H]$^+$: 386.0612.

Intermediate 54: To an oven-dried 10 mL vial wrapped with aluminum foil and equipped with a stir bar was added allenoate **2** (17.4 mg, 0.1 mmol, 1.0 equiv), *N*-chloropiperidine (13.1 mg, 0.11 mmol, 1.1 equiv), tetrabutylammonium iodide (TBAI, 55.4 mg, 0.15 mmol, 1.5 equiv) and CH_3CN (2.0 mL) under argon atmosphere. The reaction mixture was stirred for 4 h at room temperature. Cs_2CO_3 (48.9 mg, 0.15 mmol, 1.5 equiv) and 1-phenylpiperazine **3** (24.3 mg, 0.15 mmol, 1.5 equiv) was added. The reaction mixture was then stirred for 24 h at room temperature. The reaction mixture was passed through a celite plug to remove the solid. The solvent was removed under vacuum to give a crude product. The crude product was passed through a column of neutral Al_2O_3 to provide crude intermediate **54**. The identity of **54** was confirmed by NMR (crude) and HRMS analyses. (*Note: Because the intermediate **54** can be hydrolyzed to generate **55** by silica gel column purification, we used Al_2O_3 column chromatography to isolate **54**. However, the intermediate **54** was very unstable during the NMR analysis process*).

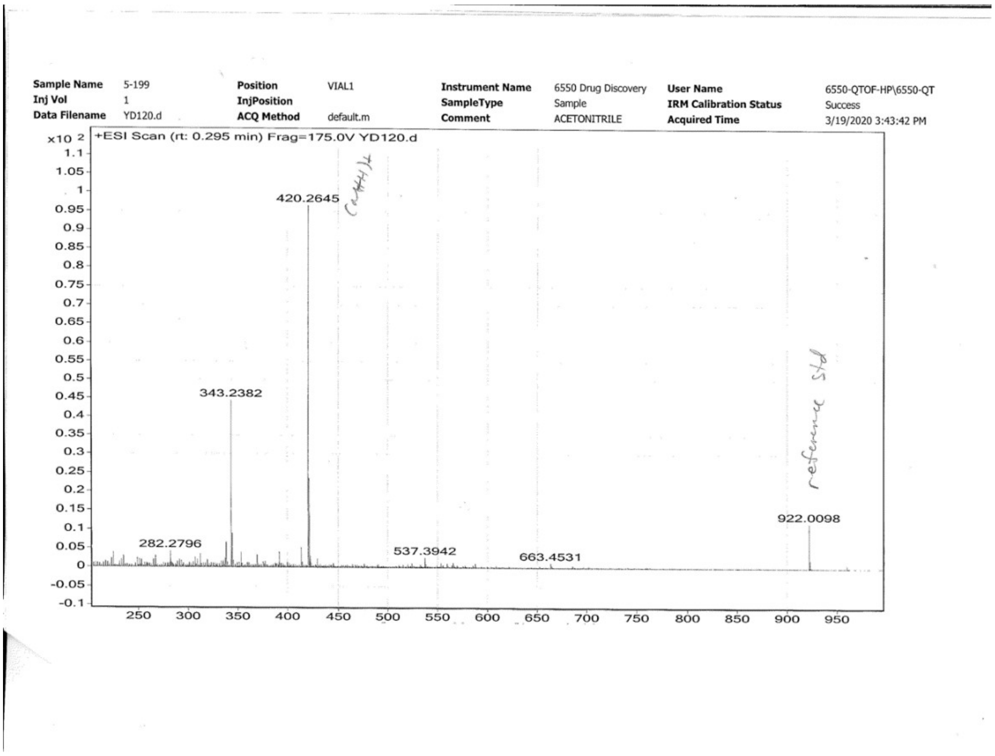

Supplementary Fig. 5. HRMS spectra of crude **54**. HRMS spectrum of **54** (ESI/$[M+H]^+$) calcd. for $C_{26}H_{34}N_3O_2^+$: 420.2646, found $[M+H]^+$: 420.2645.

c. Radical trapping experiments

When BHT was added to the reaction mixture of *N*-chloropiperidine and TBAI, compound **56** was formed in 43% yield (eq. 1). Its formation was not observed without TBAI (eq. 2). Both TEMPO and BHT inhibited the formation of **53** in presence of allenoate **2** (eqs. 3 and 4). The formation of **56** was again observed when BHT was used in the presence allenoate **2** (eq. 3). These results indicate the amino radical process, but still cannot rule out the ionic processes. Alkyl amine radical addition to allene is very rare in literature[5]. Notably, one example was reported by Neale in 1967. However, in Neale's case, the alkyl amine radical added to the allene terminal carbon, not the sp carbon[6].

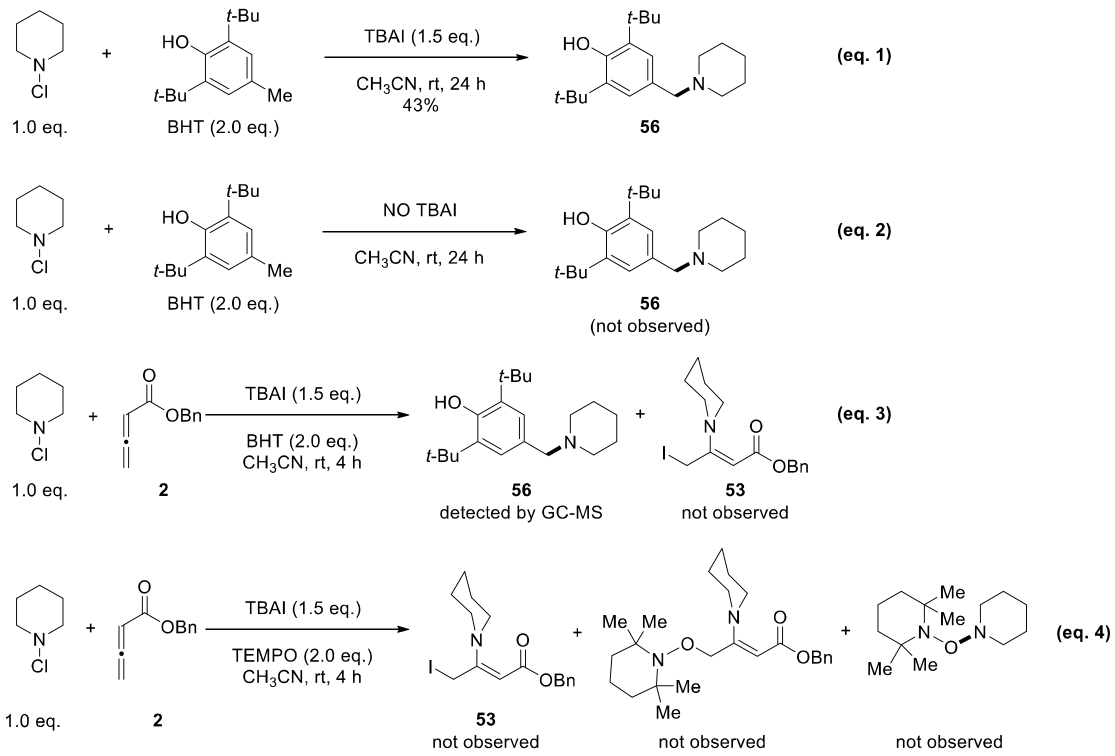

Supplementary Fig. 6. Radical trapping experiments.

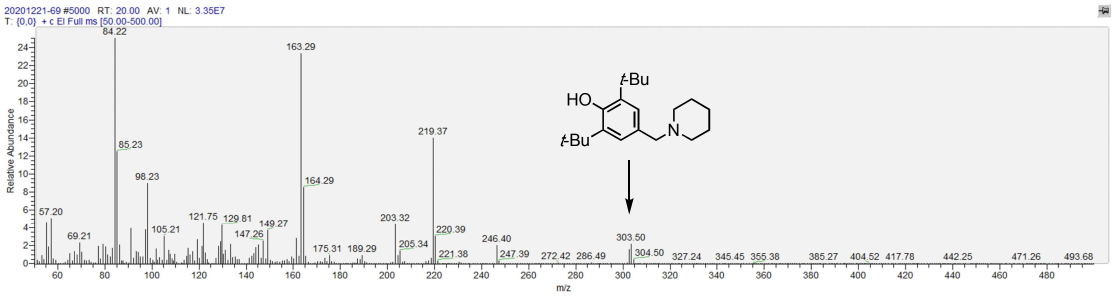

Supplementary Fig. 7. GC-MS analysis of **56**.

Characterization for compound 55

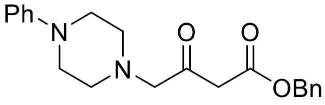

Benzyl 3-oxo-4-(4-phenylpiperazin-1-yl)butanoate (55)

^{1}H NMR (500 MHz, $CDCl_3$) δ 7.42 – 7.29 (m, 5H), 7.31 – 7.22 (m, 2H), 7.06 – 6.81 (m, 3H), 5.18 (s, 2H), 3.58 (s, 2H), 3.30 (s, 2H), 3.23 – 3.10 (m, 4H), 2.67 – 2.56 (m, 4H); **^{13}C NMR** (126 MHz, $CDCl_3$) δ 201.9, 167.3, 151.2, 135.4, 129.3, 128. 8, 128.7, 128.6, 120.1, 116.3, 67.6, 67.3, 53.5, 49.2, 47.0; **HRMS** (ESI/[M+H]$^+$) calcd. for $C_{21}H_{25}N_2O_3^+$: 353.1860, found [M+H]$^+$: 353.1861; **FTIR** (neat) ν_{max} 2931, 2823, 1744, 1720, 1599, 1497, 1453, 1383, 1307, 1231, 753, 696 cm^{-1}.

Characterization for compound 56

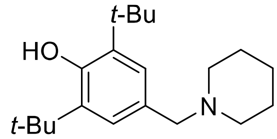

2,6-di-*tert*-butyl-4-(piperidin-1-ylmethyl)phenol (56)

^{1}H NMR (400 MHz, $CDCl_3$) δ 7.09 (s, 2H), 5.11 (s, 1H), 3.42 (s, 2H), 2.39 (s, 4H), 1.62 – 1.55 (m, 4H), 1.45 (s, 20H); **^{13}C NMR** (101 MHz, $CDCl_3$) δ 152.8, 135.5, 128.9, 126.1, 64.1, 54.5, 34.4, 30.5, 26.1, 24.6; **GC-MS** *m/z* 303. This is a known compound and spectral data matched those previously reported[7].

Synthesis and characterization for compound 57

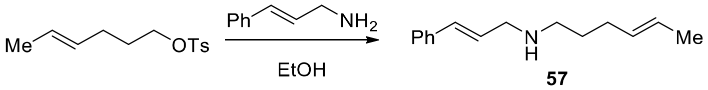

(*E*)-*N*-Cinnamylhex-4-en-1-amine (57)

The tosylate (200 mg, 0.79 mmol) was added to a solution of (*E*)-3-phenylprop-2-en-1-amine (314 mg, 2.36 mmol) in EtOH (3 mL) at 0 °C and the mixture was heated to 50 °C overnight. 2 N Aqueous sodium hydroxide was then added until the pH was greater than 10. The aqueous phase was extracted three times with EA and dried with Na_2SO_4. After removal of the solvent, the amine **57** was purified by silica gel chromatography (0.3% Et_3N and 5% MeOH in Ethyl acetate) as a yellowish oil (95 mg, 56%). **^{1}H NMR** (500 MHz, $CDCl_3$) δ 7.37 (d, *J* = 6.9 Hz, 2H), 7.30 (t, *J* = 7.6 Hz, 2H), 7.22 (t, *J* = 7.3 Hz, 1H), 6.52 (d, *J* = 15.9 Hz, 1H), 6.31 (dt, *J* = 15.8, 6.3 Hz, 1H), 5.53 – 5.36 (m, 2H), 3.41 (dd, *J* = 6.4, 1.5 Hz, 2H), 2.74 – 2.61 (m, 2H), 2.08 – 1.99 (m, 2H), 1.65 (dt, *J* = 5.1, 1.3 Hz, 3H), 1.62 – 1.53 (m, 2H); **^{13}C NMR** (125 MHz, $CDCl_3$) δ 137.2, 131.1, 130.9, 128.7, 128.5, 127.3, 126.3, 125.2, 52.0, 49.0, 30.4, 30.0, 17.9; **HRMS (**ESI/[M+H]$^+$) *m/z* calcd. for $C_{15}H_{22}N^+$: 216.1747, found [M+H]$^+$: 216.1751; **FTIR** (neat) ν_{max} 3024, 2929, 2854, 1448, 1122, 964, 742, 692 cm^{-1}.

Synthesis and characterization for compound 58

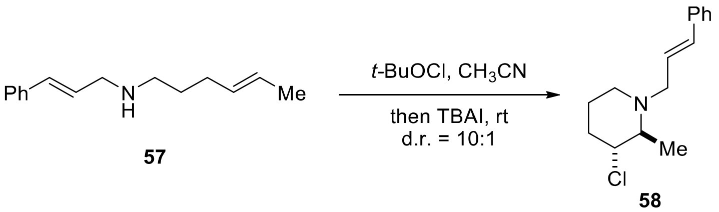

3-Chloro-1-cinnamyl-2-methylpiperidine (58)

To an oven-dried 10 mL vial wrapped with aluminum foil and equipped with a stir bar was added amine **57** (23.7 mg, 0.11 mmol, 1.0 equiv), *t*-BuOCl (14.0 μL, 0.12 mmol, 1.1 equiv), and CH_3CN (2.0 mL) under argon atmosphere. The reaction mixture was stirred for 1 h at room temperature. Then, TBAI (55.4 mg, 0.15 mmol, 1.4 equiv) was added and the reaction mixture was stirred for overnight. The solvent was evaporated under reduced pressure and the crude was isolated by silica gel chromatography (0.3% Et_3N and 10% Ethyl acetate in Hexane to 0.3% Et_3N and 5%MeOH in Ethyl acetate) to give the product as a yellow oil (7.7 mg, 28% yield, 10:1 d.r.) and recovered amine **57** (8.3 mg, 35%). **^{1}H NMR** (500 MHz, $CDCl_3$) (a pair of inseparable diastereomers were included, d.r. = 10:1) δ 7.41 – 7.36 (m, 2H), 7.35 – 7.28 (m, 2H), 7.26 – 7.20 (m, 1H), 6.54 (d, *J* = 15.8 Hz, 1H), 6.29 (ddd, *J* = 15.9, 7.7, 6.0 Hz, 1H), 3.74 (br s, 1H), 3.56 (dd, *J* = 14.8, 5.3 Hz, 1H), 3.32 (dd, *J* = 14.4, 7.7 Hz, 1H), 2.92 (d, *J* = 10.5 Hz, 1H), 2.58 – 2.50 (m, 1H), 2.43 – 2.31 (m, 1H), 2.31 – 2.20 (m, 1H), 1.79 – 1.72 (m, 1H), 1.72 – 1.58 (m, 2H), 1.38 (d, *J* = 6.2 Hz, 3H); **^{13}C NMR** (125 MHz, $CDCl_3$) (three carbon missing) δ 136.8, 128.6, 127.6, 126.3, 62.6, 56.1, 51.6, 35.0, 24.6, 16.3; **HRMS** (ESI/[M+H]$^+$) *m/z* calcd. for $C_{15}H_{21}NCl^+$: 250.1358 and 252.1328, found [M+H]$^+$: 250.1364 and 252.1334; **FTIR** (neat) ν_{max} 3026, 2941, 2854, 2797, 1449, 1214, 1114, 968, 739, 692 cm^{-1}.

Synthesis and characterization for compound 60

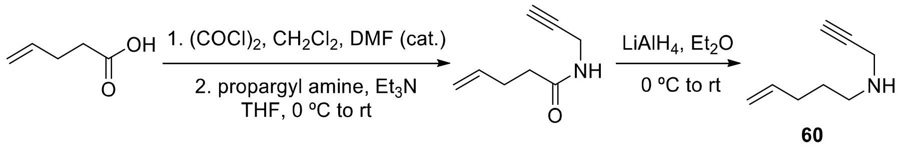

Step-I:

***N*-(prop-2-yn-1-yl)pent-4-enamide:** An oven-dried 250 mL round-bottom flask was charged with 5-pentenoic acid (4.0 g, 39.95 mmol) and CH_2Cl_2 (140 mL) and catalytic amount of DMF (2.9 μL, 0.04 mmol). The reaction mixture was cooled to 0 °C. Then, $(COCl)_2$ (6.76 mL, 79.9 mmol) was added dropwise to the reaction mixture. The reaction mixture was then warmed up to room temperature, and was stirred for 1 h. upon completion, the solvent was removed under reduced pressure to afford the acid chloride quantitatively which was used directly without further purification for the next step. To a cooled (0 °C) solution of the crude acid chloride in CH_2Cl_2 (130 mL) was added propargyl amine (3.3 mL, 51.94 mmol) drop wise followed by Et_3N (7.2 mL, 51.94 mmol).

The reaction was then warmed to room temperature, and was stirred for 6 h. The reaction was quenched with water (50 mL) and the aqueous layer was extracted with EtOAc (3 × 50 mL). The combined organic layer was dried with anhydrous Na_2SO_4. After removal of the solvent, the *N*-(prop-2-yn-1-yl)pent-4-enamide was purified by column chromatography (30% Ethyl acetate in Hexane) as a yellow solid (4.7 g, 86%). **^{1}H NMR** (400 MHz, $CDCl_3$) δ 6.23 (brs, 1H), 5.89 – 5.72 (m, 1H), 5.12 – 5.02 (m, 1H), 5.02 – 4.94 (m, 1H), 4.03 (ddt, *J* = 7.9, 5.2, 2.5 Hz, 2H), 2.45 – 2.33 (m, 2H), 2.32 – 2.25 (m, 2H), 2.22 (dq, *J* = 6.8, 2.5 Hz, 1H); **^{13}C NMR** (101 MHz, $CDCl_3$) δ 172.3, 136.9, 115.8, 79.7, 71.6, 35.5, 29.5, 29.2. Spectral data are in accordance with the reported data[8].

Step-II:

***N*-(prop-2-yn-1-yl)pent-4-en-1-amine (60)**

To a cooled (0 ºC) suspension of $LiAlH_4$ (2.0 g, 52.7 mmol) in anhydrous Et_2O (50 mL) was added the *N*-(prop-2-yn-1-yl)pent-4-enamide (1.8 g, 13.13 mmol) in anhydrous Et_2O (70 mL) under nitrogen atmosphere. The reaction was allowed to warm to room temperature and was run under nitrogen atmosphere for 12 h. Upon completion, the reaction was cooled to 0 ºC and water (30 mL) was added carefully drop wise followed by aqueous solution of Rochelle (potassium sodium tartrate) salt (50 mL). The aqueous layer was extracted with EtOAc (3 × 60 mL). The combined organic layer was dried with anhydrous Na_2SO_4 and concentrated under reduced pressure. The crude mixture was purified by column chromatography (40% Ethyl acetate in Hexane) to afford amine **60** as a yellow oil (370 mg, 23% yield). **^{1}H NMR** (400 MHz, $CDCl_3$) δ 5.79 (ddt, *J* = 16.9, 10.1, 6.6 Hz, 1H), 5.00 (dq, *J* = 17.1, 1.7 Hz, 1H), 4.94 (dq, *J* = 10.2, 1.5 Hz, 1H), 3.40 (d, *J* = 2.4 Hz, 2H), 2.67 (t, *J* = 7.3 Hz, 2H), 2.19 (t, *J* = 2.4 Hz, 1H), 2.09 (q, *J* = 8.0, 7.5 Hz, 2H), 1.56 (p, *J* = 7.4 Hz, 2H); **^{13}C NMR** (101 MHz, $CDCl_3$) δ 138.4, 114.8, 82.3, 71.3, 48.2, 38.2, 31.5, 29.0. Spectral data are in accordance with the reported data[8].

Synthesis and characterization for compound 61

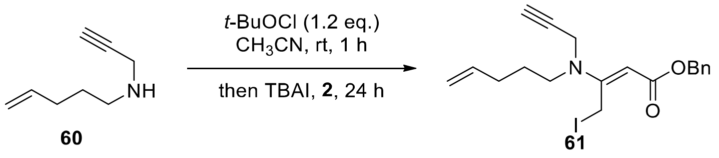

To an oven-dried 10 mL vial wrapped with aluminum foil and equipped with a stir bar was added amine **60** (13.5 mg, 0.11 mmol, 1.1 equiv), *t*-BuOCl (14.0 μL, 0.12 mmol, 1.2 equiv), and CH_3CN (2.0 mL) under argon atmosphere. The reaction mixture was stirred for 1 h at room temperature. Then, TBAI (55.4 mg, 0.15 mmol, 1.5 equiv), allenoate **2** (0.1 mmol, 1.0 equiv) was added and the reaction mixture was stirred for 24 h (TLC monitored the conversion of allenoate). The solvent was evaporated under reduced pressure and the crude was isolated by aluminum oxide column chromatography (1% to 5% Ethyl acetate in Hexane) to give compound **61** as a light-yellow oil (17.0 mg, 40% yield). **^{1}H NMR** (600 MHz, $CDCl_3$) δ 7.46 – 7.29 (m, 5H), 5.80 (ddd, *J* = 16.9, 11.5, 5.7 Hz, 1H), 5.14 (s, 2H), 5.09 – 4.99 (m, 4H), 4.90 (s, 1H), 4.03 (s, 2H), 3.35 (t, *J* = 7.7 Hz, 2H), 2.32

(s, 1H), 2.09 (q, *J* = 7.2 Hz, 2H), 1.77 (p, *J* = 7.4 Hz, 2H); **^{13}C NMR** (151 MHz, $CDCl_3$) δ 167.7, 156.8, 137.3, 137.0, 128.6, 128.3, 128.0, 115.9, 88.9, 78.4, 73.4, 65.3, 50.2, 39.8, 36.3, 30.9, 26.5; **HRMS** (ESI/[M+H]$^+$) *m/z* calcd. for $C_{19}H_{23}INO_2^+$: 424.0768, found [M+H]$^+$: 424.0763.

Synthesis and characterization for compound 64

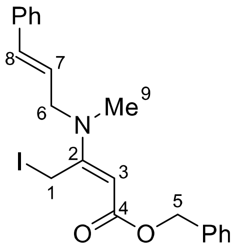

Benzyl (*E*)-3-(cinnamyl(methyl)amino)-4-iodobut-2-enoate (64)

To an oven-dried 10 mL vial wrapped with aluminum foil and equipped with a stir bar was added amine **63** (16.2 mg, 0.11 mmol, 1.1 equiv), *t*-BuOCl (14.0 μL, 0.12 mmol, 1.2 equiv), and CH_3CN (2.0 mL) under argon atmosphere. The reaction mixture was stirred for 1 h at room temperature. Then, TBAI (55.4 mg, 0.15 mmol, 1.5 equiv), allenoate **2** (0.1 mmol, 1.0 equiv) was added and the reaction mixture was stirred for 5 h (TLC monitored the conversion of allenoate). The solvent was evaporated under reduced pressure and the crude was isolated by silica gel chromatography (10% Ethyl acetate in Hexane) to give the enamine **64** as a yellowish oil (19.8 mg, 36% yield). **^{1}H NMR** (500 MHz, $CDCl_3$) δ 7.44 – 7.21 (m, 10H, *Ph*), 6.44 (d, *J* = 15.9 Hz, 1H, PhC*H*), 6.15 (dt, *J* = 15.9, 5.4 Hz, 1H, NCH_2-C*H*), 5.14 (s, 2H, Bn-CH_2), 5.03 (s, 2H, I-CH_2), 4.80 (s, 1H, NC=C*H*), 4.12 (dd, *J* = 5.5, 1.8 Hz, 2H, NCH_2), 2.96 (s, 3H, N-CH_3); **^{13}C NMR** (125 MHz, $CDCl_3$) δ 167.8 (*C*=O), 158.0 (N-*C*=C), 137.1 (*Ph*), 136.1 (*Ph*), 132.2 (Ph-*C*H=C), 128.7 (*Ph*), 128.5 (*Ph*), 128.2(*Ph*), 128.0 (*Ph*), 127.9 (*Ph*), 126.5 (*Ph*), 123.9 (Ph-CH=*C*H-), 87.0 (N-C=*C*H), 65.1 (Ph-*C*H_2-O), 53.5 (N-*C*H_2-CH), 37.9 (N-*C*H_3), 36.3 (C-*C*H_2-I); **HRMS** (ESI/[M+H]$^+$) *m/z* calcd. for $C_{21}H_{23}INO_2^+$: 448.0768, found [M+H]$^+$: 448.0772; **FTIR** (neat) v_{max} 2929, 2853, 1732, 1600, 1496, 1453, 1379, 1295, 1232, 1137, 1009, 967, 757, 692 cm^{-1}.

Synthesis and characterization for compound 72

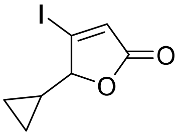

5-Cyclopropyl-4-iodofuran-2(5*H*)-one (72)

To an oven-dried 10 mL vial wrapped with aluminum foil and equipped with a stir bar was added allenote **71** (21.5 mg, 0.1 mmol, 1.0 equiv), CH_3CN (2.0 mL) and NIS (27 mg, 1.2 equiv) under argon atmosphere. The reaction mixture was stirred for 36 h at room temperature. The solvent was evaporated under reduced pressure and the crude was isolated by silica gel chromatography (5% Ethyl acetate in Hexane) to give the lactone **72** (5.8 mg, 23% yield). **^{1}H NMR** (500 MHz, $CDCl_3$) δ

6.53 (d, J = 1.7 Hz, 1H), 4.38 (dd, J = 8.1, 1.7 Hz, 1H), 1.06 – 0.91 (m, 1H), 0.86 – 0.75 (m, 1H), 0.75 – 0.61 (m, 2H), 0.61 – 0.42 (m, 1H); **^{13}C NMR** (125 MHz, $CDCl_3$) δ 170.8, 129.7, 124.6, 90.8, 13.4, 3.8, 1.8; **HRMS (**ESI/[M+H]$^+$) *m/z* calcd. for $C_7H_8IO_2^+$: 250.9564, found [M+H]$^+$: 250.9557; **FTIR** (neat) ν_{max} 1791, 1765, 1187, 962, 743 cm^{-1}.

7. DFT Calculation

Computational method: All the density functional theory (DFT) calculations were carried out using Gaussian 16 package[9]. Geometry optimizations were performed with the M06-2X functional[10–12] and def2-SVP[13,14] basis set. The single-point energy calculations were performed with the M06-2X functional and def2-TZVPP[13,14] basis set. To introduce the dispersion interaction, the functional was corrected with the empirical dispersion term (known as Grimme-D3)[15], as implemented in Gaussian16 software package for both geometry optimizations and single-point calculations. The solvent effect was introduced through SMD[16] universal solvation model with acetonitrile and SMD18 correction[17] was applied by modifying new Coulomb radii of I atom as 2.74 Å. Intrinsic reaction coordinate (IRC)[18] calculations were conducted to verify the transition state connects the correct reactant and product. The 3D structures were drawn using CYLview20 software (Supplementary Fig. 8)[19]. The single-point energies were corrected by the thermal correction to Gibbs free energies (TCG, obtained from frequency calculations) and reported as the free energy in this work (Supplementary Table 3). All calculations were performed at 1 atm and 298.15 K. Harmonic frequency calculations were performed to ensure each structure is either an energy minimum (no imaginary frequency) or a transition state (only one imaginary frequency) (Supplementary Table 4). Electrostatic potential surfaces and natural population analysis of atomic charges related to **SM-Cl**, **INT3**, **53-Cl** were performed at the same level with single-point energy calculations (Supplementary Figs. 9–11).

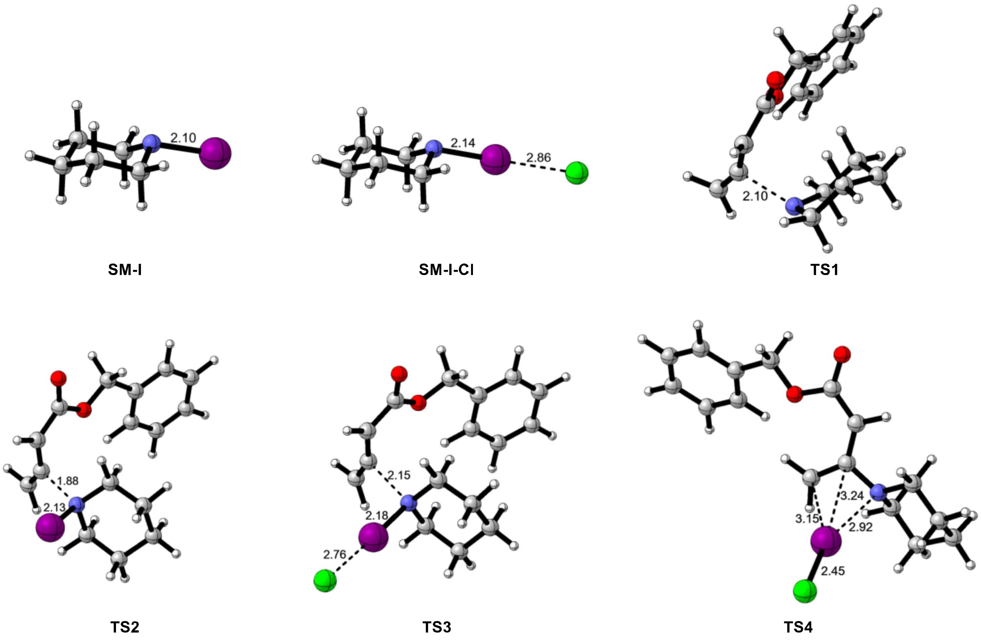

Supplementary Fig. 8. Computed structures **SM-I**, **SM-I-Cl**, **TS1**, **TS2**, **TS3**, **TS4**, selected bond distance (Å) (color code, C: grey, N: blue, O: red, H: white, I: purple, Cl: green).

Supplementary Table 3. TCG and Electronic Energies (EE) of related compounds and transition states.

Label	TCG (Hartree)	EE (Hartree)
Cl anion	-0.015023	-460.364085
I radical	-0.017503	-297.611070
**SM-I**	0.115690	-548.897329
**2**	0.141042	-575.587548
**INT1**	0.115869	-251.226933
**INT2**	0.284400	-826.879519
**INT3**	0.284055	-1124.471329
**SM-I-Cl**	0.111922	-1009.269893
**INT4**	0.281945	-1584.878465
**53-Cl***	0.281729	-1584.938508
**53**	0.284774	-1124.572821
**TS1**	0.278618	-826.804312
**TS2**	0.281411	-1124.465564
**TS3**	0.276900	-1584.846619
**TS4**	0.279696	-1584.858903

***53-Cl** complex is the product of **TS4**, and **53** would form when chlorine anion left.

Supplementary Table 4. Imaginary frequencies of related transition states.

Label	Imaginary Frequency
**TS1**	-390.34
**TS2**	-327.54
**TS3**	-252.05
**TS4**	-174.94

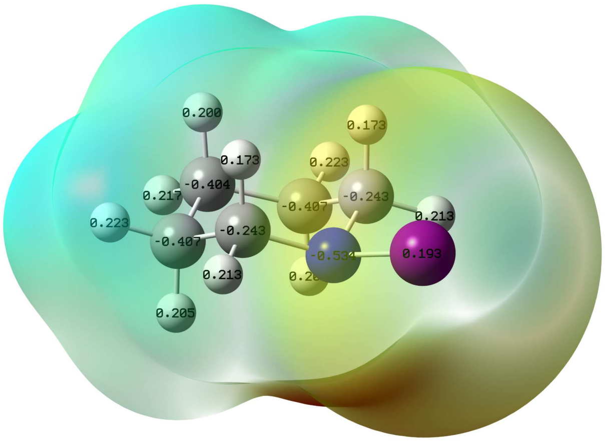

Supplementary Fig. 9. Electrostatic potential surfaces and natural population analysis of atomic charges related to **SM-I**.

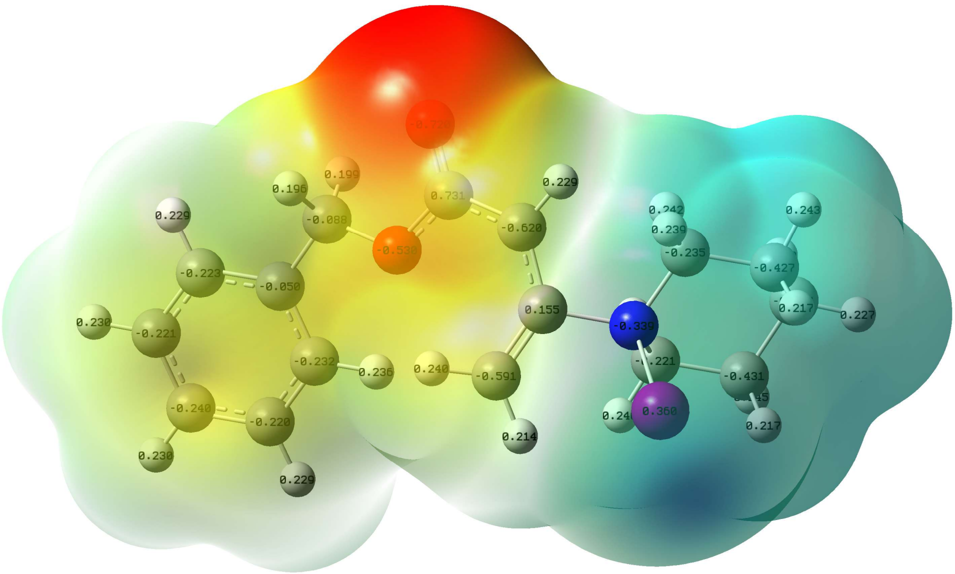

Supplementary Fig. 10. Electrostatic potential surfaces and natural population analysis of atomic charges related to **INT3**.

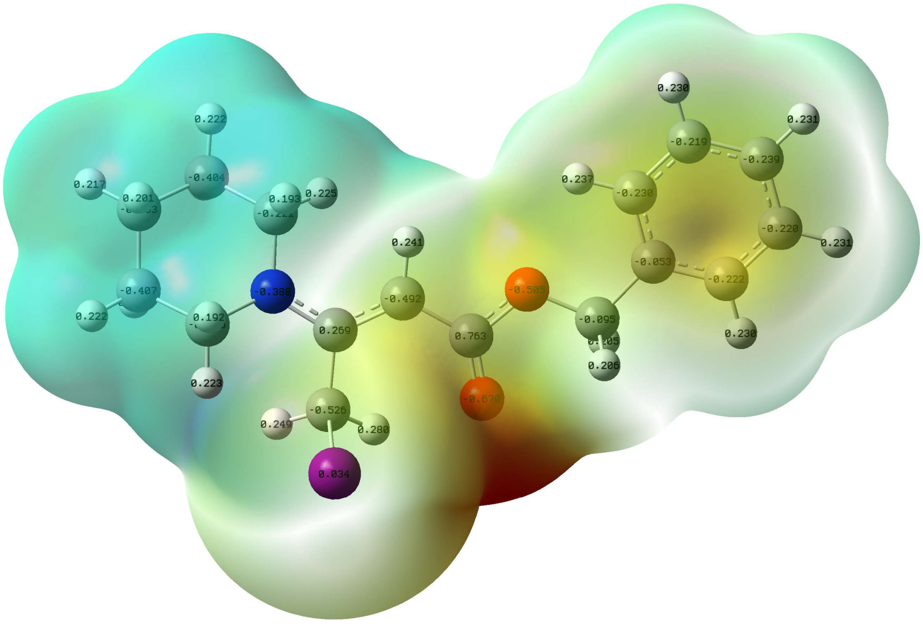

Supplementary Fig. 11. Electrostatic potential surfaces and natural population analysis of atomic charges related to **53-Cl**.

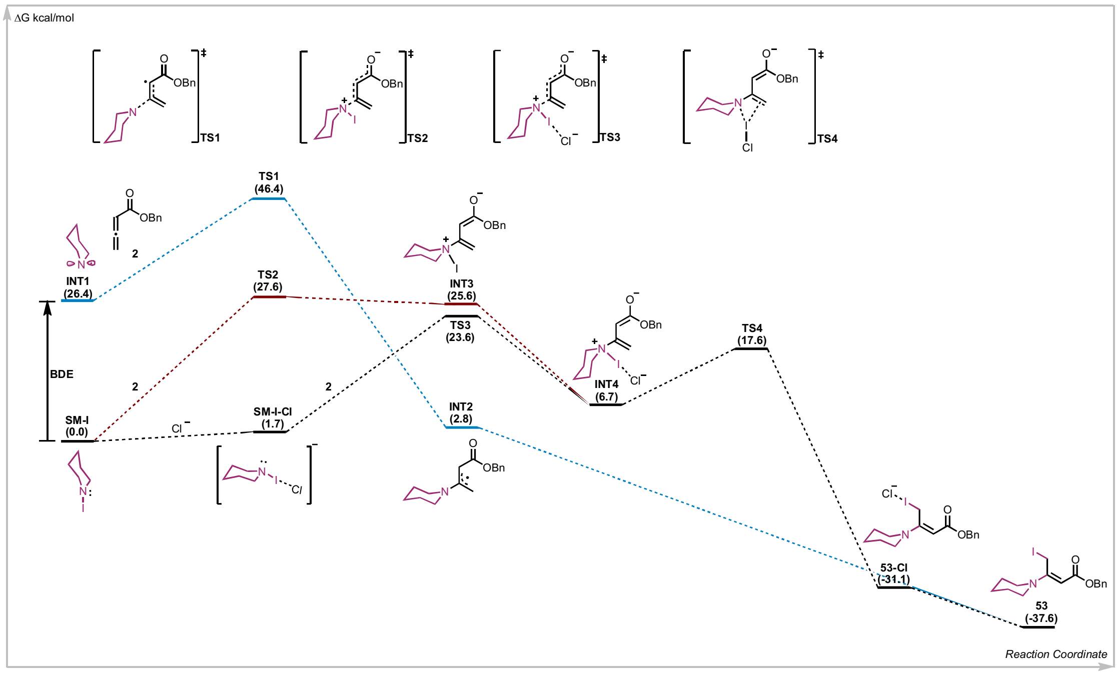

Supplementary Fig. 12. Free energy profiles (kcal/mol) of the radical and ionic processes for the formation of **53** at the Mo6-2x-D3/def2-TZVPP (SMD18, acetonitrile) //M06-2x-D3/def2-SVP (SMD18, acetonitrile) level.

The free energies of **SM-I-Cl** and **53-Cl** are 1.7 kcal/mol and 6.5 kcal/mol higher separately than **SM-I** and **53** through halogen bond (Supplementary Fig. 12). Interestingly, the free energy of

zwitterionic intermediate **INT4** is 18.9 kcal/mol lower than zwitterionic intermediate **INT3**. Thus, **INT4** is extremely stabilized by halogen bond. We propose it is because of the difference of charge distribution between R_3N and R_4N^+, which further leads to the charge differences on iodine atoms. Based on the natural population analysis of atomic charges on iodine (+0.034 for **53**, +0.193 for **SM-Cl**, +0.360 for **INT3**), electrostatic interaction between iodine atom on **INT3** and chloride anion to form **INT4** should be the strongest one. This is also supported by electrostatic potential surfaces related to **SM-I**, **INT3**, **53-Cl**. An obvious sigma hole could be found on the terminal position of iodine atom of **INT3** (Supplementary Fig. 10, blue: positive charge, red: negative charge) compared with **SM-I** and **53-Cl**. Thus, a chloride anion could form a stronger halogen bond with **INT3** to form stabilized zwitterionic intermediate **INT4**.

IV. Supplementary Figures

8. Copies of NMR Spectra

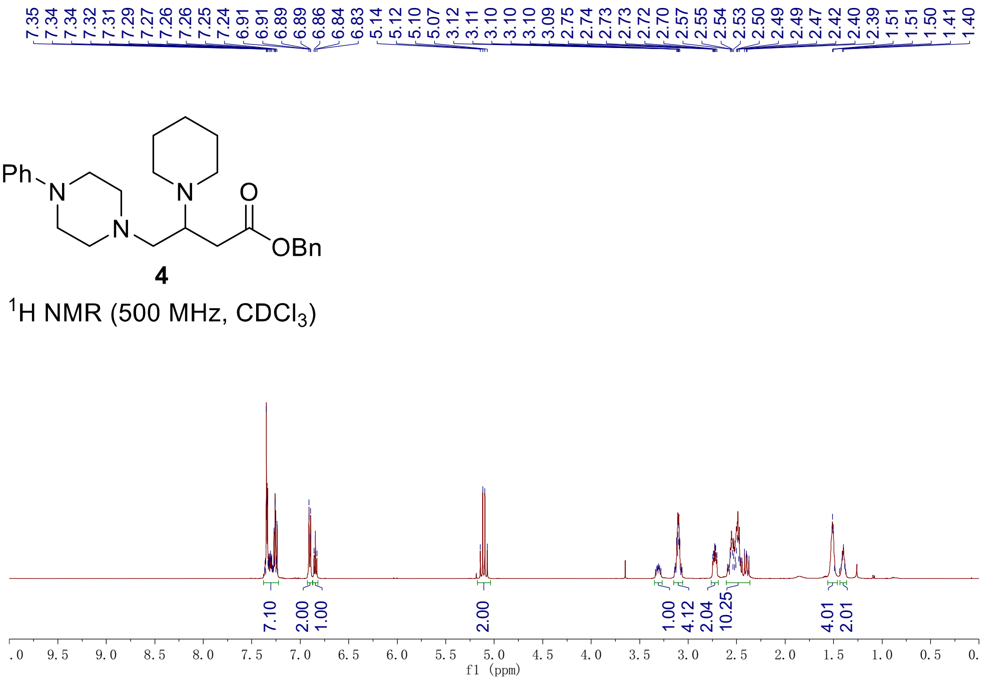

Supplementary Fig. 13. ^{1}H NMR (500 MHz, $CDCl_3$) spectrum of compound **4**.

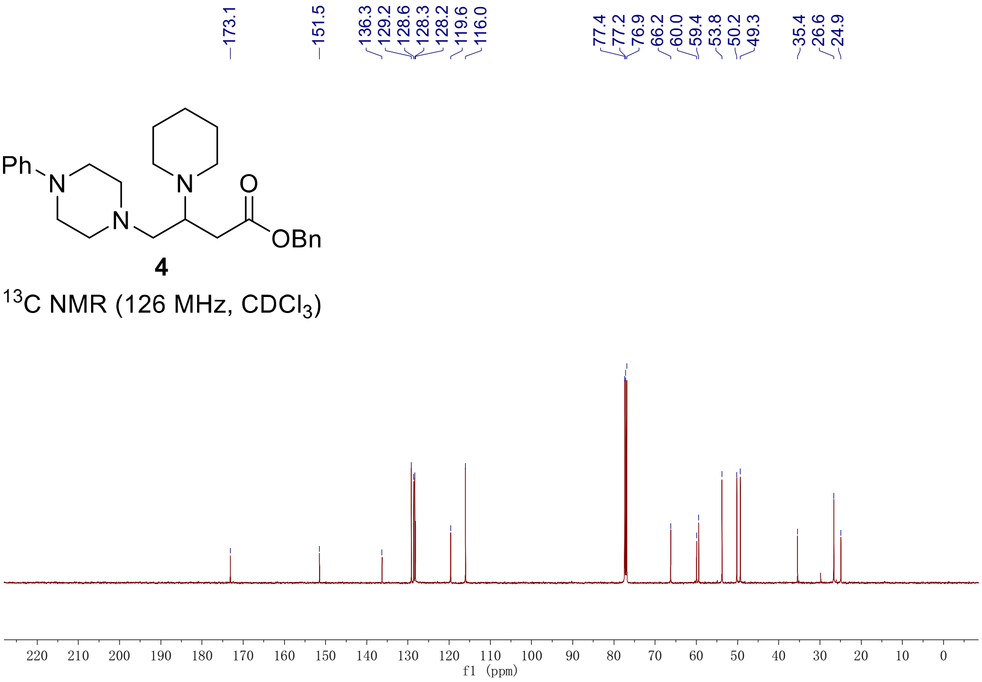

Supplementary Fig. 14. ^{13}C NMR (126 MHz, $CDCl_3$) spectrum of compound **4**.

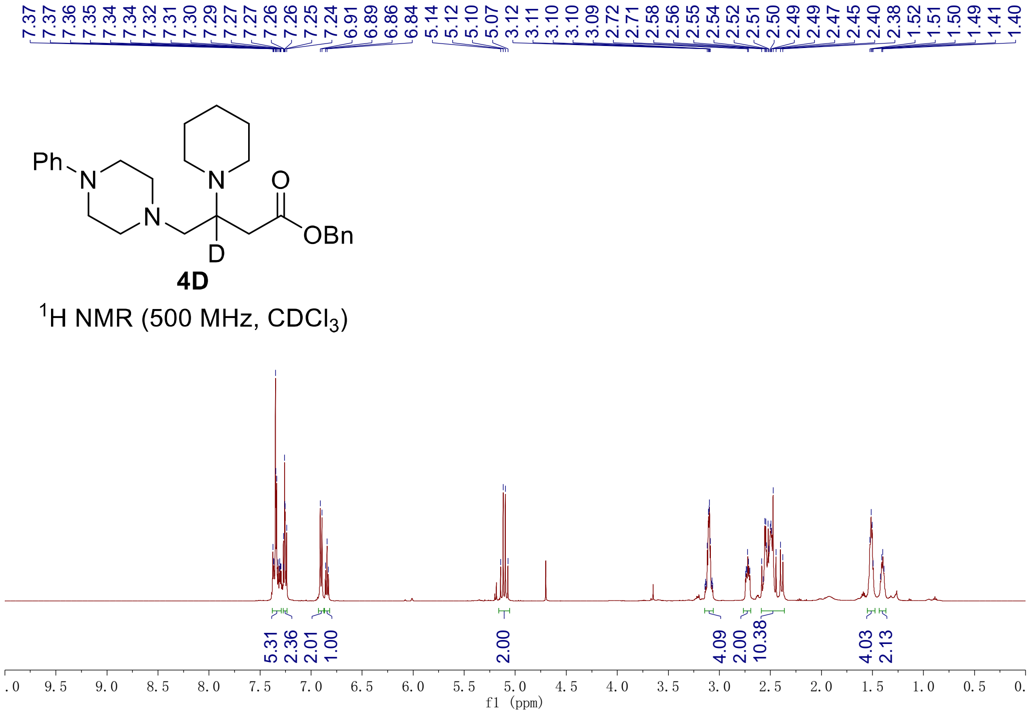

Supplementary Fig. 15. ^{1}H NMR (500 MHz, $CDCl_3$) spectrum of compound **4D**.

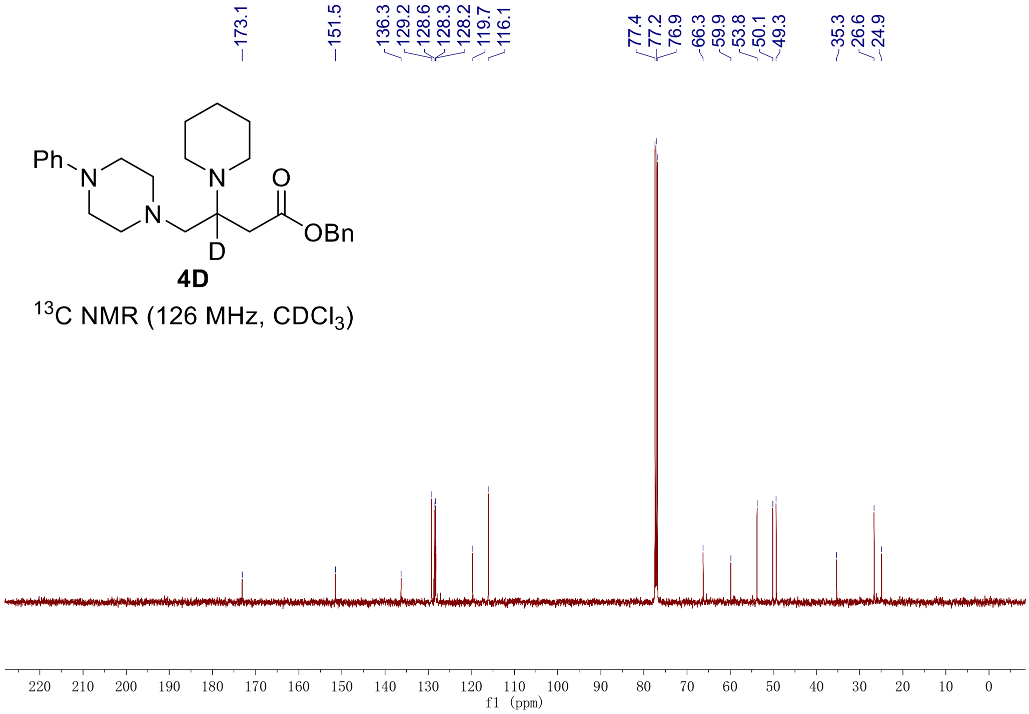

Supplementary Fig. 16. ^{13}C NMR (126 MHz, $CDCl_3$) spectrum of compound **4D**.

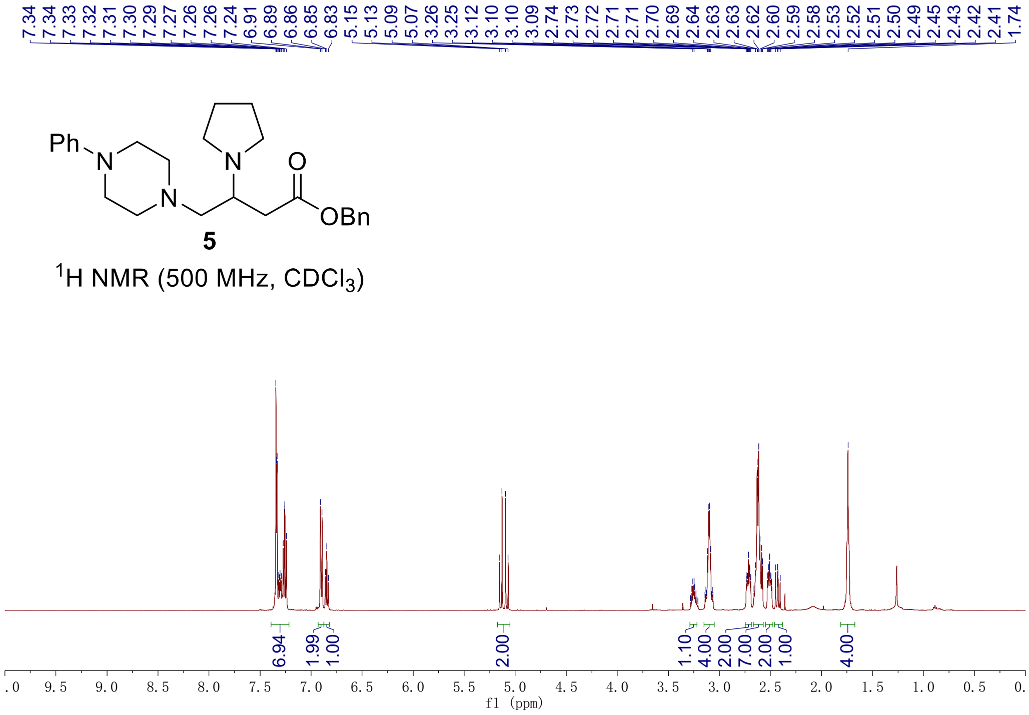

Supplementary Fig. 17. ^{1}H NMR (500 MHz, $CDCl_3$) spectrum of compound **5**.

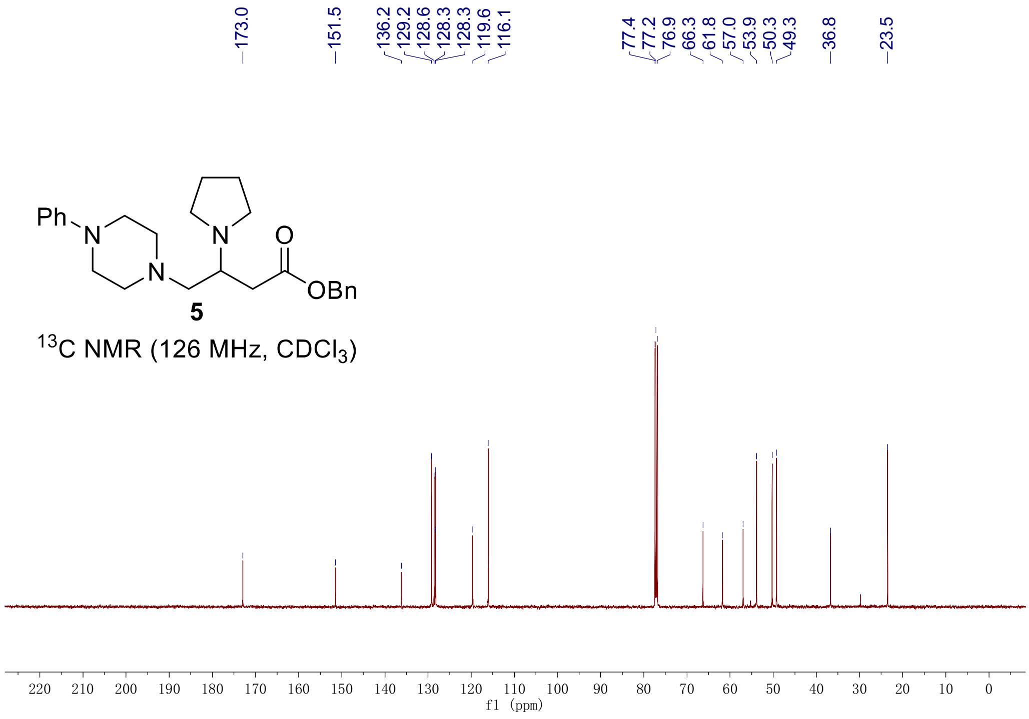

Supplementary Fig. 18. ^{13}C NMR (126 MHz, $CDCl_3$) spectrum of compound **5**.

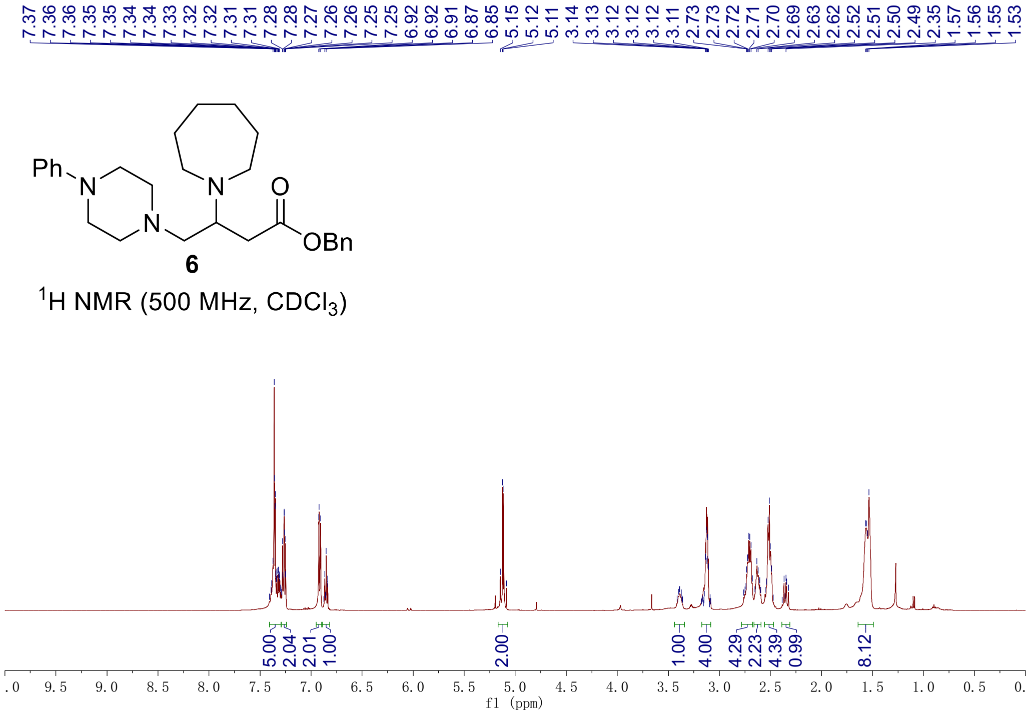

Supplementary Fig. 19. ^{1}H NMR (500 MHz, $CDCl_3$) spectrum of compound **6**.

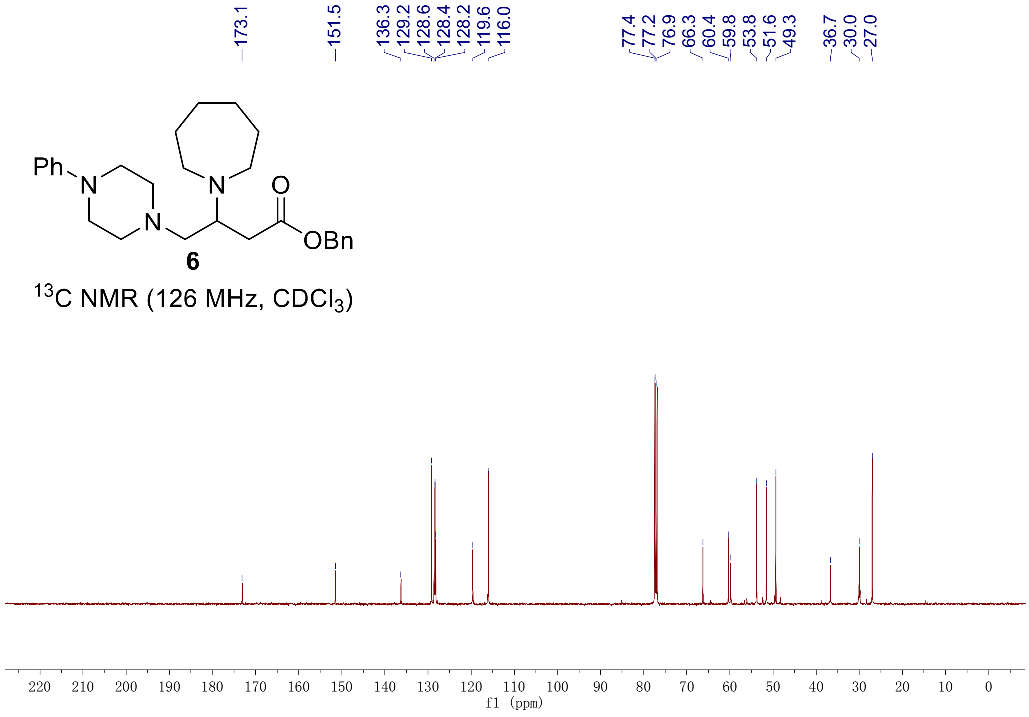

Supplementary Fig. 20. ^{13}C NMR (126 MHz, $CDCl_3$) spectrum of compound **6**.

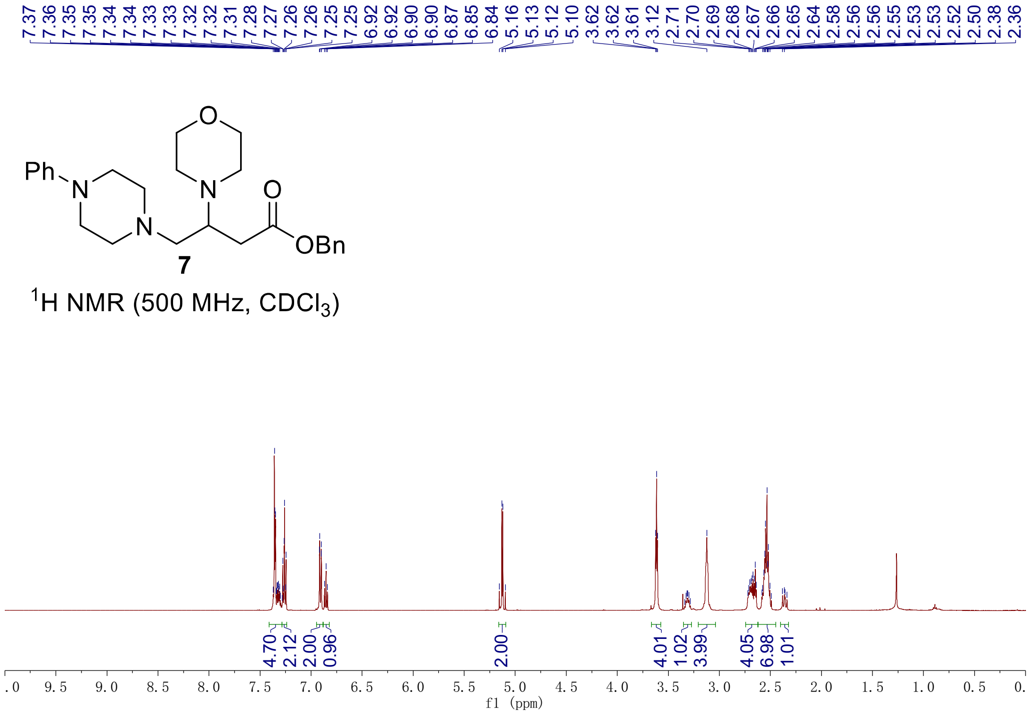

Supplementary Fig. 21. ^{1}H NMR (500 MHz, CDCl$_3$) spectrum of compound **7**.

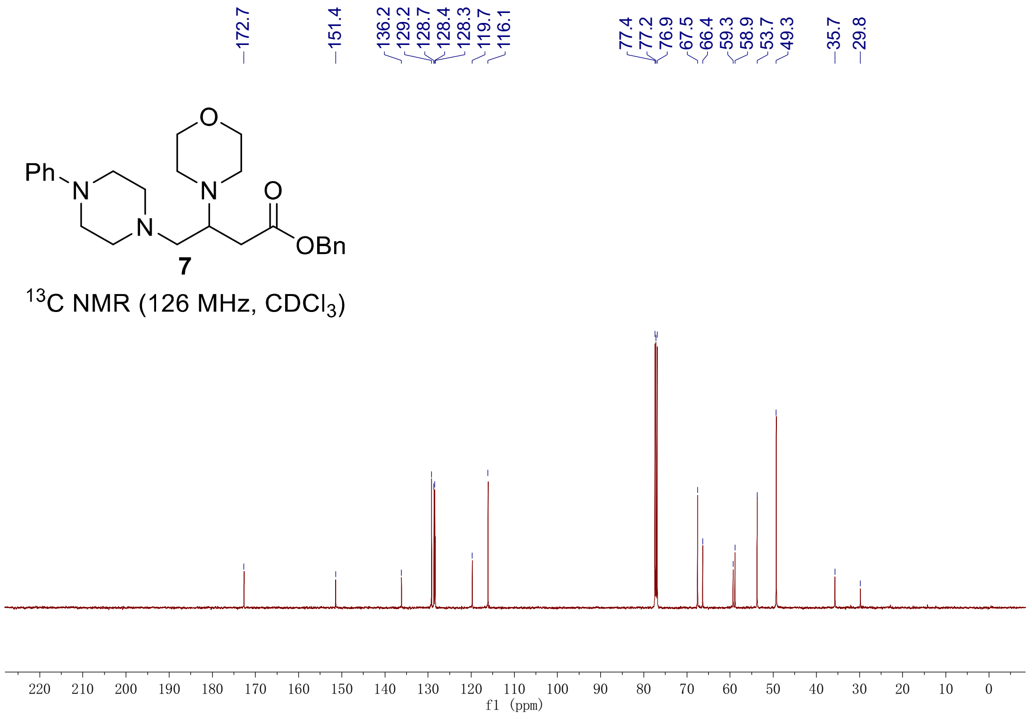

Supplementary Fig. 22. ^{13}C NMR (126 MHz, CDCl$_3$) spectrum of compound **7**.

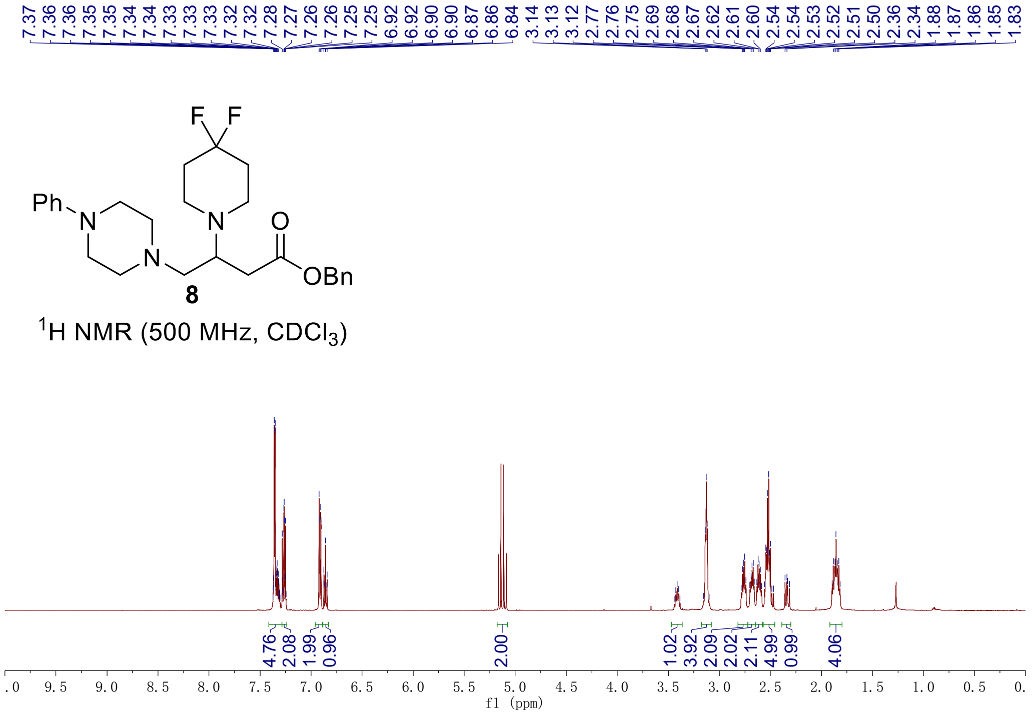

Supplementary Fig. 23. ^{1}H NMR (500 MHz, $CDCl_3$) spectrum of compound **8**.

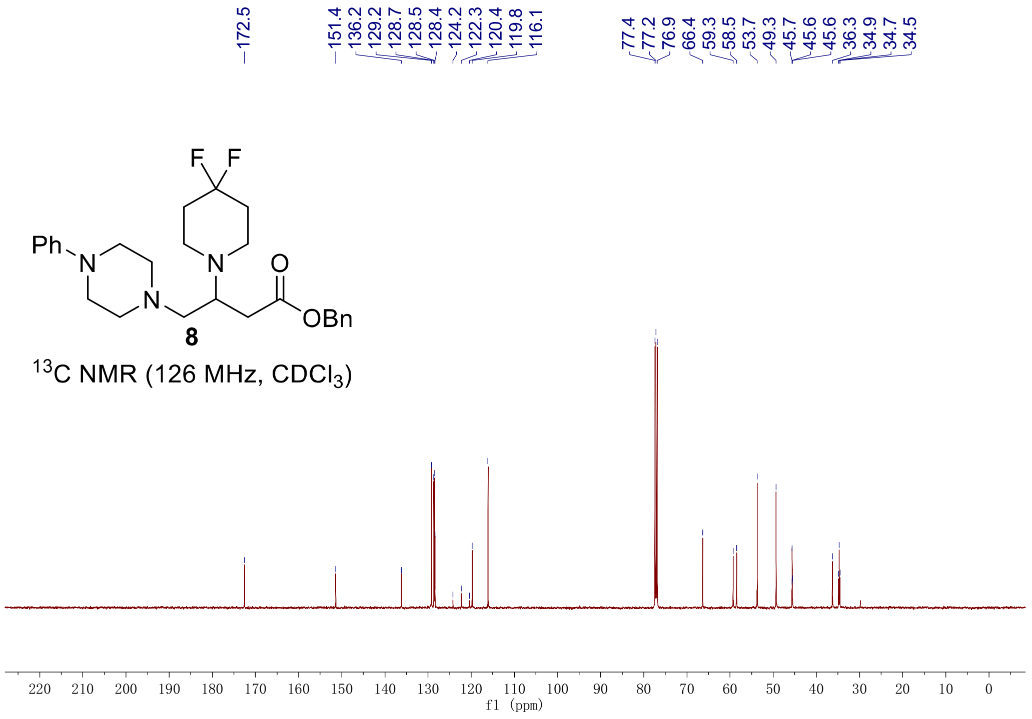

Supplementary Fig. 24. ^{13}C NMR (126 MHz, $CDCl_3$) spectrum of compound **8**.

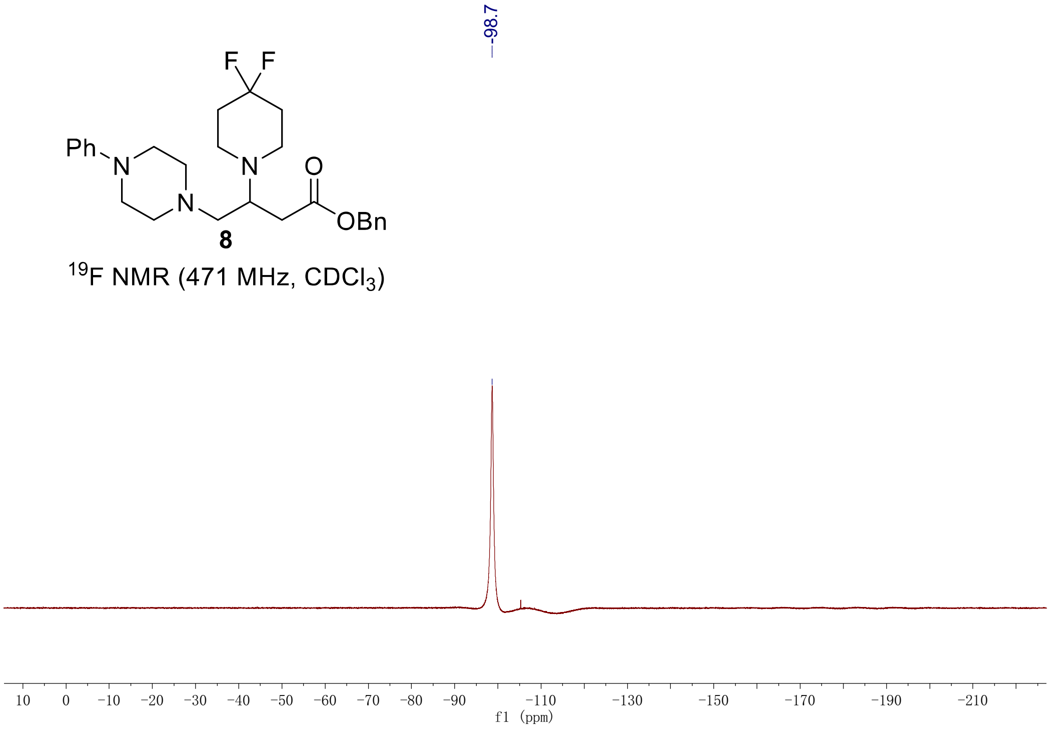

Supplementary Fig. 25. ^{19}F NMR (471 MHz, $CDCl_3$) spectrum of compound **8**.

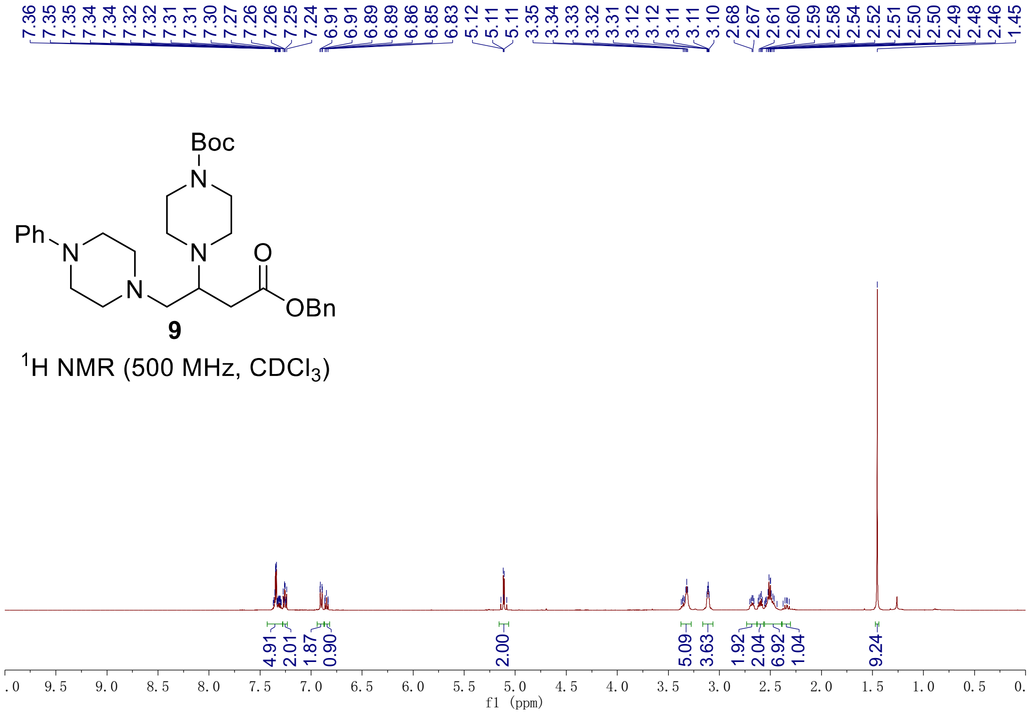

Supplementary Fig. 26. ^{1}H NMR (500 MHz, $CDCl_3$) spectrum of compound **9**.

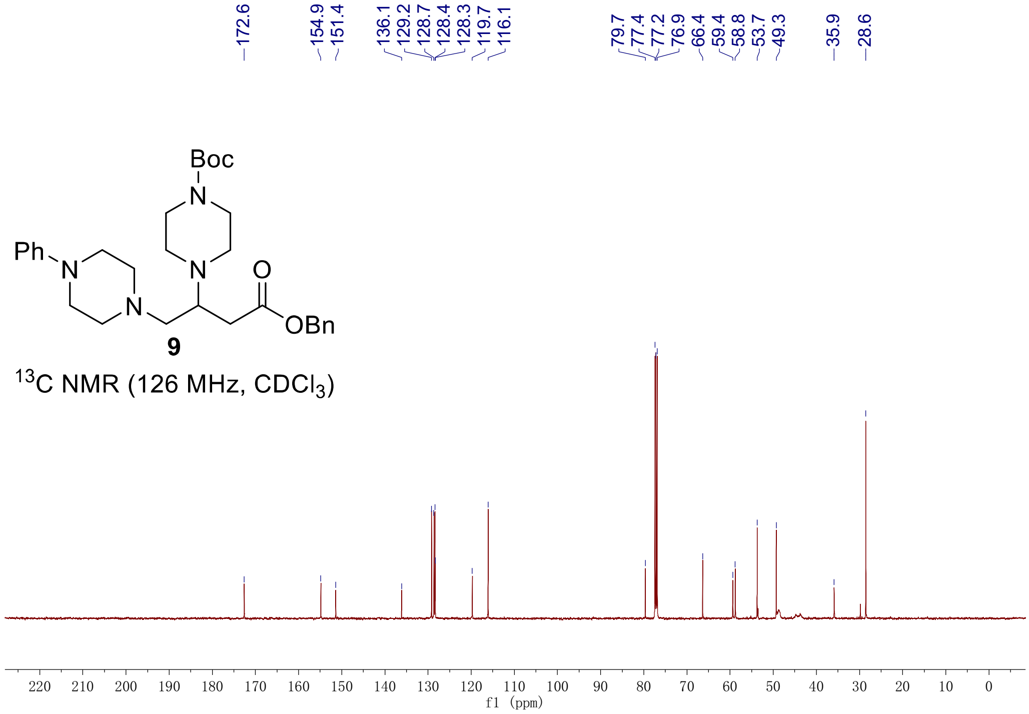

Supplementary Fig. 27. ^{13}C NMR (126 MHz, $CDCl_3$) spectrum of compound **9**.

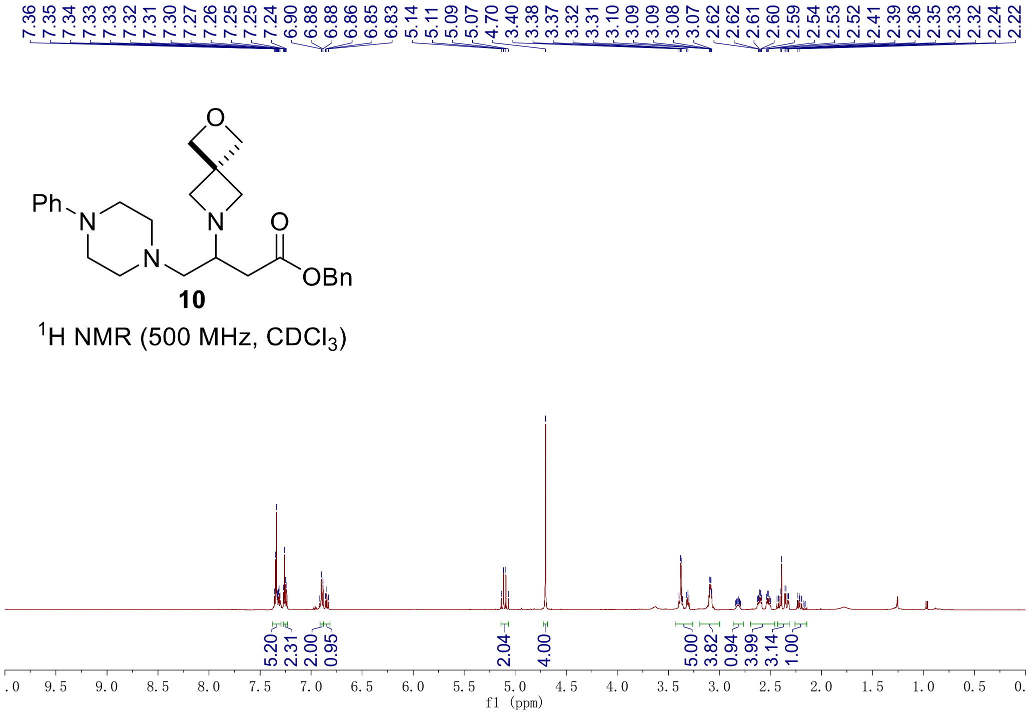

Supplementary Fig. 28. ^{1}H NMR (500 MHz, CDCl$_3$) spectrum of compound **10**.

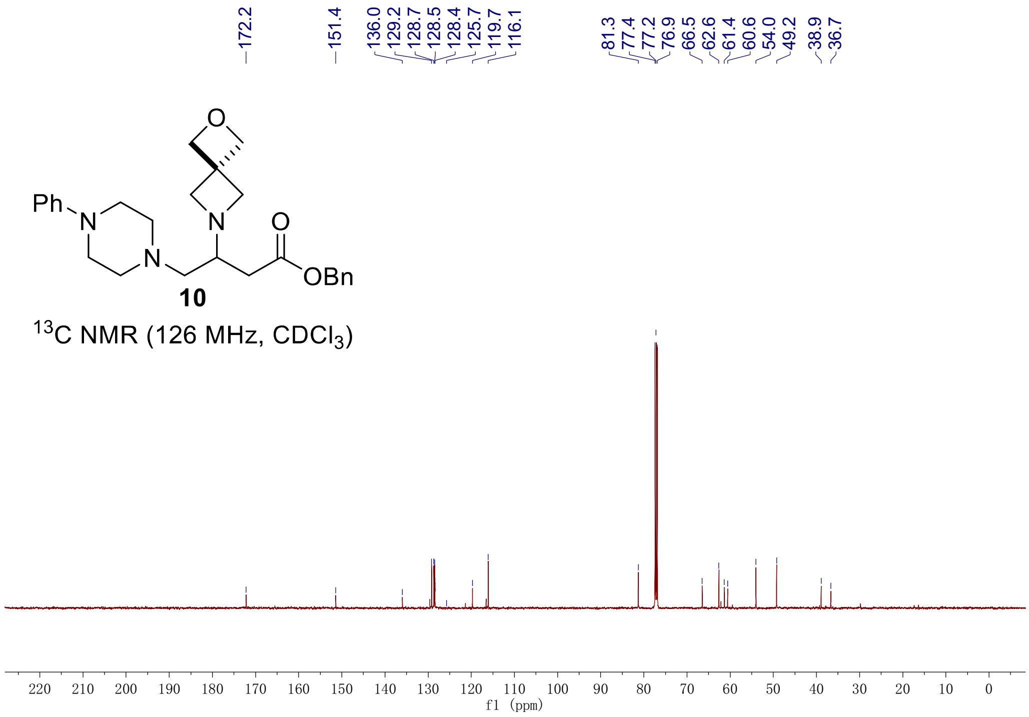

Supplementary Fig. 29. ^{13}C NMR (126 MHz, CDCl$_3$) spectrum of compound **10**.

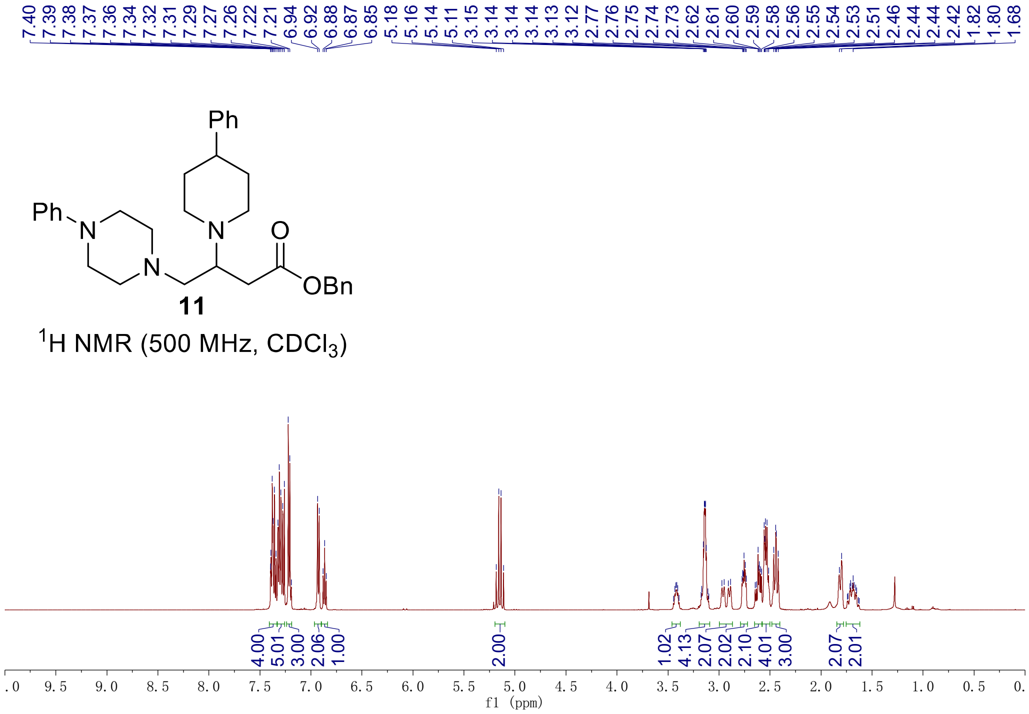

Supplementary Fig. 30. ^{1}H NMR (500 MHz, $CDCl_3$) spectrum of compound **11**.

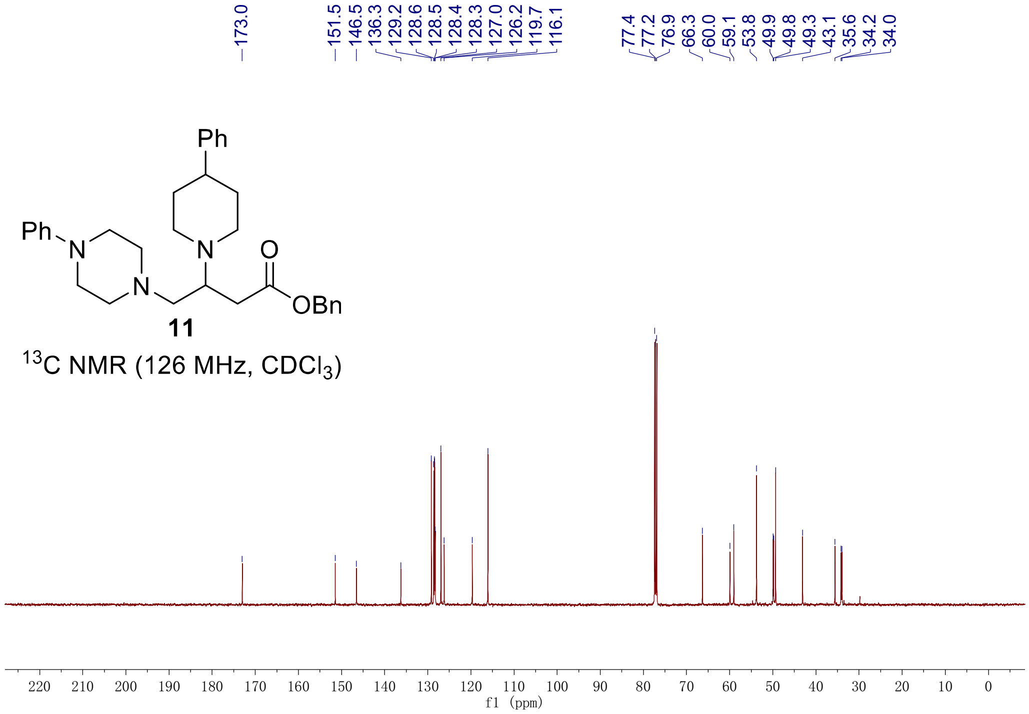

Supplementary Fig. 31. ^{13}C NMR (126 MHz, $CDCl_3$) spectrum of compound **11**.

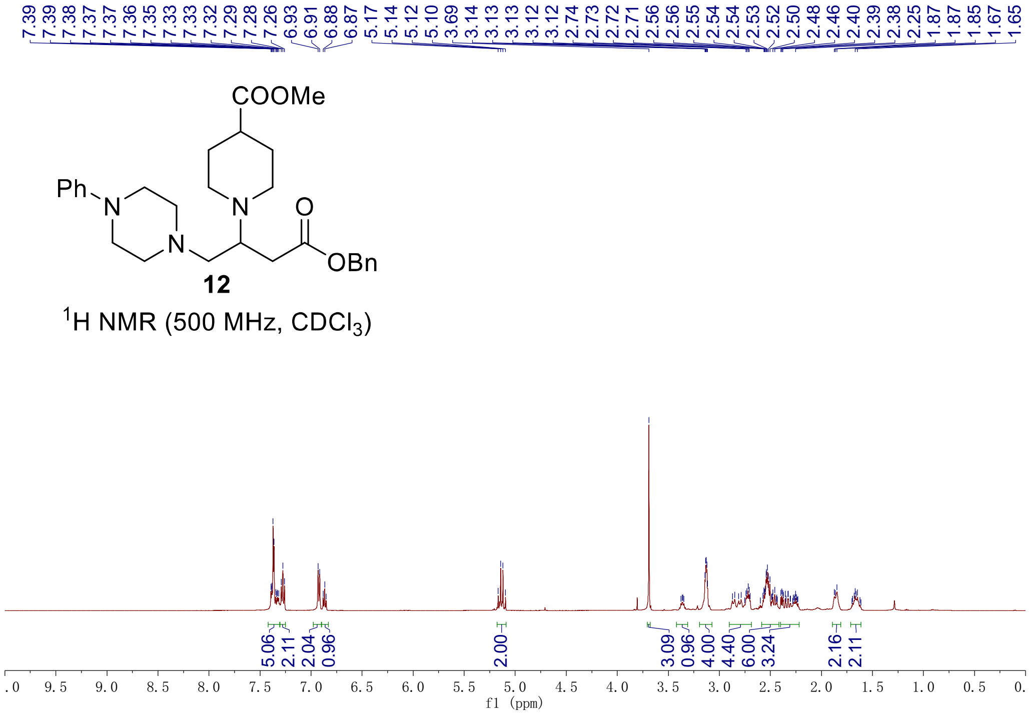

Supplementary Fig. 32. ^{1}H NMR (500 MHz, $CDCl_3$) spectrum of compound **12**.

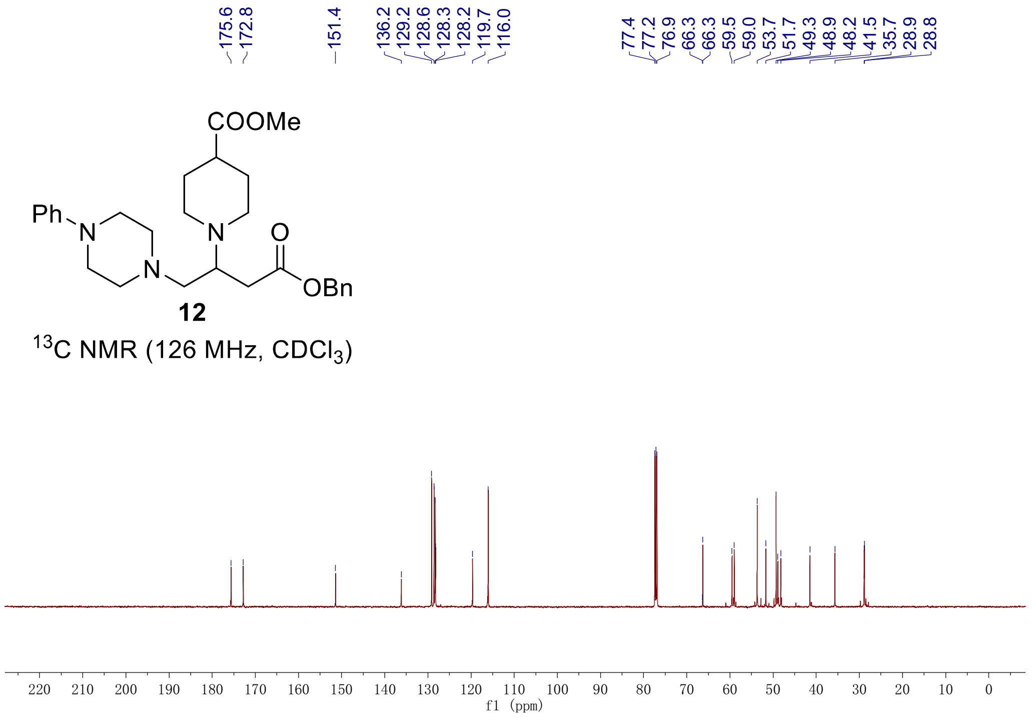

Supplementary Fig. 33. ^{13}C NMR (126 MHz, $CDCl_3$) spectrum of compound **12**.

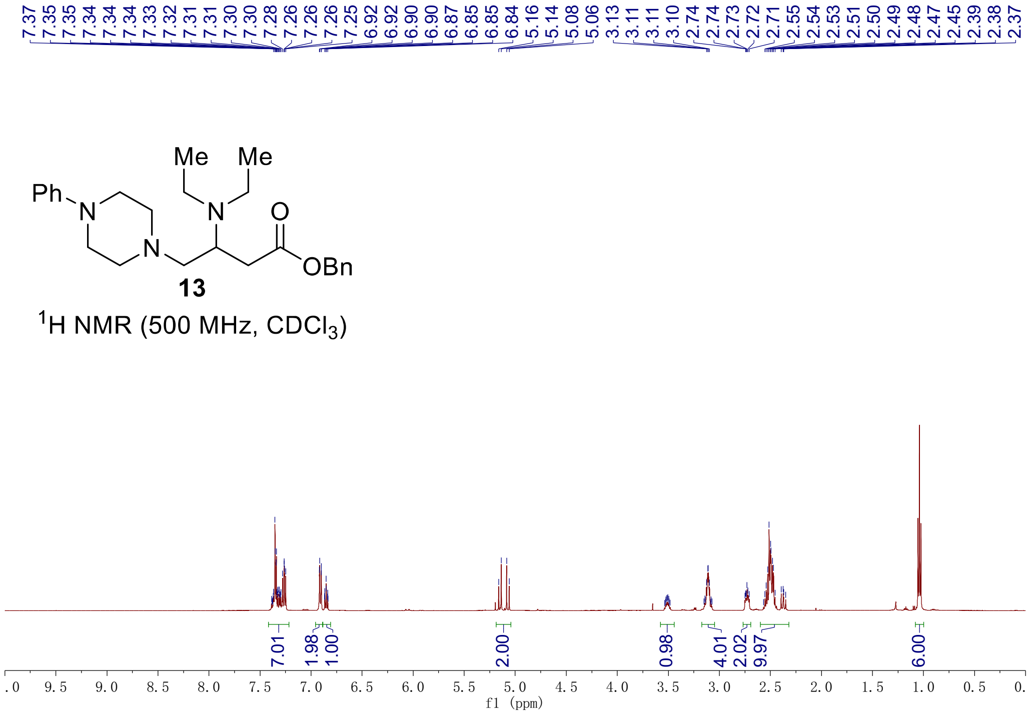

Supplementary Fig. 34. ^{1}H NMR (500 MHz, $CDCl_3$) spectrum of compound **13**.

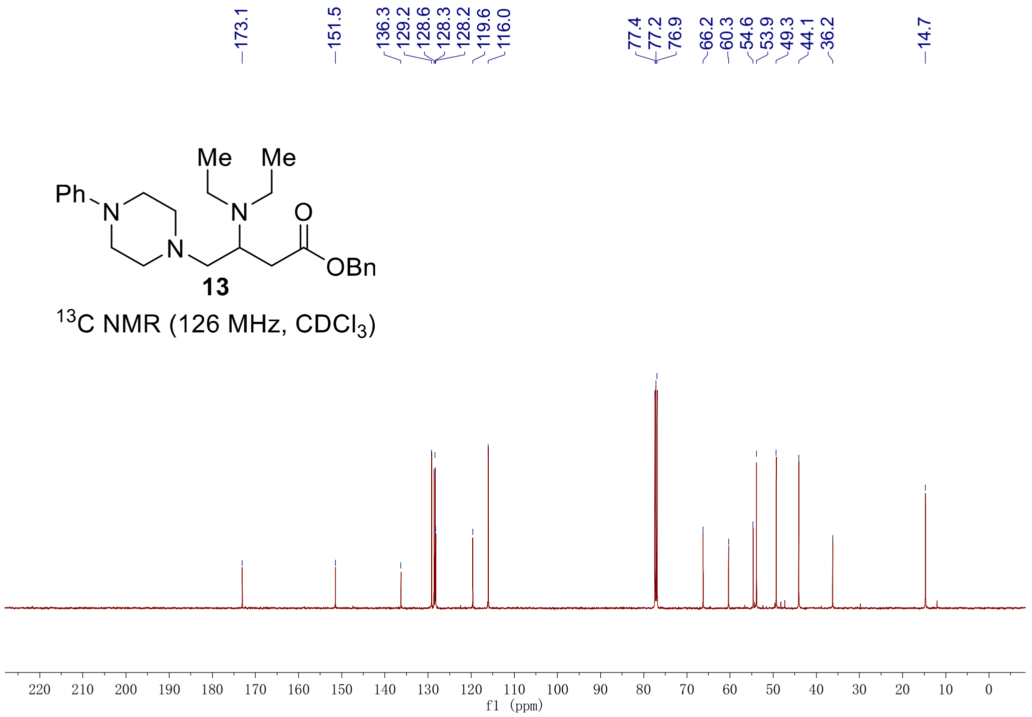

Supplementary Fig. 35. ^{13}C NMR (126 MHz, $CDCl_3$) spectrum of compound **13**.

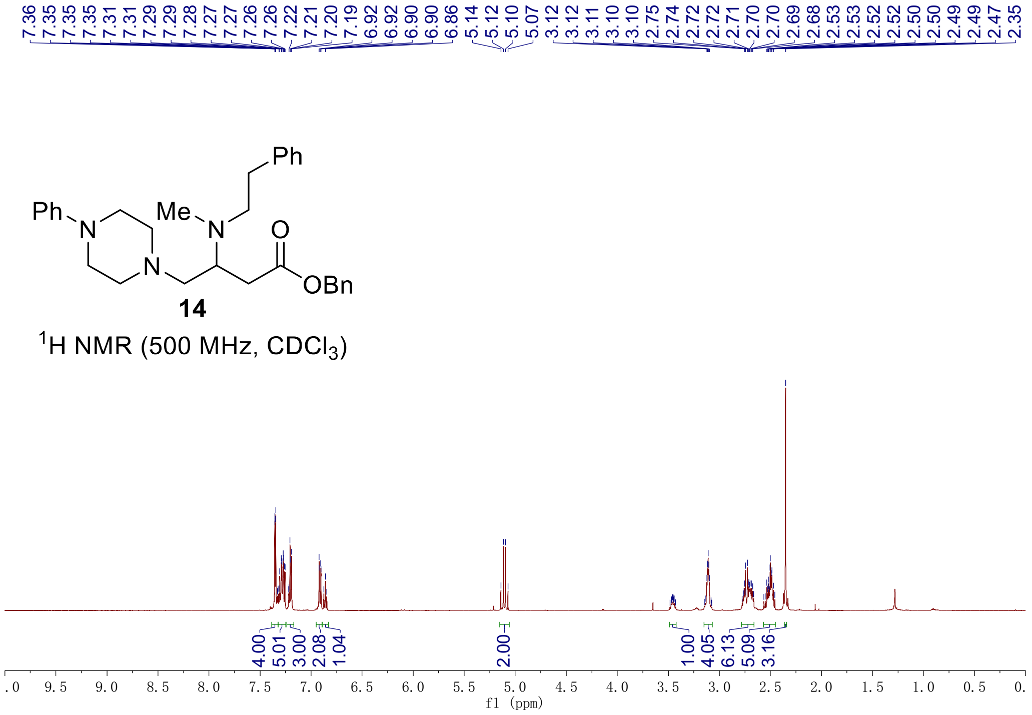

Supplementary Fig. 36. ^{1}H NMR (500 MHz, $CDCl_3$) spectrum of compound **14**.

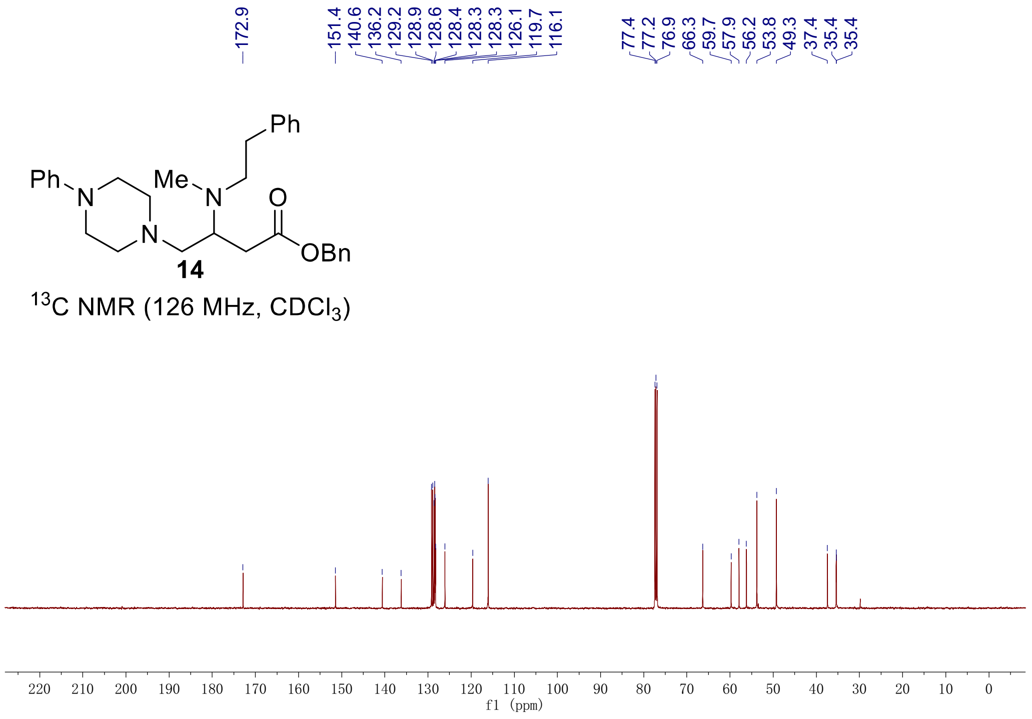

Supplementary Fig. 37. ^{13}C NMR (126 MHz, $CDCl_3$) spectrum of compound **14**.

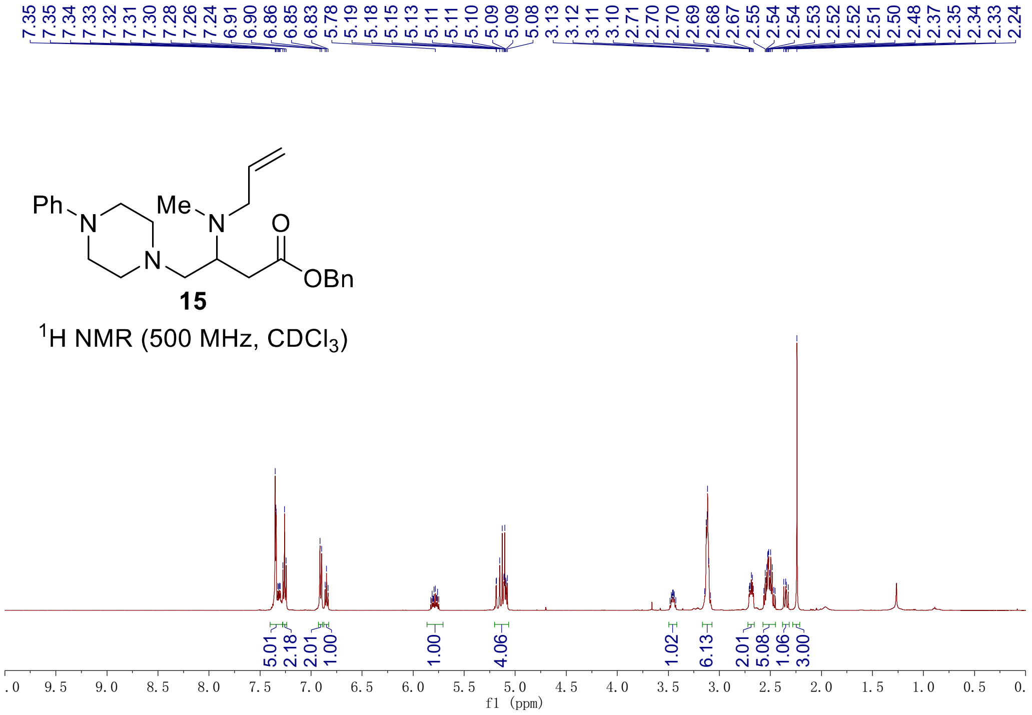

Supplementary Fig. 38. ^{1}H NMR (500 MHz, $CDCl_3$) spectrum of compound **15**.

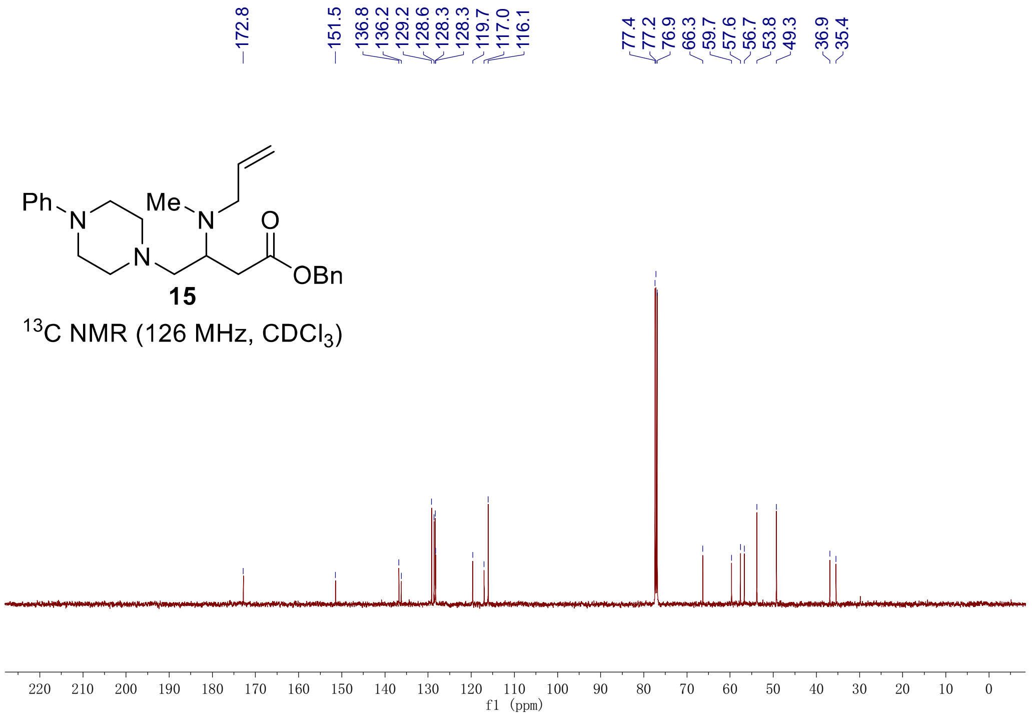

Supplementary Fig. 39. ^{13}C NMR (126 MHz, $CDCl_3$) spectrum of compound **15**.

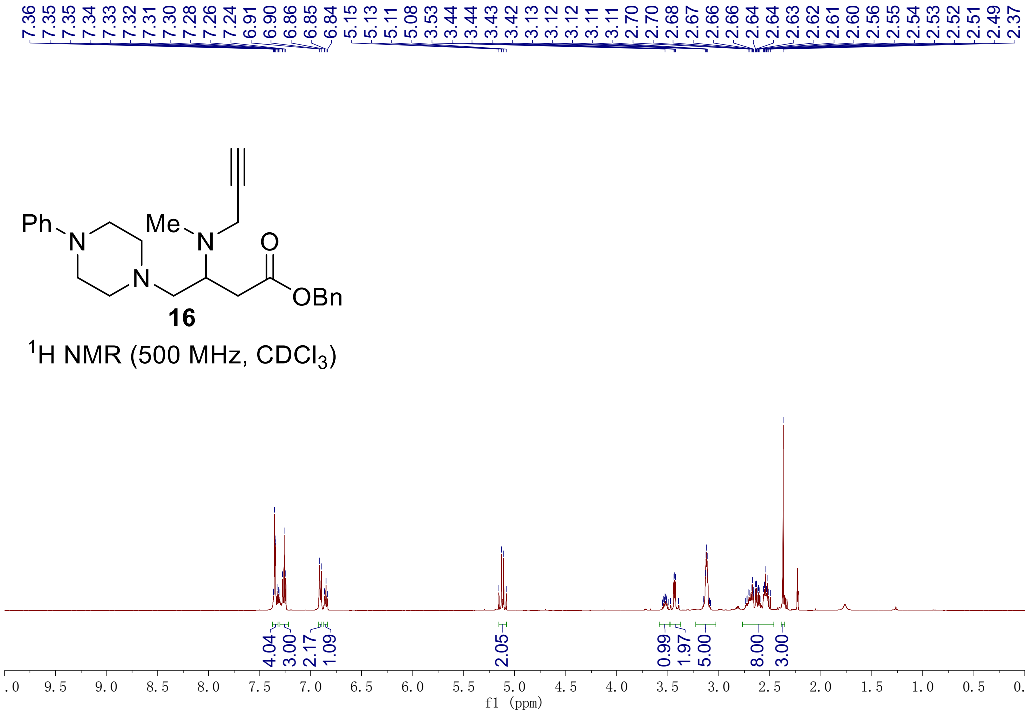

Supplementary Fig. 40. ^{1}H NMR (500 MHz, $CDCl_3$) spectrum of compound **16**.

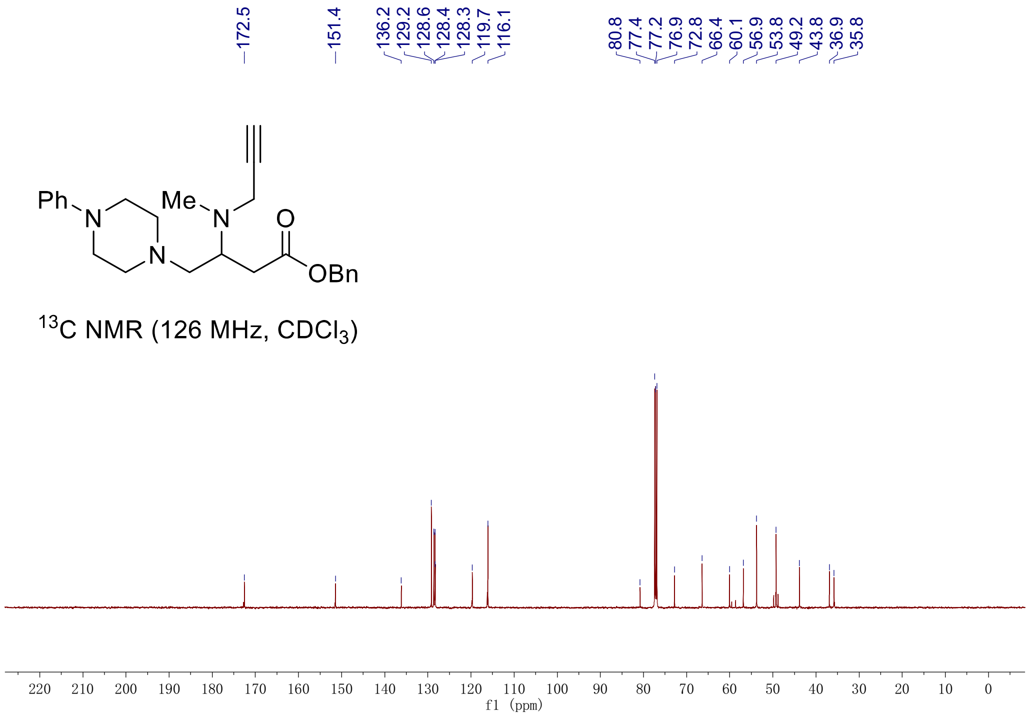

Supplementary Fig. 41. ^{13}C NMR (126 MHz, $CDCl_3$) spectrum of compound **16**.

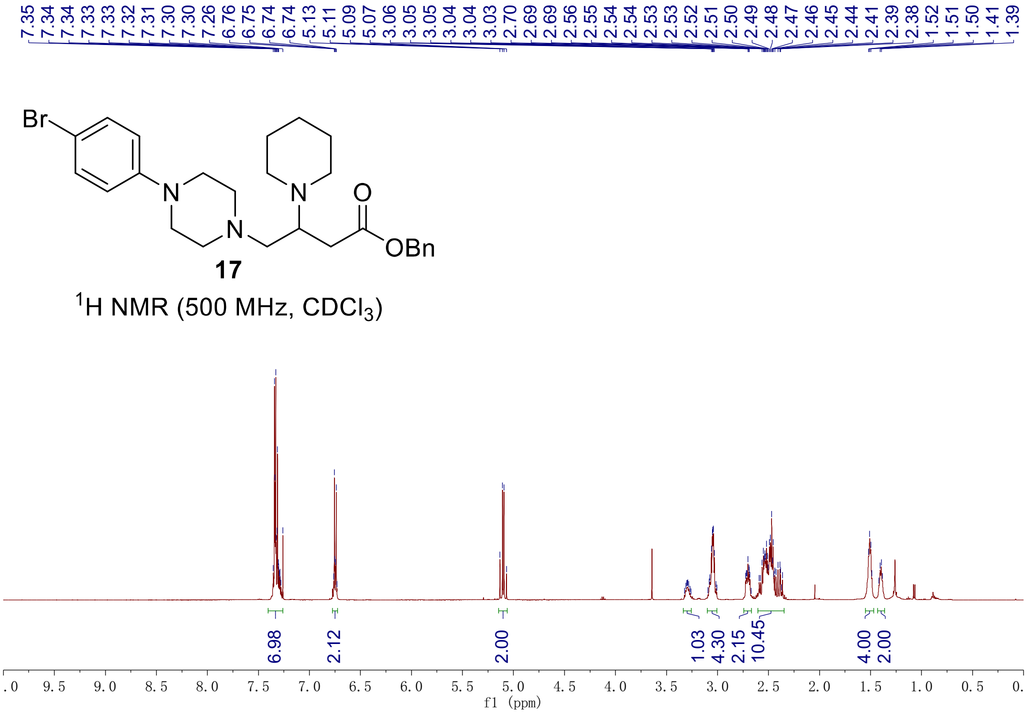

Supplementary Fig. 42. ^{1}H NMR (500 MHz, $CDCl_3$) spectrum of compound **17**.

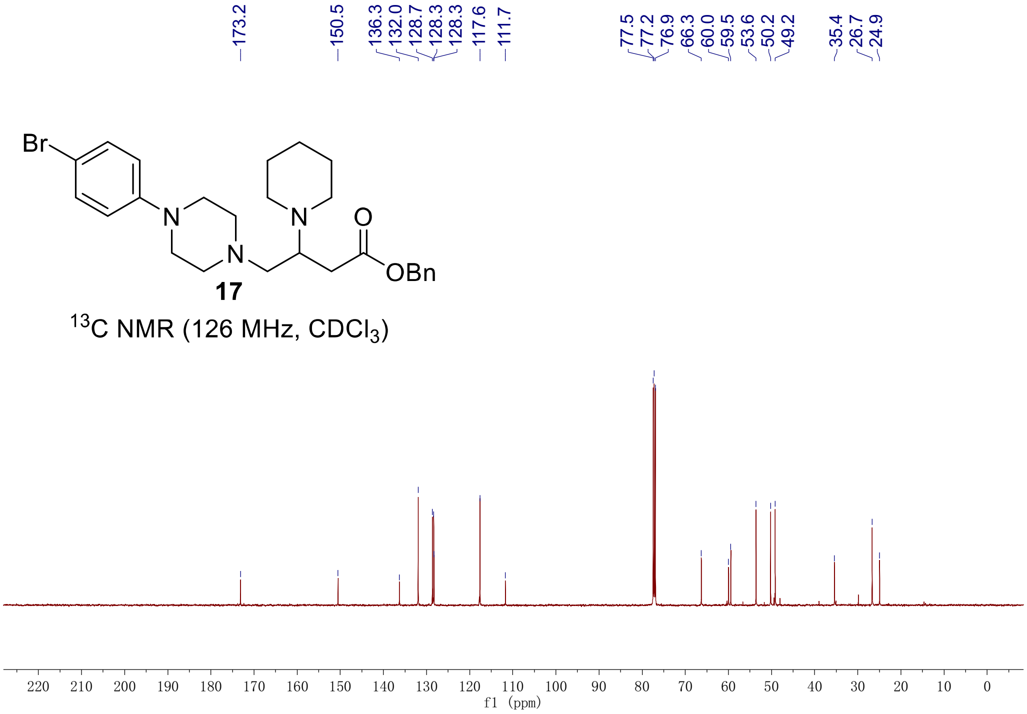

Supplementary Fig. 43. ^{13}C NMR (126 MHz, $CDCl_3$) spectrum of compound **17**.

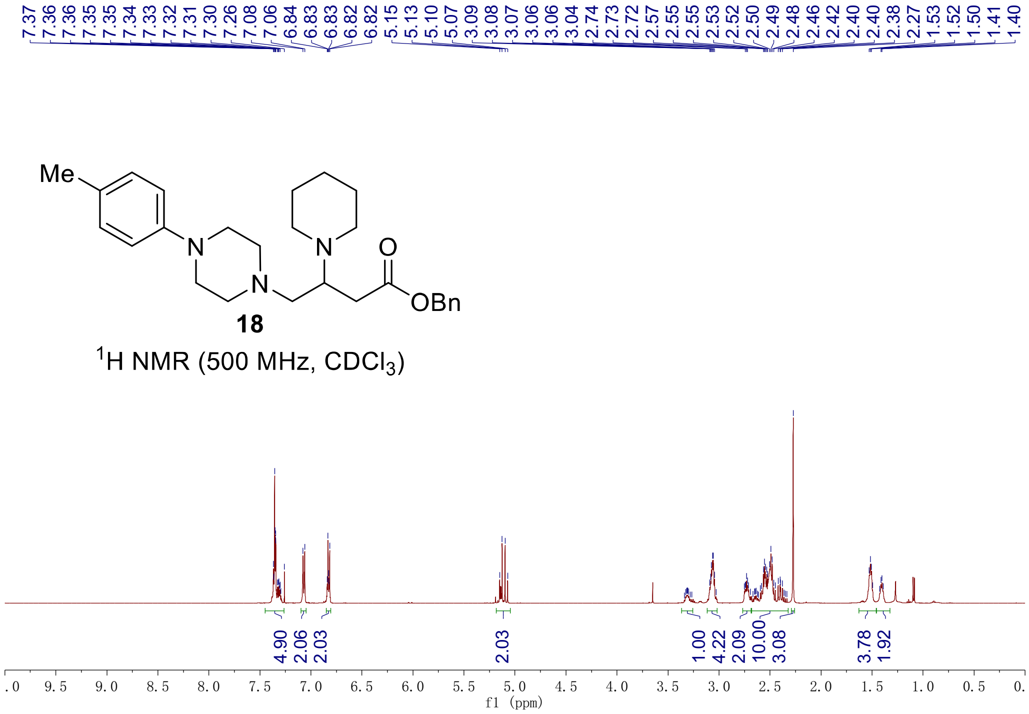

Supplementary Fig. 44. ^{1}H NMR (500 MHz, $CDCl_3$) spectrum of compound **18**.

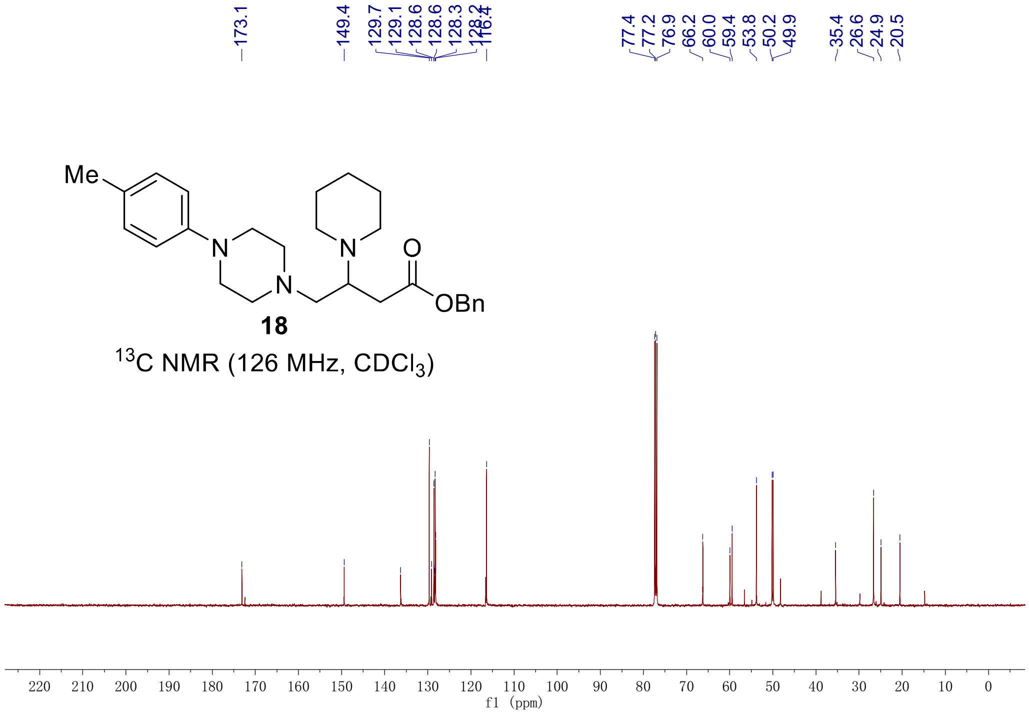

Supplementary Fig. 45. ^{13}C NMR (126 MHz, $CDCl_3$) spectrum of compound **18**.

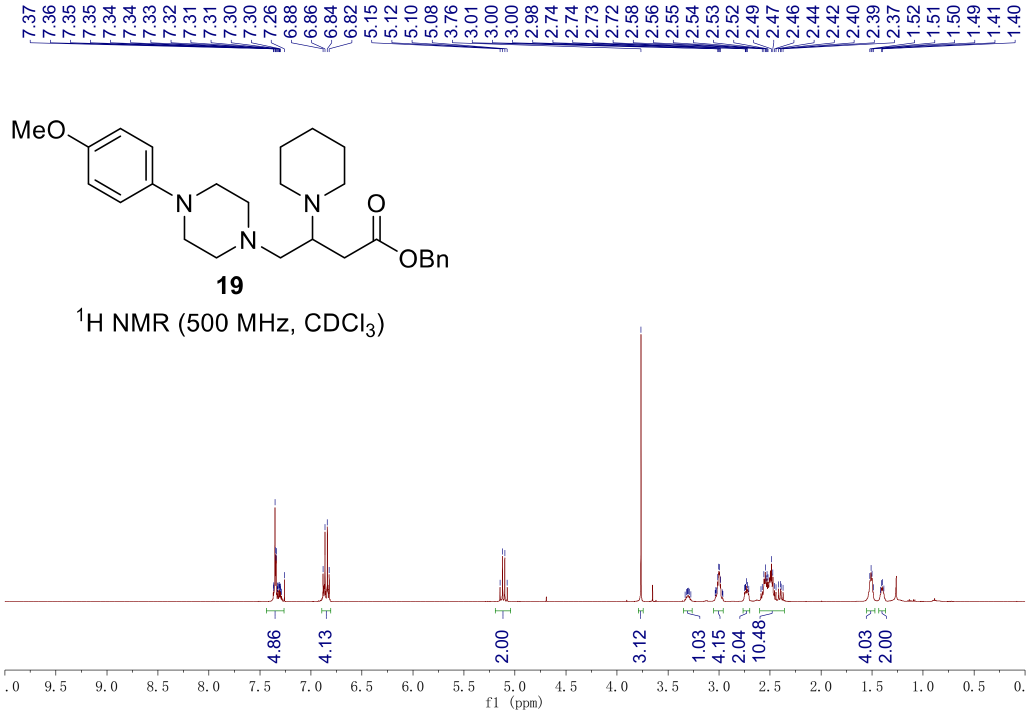

Supplementary Fig. 46. ^{1}H NMR (500 MHz, $CDCl_3$) spectrum of compound **19**.

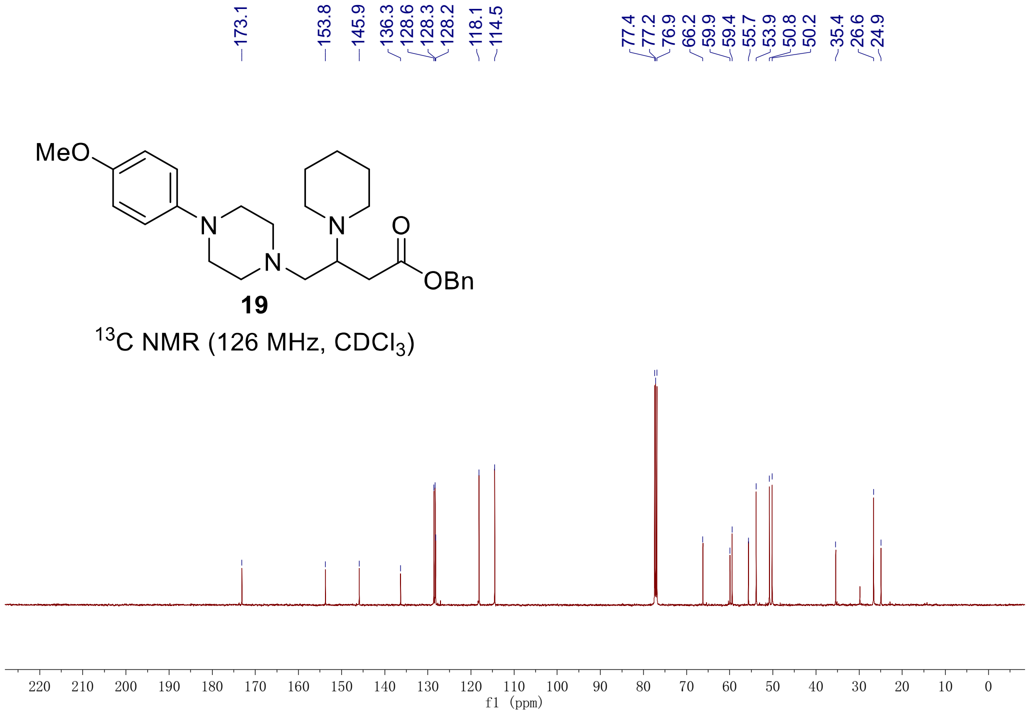

Supplementary Fig. 47. ^{13}C NMR (126 MHz, $CDCl_3$) spectrum of compound **19**.

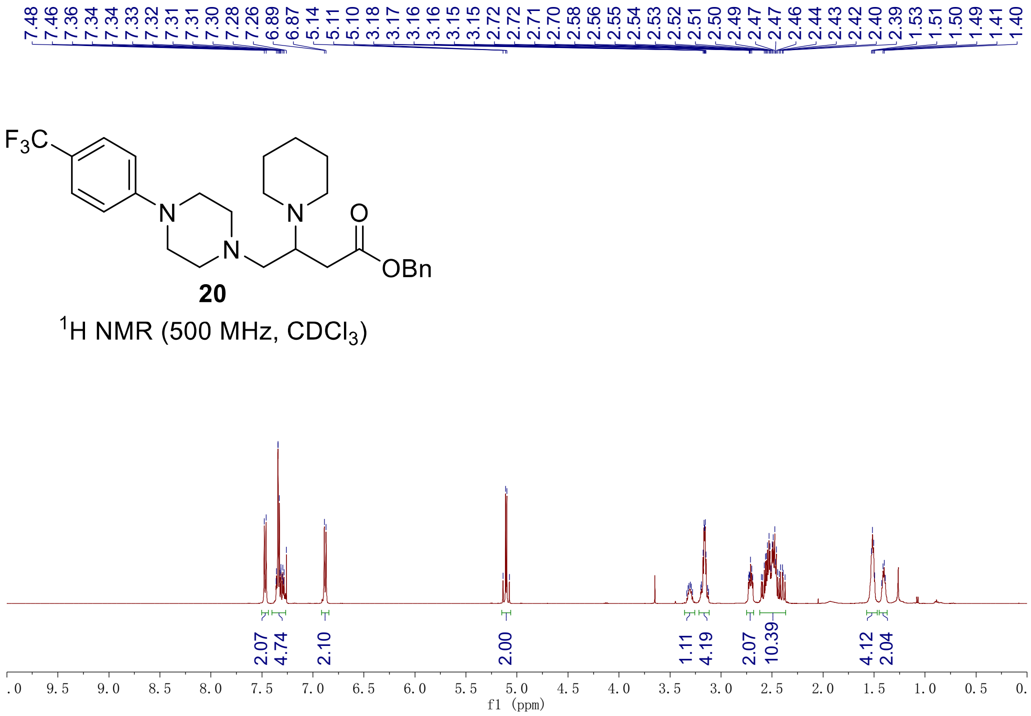

Supplementary Fig. 48. ^{1}H NMR (500 MHz, $CDCl_3$) spectrum of compound **20**.

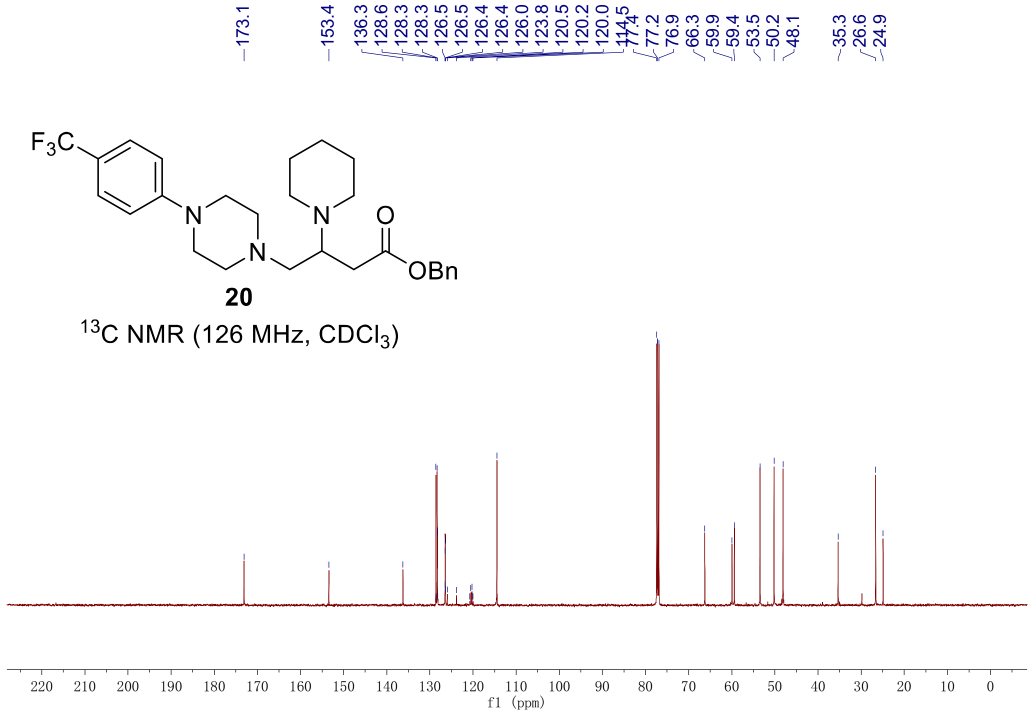

Supplementary Fig. 49. ^{13}C NMR (126 MHz, $CDCl_3$) spectrum of compound **20**.

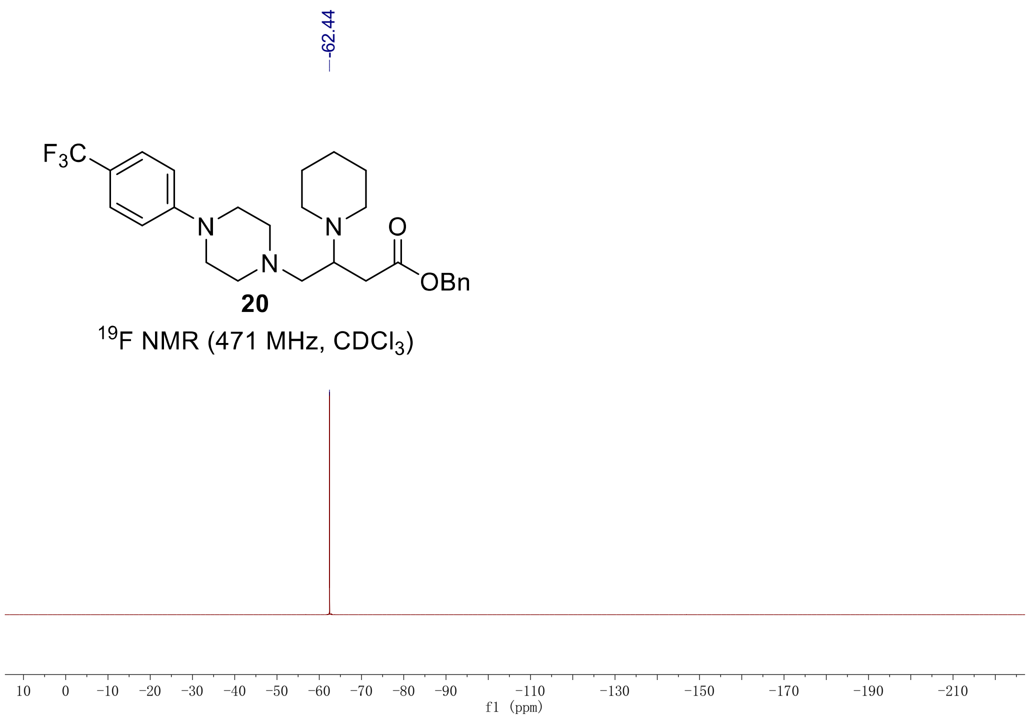

Supplementary Fig. 50. ^{19}F NMR (471 MHz, $CDCl_3$) spectrum of compound **20**.

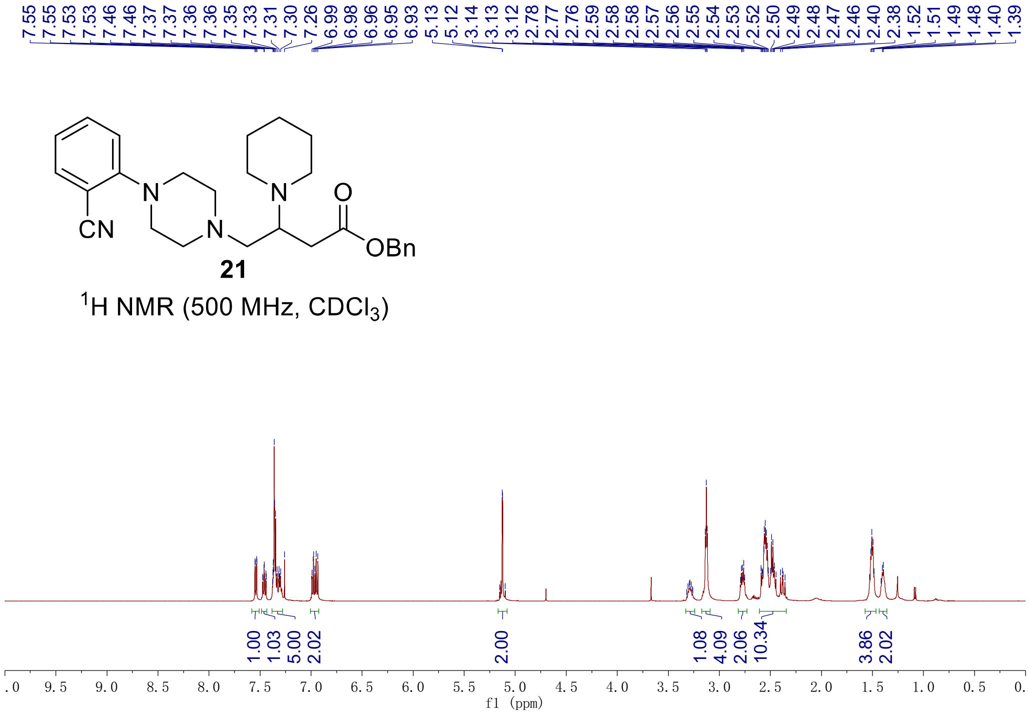

Supplementary Fig. 51. ^{1}H NMR (500 MHz, $CDCl_3$) spectrum of compound **21**.

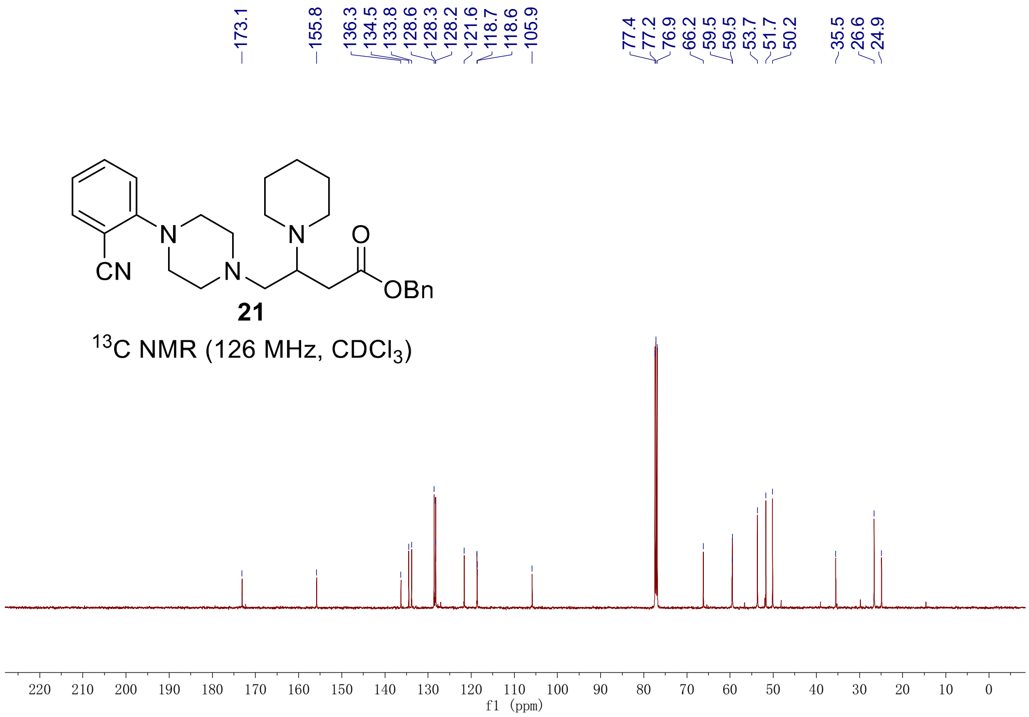

Supplementary Fig. 52. ^{13}C NMR (126 MHz, $CDCl_3$) spectrum of compound **21**.

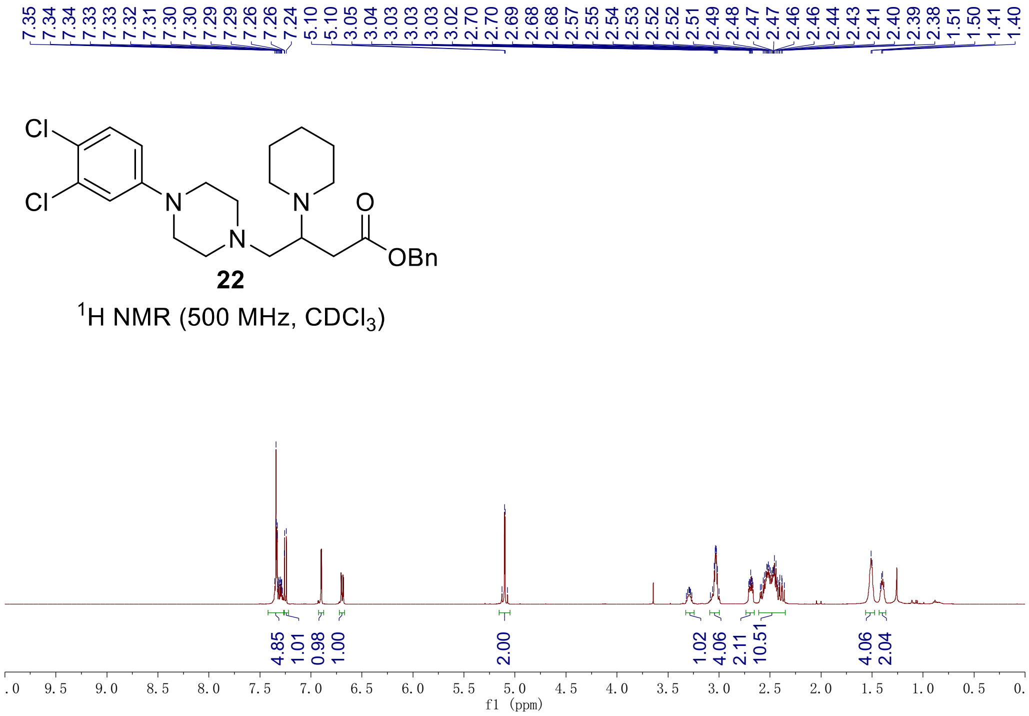

Supplementary Fig. 53. ^{1}H NMR (500 MHz, $CDCl_3$) spectrum of compound **22**.

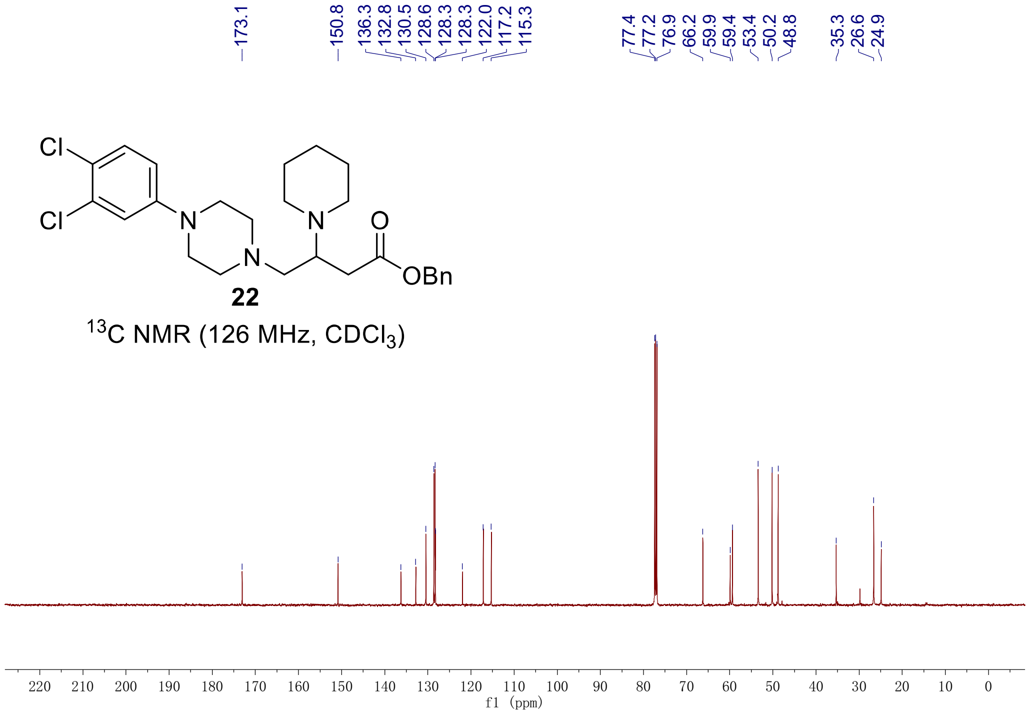

Supplementary Fig. 54. ^{13}C NMR (126 MHz, $CDCl_3$) spectrum of compound **22**.

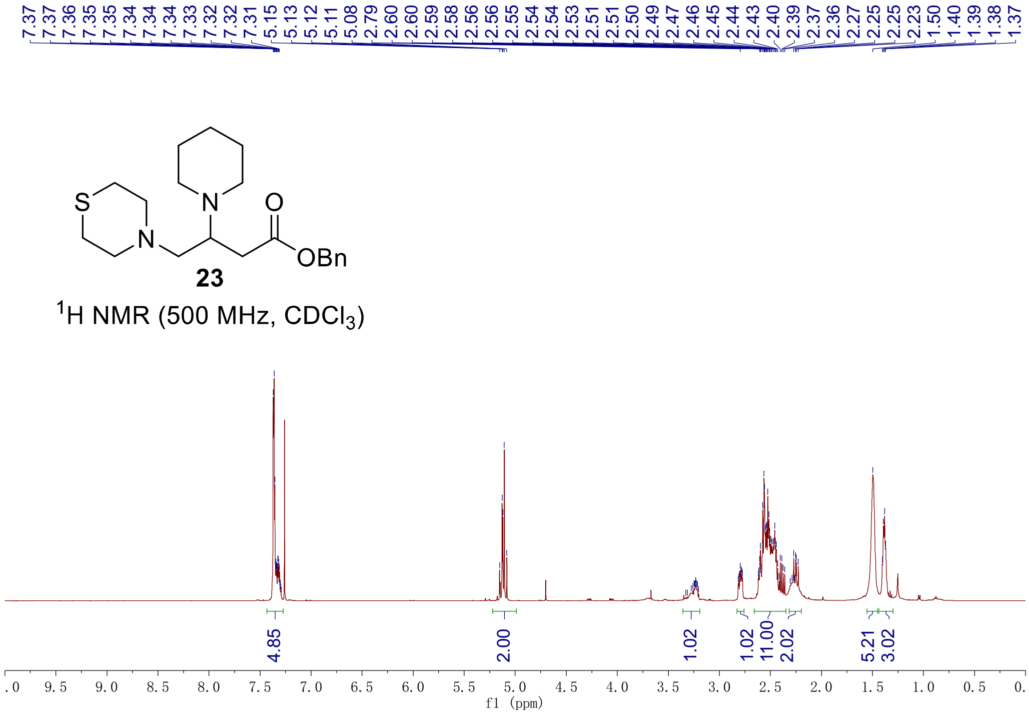

Supplementary Fig. 55. ^{1}H NMR (500 MHz, $CDCl_3$) spectrum of compound **23**.

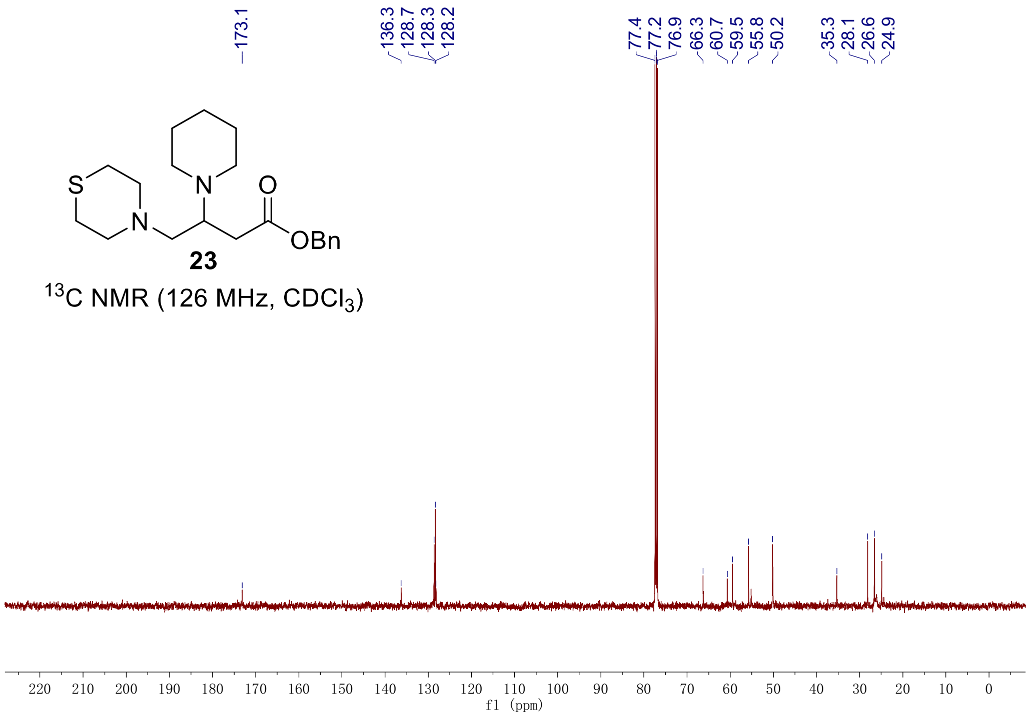

Supplementary Fig. 56. ^{13}C NMR (126 MHz, $CDCl_3$) spectrum of compound **23**.

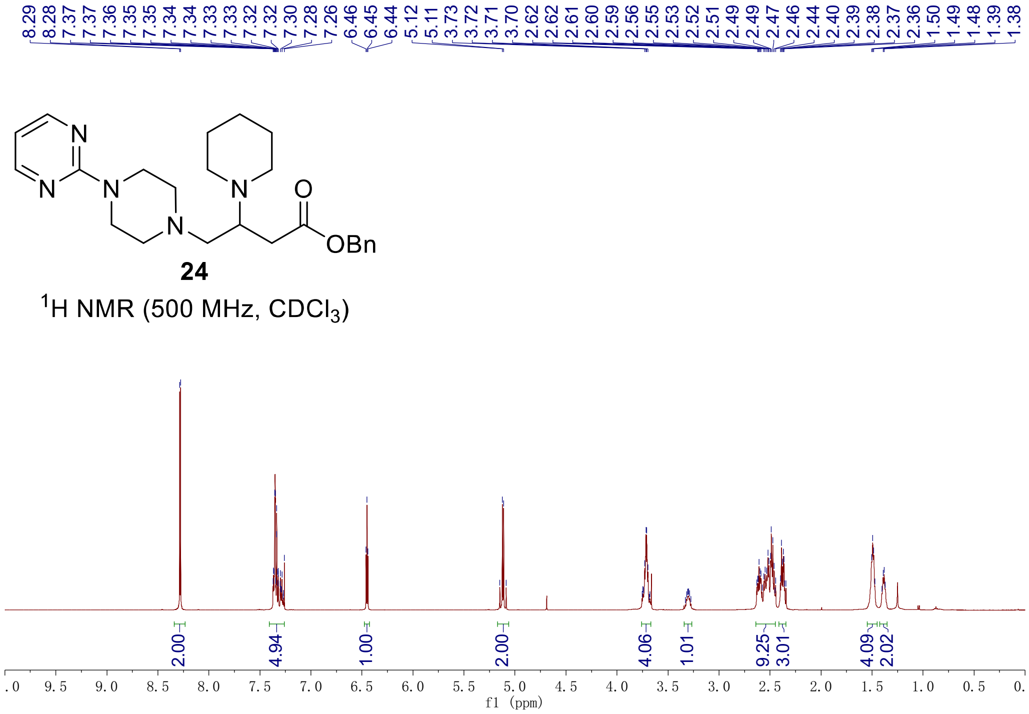

Supplementary Fig. 57. ^{1}H NMR (500 MHz, $CDCl_3$) spectrum of compound **24**.

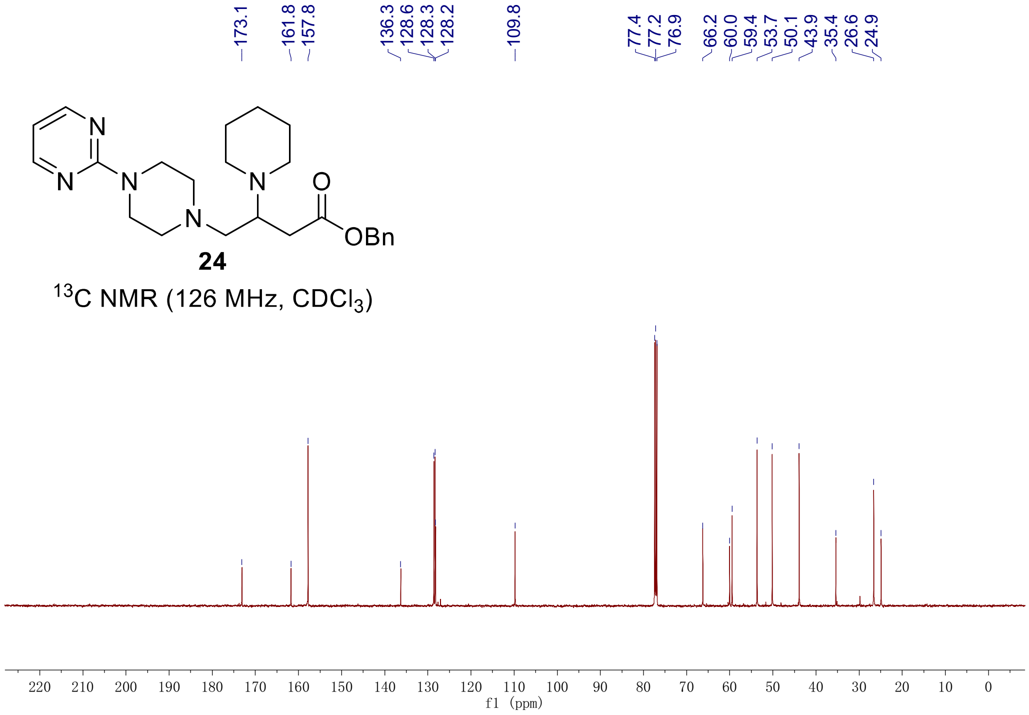

Supplementary Fig. 58. ^{13}C NMR (126 MHz, $CDCl_3$) spectrum of compound **24**.

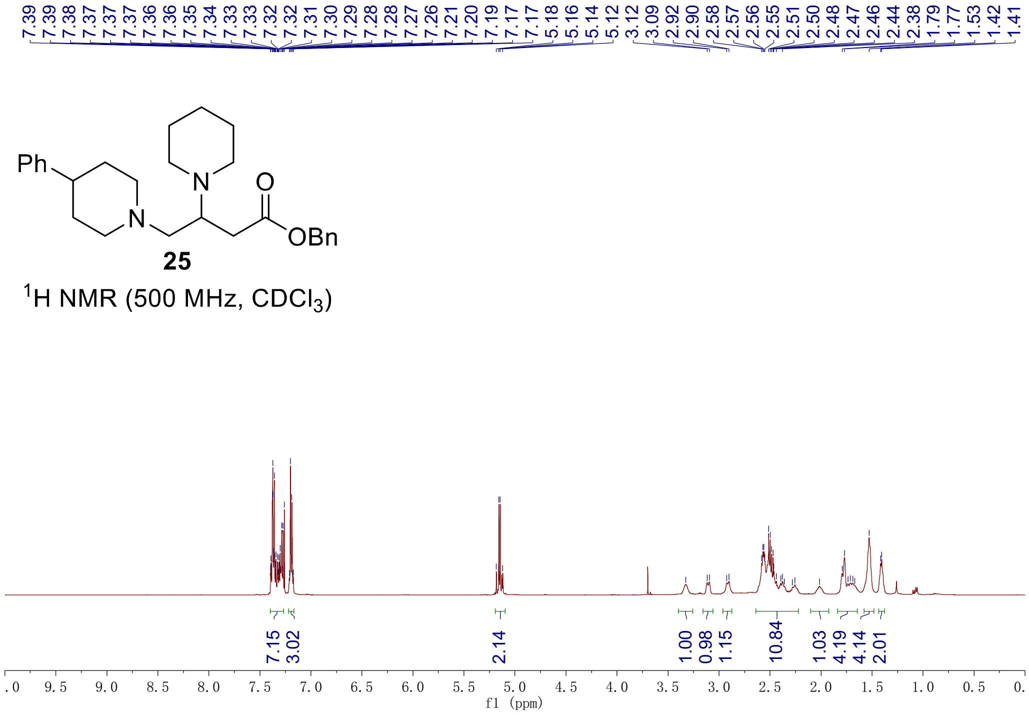

Supplementary Fig. 59. ^{1}H NMR (500 MHz, CDCl$_3$) spectrum of compound **25**.

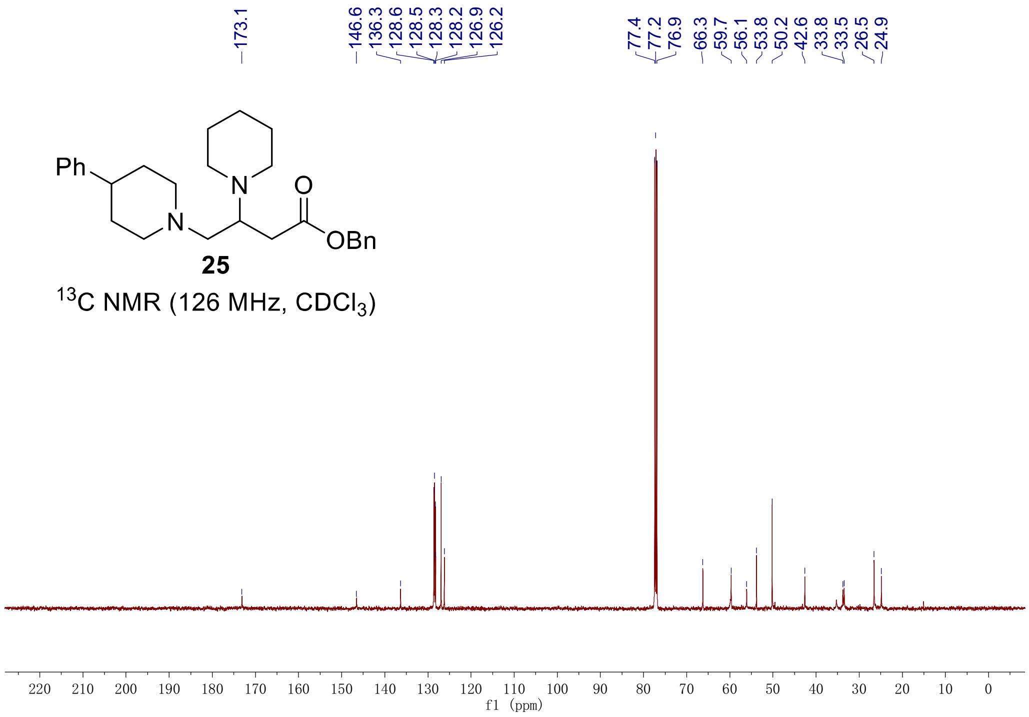

Supplementary Fig. 60. ^{13}C NMR (126 MHz, CDCl$_3$) spectrum of compound **25**.

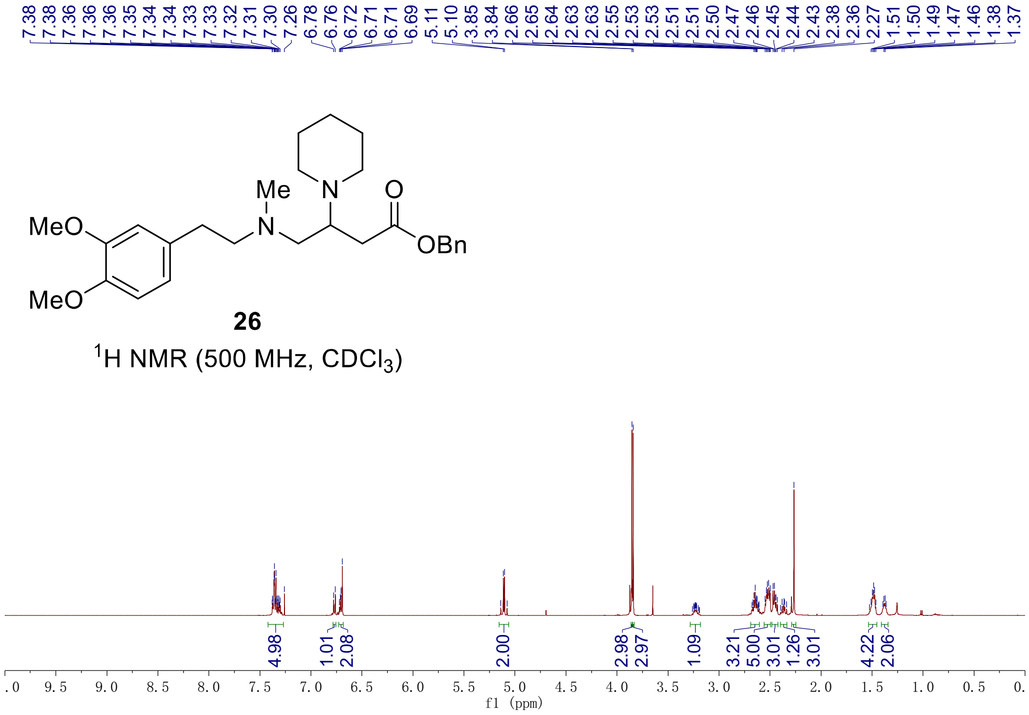

Supplementary Fig. 61. ^{1}H NMR (500 MHz, CDCl$_3$) spectrum of compound **26**.

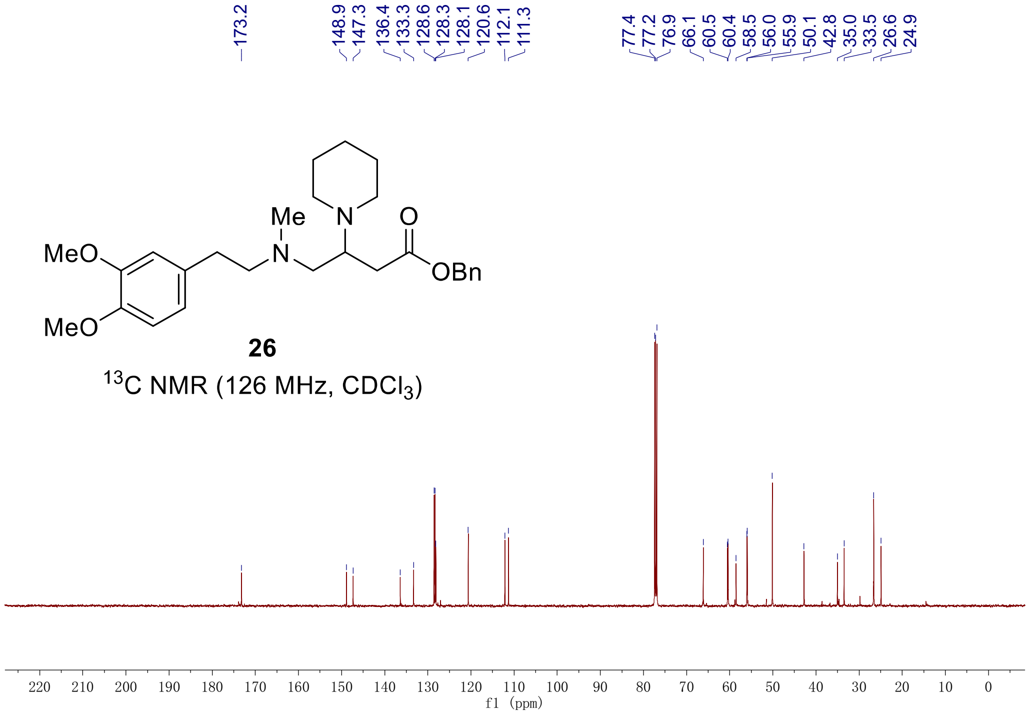

Supplementary Fig. 62. ^{13}C NMR (126 MHz, CDCl$_3$) spectrum of compound **26**.

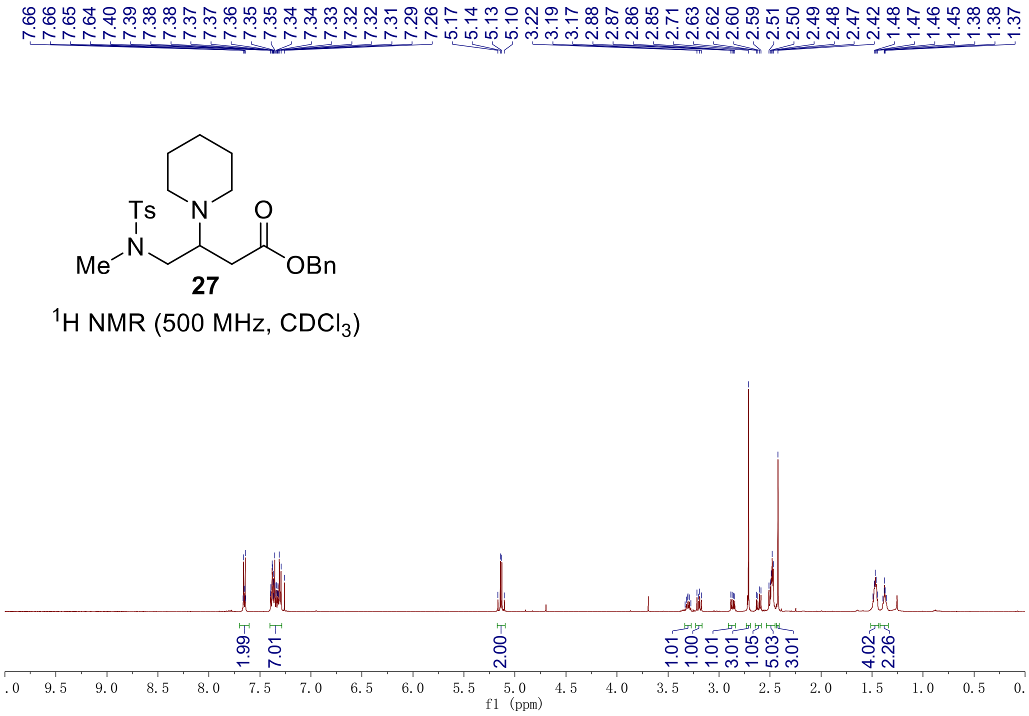

Supplementary Fig. 63. ^{1}H NMR (500 MHz, $CDCl_3$) spectrum of compound **27**.

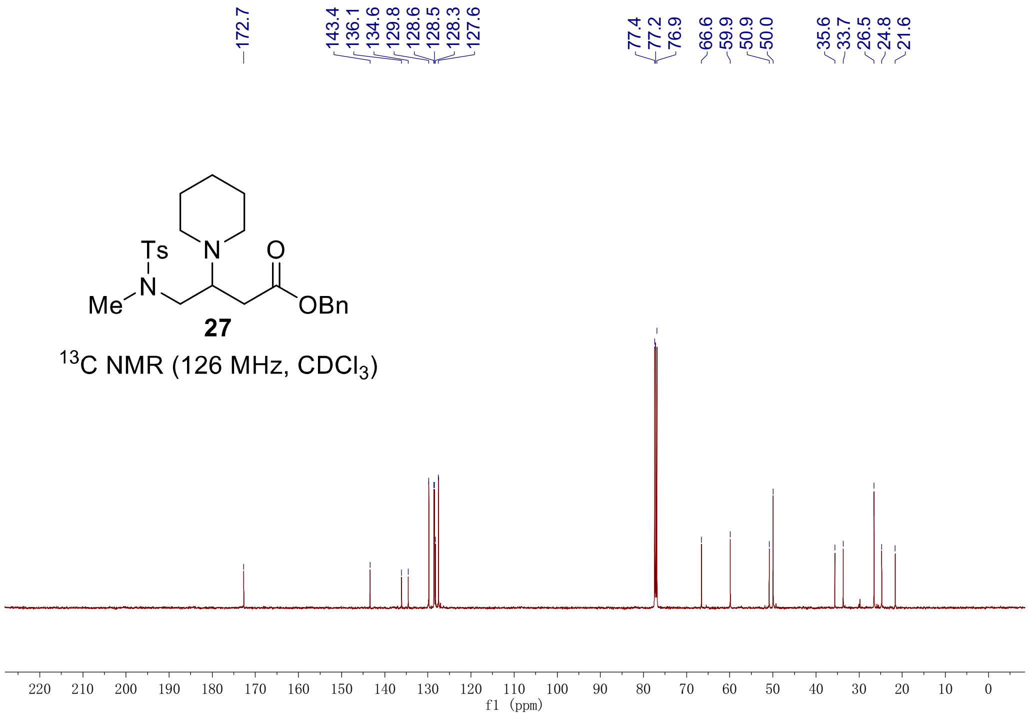

Supplementary Fig. 64. ^{13}C NMR (126 MHz, $CDCl_3$) spectrum of compound **27**.

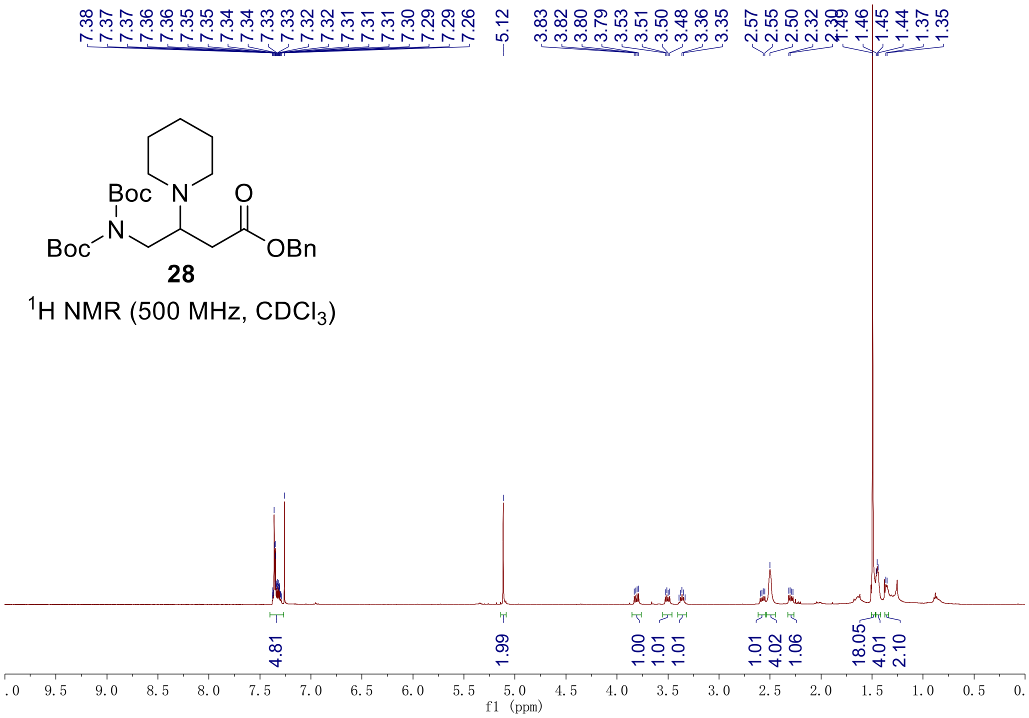

Supplementary Fig. 65. ^{1}H NMR (500 MHz, $CDCl_3$) spectrum of compound **28**.

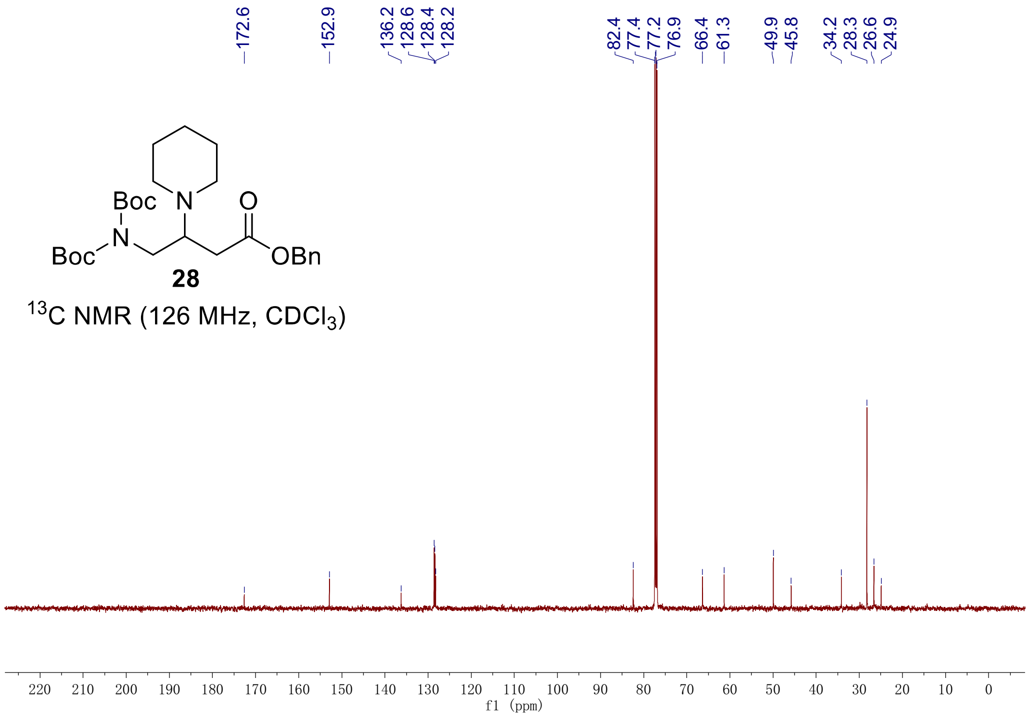

Supplementary Fig. 66. ^{13}C NMR (126 MHz, $CDCl_3$) spectrum of compound **28**.

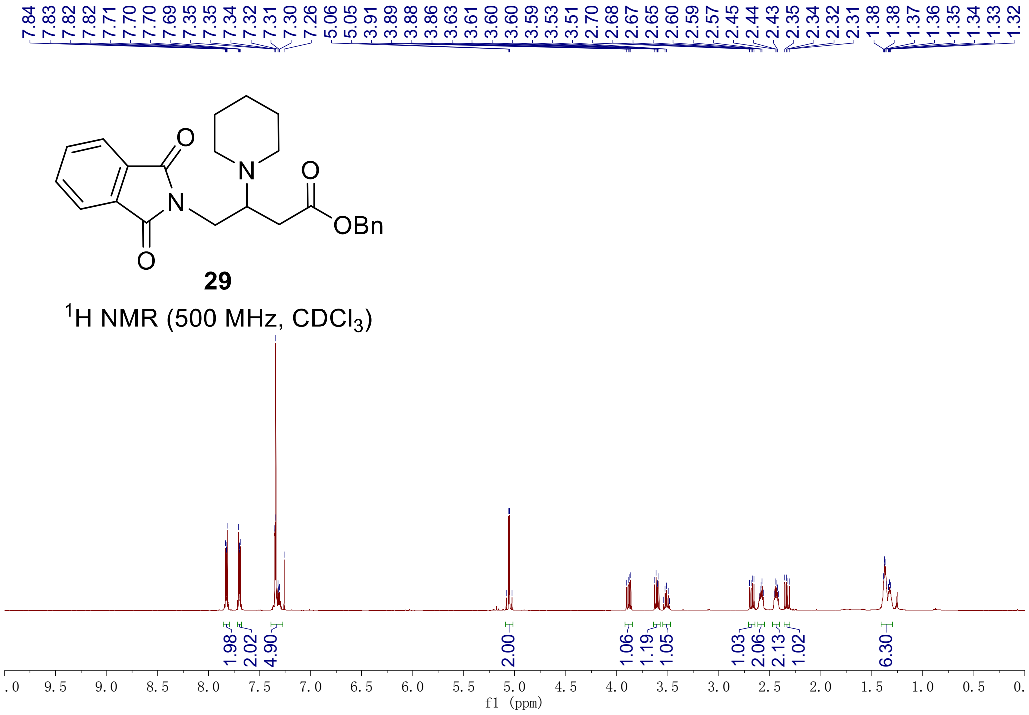

Supplementary Fig. 67. ^{1}H NMR (500 MHz, CDCl$_3$) spectrum of compound **29**.

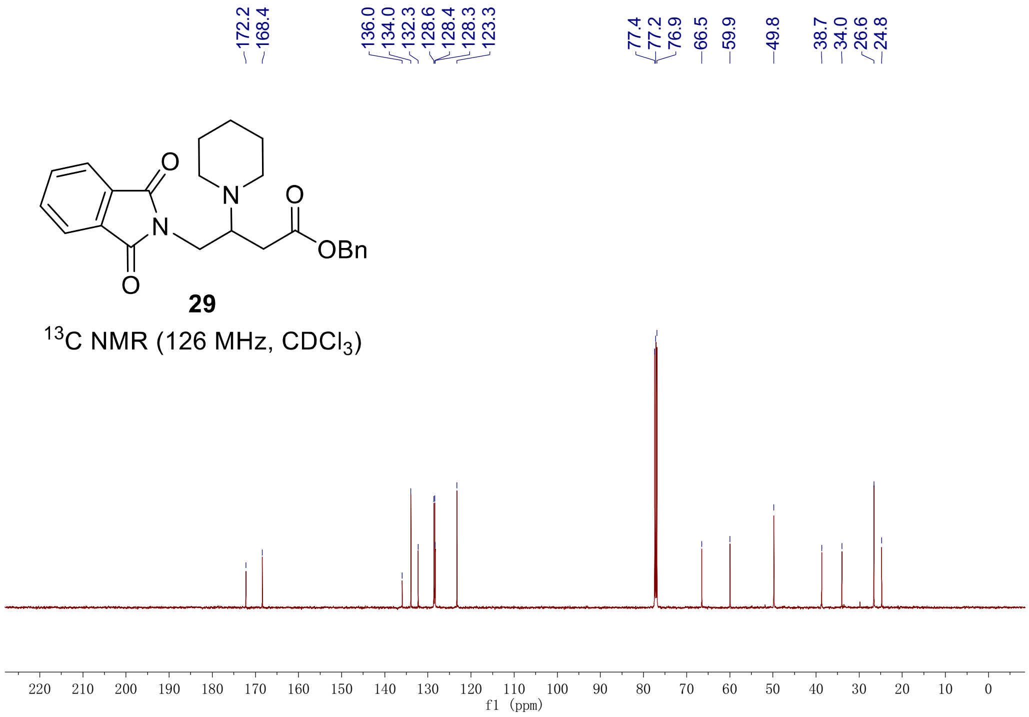

Supplementary Fig. 68. ^{13}C NMR (126 MHz, CDCl$_3$) spectrum of compound **29**.

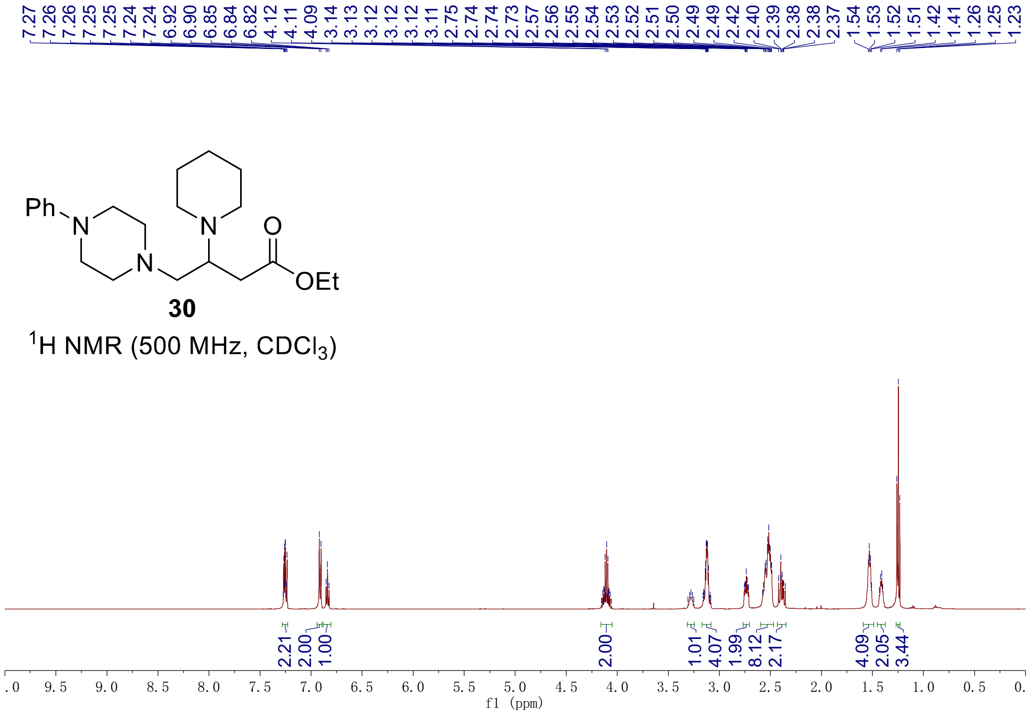

Supplementary Fig. 69. ^{1}H NMR (500 MHz, $CDCl_3$) spectrum of compound **30**.

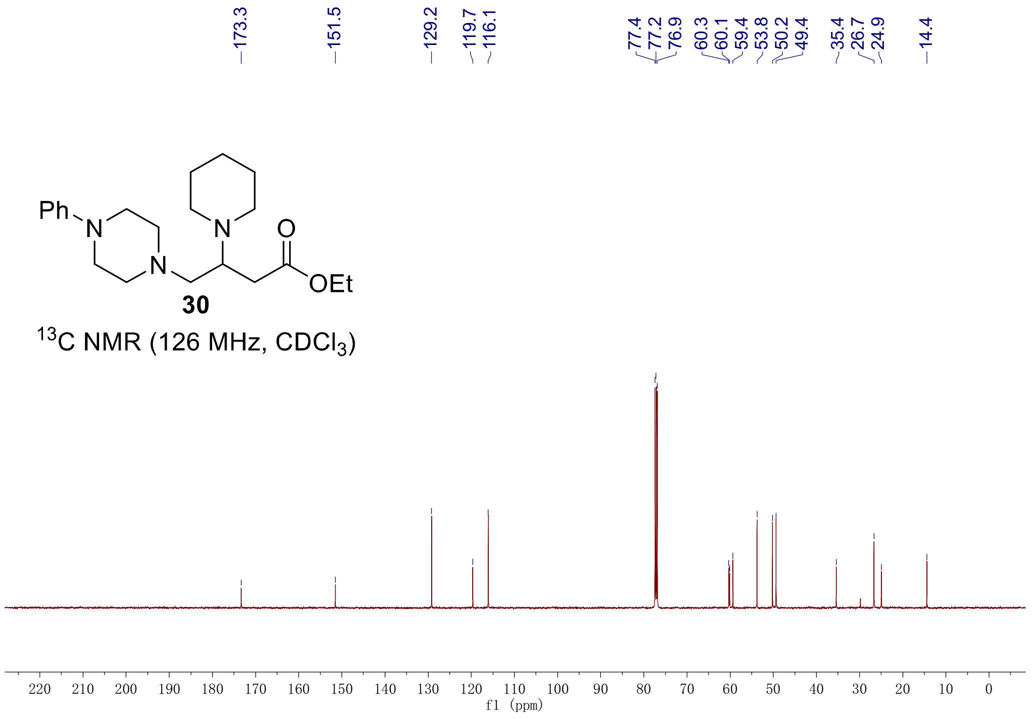

Supplementary Fig. 70. ^{13}C NMR (126 MHz, $CDCl_3$) spectrum of compound **30**.

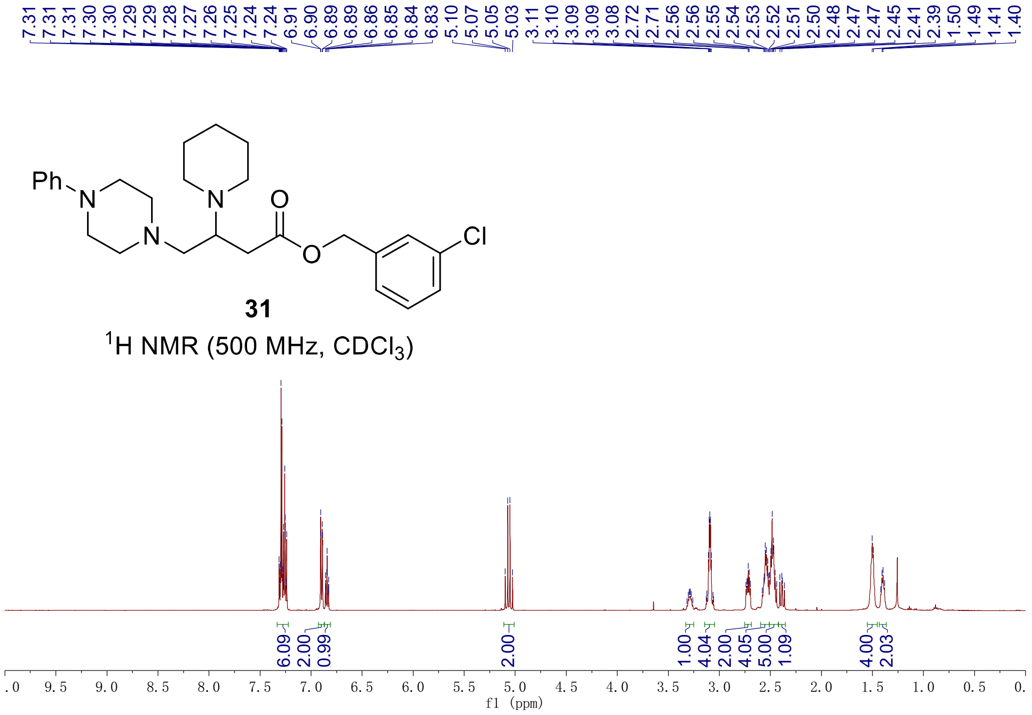

Supplementary Fig. 71. ^{1}H NMR (500 MHz, $CDCl_3$) spectrum of compound **31**.

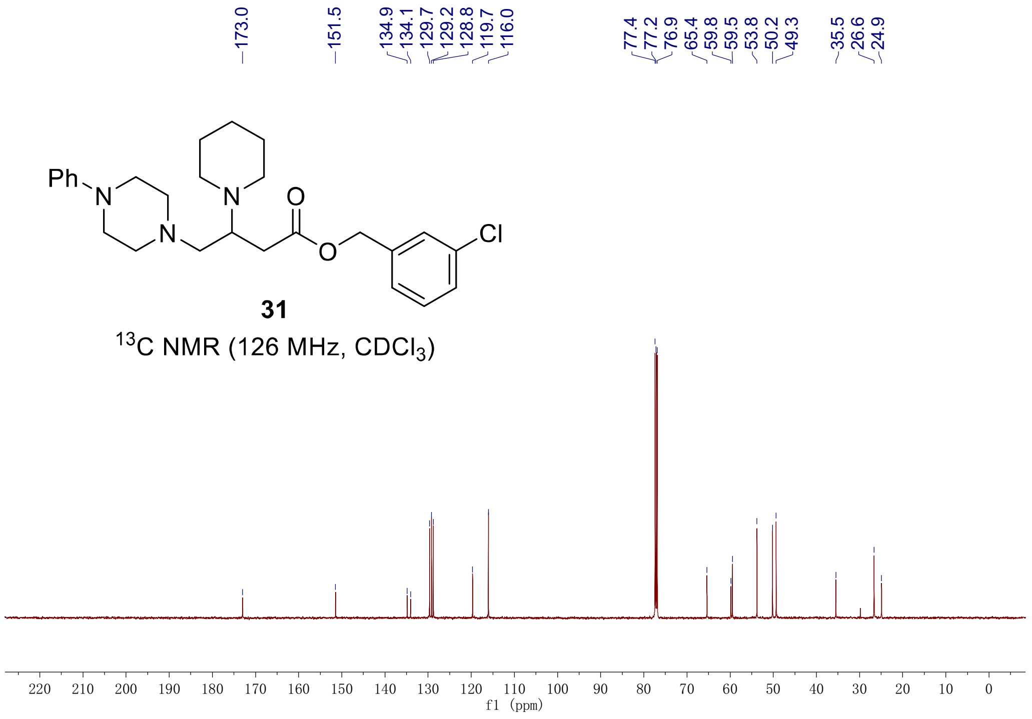

Supplementary Fig. 72. ^{13}C NMR (126 MHz, $CDCl_3$) spectrum of compound **31**.

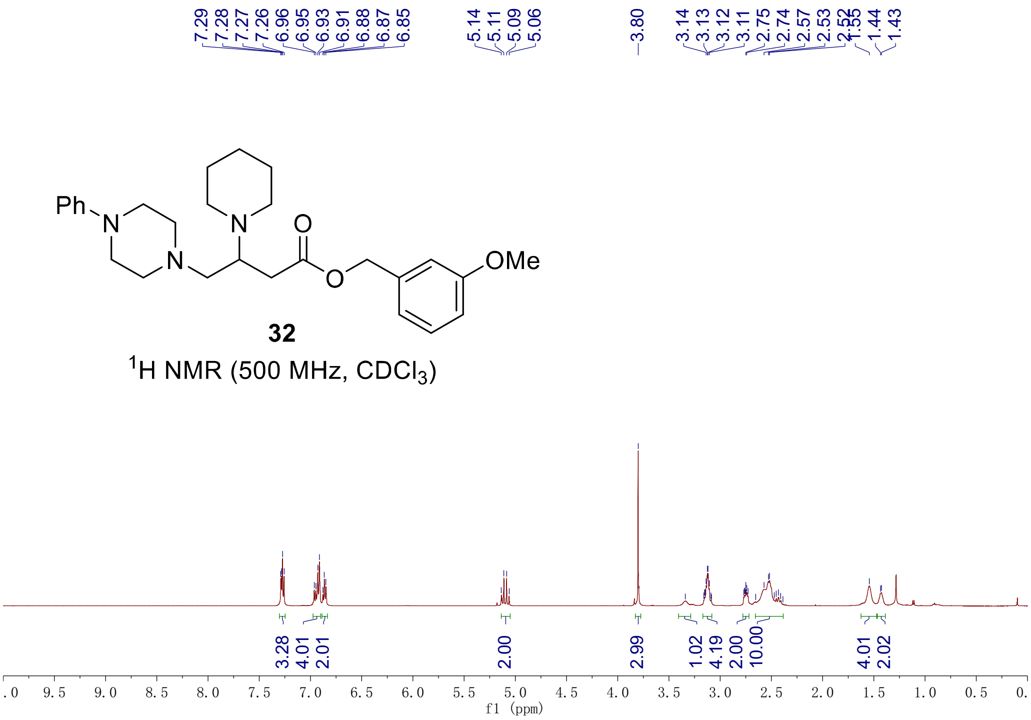

Supplementary Fig. 73. ^{1}H NMR (500 MHz, $CDCl_3$) spectrum of compound **32**.

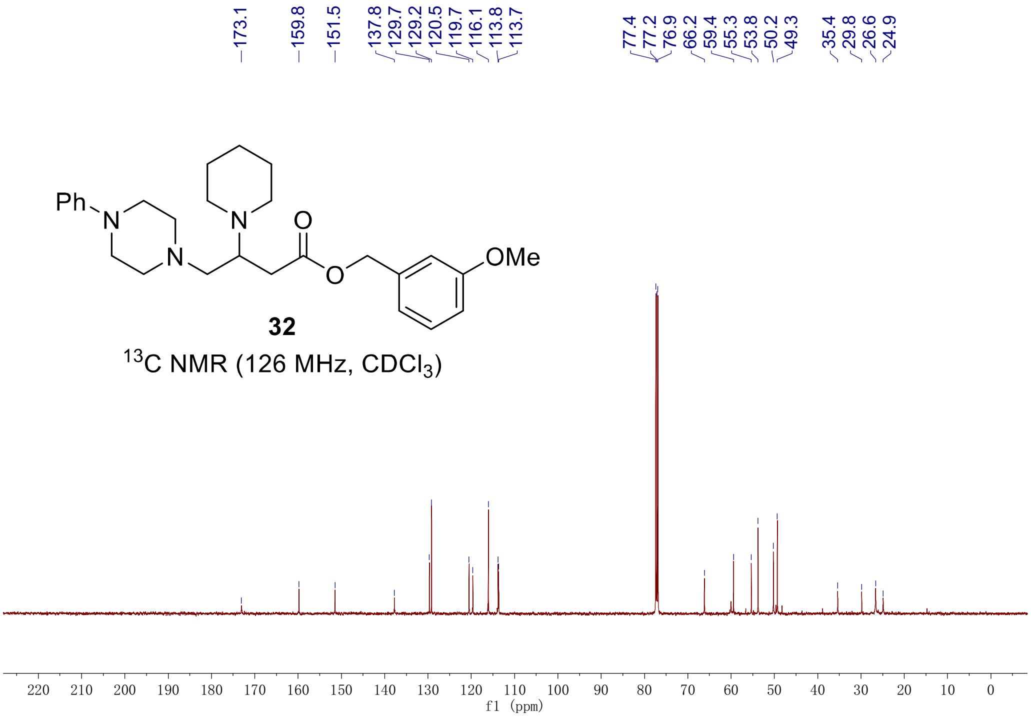

Supplementary Fig. 74. ^{13}C NMR (126 MHz, $CDCl_3$) spectrum of compound **32**.

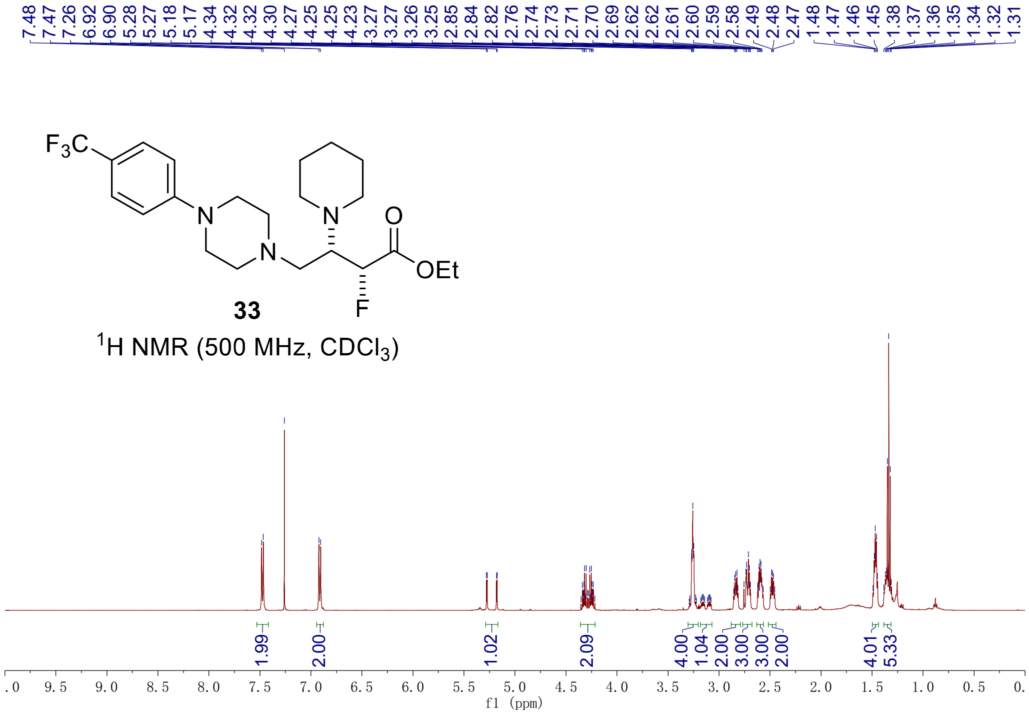

Supplementary Fig. 75. ^{1}H NMR (500 MHz, $CDCl_3$) spectrum of compound **33**.

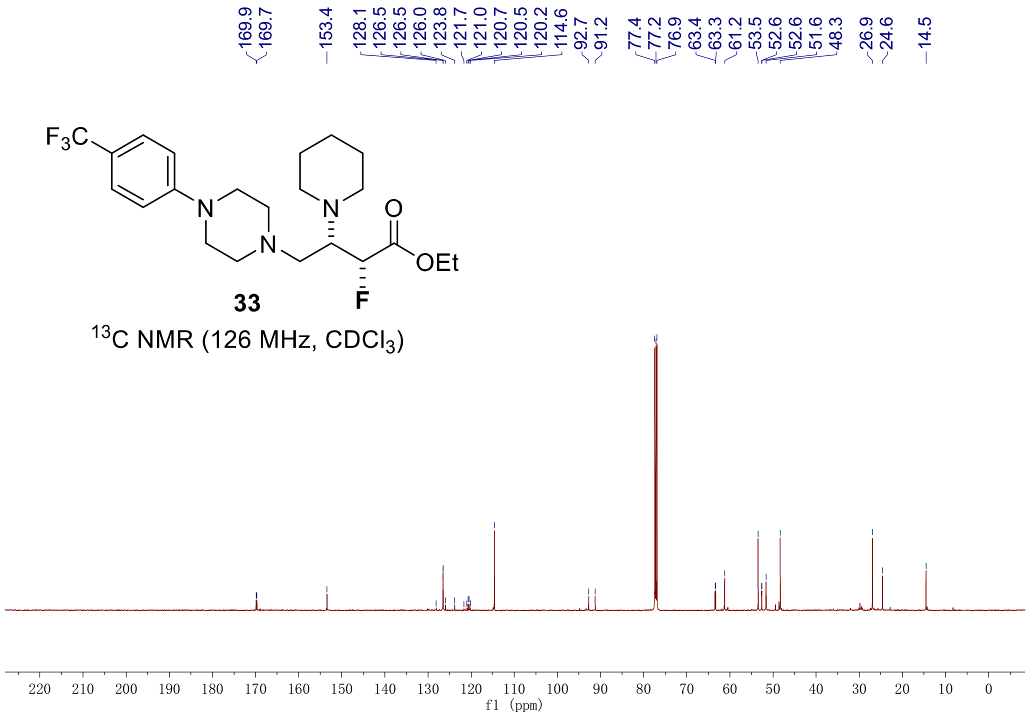

Supplementary Fig. 76. ^{13}C NMR (126 MHz, $CDCl_3$) spectrum of compound **33**.

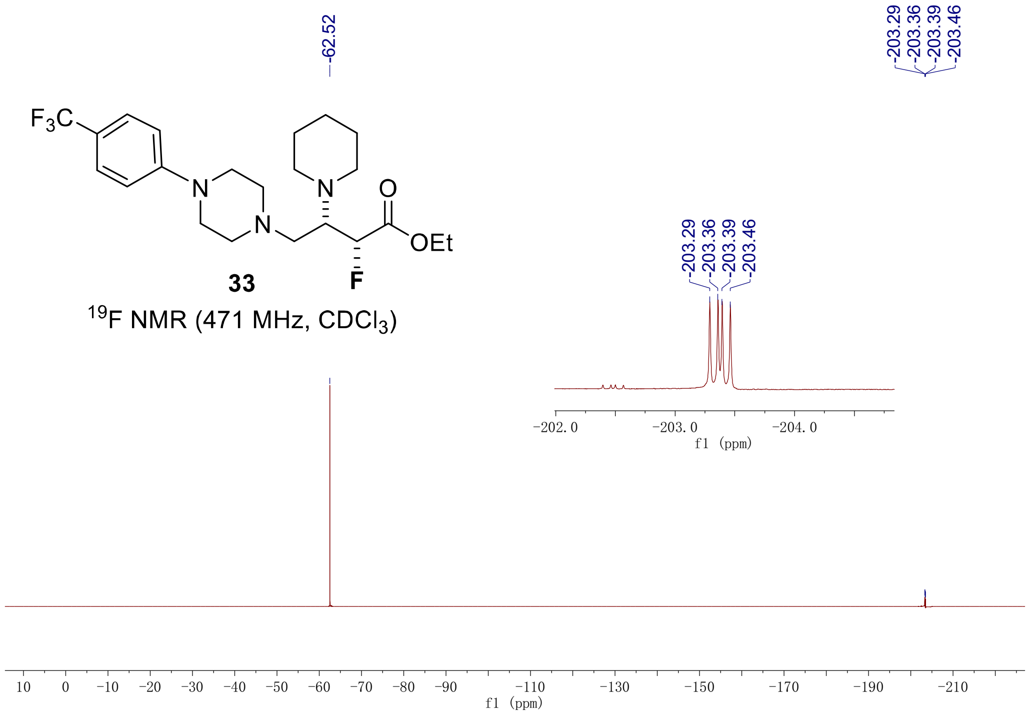

Supplementary Fig. 77. ^{19}F NMR (471 MHz, $CDCl_3$) spectrum of compound **33**.

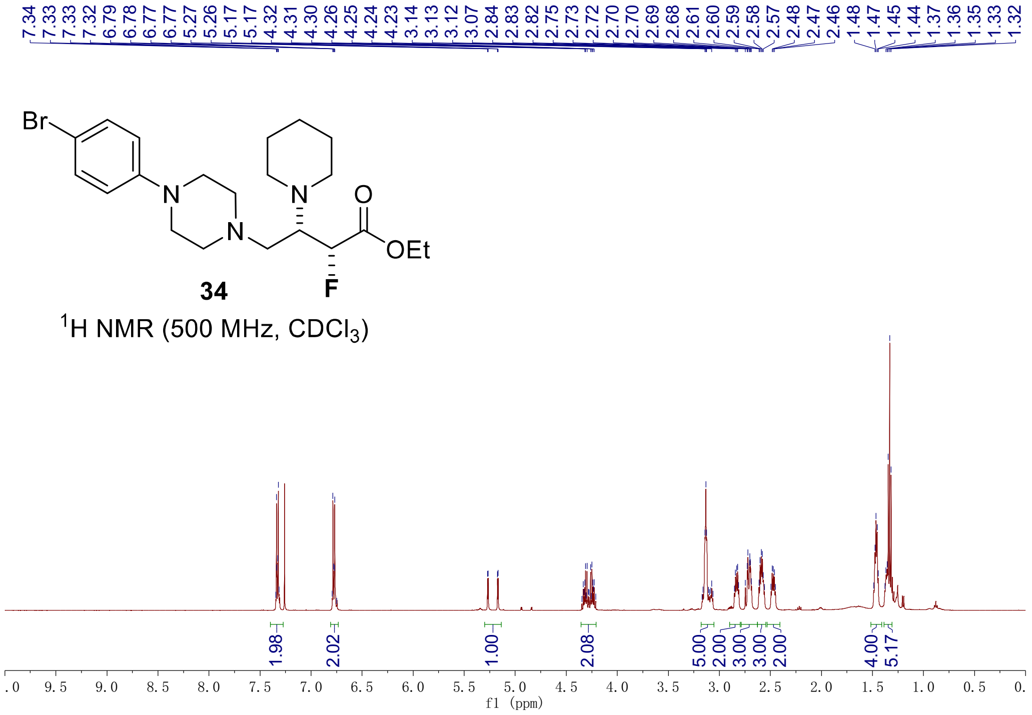

Supplementary Fig. 78. ^{1}H NMR (500 MHz, $CDCl_3$) spectrum of compound **34**.

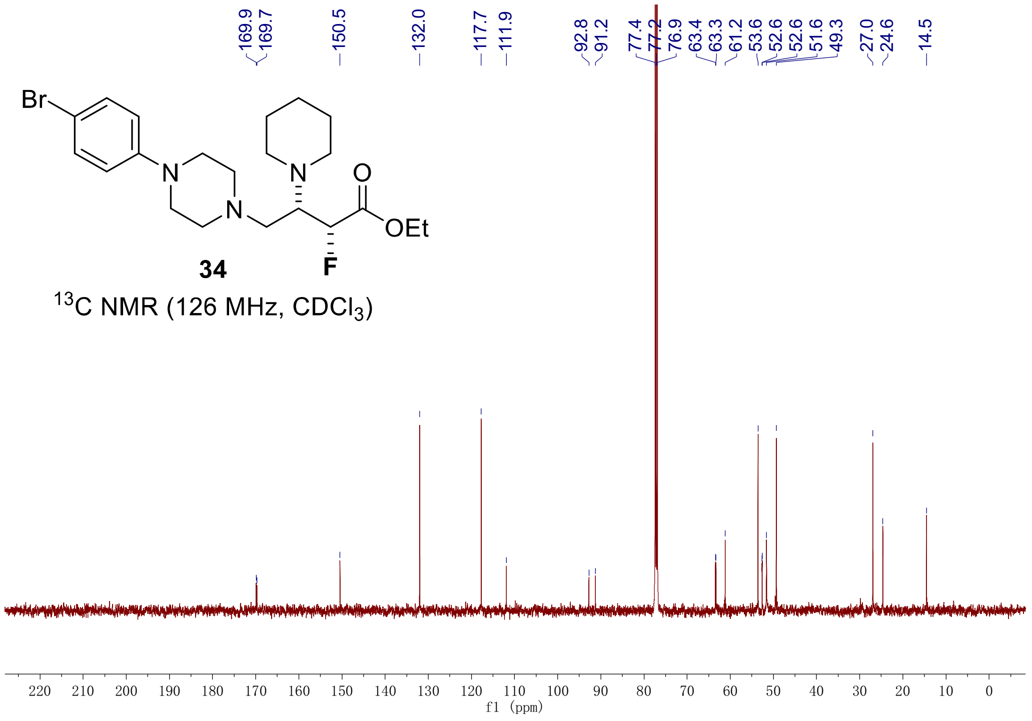

Supplementary Fig. 79. ^{13}C NMR (126 MHz, $CDCl_3$) spectrum of compound **34**.

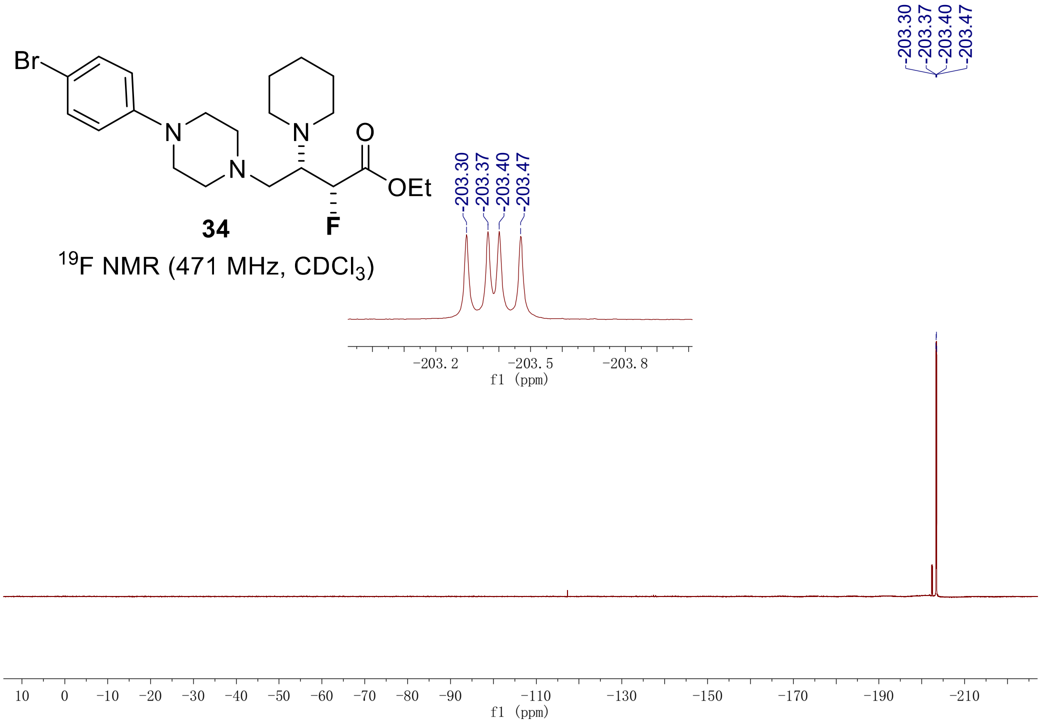

Supplementary Fig. 80. ^{19}F NMR (471 MHz, $CDCl_3$) spectrum of compound **34**.

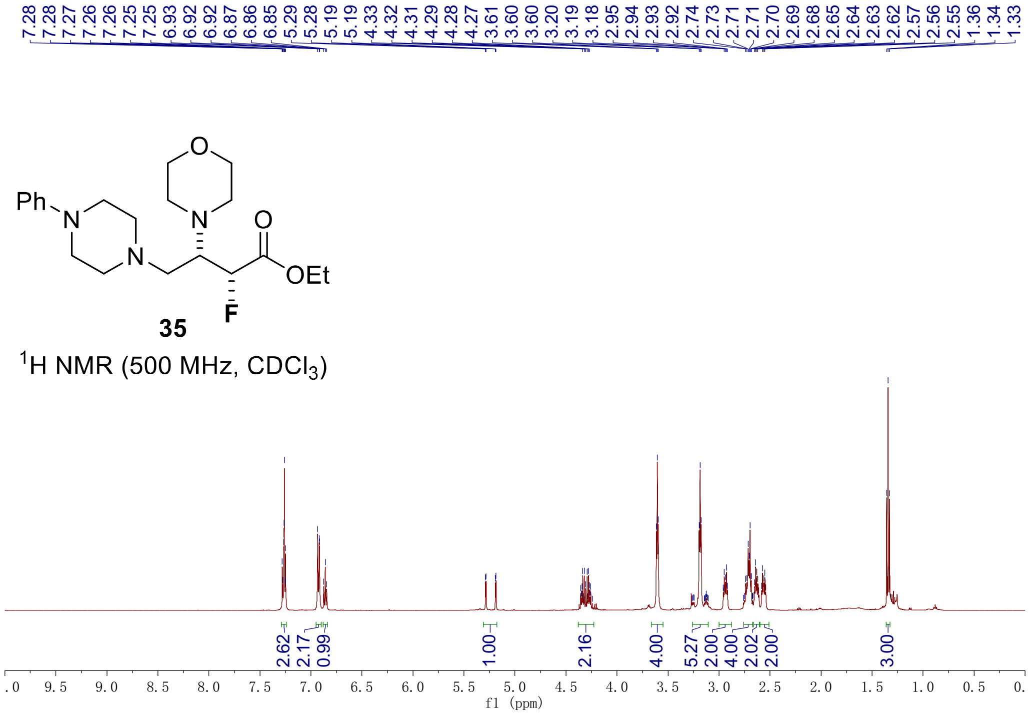

Supplementary Fig. 81. ^{1}H NMR (500 MHz, $CDCl_3$) spectrum of compound **35**.

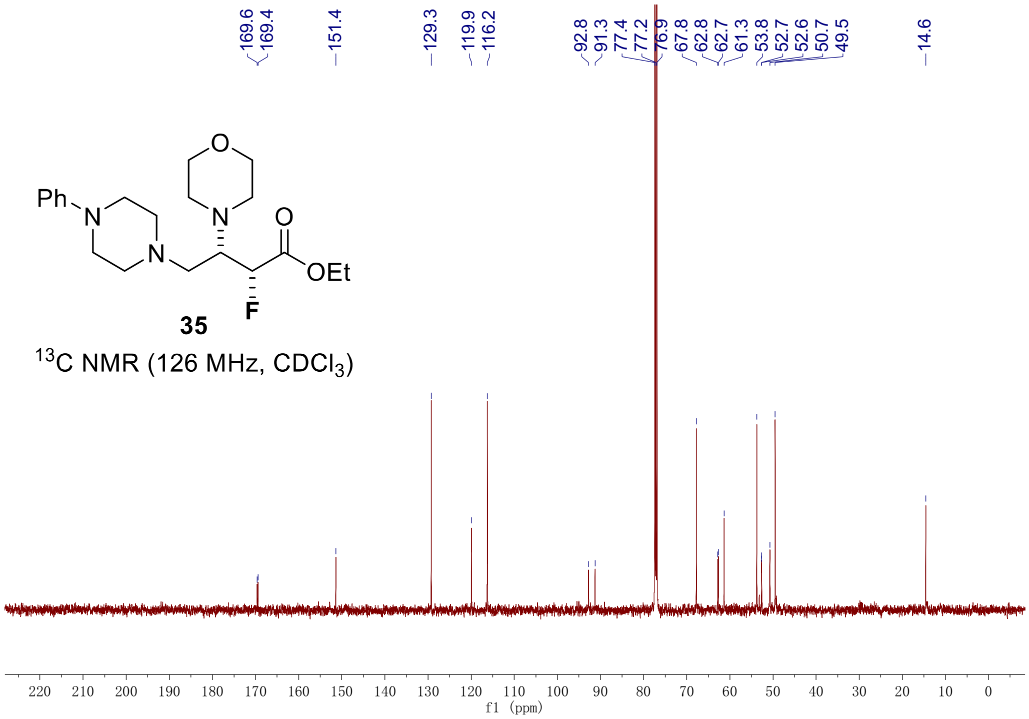

Supplementary Fig. 82. ^{13}C NMR (126 MHz, $CDCl_3$) spectrum of compound **35**.

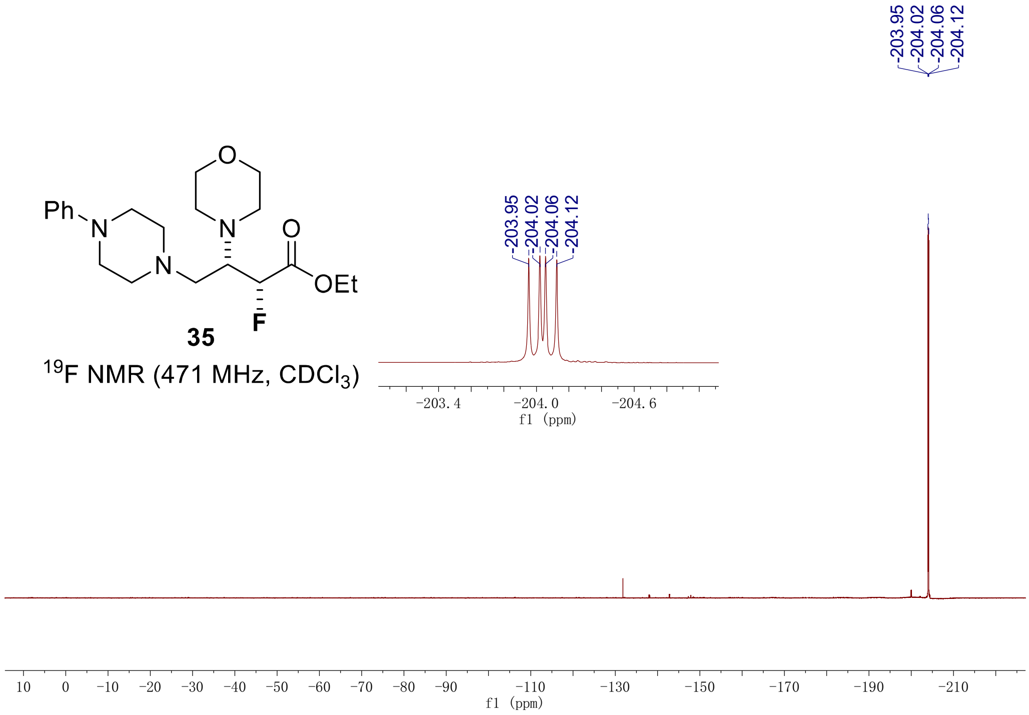

Supplementary Fig. 83. ^{19}F NMR (471 MHz, $CDCl_3$) spectrum of compound **35**.

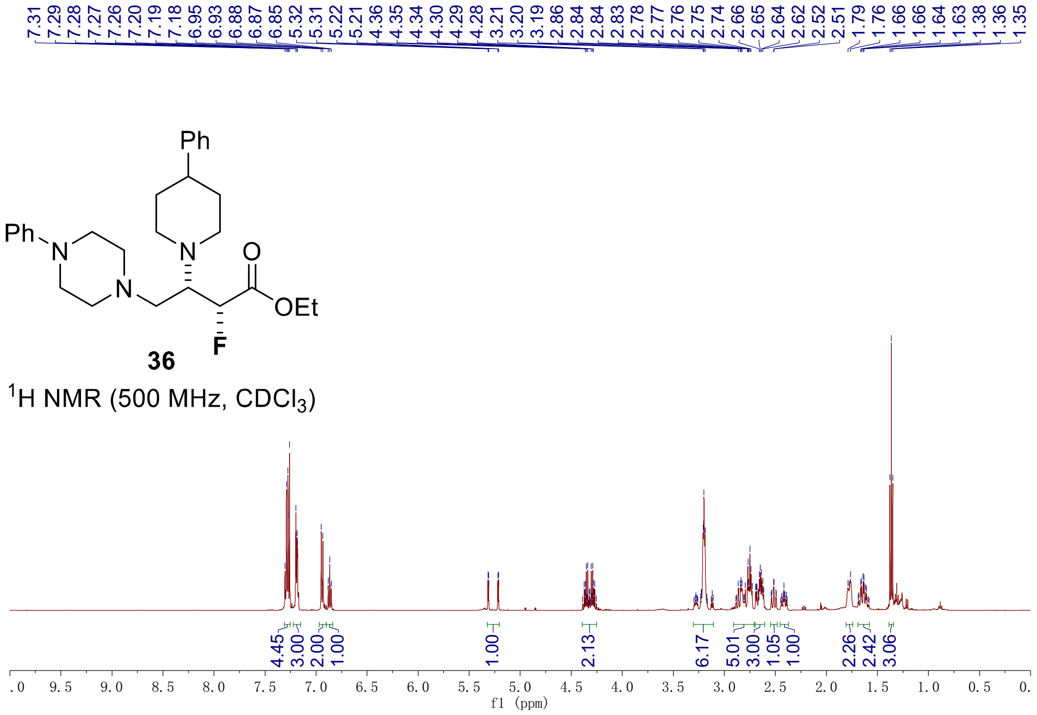

Supplementary Fig. 84. ^{1}H NMR (500 MHz, $CDCl_3$) spectrum of compound **36**.

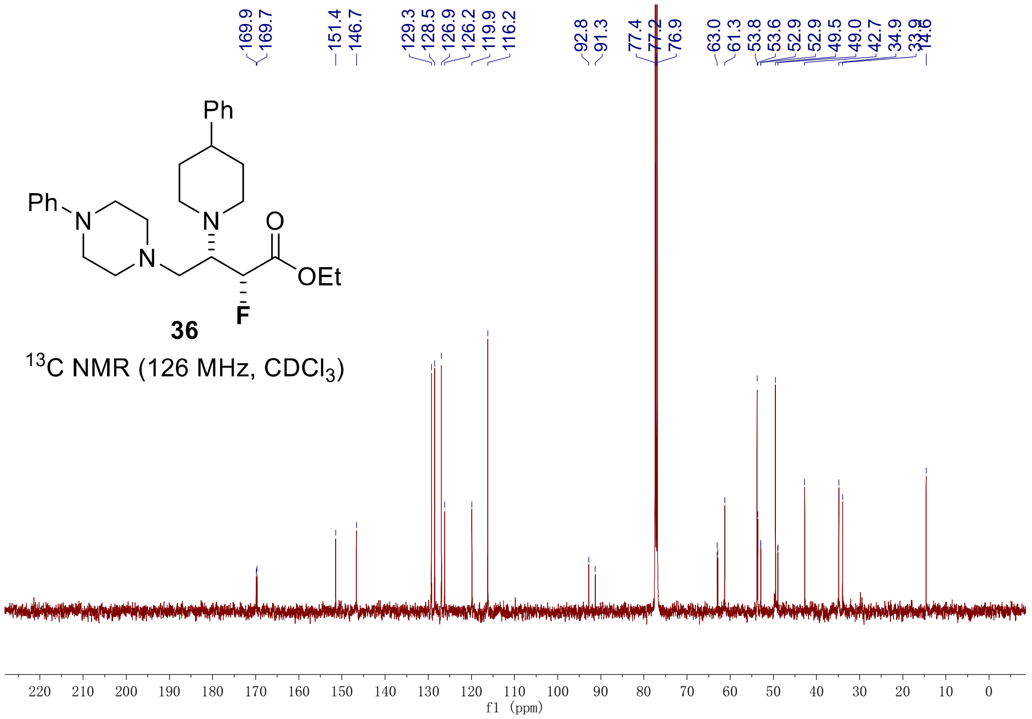

Supplementary Fig. 85. ^{13}C NMR (126 MHz, $CDCl_3$) spectrum of compound **36**.

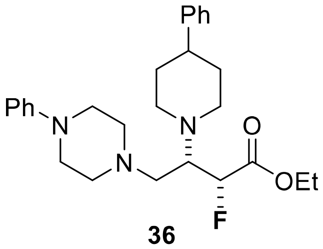

36

^{19}F NMR (471 MHz, $CDCl_3$)

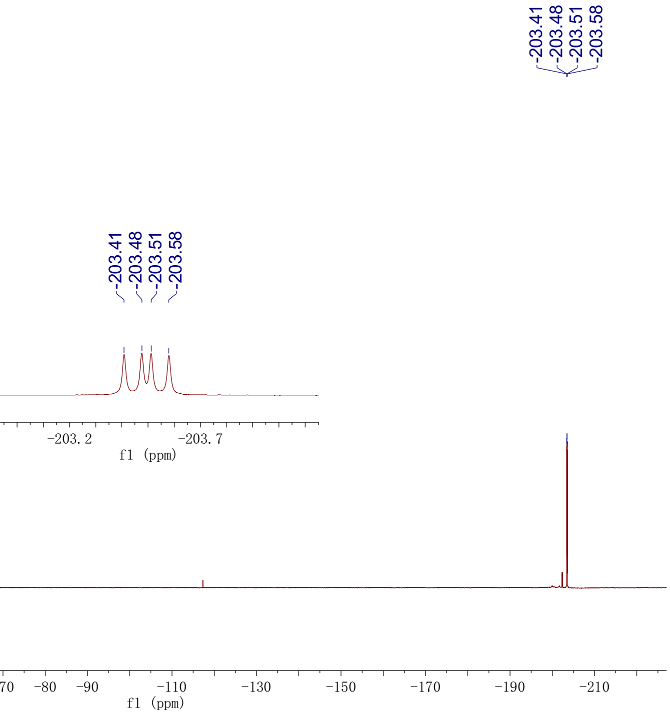

Supplementary Fig. 86. ^{19}F NMR (471 MHz, $CDCl_3$) spectrum of compound **36**.

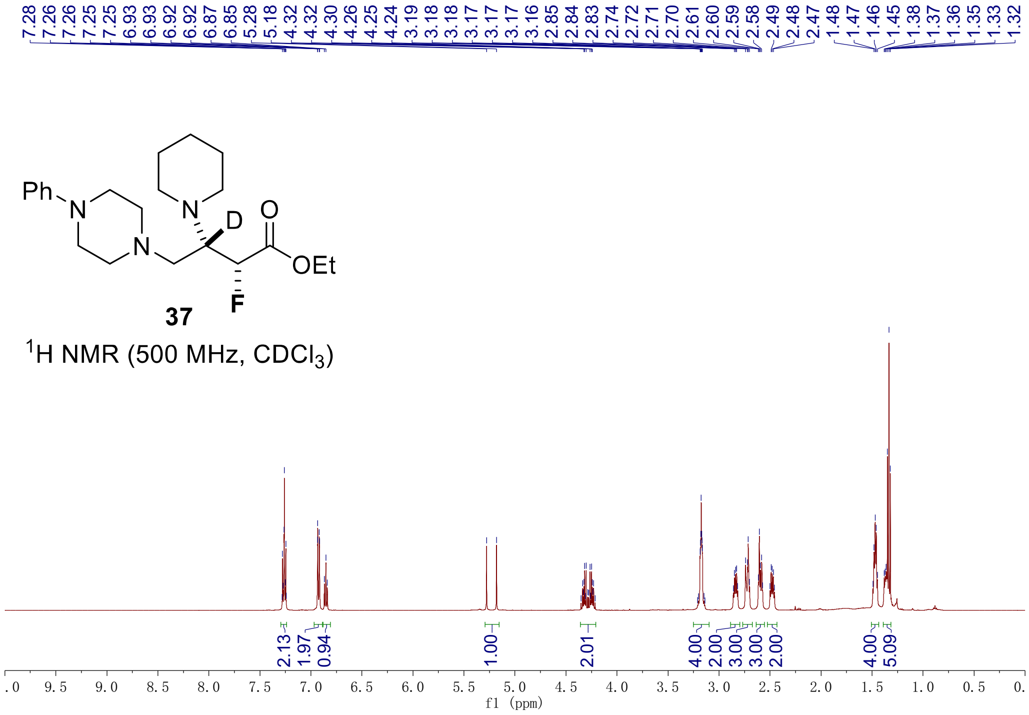

Supplementary Fig. 87. ^{1}H NMR (500 MHz, $CDCl_3$) spectrum of compound **37**.

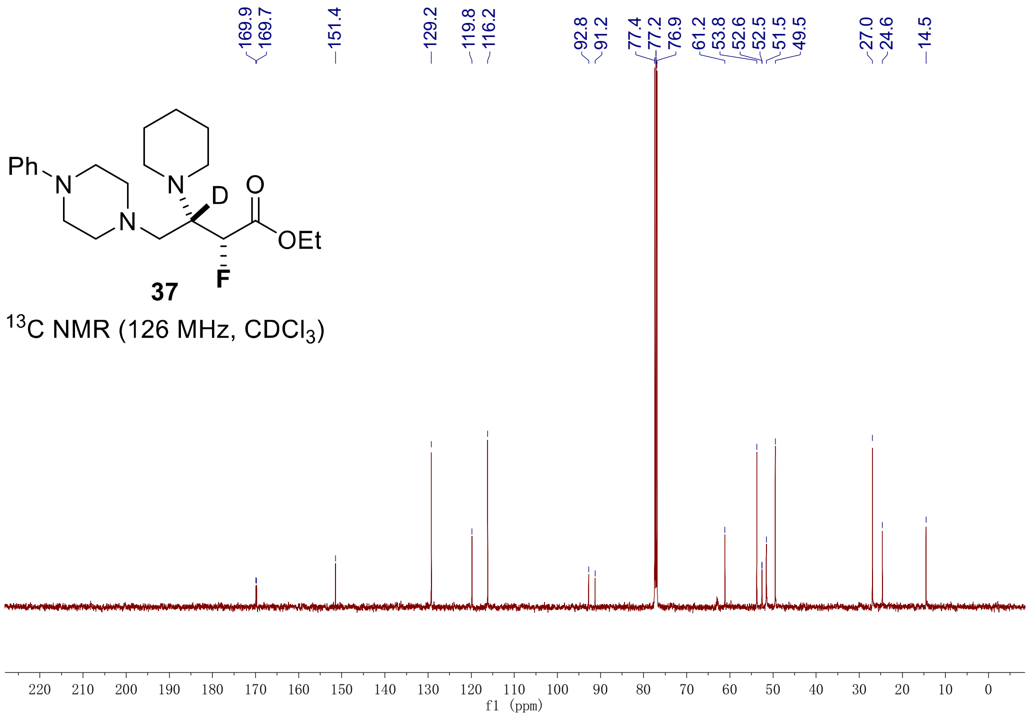

Supplementary Fig. 88. ^{13}C NMR (126 MHz, $CDCl_3$) spectrum of compound **37**.

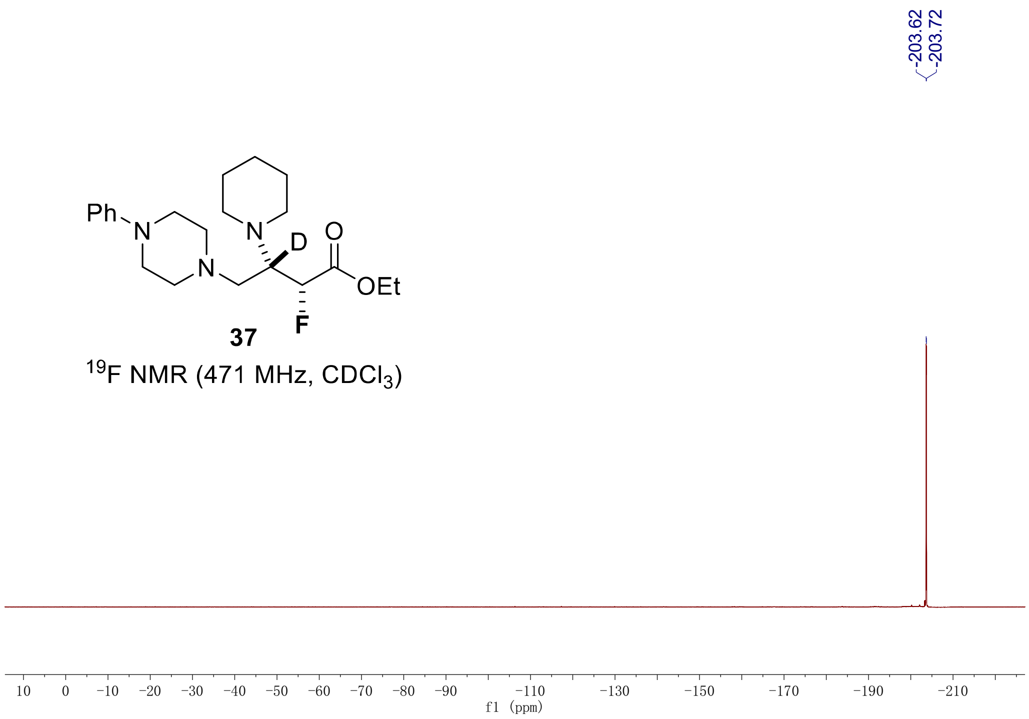

Supplementary Fig. 89. ^{19}F NMR (471 MHz, $CDCl_3$) spectrum of compound **37**.

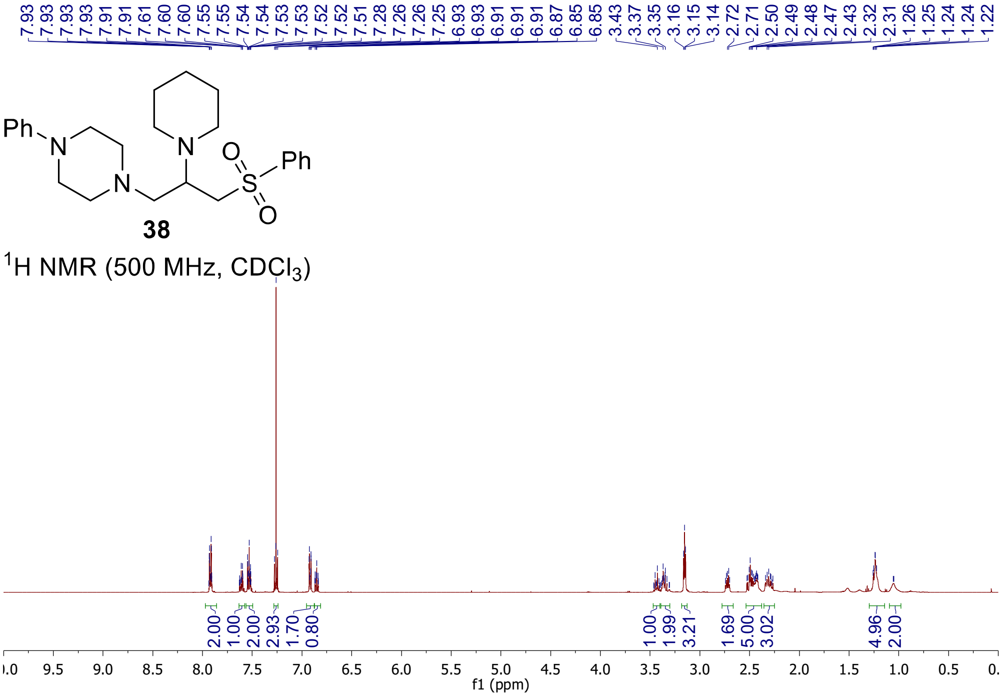

Supplementary Fig. 90. ^{1}H NMR (500 MHz, $CDCl_3$) spectrum of compound **38**.

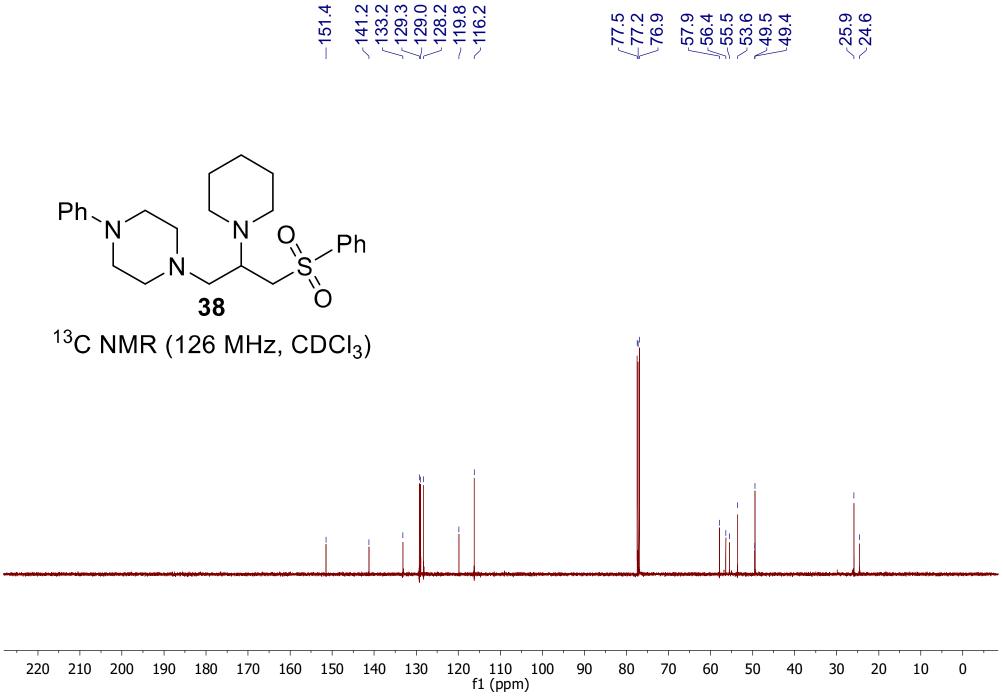

Supplementary Fig. 91. ^{13}C NMR (126 MHz, $CDCl_3$) spectrum of compound **38**.

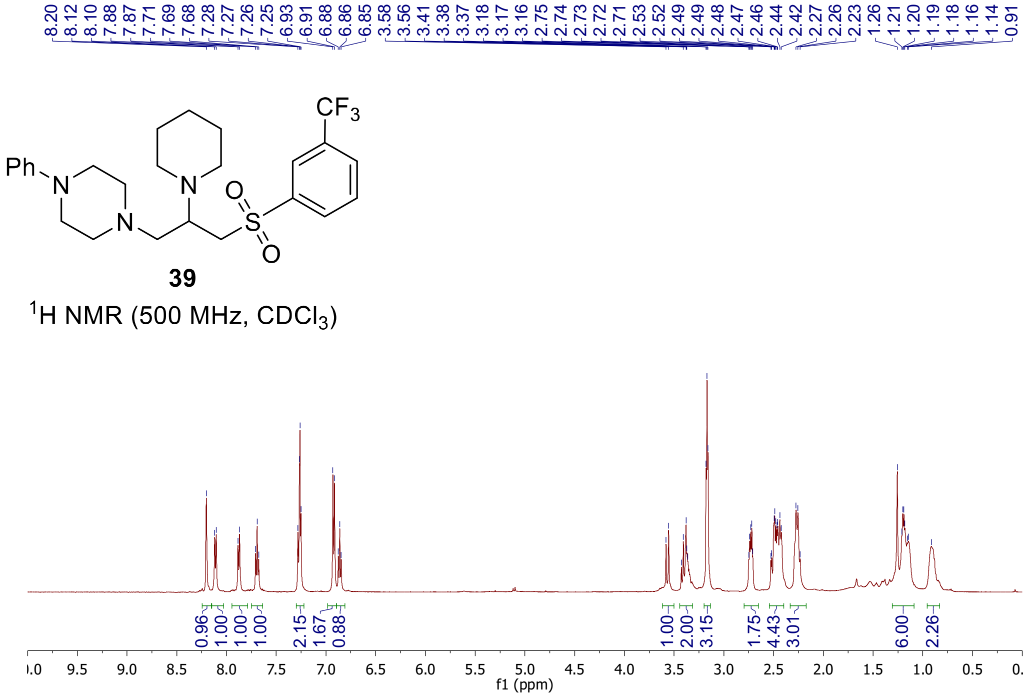

Supplementary Fig. 92. ^{1}H NMR (500 MHz, $CDCl_3$) spectrum of compound **39**.

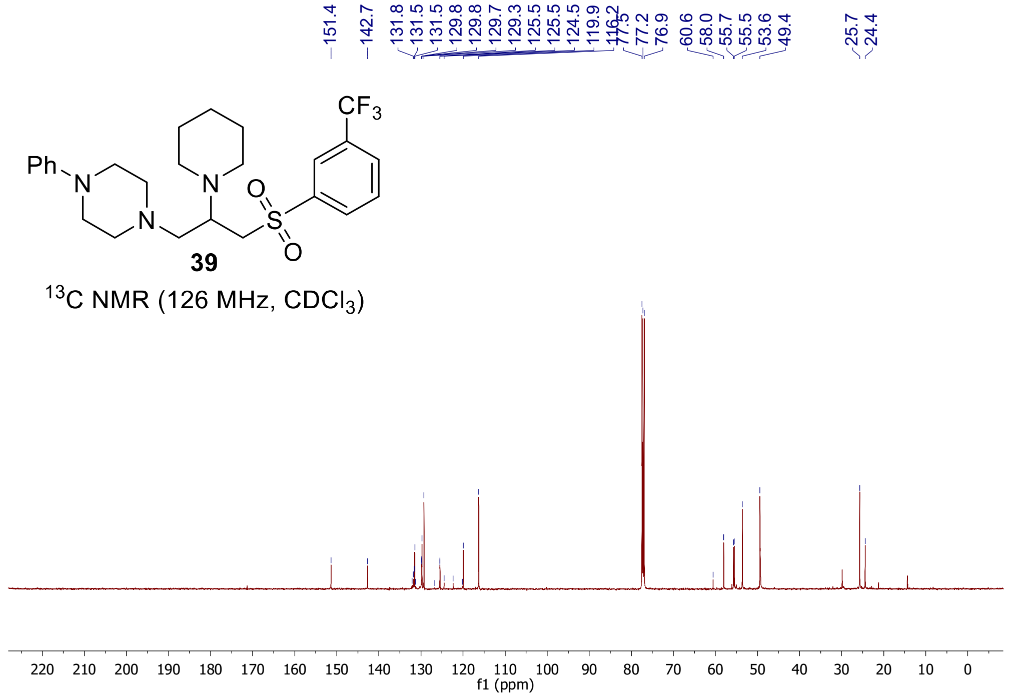

Supplementary Fig. 93. ^{13}C NMR (126 MHz, $CDCl_3$) spectrum of compound **39**.

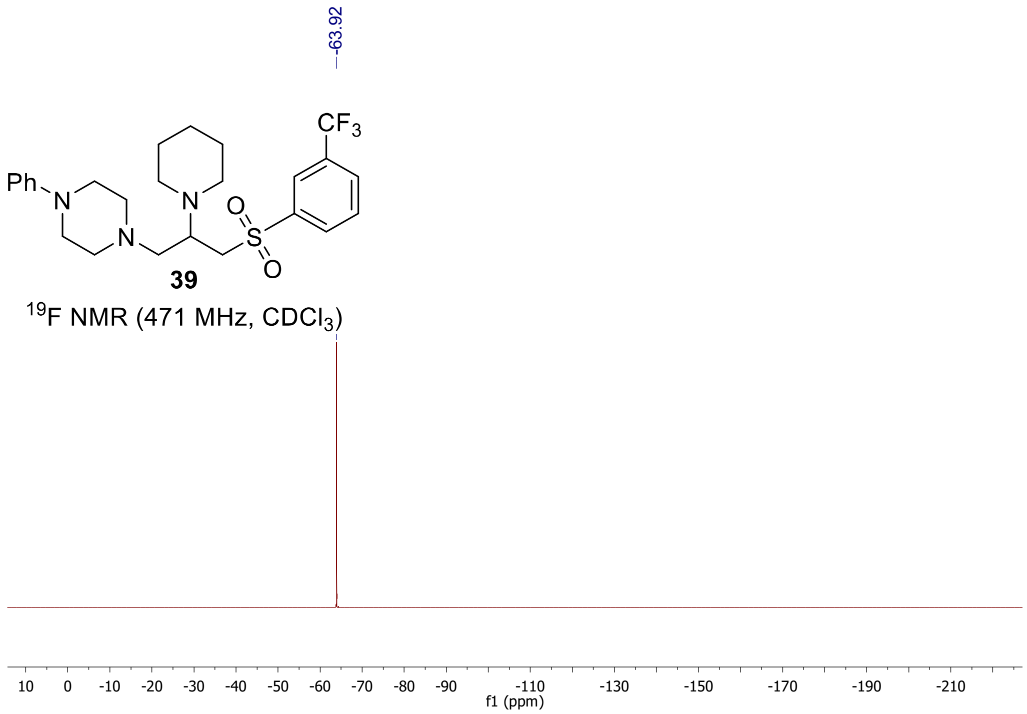

Supplementary Fig. 94. ^{19}F NMR (471 MHz, $CDCl_3$) spectrum of compound **39**.

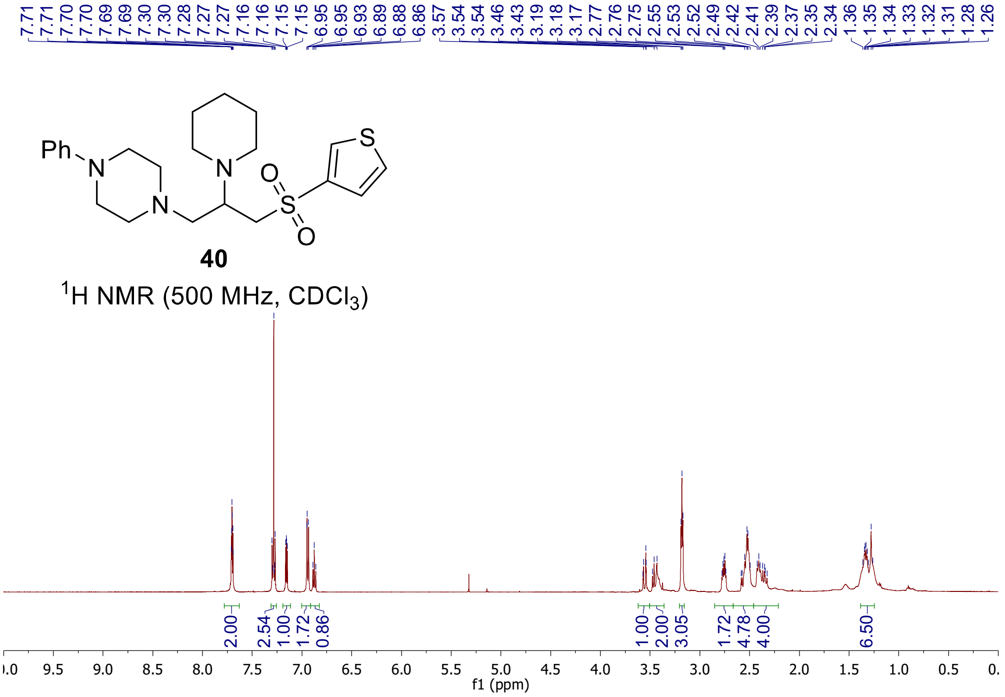

Supplementary Fig. 95. ^{1}H NMR (500 MHz, $CDCl_3$) spectrum of compound **40**.

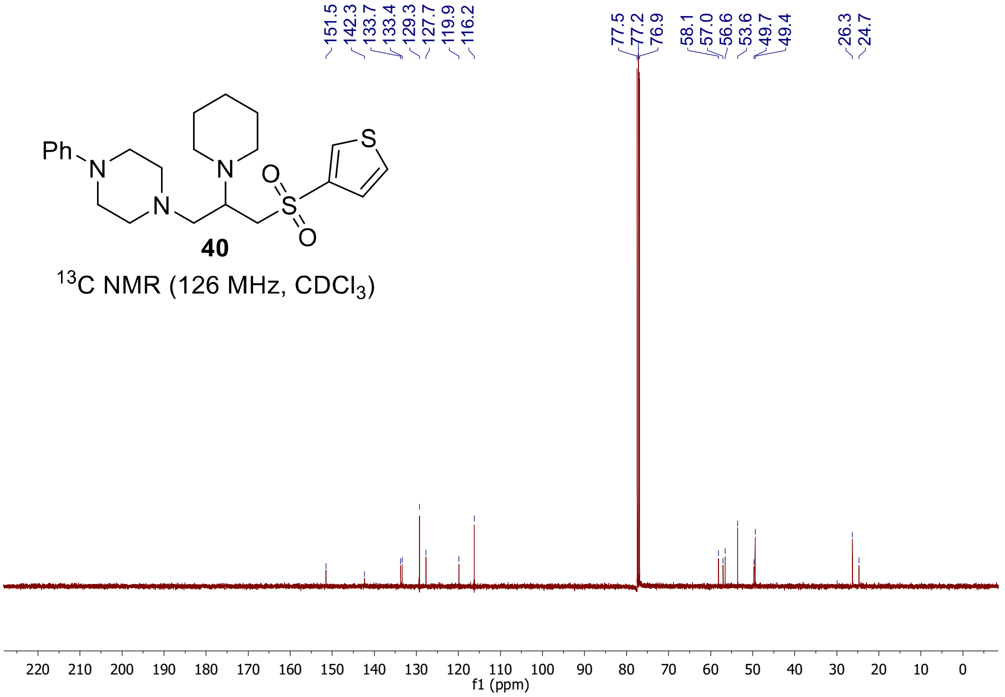

Supplementary Fig. 96. ^{13}C NMR (126 MHz, $CDCl_3$) spectrum of compound **40**.

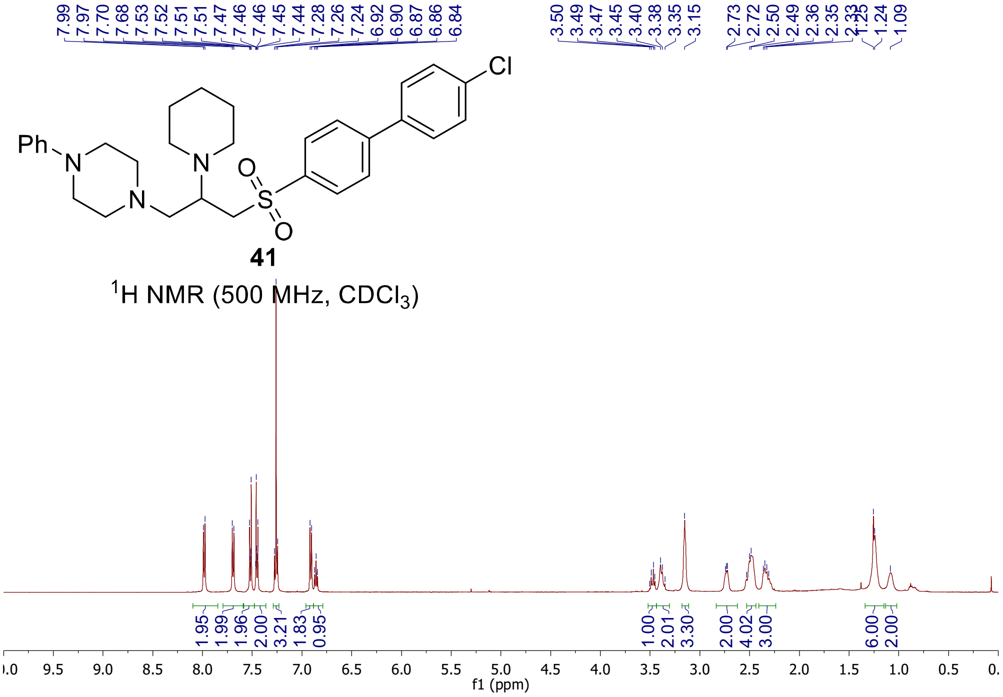

Supplementary Fig. 97. ^{1}H NMR (500 MHz, $CDCl_3$) spectrum of compound **41**.

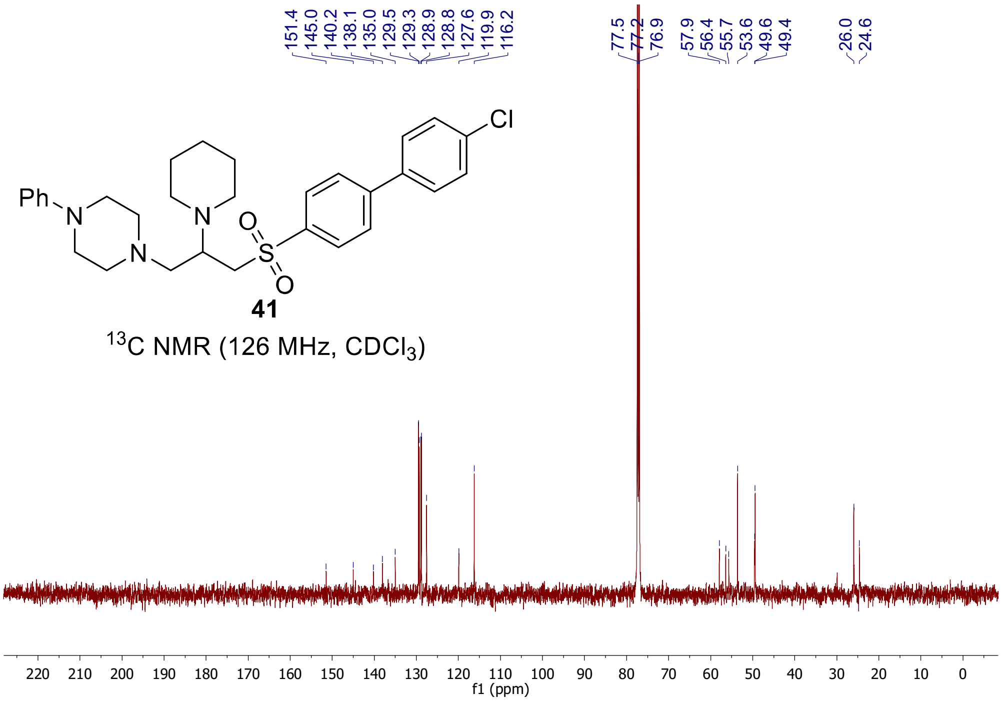

Supplementary Fig. 98. ^{13}C NMR (126 MHz, $CDCl_3$) spectrum of compound **41**.

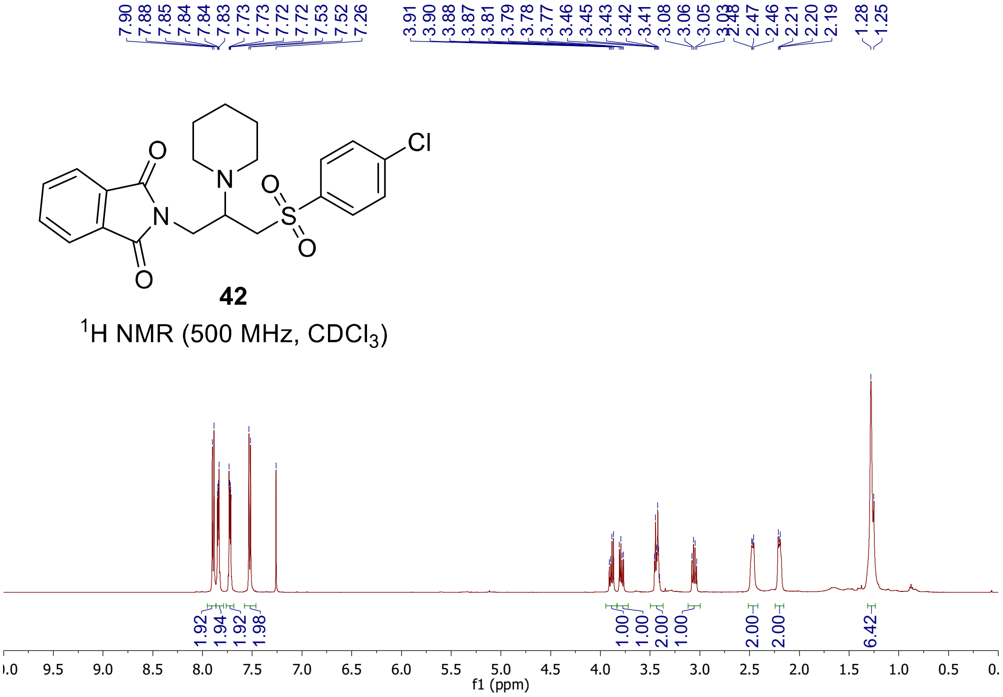

Supplementary Fig. 99. ^{1}H NMR (500 MHz, $CDCl_3$) spectrum of compound **42**.

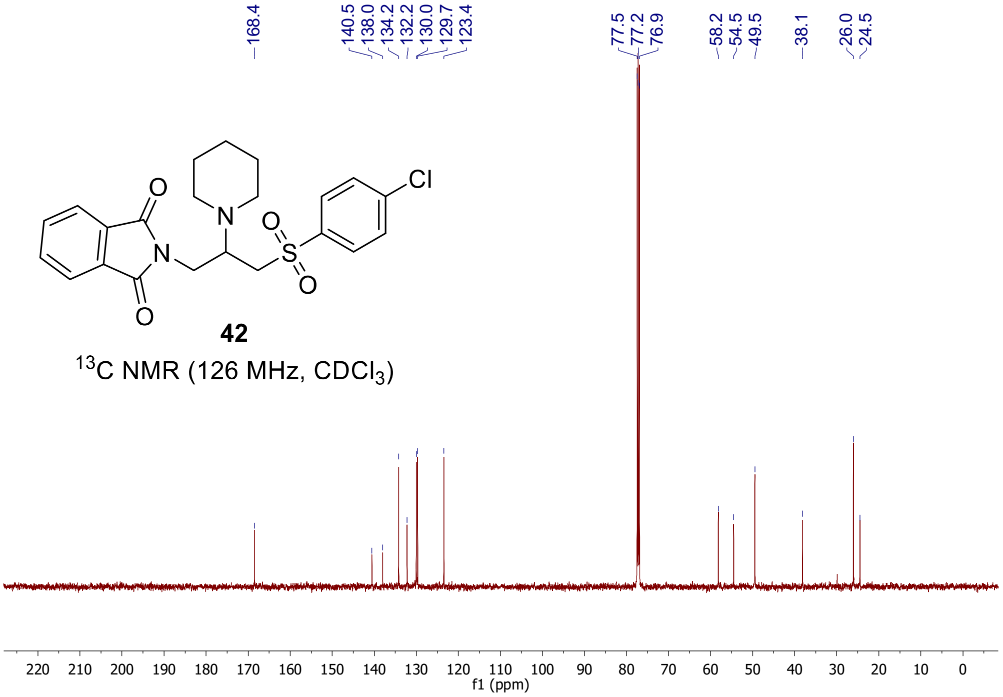

Supplementary Fig. 100. ^{13}C NMR (126 MHz, $CDCl_3$) spectrum of compound **42**.

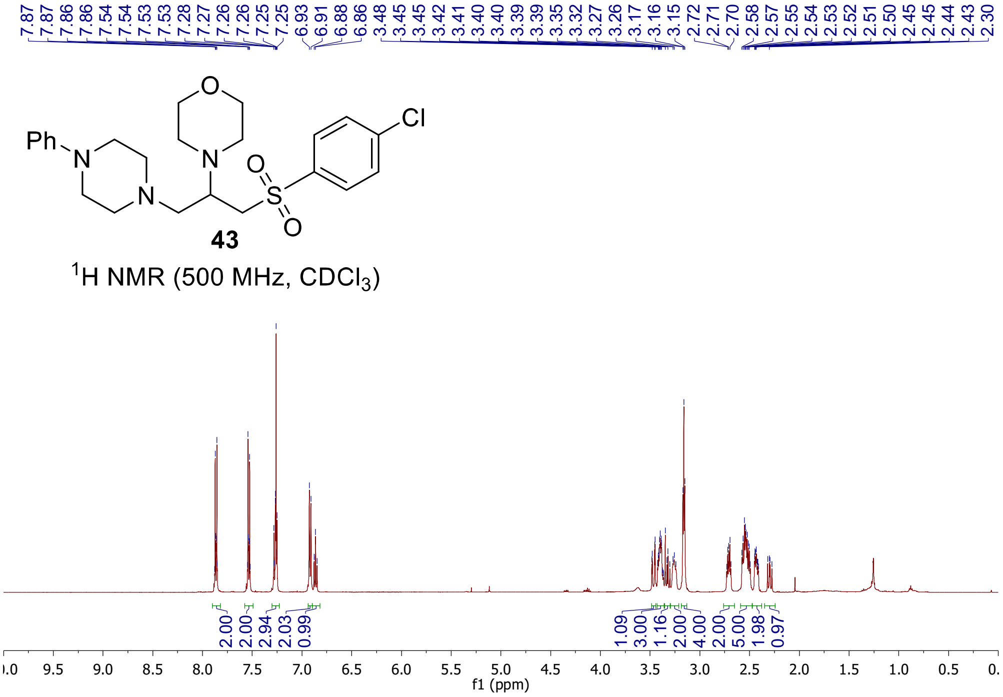

Supplementary Fig. 101. ^{1}H NMR (500 MHz, CDCl$_3$) spectrum of compound **43**.

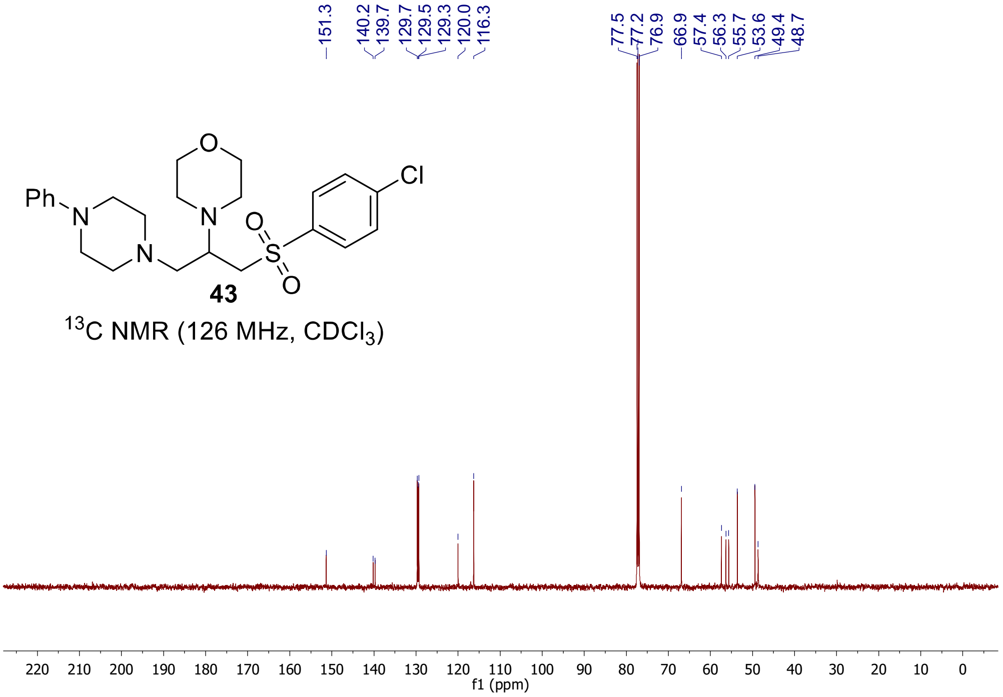

Supplementary Fig. 102. ^{13}C NMR (126 MHz, CDCl$_3$) spectrum of compound **43**.

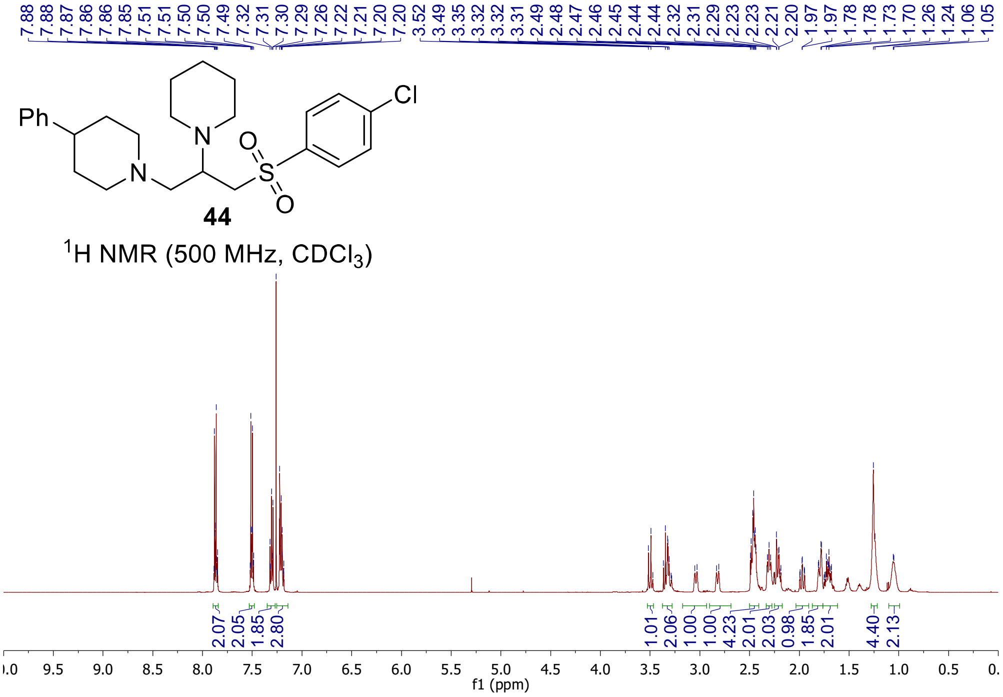

Supplementary Fig. 103. ^{1}H NMR (500 MHz, $CDCl_3$) spectrum of compound **44**.

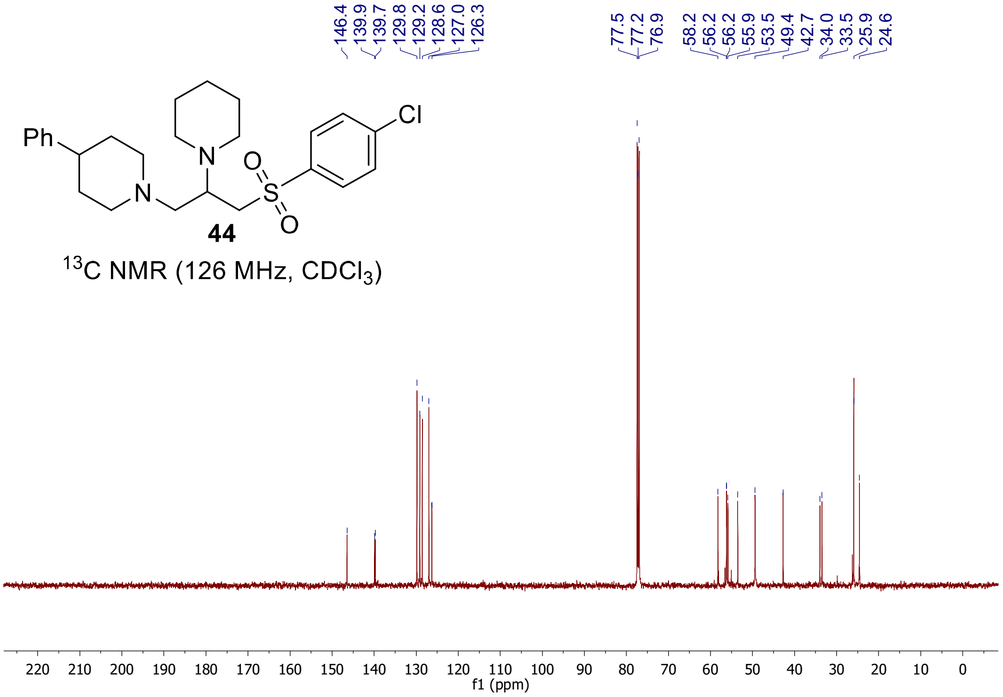

Supplementary Fig. 104. ^{13}C NMR (126 MHz, $CDCl_3$) spectrum of compound **44**.

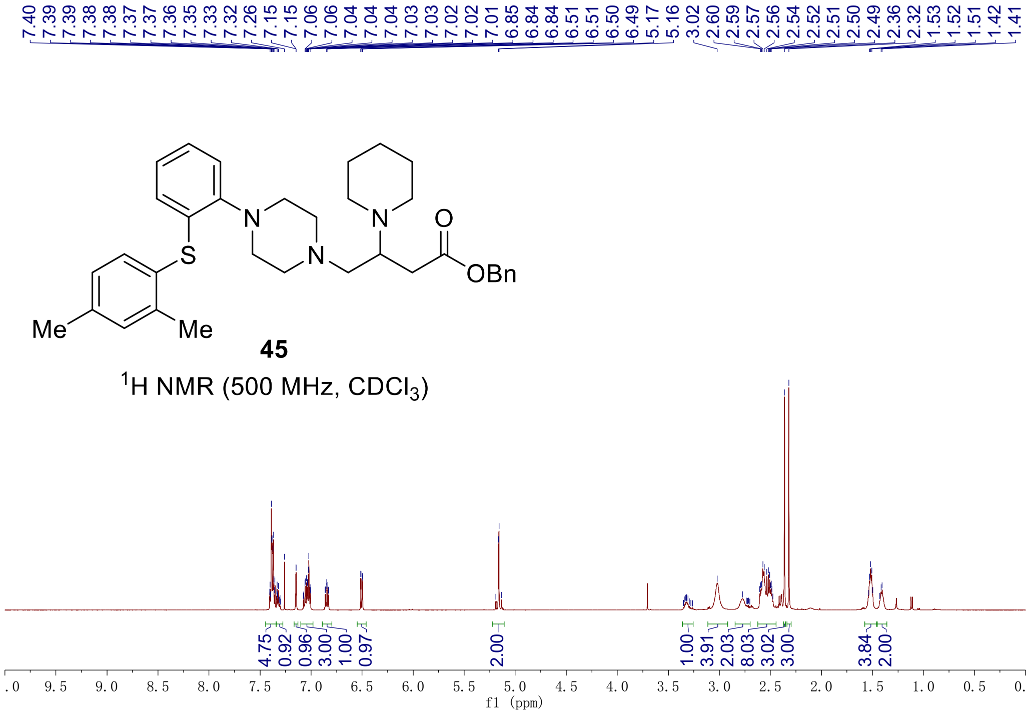

Supplementary Fig. 105. ^{1}H NMR (500 MHz, $CDCl_3$) spectrum of compound **45**.

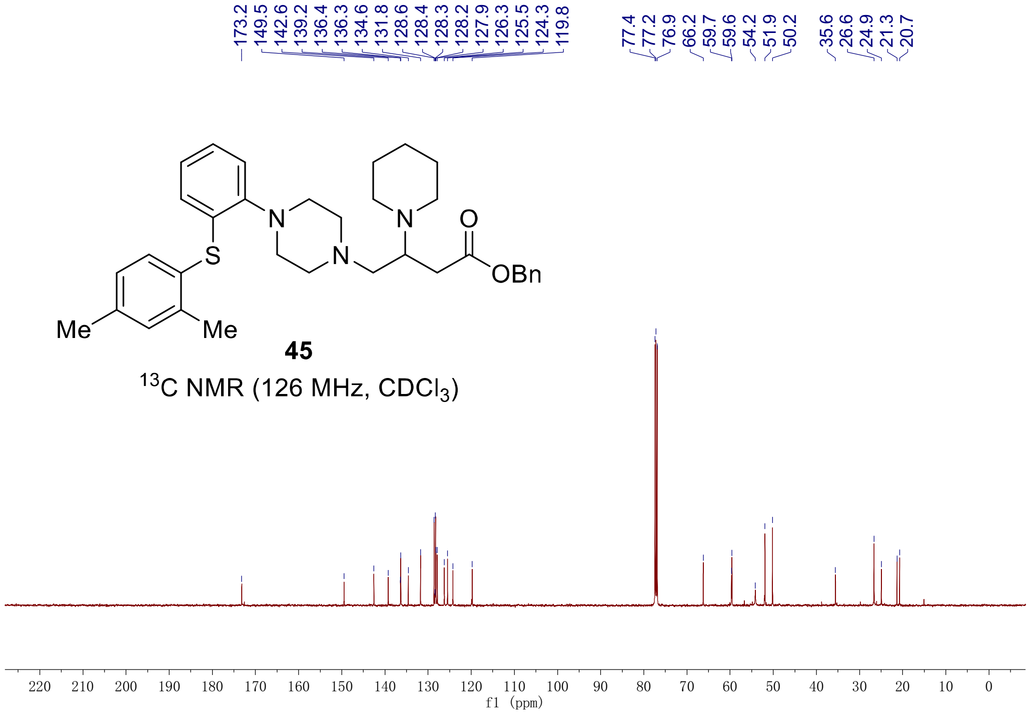

Supplementary Fig. 106. ^{13}C NMR (126 MHz, $CDCl_3$) spectrum of compound **45**.

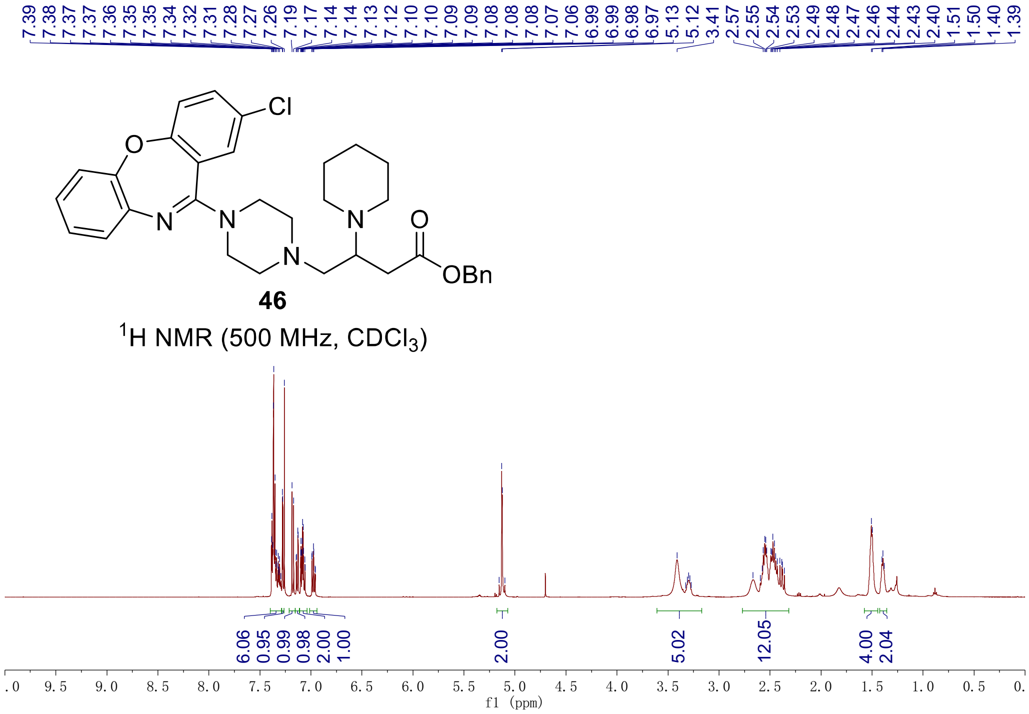

Supplementary Fig. 107. ^{1}H NMR (500 MHz, CDCl$_3$) spectrum of compound **46**.

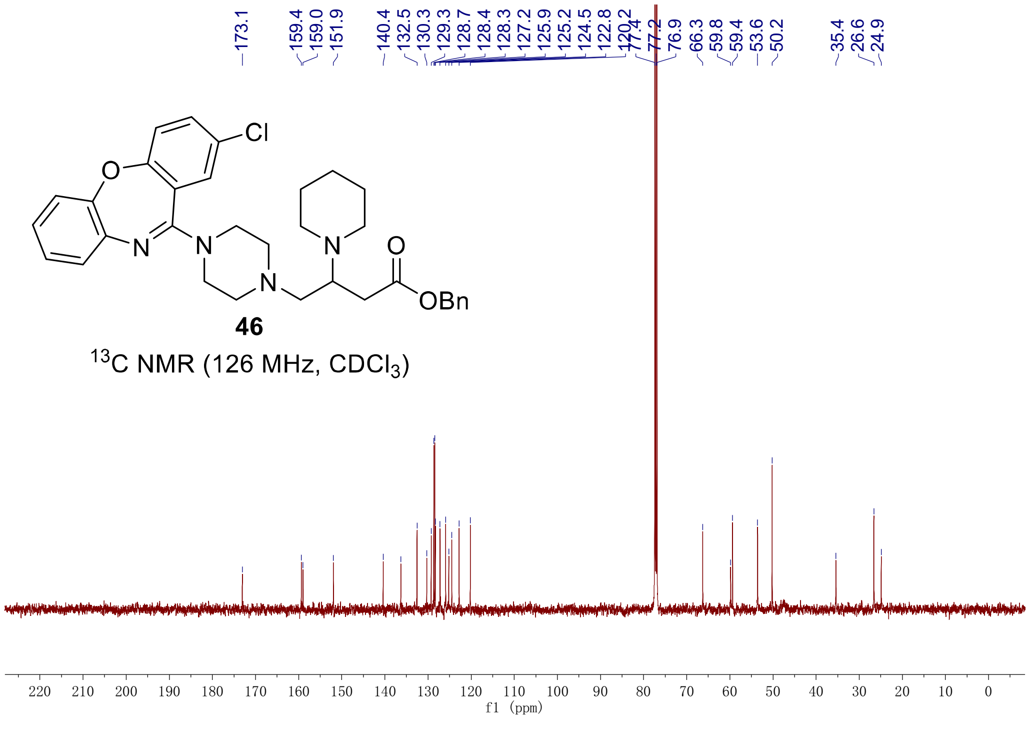

Supplementary Fig. 108. ^{13}C NMR (126 MHz, CDCl$_3$) spectrum of compound **46**.

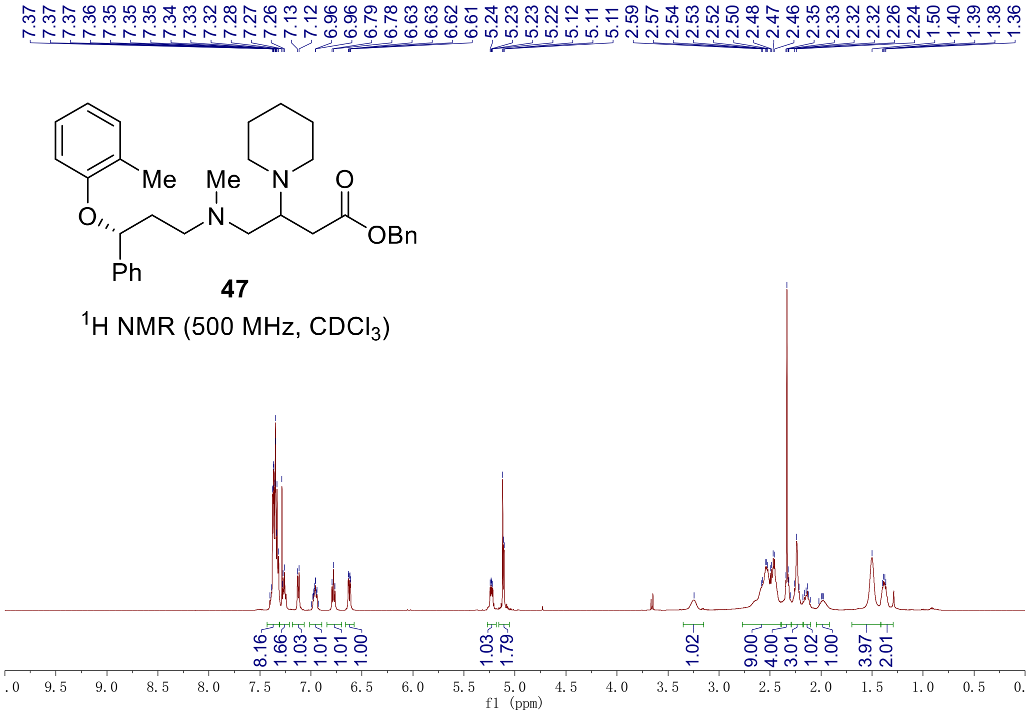

Supplementary Fig. 109. ^{1}H NMR (500 MHz, $CDCl_3$) spectrum of compound **47**.

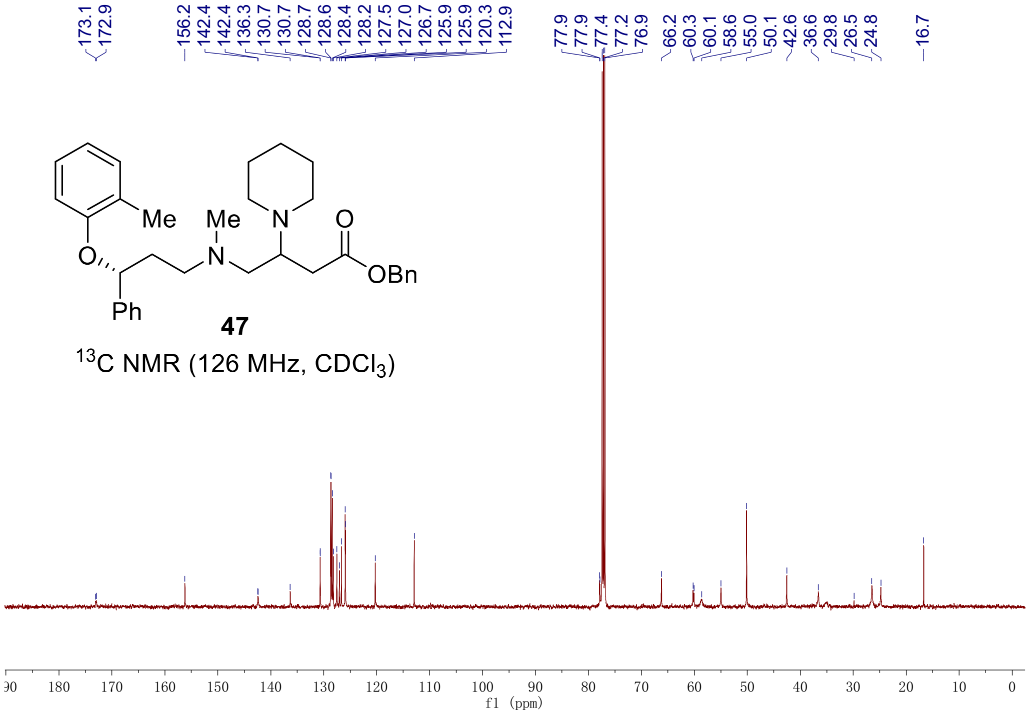

Supplementary Fig. 110. ^{13}C NMR (126 MHz, $CDCl_3$) spectrum of compound **47**.

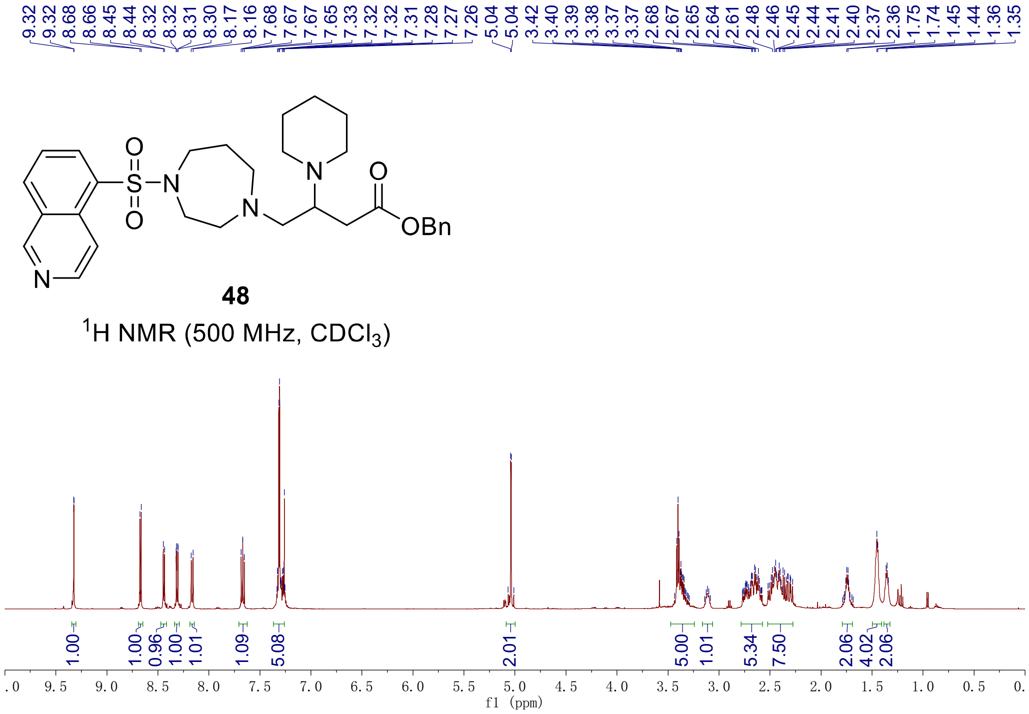

Supplementary Fig. 111. ^{1}H NMR (500 MHz, CDCl$_3$) spectrum of compound **48**.

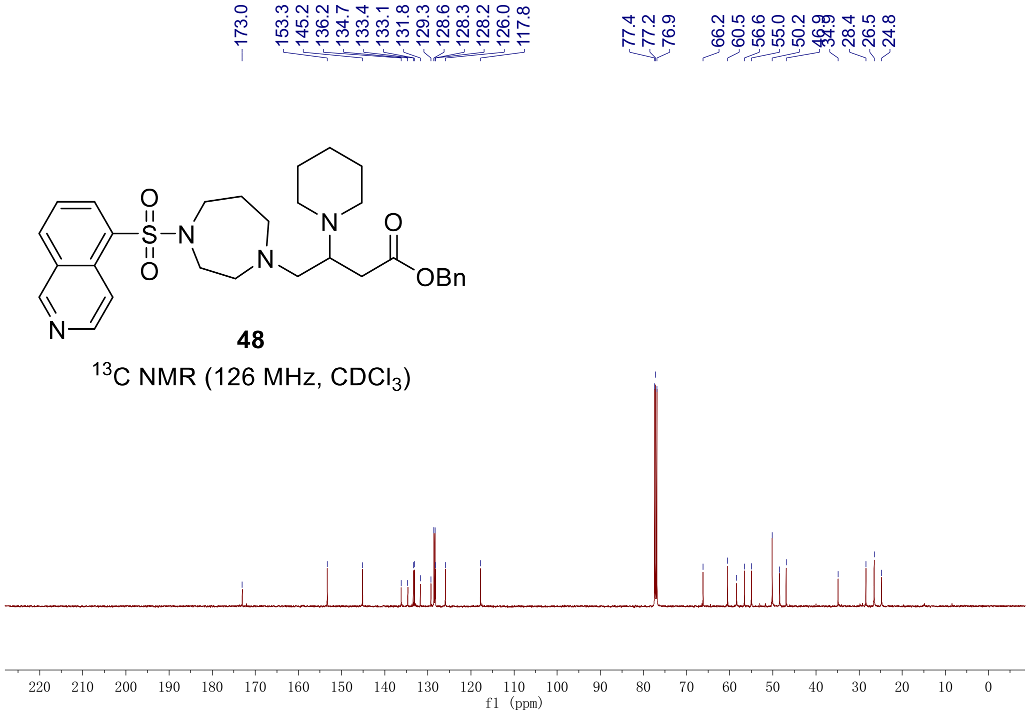

Supplementary Fig. 112. ^{13}C NMR (126 MHz, CDCl$_3$) spectrum of compound **48**.

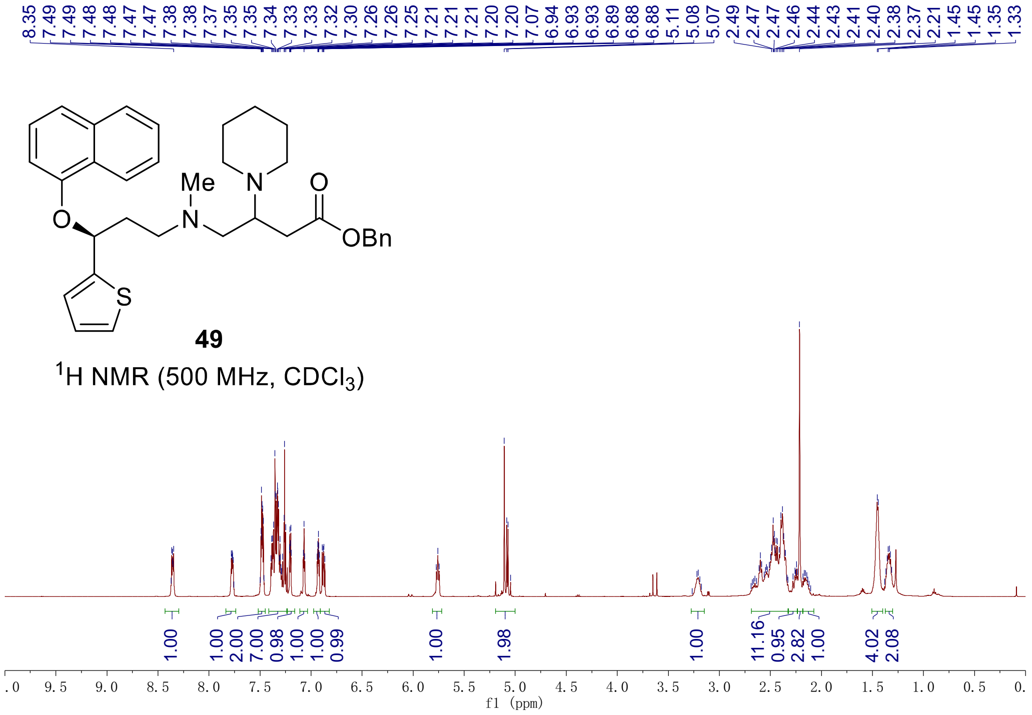

Supplementary Fig. 113. ^{1}H NMR (500 MHz, $CDCl_3$) spectrum of compound **49**.

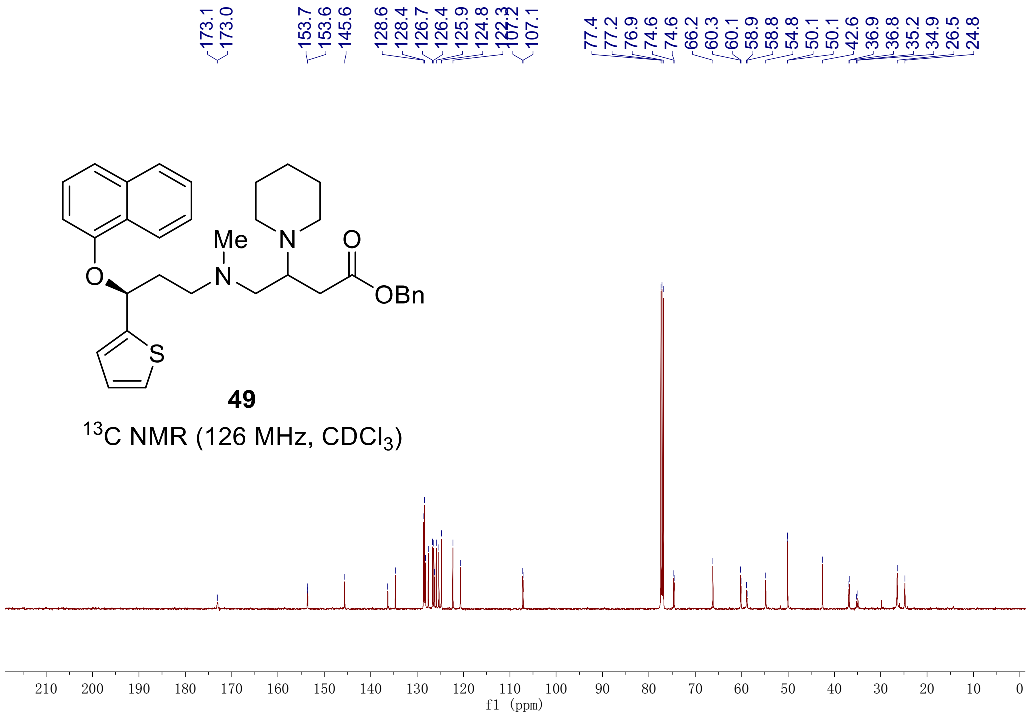

Supplementary Fig. 114. ^{13}C NMR (126 MHz, $CDCl_3$) spectrum of compound **49**.

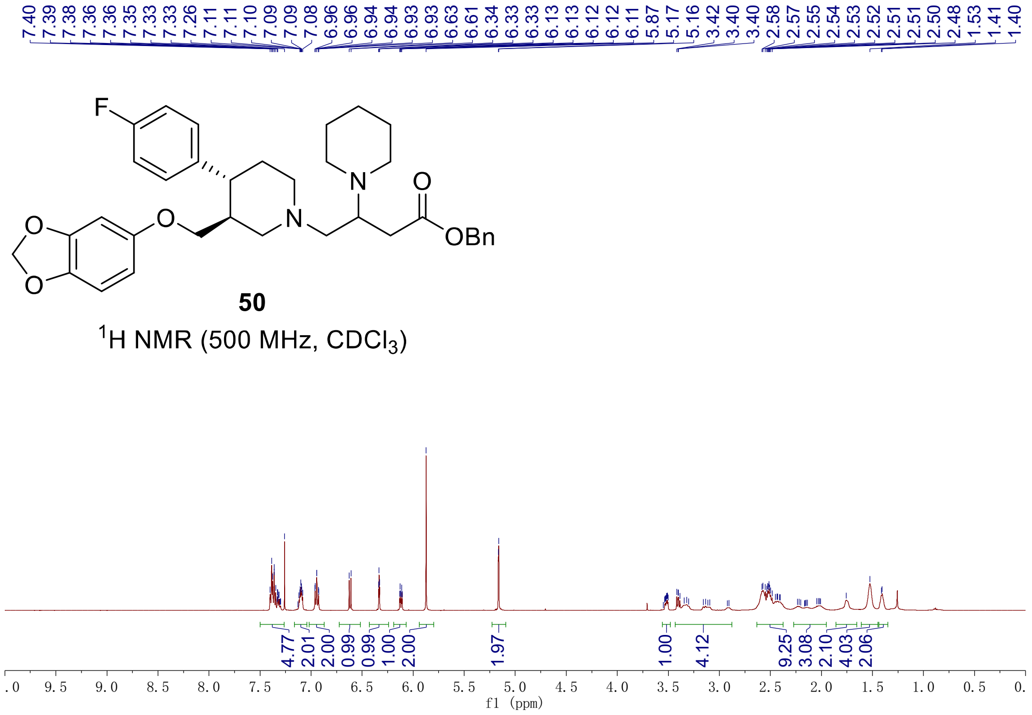

Supplementary Fig. 115. ^{1}H NMR (500 MHz, $CDCl_3$) spectrum of compound **50**.

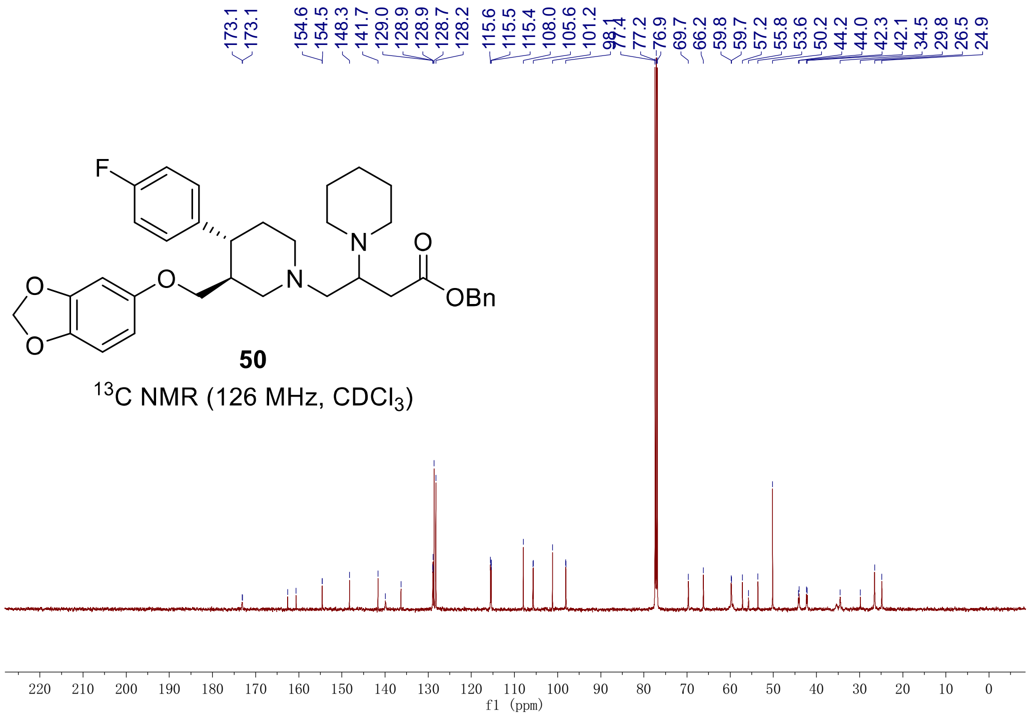

Supplementary Fig. 116. ^{13}C NMR (126 MHz, $CDCl_3$) spectrum of compound **50**.

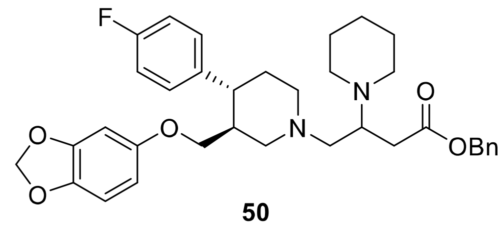

50

^{19}F NMR (471 MHz, $CDCl_3$)

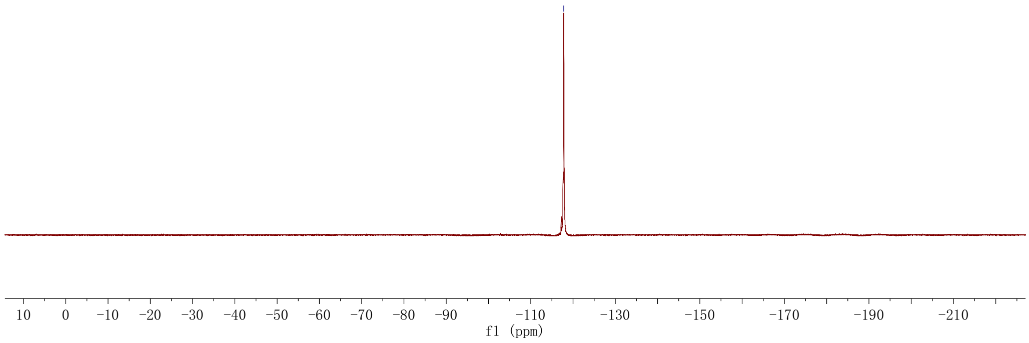

Supplementary Fig. 117. ^{19}F NMR (471 MHz, $CDCl_3$) spectrum of compound **50**.

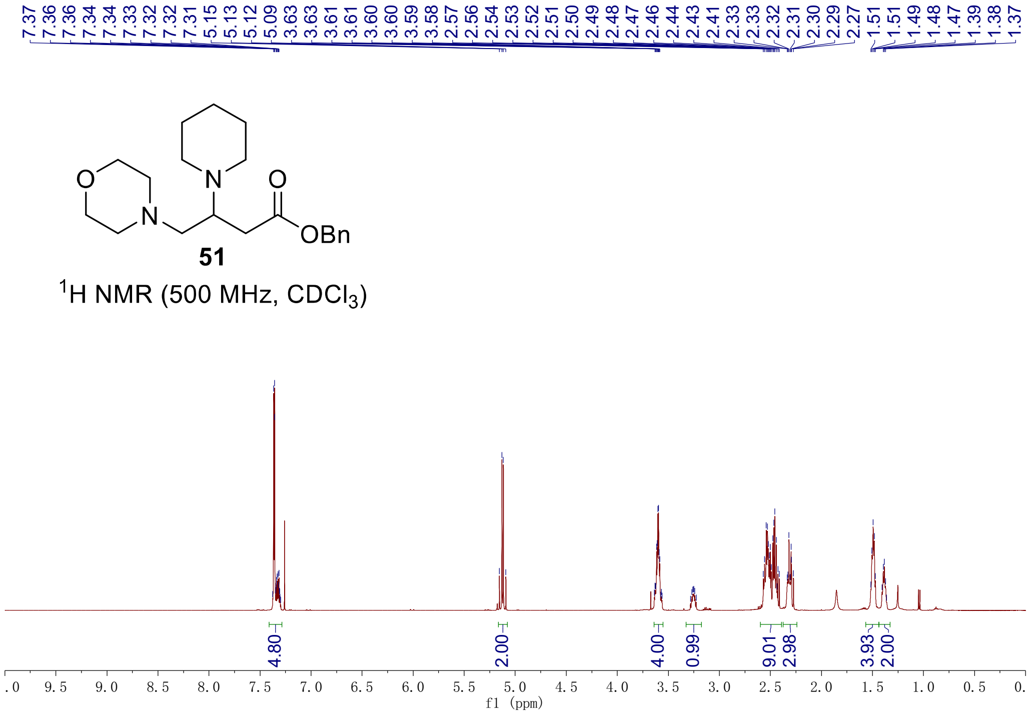

Supplementary Fig. 118. ^{1}H NMR (500 MHz, $CDCl_3$) spectrum of compound **51**.

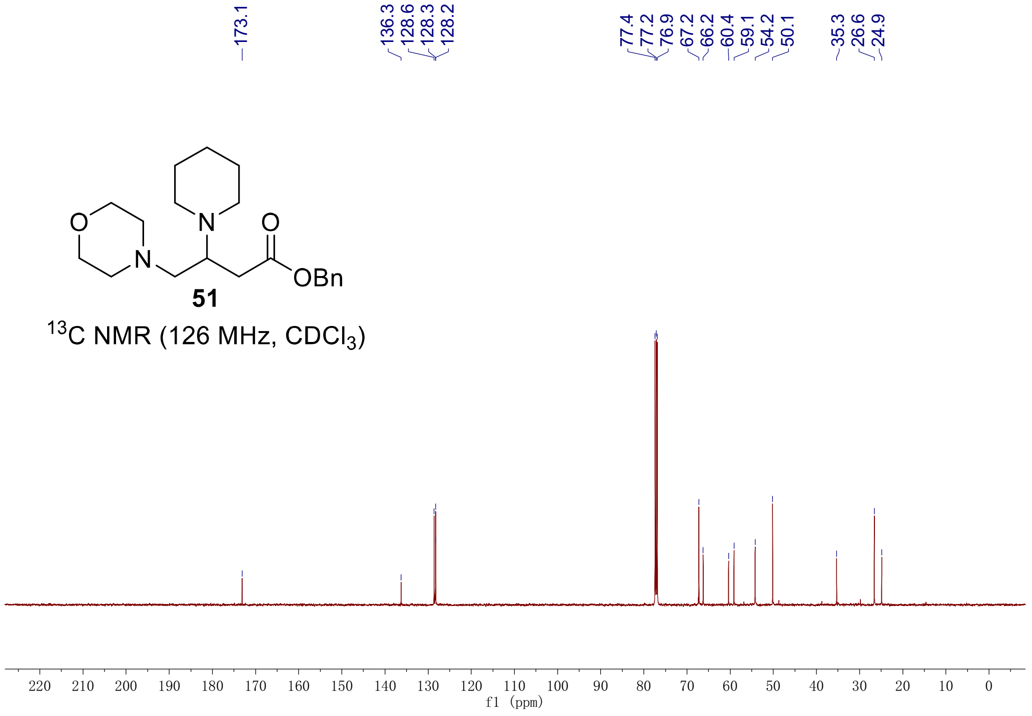

Supplementary Fig. 119. ^{13}C NMR (126 MHz, $CDCl_3$) spectrum of compound **51**.

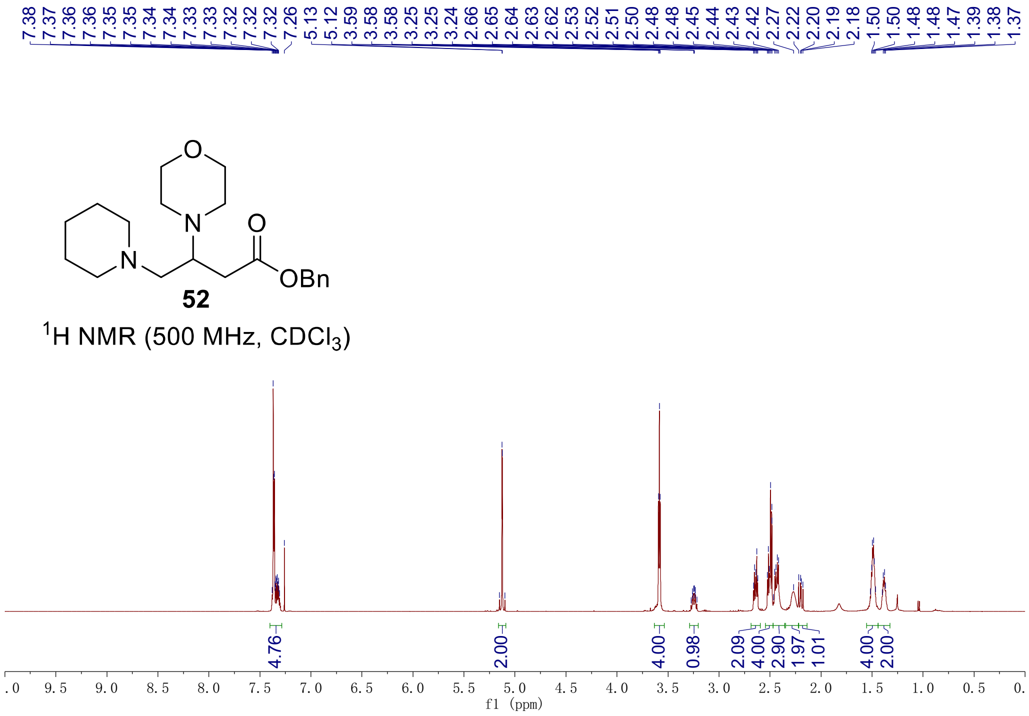

Supplementary Fig. 120. ^{1}H NMR (500 MHz, $CDCl_3$) spectrum of compound **52**.

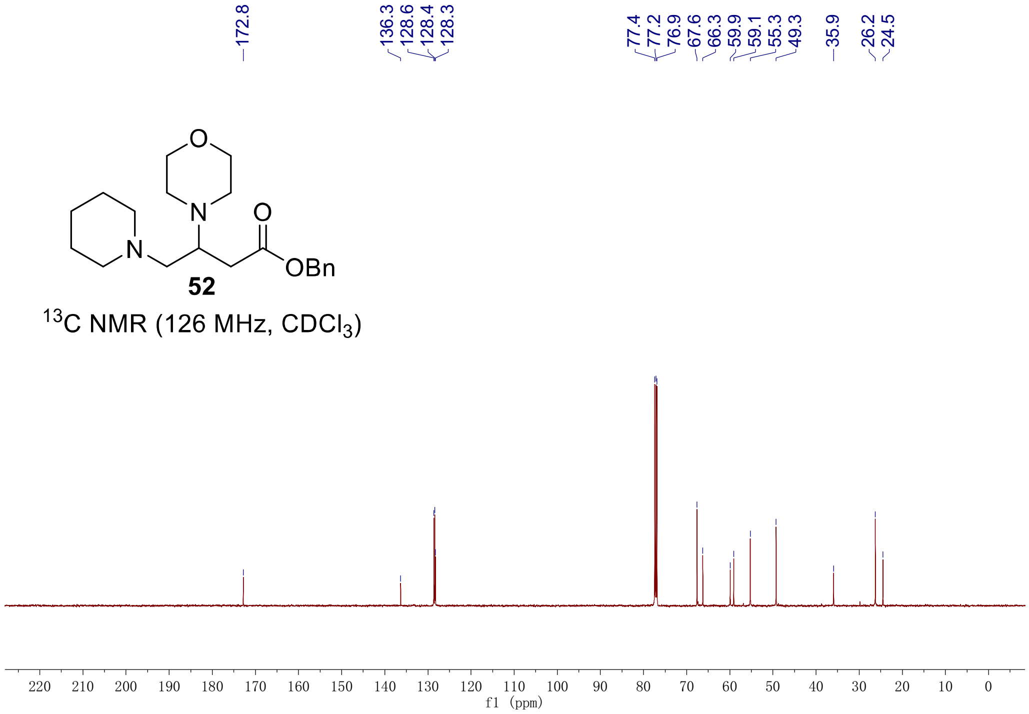

Supplementary Fig. 121. ^{13}C NMR (126 MHz, $CDCl_3$) spectrum of compound **52**.

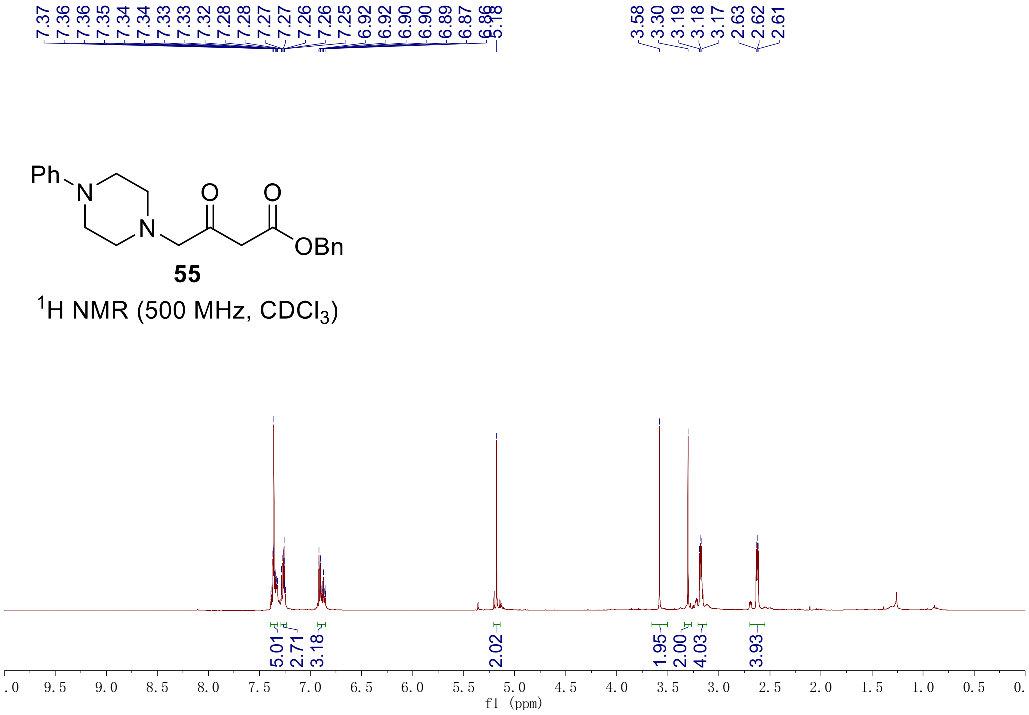

Supplementary Fig. 122. ^{1}H NMR (500 MHz, $CDCl_3$) spectrum of compound **55**.

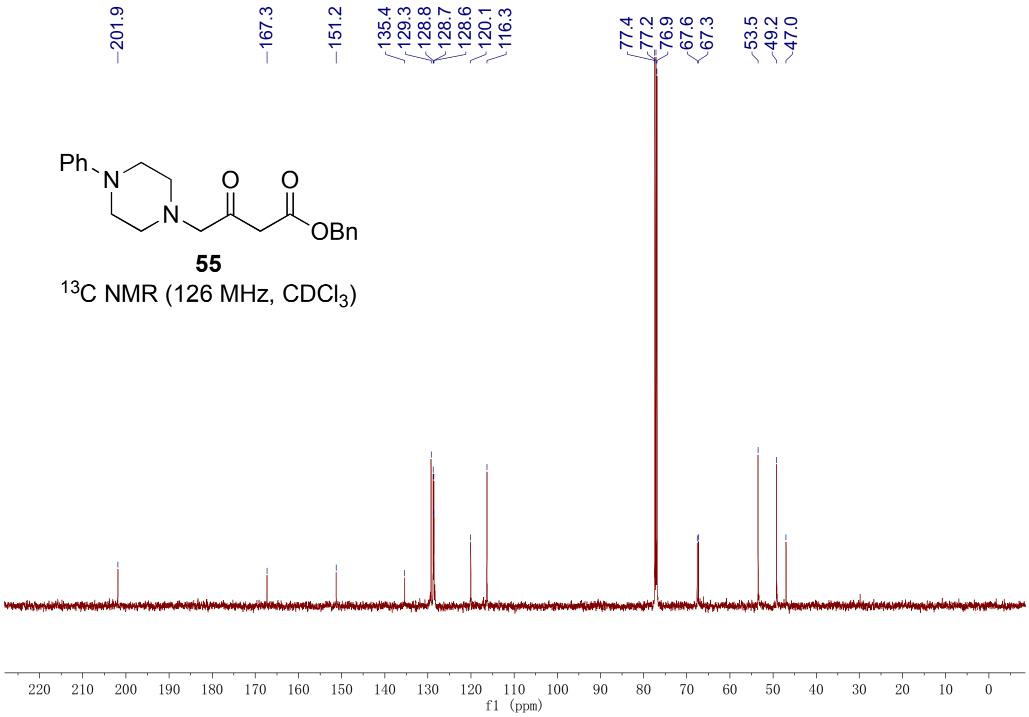

Supplementary Fig. 123. ^{13}C NMR (126 MHz, $CDCl_3$) spectrum of compound **55**.

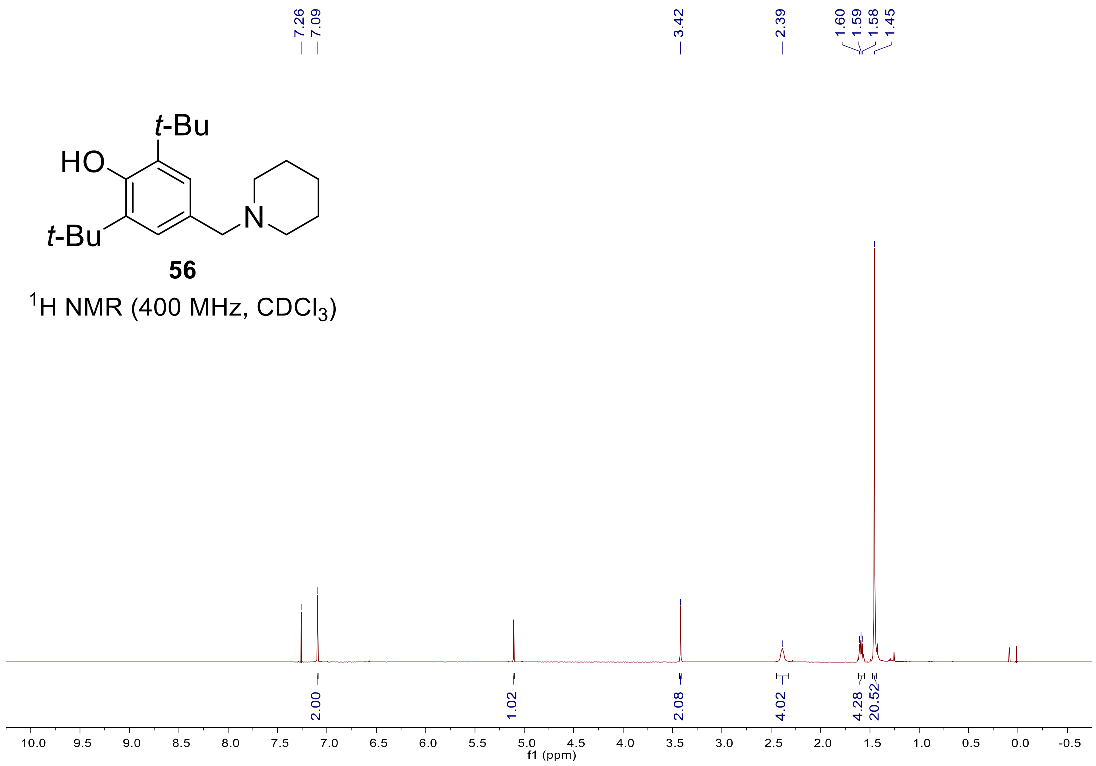

Supplementary Fig. 124. ^{1}H NMR (400 MHz, $CDCl_3$) spectrum of compound **56**.

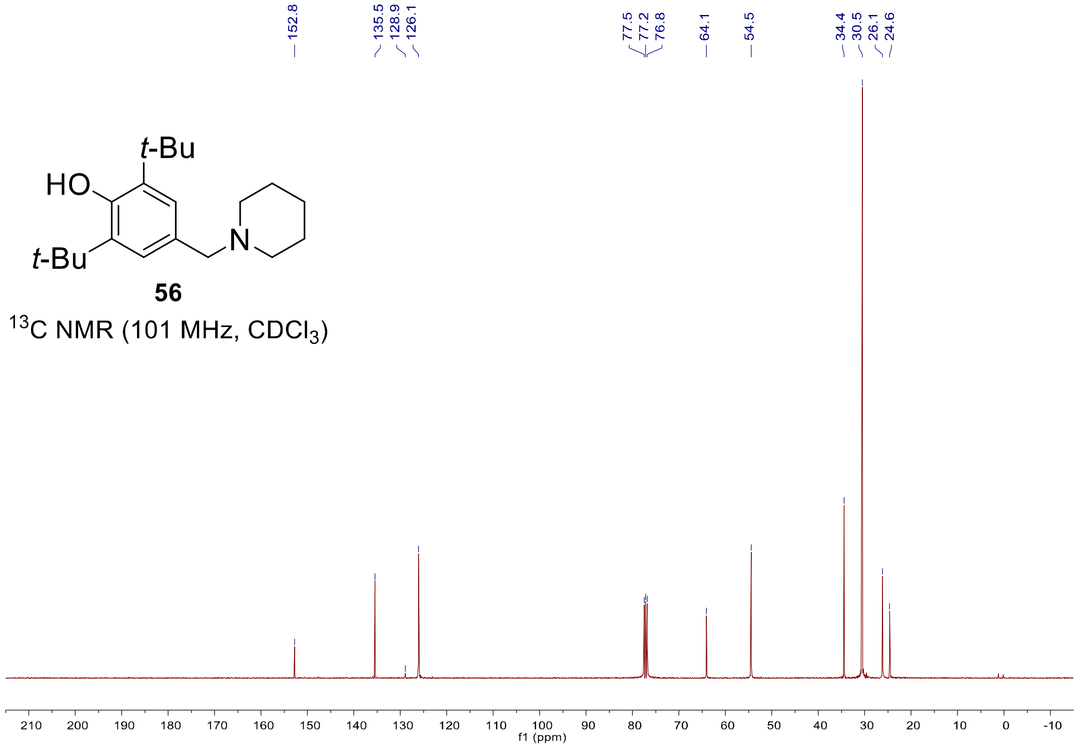

Supplementary Fig. 125. ^{13}C NMR (101 MHz, $CDCl_3$) spectrum of compound **56**.

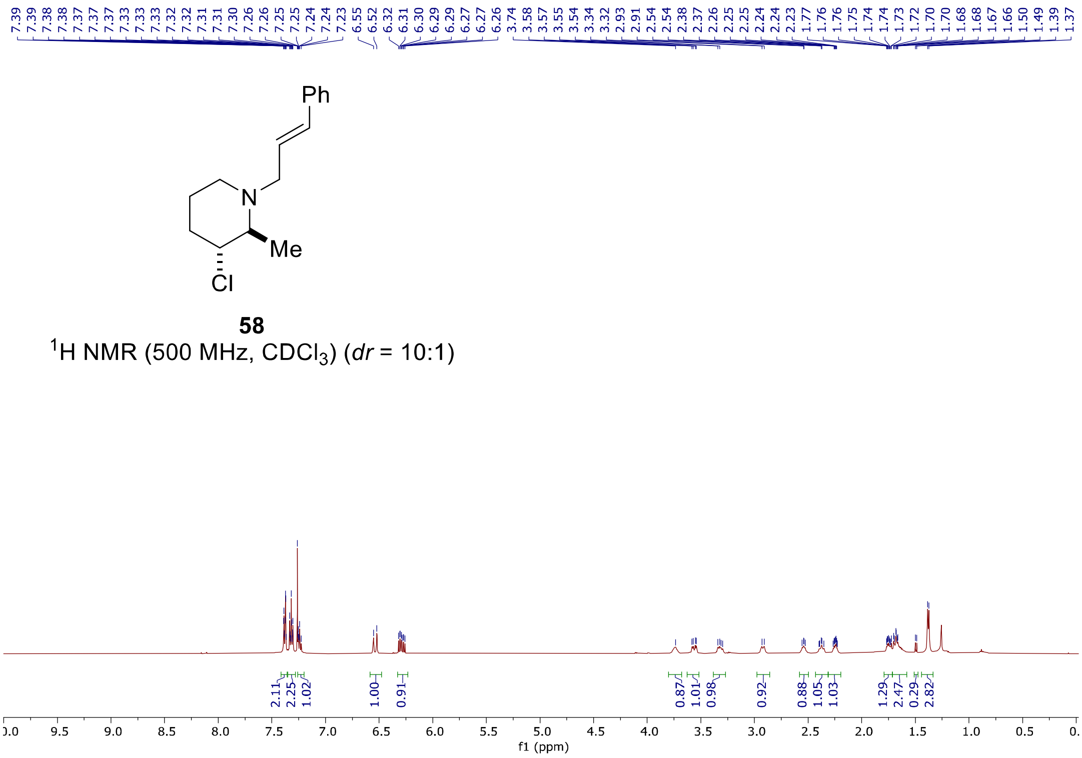

Supplementary Fig. 126. ^{1}H NMR (500 MHz, $CDCl_3$) spectrum of compound **58**.

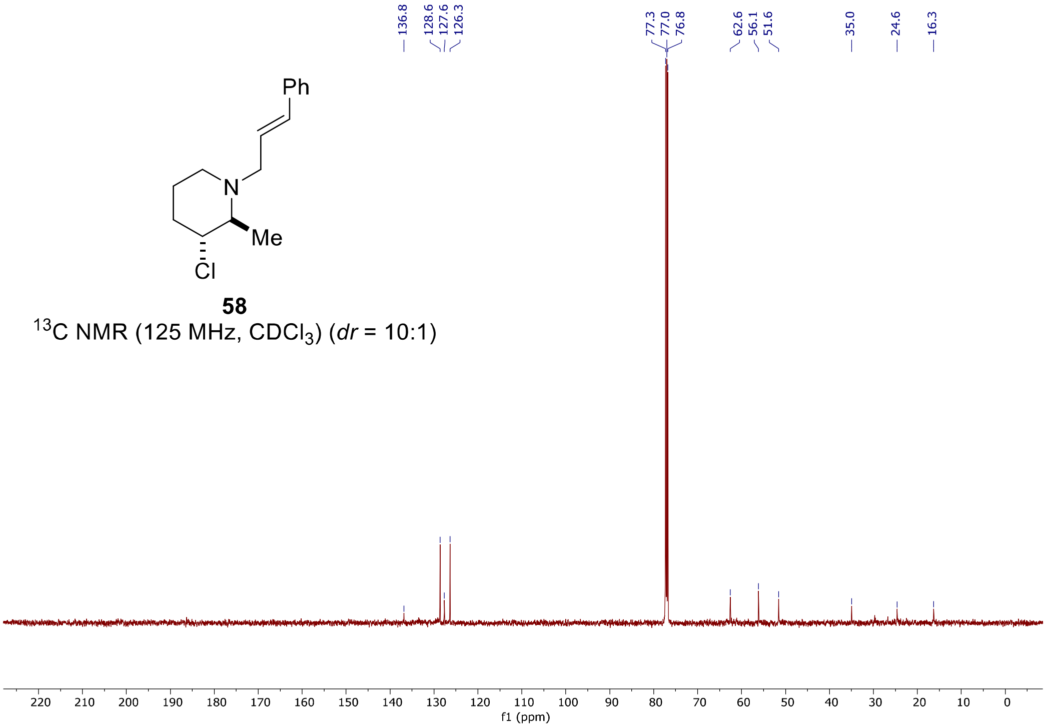

Supplementary Fig. 127. ^{13}C NMR (125 MHz, $CDCl_3$) spectrum of compound **58**.

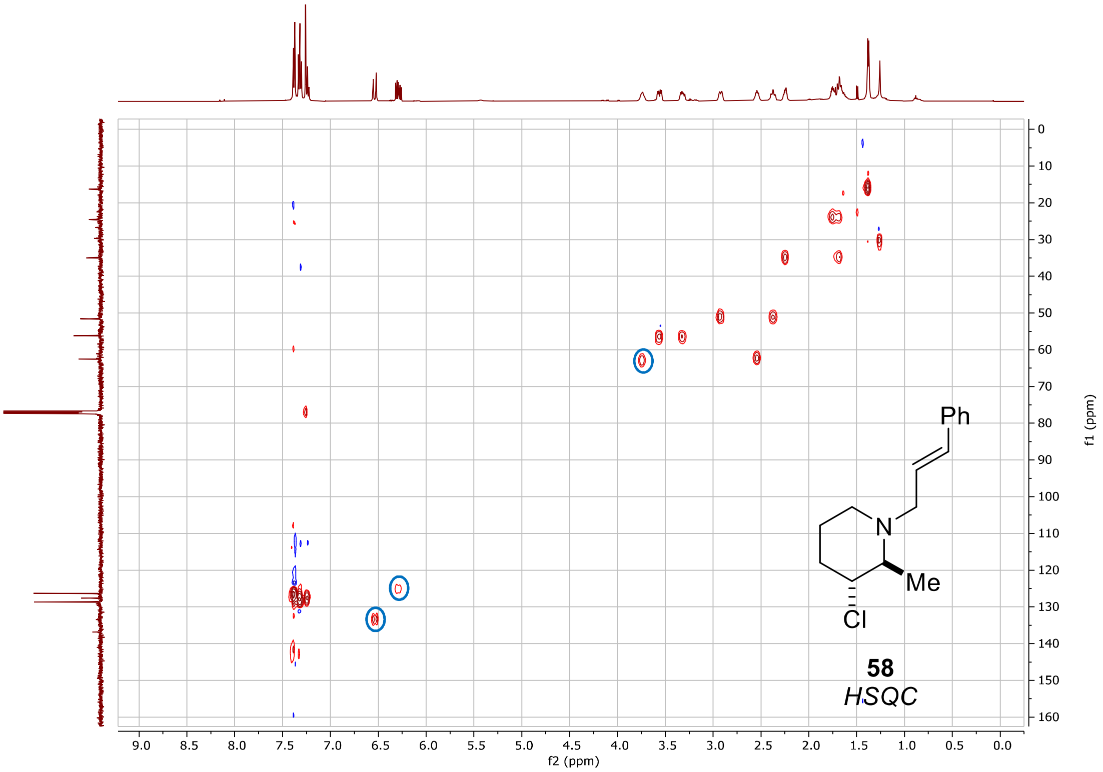

Supplementary Fig. 128. HSQC spectrum of compound **58**.

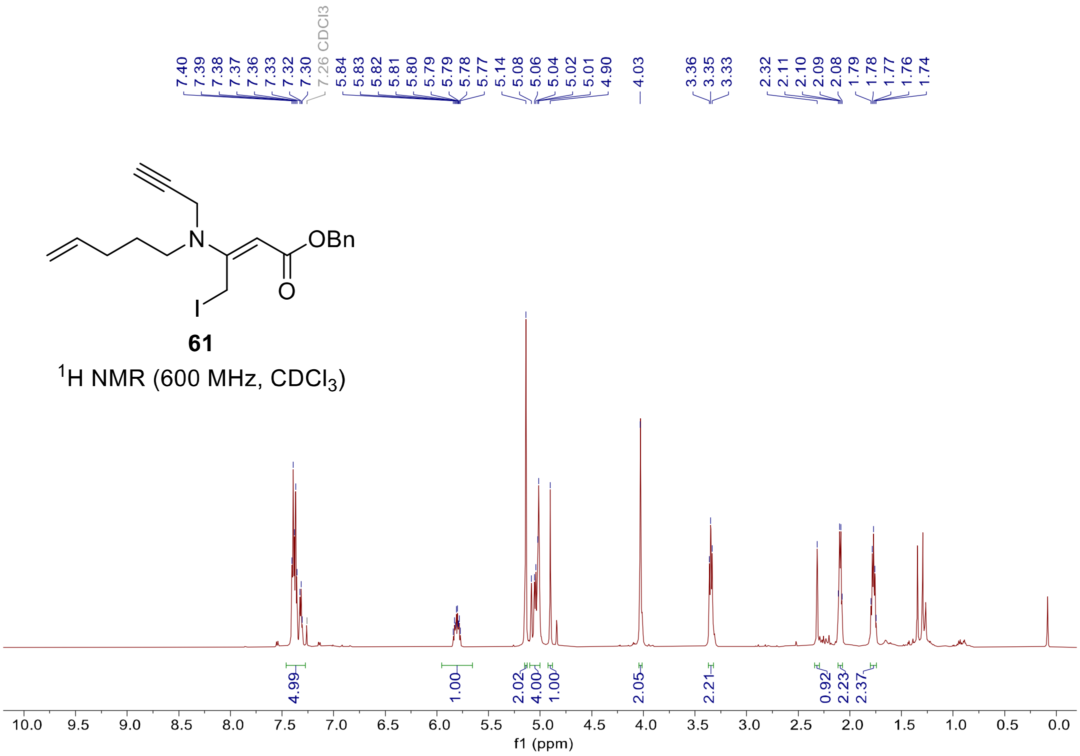

Supplementary Fig. 129. ^{1}H NMR (600 MHz, $CDCl_3$) spectrum of compound **61**.

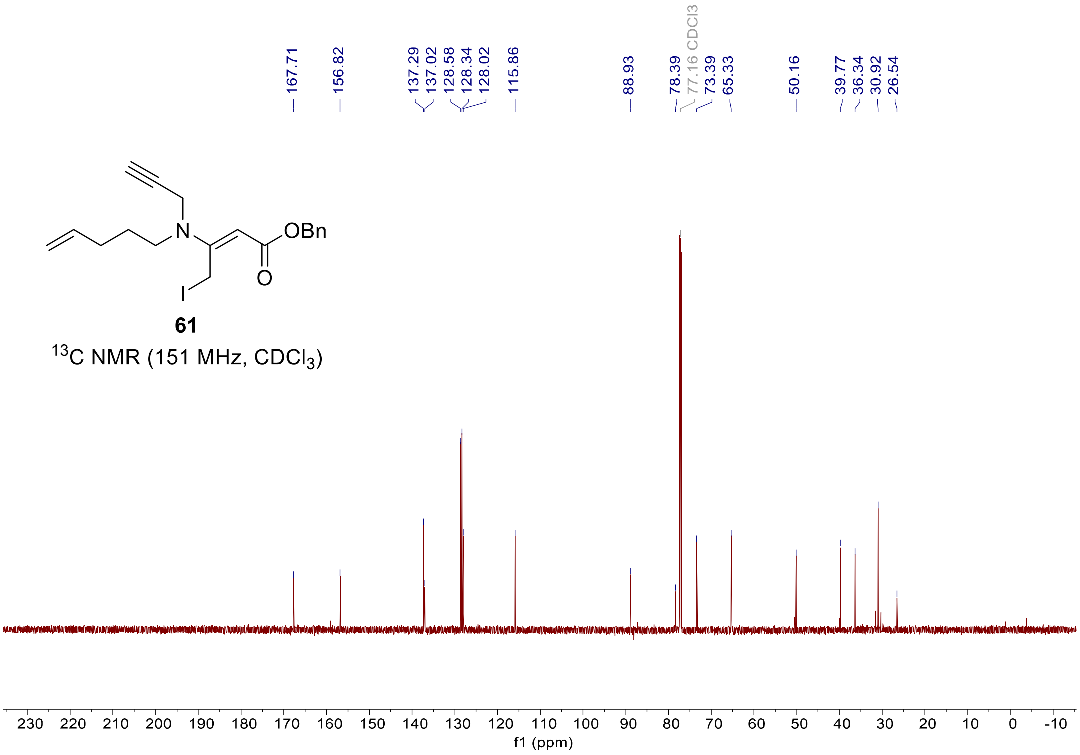

Supplementary Fig. 130. ^{13}C NMR (151 MHz, $CDCl_3$) spectrum of compound **61.**

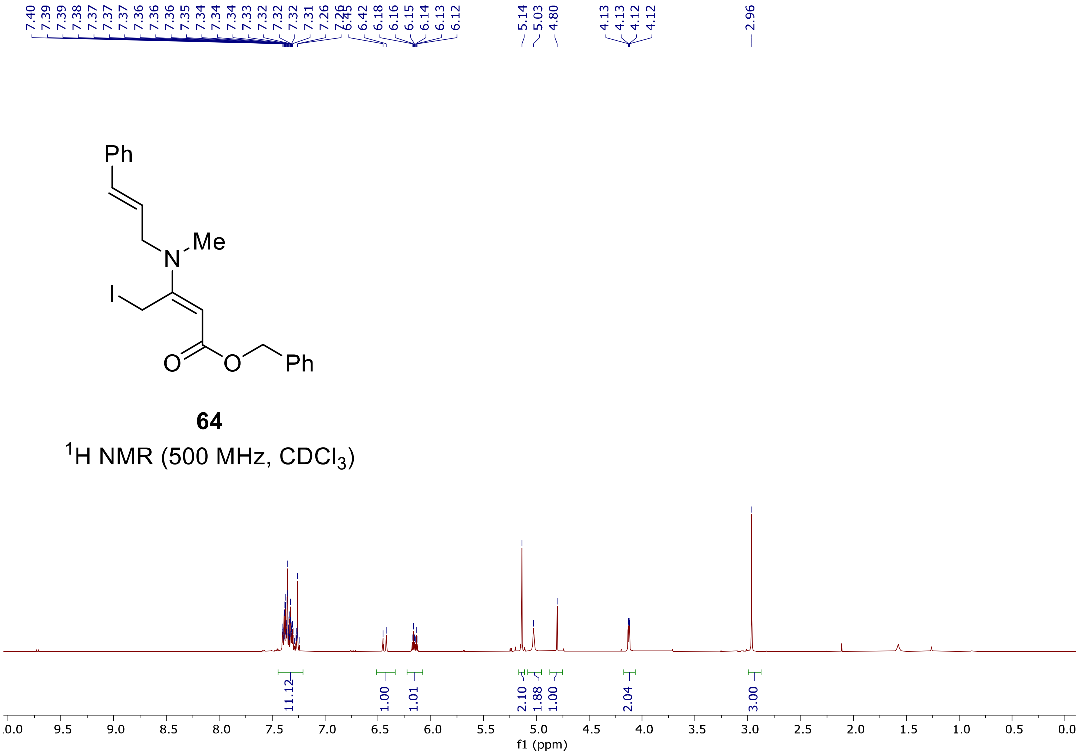

Supplementary Fig. 131. ^{1}H NMR (500 MHz, $CDCl_3$) spectrum of compound **64.**

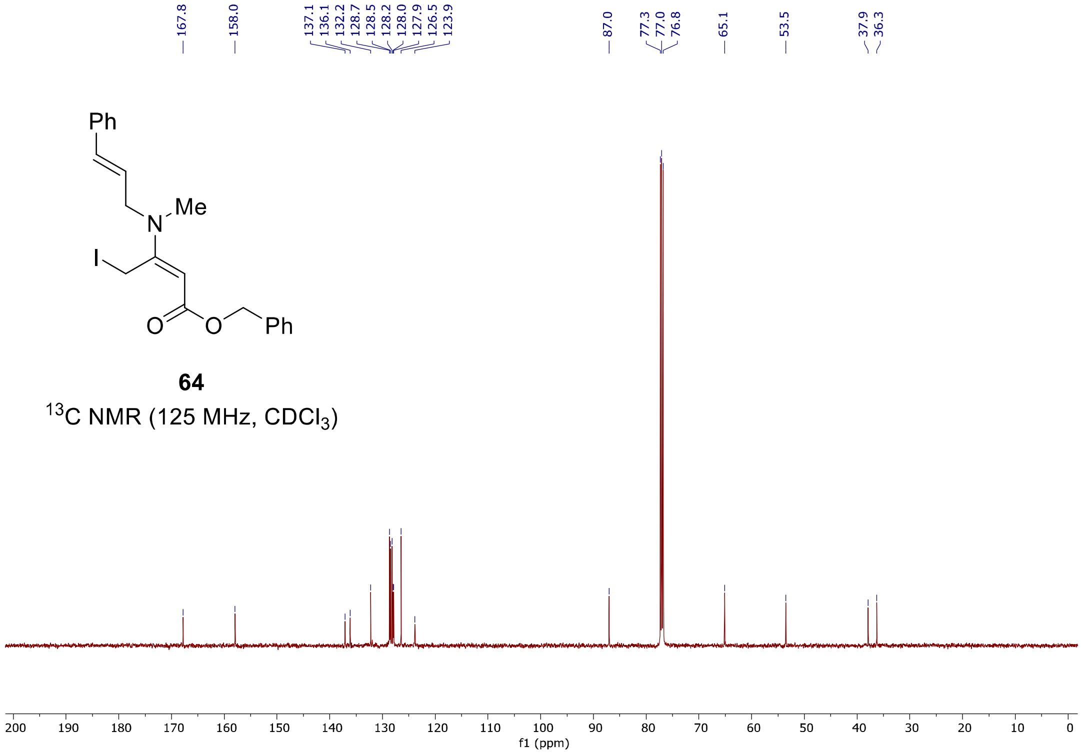

Supplementary Fig. 132. ^{13}C NMR (125 MHz, $CDCl_3$) spectrum of compound **64.**

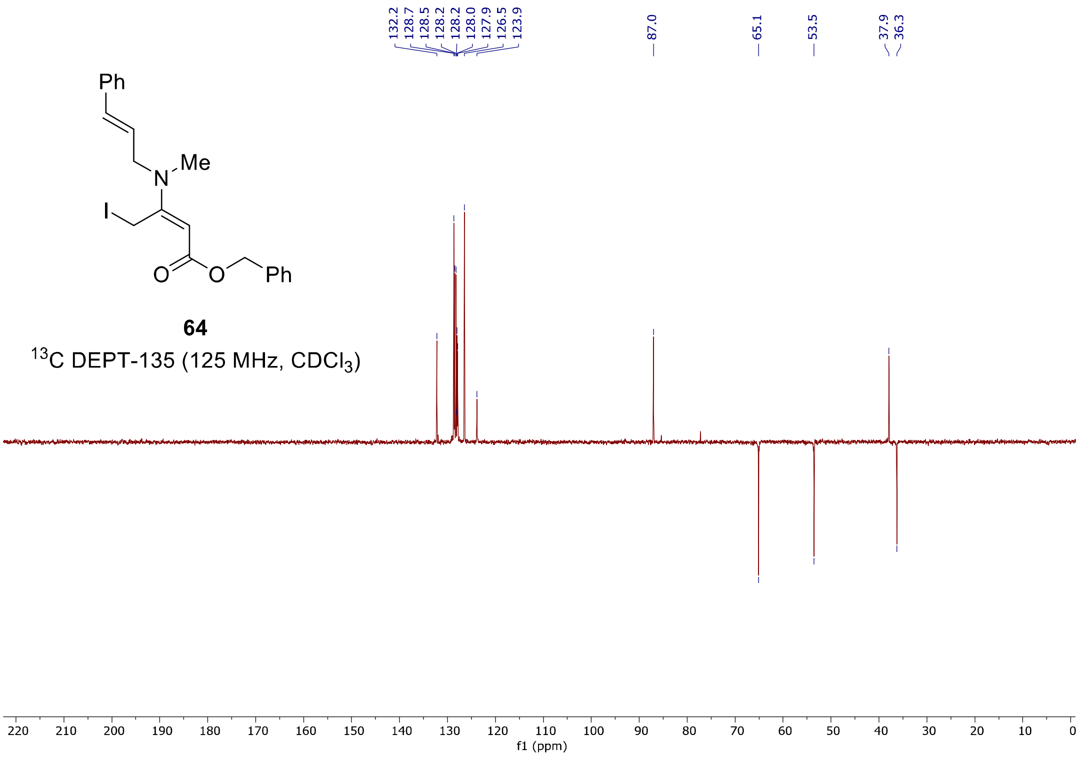

Supplementary Fig. 133. ^{13}C DEPT-135 (125 MHz, CDCl$_3$) spectrum of compound **64.**

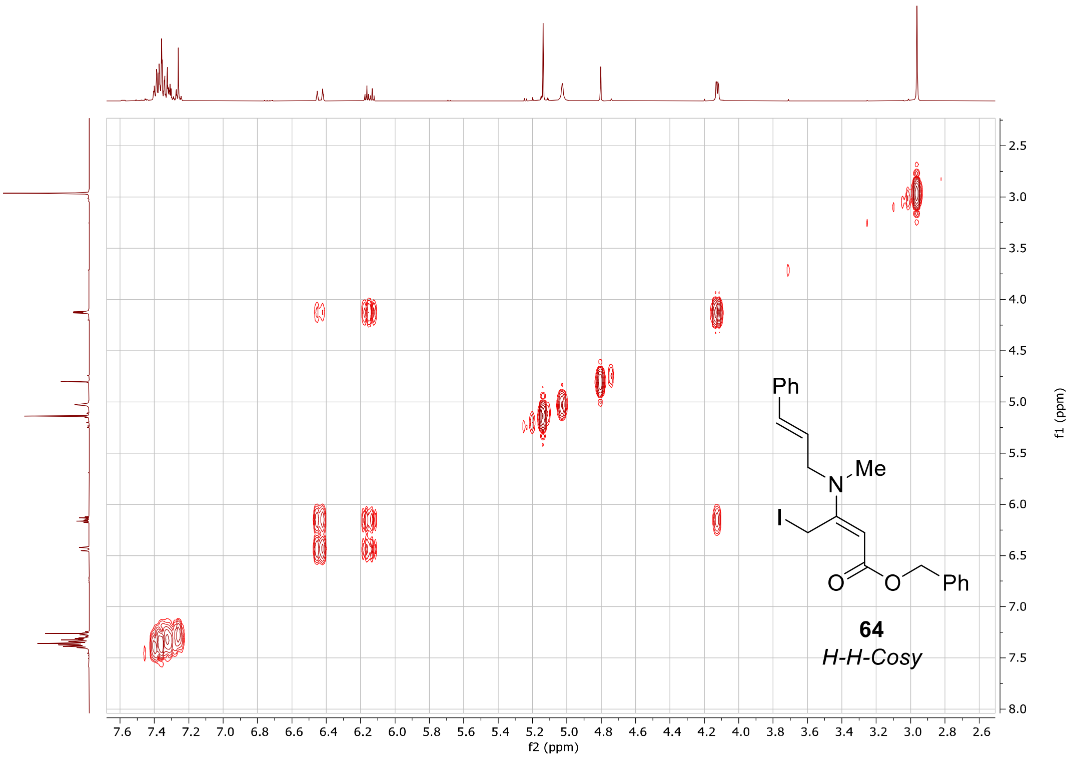

Supplementary Fig. 134. H-H COSY spectrum of compound **64.**

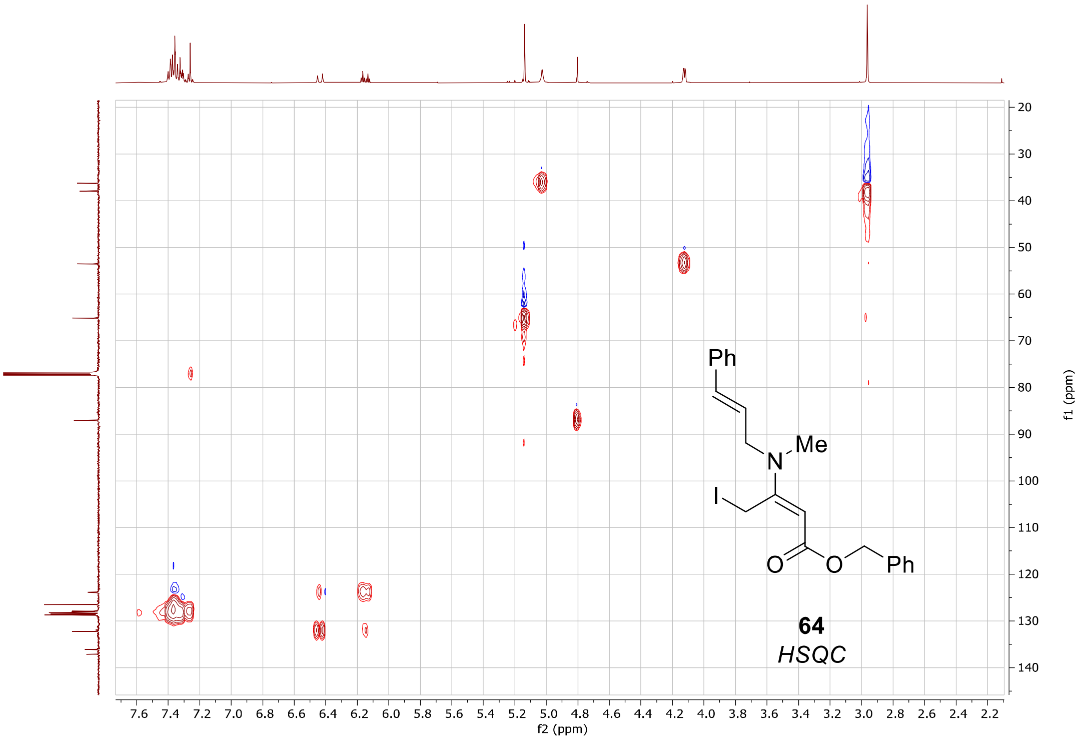

Supplementary Fig. 135. HSQC spectrum of compound **64.**

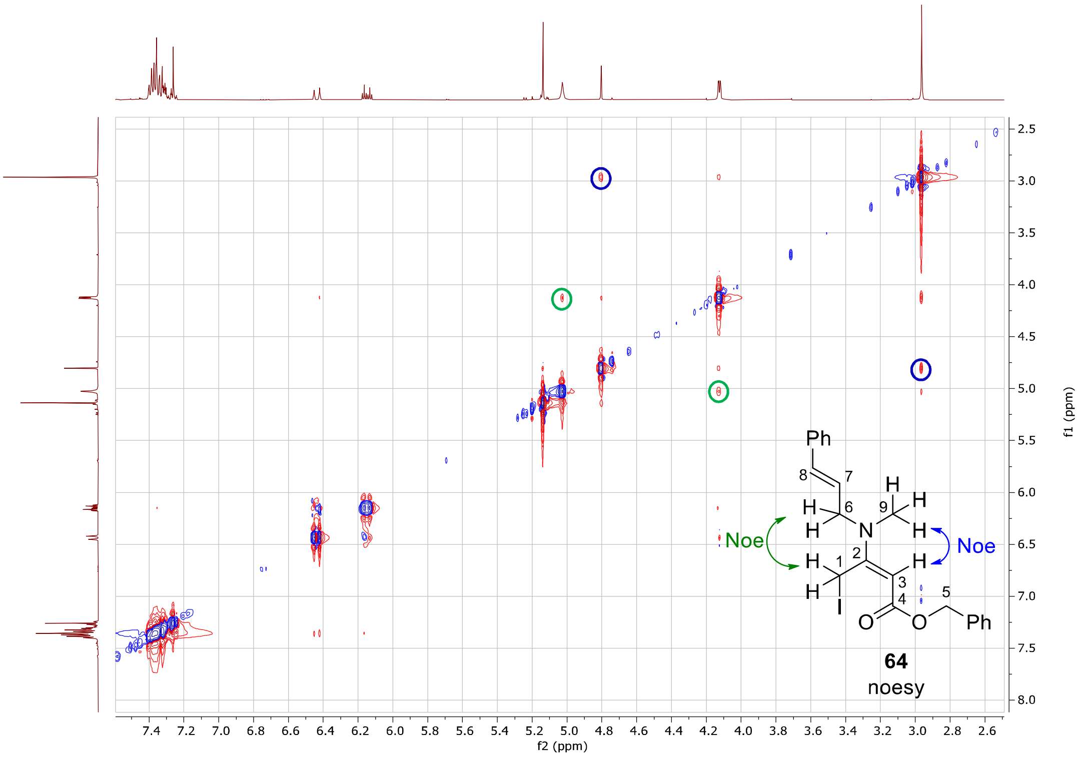

Supplementary Fig. 136. NOE spectrum of compound **64.**

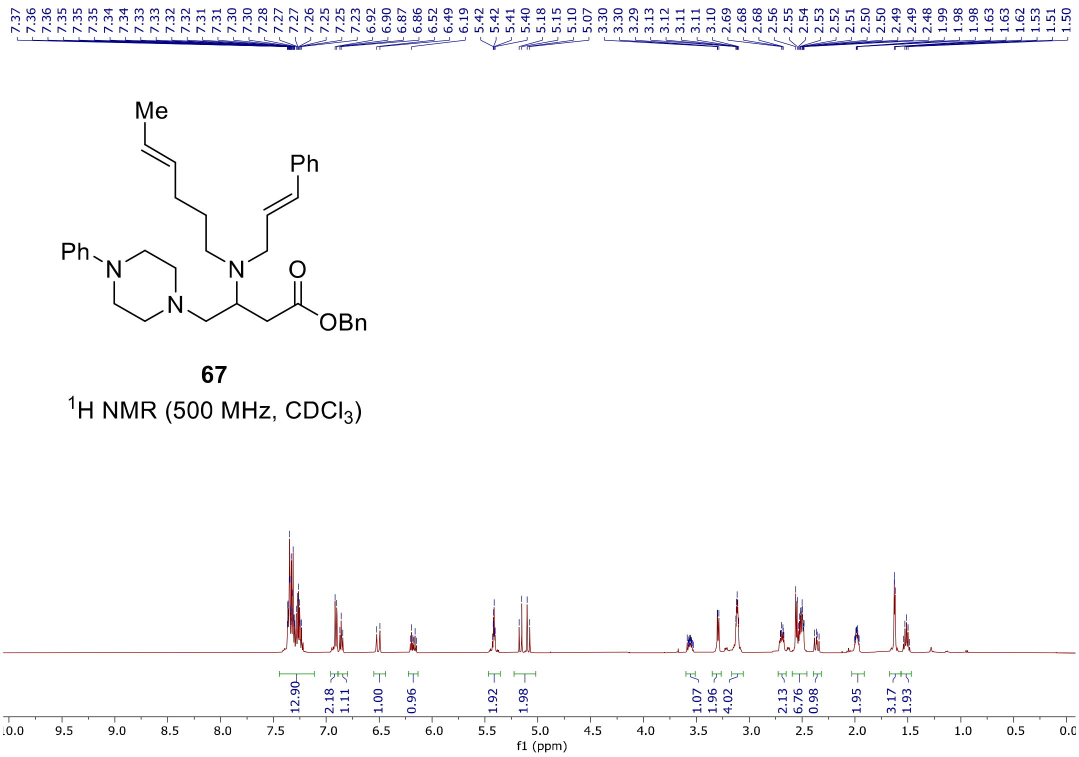

Supplementary Fig. 137. ^{1}H NMR (500 MHz, $CDCl_3$) spectrum of compound **67**.

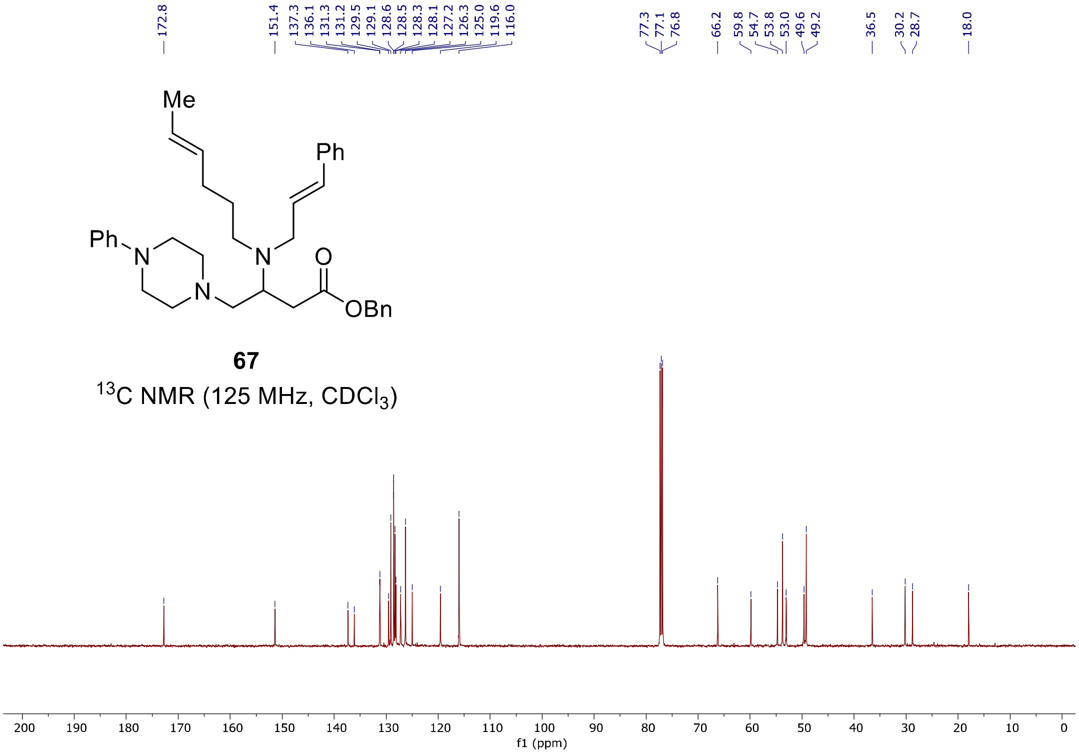

Supplementary Fig. 138. ^{13}C NMR (125 MHz, $CDCl_3$) spectrum of compound **67**.

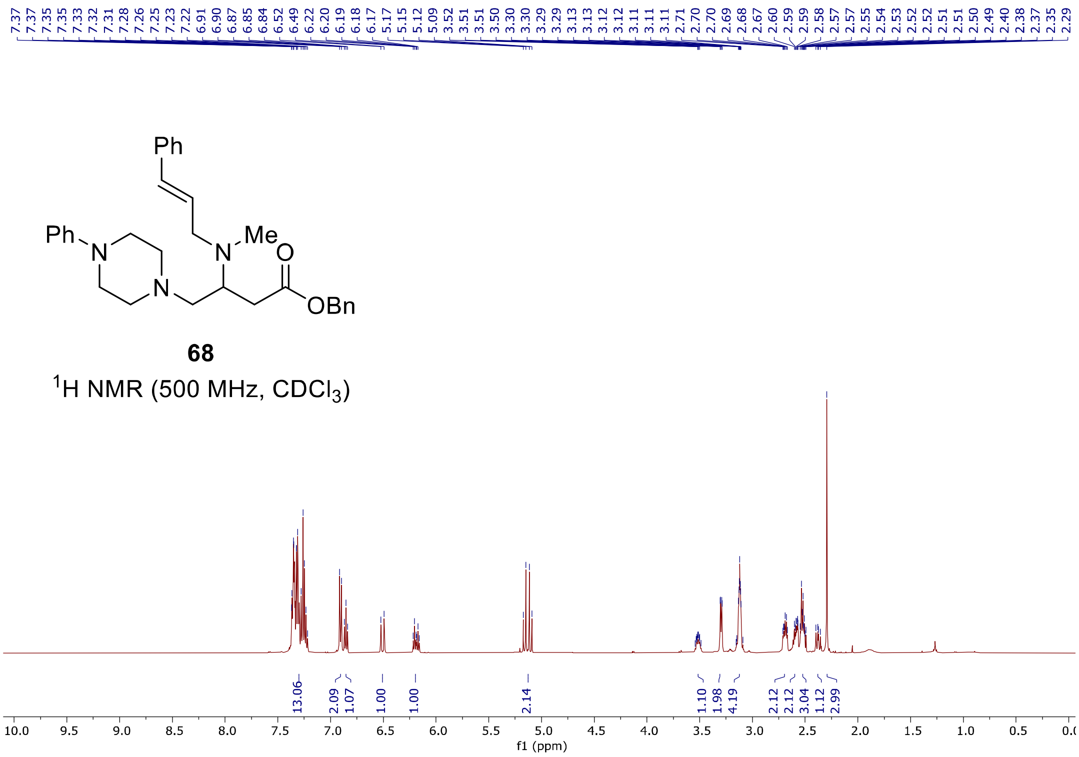

Supplementary Fig. 139. ^{1}H NMR (500 MHz, $CDCl_3$) spectrum of compound **68**.

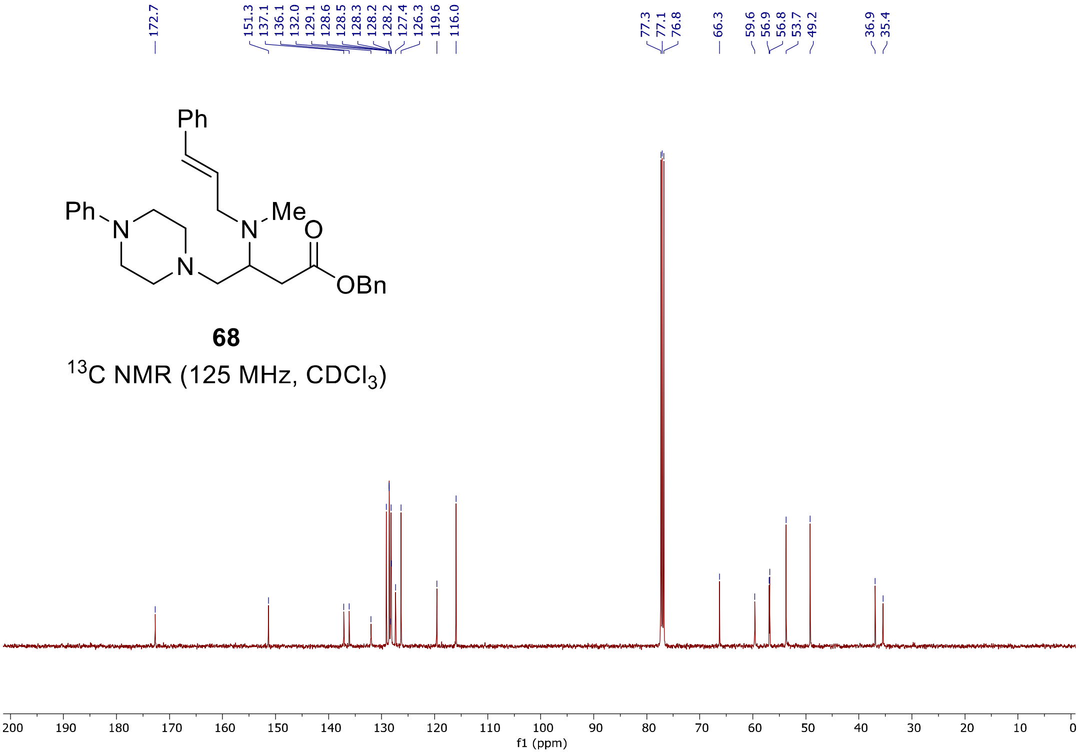

Supplementary Fig. 140. ^{13}C NMR (125 MHz, $CDCl_3$) spectrum of compound **68**.

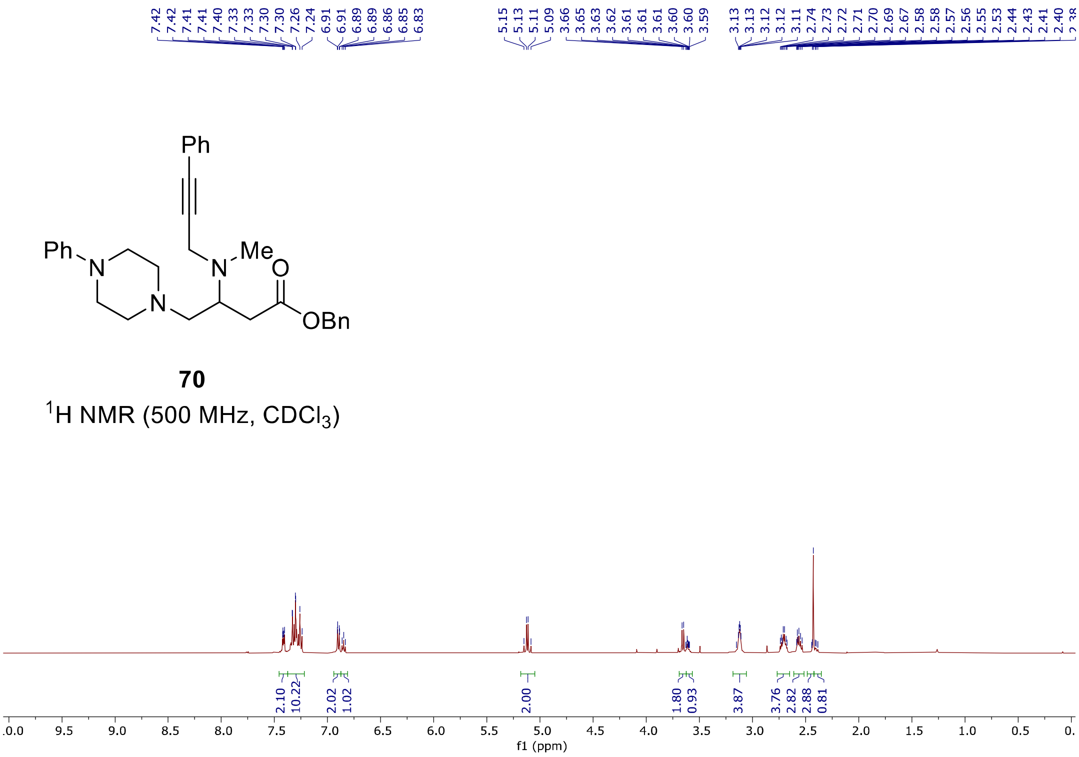

Supplementary Fig. 141. ^{1}H NMR (500 MHz, CDCl$_3$) spectrum of compound **70**.

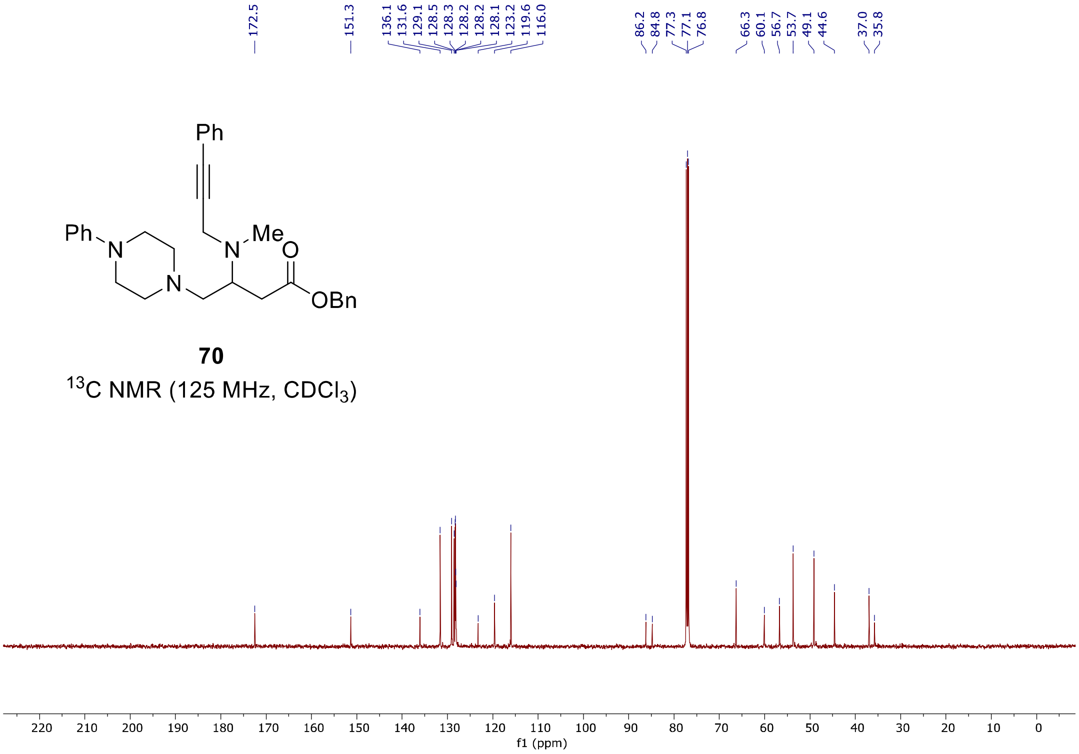

Supplementary Fig. 142. ^{13}C NMR (125 MHz, CDCl$_3$) spectrum of compound **70**.

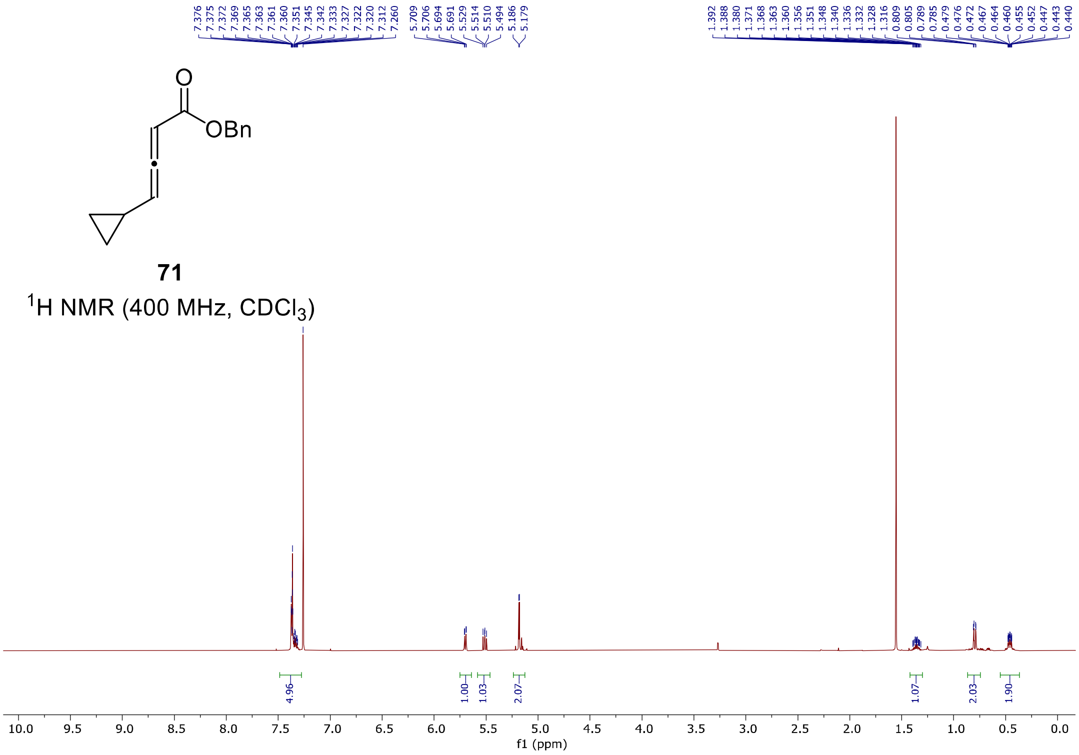

Supplementary Fig. 143. ^{1}H NMR (400 MHz, $CDCl_3$) spectrum of compound **71**.

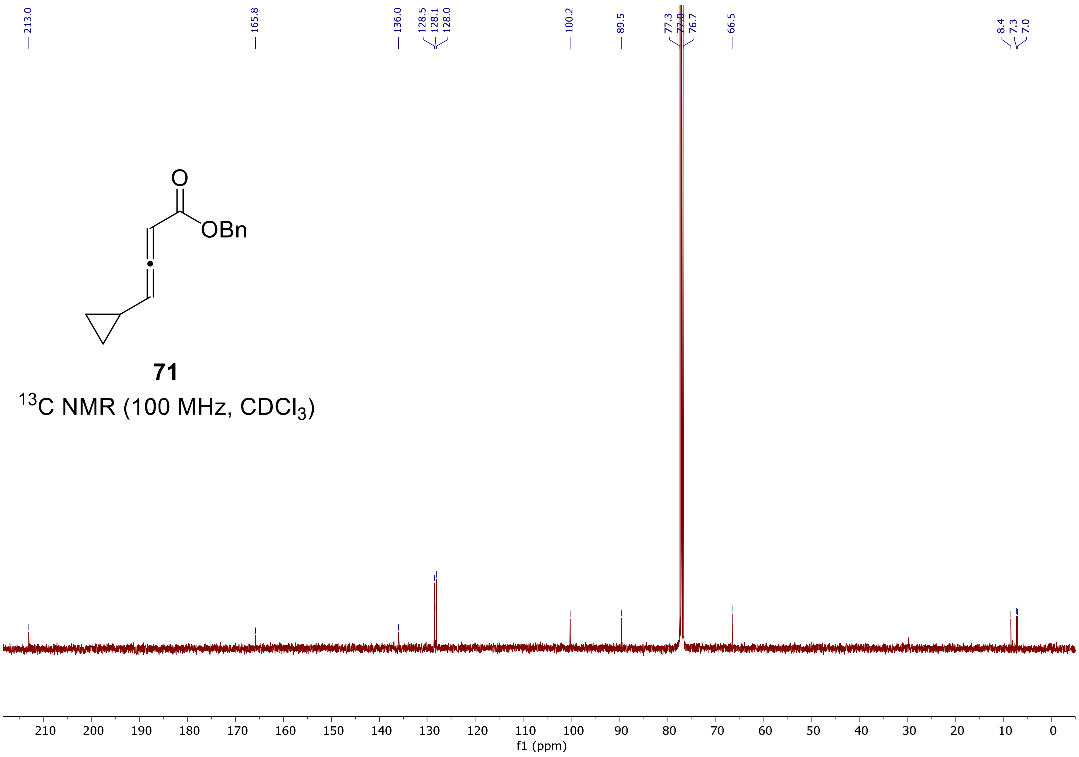

Supplementary Fig. 144. ^{13}C NMR (100 MHz, $CDCl_3$) spectrum of compound **71**.

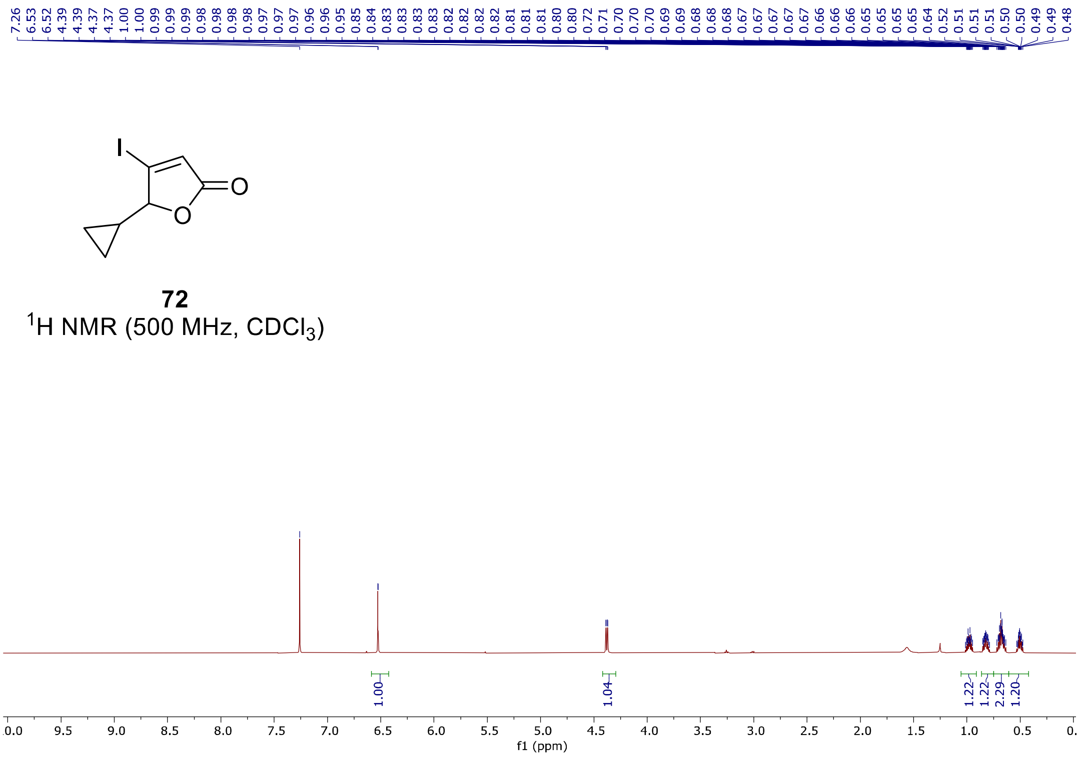

Supplementary Fig. 145. ^{1}H NMR (500 MHz, $CDCl_3$) spectrum of compound **72**.

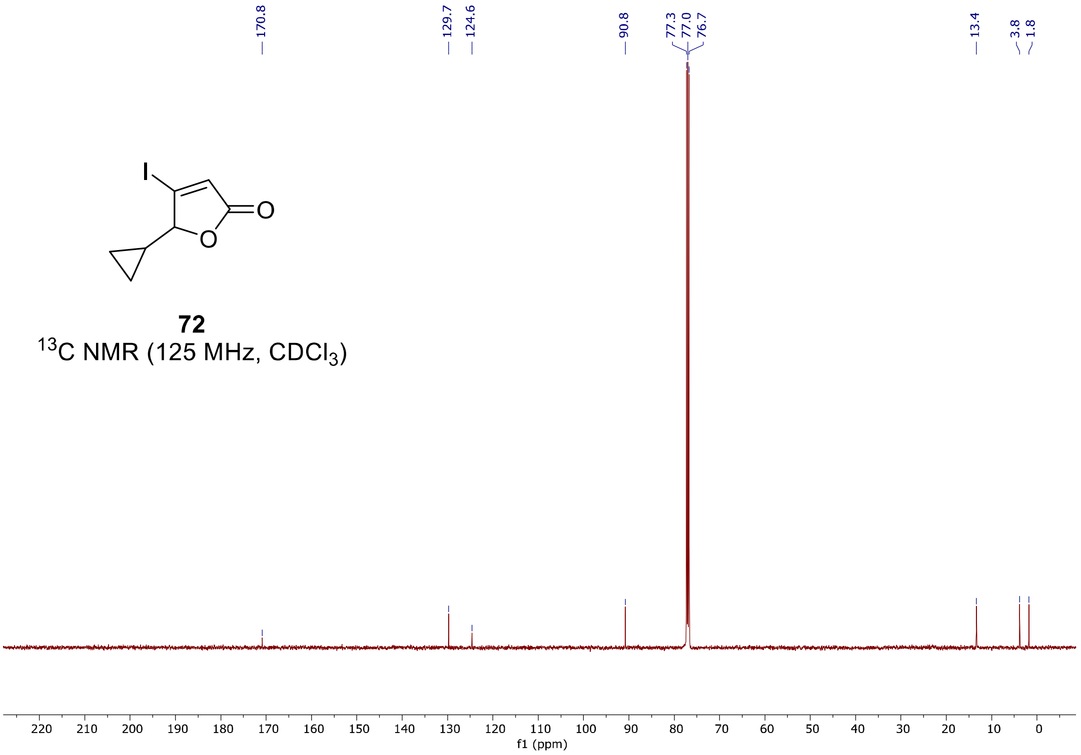

Supplementary Fig. 146. ^{13}C NMR (125 MHz, $CDCl_3$) spectrum of compound **72**.

V. Supplementary References

1. Rout, L. & Harned, A. M. Allene carboxylates as dipolarophiles in Rh-catalyzed carbonyl ylide cycloadditions. *Chem. Eur. J.* **15**, 12926−12928 (2019).
2. Huang, Z., Yang, X., Yang, F., Lu, T. & Zhou, Q. Phosphine-catalyzed domino β/γ-Additions of benzofuranones with allenoates: a method for unsymmetrical 3,3-disubstituted benzofuranones. *Org. Lett.* **19**, 3524−3527 (2017).
3. Martzel, T., Lohier, J.-F., Gaumont, A.-C., Briere, J.-F. & Perrio, S. Sulfinate-organocatalyzed (3+2) annulation reaction of propargyl or allenyl sulfones with activated imines. *Eur. J. Org. Chem*. **36**, 5069−5073 (2018).
4. Monaco, M. R., Renzi, P., Schietroma, D. M. S. & Bella, M. Biomimetic organocatalytic asymmetric synthesis of 2-substituted piperidine-type alkaloids and their analogues. *Org. Lett.* **13**, 4546−4549 (2011).
5. Lu, L., Ward, R. M. & Schomaker, J. M. Mechanistic aspects and synthetic applications of radical additions to allenes. *Chem. Rev.* **119**, 12422−12490 (2019).
6. Neale, R. S. The Chemistry of Nitrogen Radicals. V. The free-radical addition of dialkyl-*N*-chloramines to olefinic and acetylenic hydrocarbons. *J. Org. Chem.* **32**, 3263−3273 (1967).
7. Mastalir, M., Glatz, M., Pittenauer, E., Allmaier, G. & Kirchner, K. Rhenium-catalyzed dehydrogenative coupling of alcohols and amines to afford nitrogen-containing aromatics and more. *Org. Lett.* **21**, 1116−1120 (2019).
8. Sirinimal, H. S. et al. Synthetic and computational study of tin-free reductive tandem cyclizations of neutral aminyl radicals. *Org. Lett*. **20**, 6340-6344 (2018).
9. Frisch, M. J. et al. Gaussian 16 Rev. B.01. (2016).
10. Zhao, Y. & Truhlar, D. G. Density functional for spectroscopy: no long-range self-interaction error, good performance for rydberg and charge-transfer states, and better performance on average than B3LYP for ground states. *J. Phys. Chem. A.* **110**, 13126−13130 (2006).
11. Zhao, Y. & Truhlar, D. G. Density functionals with broad applicability in chemistry. *Acc. Chem. Res.* **41**, 157−167 (2008).
12. Zhao, Y. & Truhlar, D. G. Applications and validations of the Minnesota density functionals. *Chem. Phys. Lett.* **502**, 1−13 (2011).
13. Weigend, F. & Ahlrichs, R. Balanced basis sets of split valence, triple zeta valence and quadruple zeta valence quality for H to Rn: Design and assessment of accuracy. *Phys. Chem. Chem. Phys.* **7**, 3297−3305 (2005).
14. Weigend, F. Accurate Coulomb-fitting basis sets for H to Rn. *Phys. Chem. Chem. Phys.* **8**, 1057−1065 (2006).
15. Grimme, S., Antony, J., Ehrlich, S. & Krieg, H. A consistent and accurate *ab initio* parametrization of density functional dispersion correction (DFT-D) for the 94 elements H-Pu. *J. Chem. Phys.* **132**, 154104 (2010).

16. Marenich, A. V., Cramer, C. J. & Truhlar, D. G. Universal solvation model based on solute electron density and on a continuum model of the solvent defined by the bulk dielectric constant and atomic surface tensions. *J. Phys. Chem. B.* **113**, 6378−6396 (2009).
17. Engelage, E. et al. Refined SMD parameters for bromine and iodine accurately model halogen-bonding interactions in solution. *Chem. Eur. J.* **24**, 15983−15987 (2018).
18. Fukui, K. The path of chemical reactions-the IRC approach. *Acc. Chem. Res.* **14**, 363−368 (1981).
19. Legault, C. Y. CYLview20. Université de Sherbrooke (2020).
