# Supplementary material for: Modular and practical diamination of allenes
Source: Nat Commun. 2023 Mar 30;14:1774. doi: 10.1038/s41467-023-37345-8 (PMC10063549; doi:10.1038/s41467-023-37345-8)
Supplement: Supplementary file 2 — Description of Additional Supplementary Files [file 41467_2023_37345_MOESM2_ESM.docx]

**Description of Additional Supplementary Files**

**File Name: Supplementary Data 1
Description:** **Cartesian Coordinates of the Intermediates and Transition States**
