# Supplementary material for: Modular and practical diamination of allenes
Source: Nat Commun. 2023 Mar 30;14:1774. doi: 10.1038/s41467-023-37345-8 (PMC10063549; doi:10.1038/s41467-023-37345-8)
Supplement: Supplementary file 3 — Supplementary Data 1 [file 41467_2023_37345_MOESM3_ESM.docx]

**Cartesian Coordinates** **of Intermediates and Transition States:**

**SM-I**

C 3.24230600 -0.00000900 0.26849200

C 2.51465600 1.24842900 -0.22126800

C 1.04168500 1.21343300 0.18194300

N 0.42824000 0.00000400 -0.36783500

C 1.04166900 -1.21343300 0.18195000

C 2.51463400 -1.24843600 -0.22127400

I -1.63630300 0.00000100 0.00502900

H 4.28774000 -0.00001200 -0.07372400

H 3.26160200 -0.00001300 1.37182200

H 2.96783600 2.16286200 0.18992100

H 2.57925200 1.31121700 -1.31967300

H 0.51742000 2.09027400 -0.22238100

H 0.95922300 1.24531000 1.28772100

H 0.51737500 -2.09025900 -0.22236900

H 0.95919700 -1.24528400 1.28772700

H 2.57922300 -1.31122200 -1.31968000

H 2.96780700 -2.16287600 0.18990600

**2**

C 3.35014000 -0.04487400 -0.00025900

C 3.16300400 1.25752900 -0.00075600

C 2.23372900 -1.02197000 0.00015300

O 2.39029700 -2.21614000 0.00059500

O 1.03195500 -0.43901500 -0.00002800

C -1.35784700 -0.48685300 0.00030700

C -2.58736500 -1.15816100 -0.00118600

C -3.78288400 -0.44384100 -0.00138200

C -3.76438600 0.95348700 -0.00016200

C -2.54335100 1.62530500 0.00126400

C -1.34278900 0.90999300 0.00151300

C -0.09398800 -1.30601700 0.00076600

H 4.35459800 -0.47641800 -0.00059100

H -2.60306200 -2.25116400 -0.00219600

H -4.73418200 -0.97913600 -0.00251200

H -4.70051300 1.51460700 -0.00032400

H -2.51984300 2.71678000 0.00220700

H -0.38913700 1.43898300 0.00260300

H -0.05508600 -1.96028000 -0.88441400

H -0.05481500 -1.95888400 0.88699000

C 2.97958600 2.54769800 -0.00095900

H 2.90022500 3.10129800 -0.94210900

H 2.90071400 3.10166600 0.94001900

**INT1**

C 0.00008500 1.41060000 0.24158700

C -1.25169800 0.67230800 -0.22473700

C -1.18793500 -0.80822800 0.19352000

N -0.00006400 -1.43444100 -0.32688900

C 1.18784900 -0.80834400 0.19358200

C 1.25176500 0.67218100 -0.22480500

H 0.00009800 2.44496200 -0.13446300

H 0.00013300 1.46530500 1.34355200

H -2.16515300 1.12524700 0.18778400

H -1.32622300 0.72421700 -1.32336900

H -2.07870000 -1.34531700 -0.16388000

H -1.18277800 -0.85398000 1.30159000

H 2.07856100 -1.34557600 -0.16371400

H 1.18259600 -0.85389700 1.30165300

H 1.32621200 0.72399600 -1.32344200

H 2.16530100 1.12503000 0.18763300

**INT2**

C -1.03888400 1.38864600 -0.14332000

C -1.50104000 0.08784900 -0.34470500

C -0.66684500 -0.96692900 -0.79509200

C -3.58121800 -0.99341200 -1.08209800

C -3.66465500 0.74649700 0.62374000

C 0.32834700 1.89291200 -0.11555600

O 0.59897700 3.07500400 -0.07120800

O 1.29535400 0.95367100 -0.11626400

C 3.56139000 0.23742000 0.05593800

C 4.91206000 0.39584800 -0.27655800

C 5.80416700 -0.66643200 -0.14002900

C 5.35421100 -1.90419000 0.32468900

C 4.00864800 -2.06919400 0.65229700

C 3.11550700 -1.00377700 0.52139600

C 2.63431400 1.41922100 -0.05702500

H -1.76004100 2.18331400 0.03872600

H -1.03417400 -1.99214300 -0.80837600

H 0.34413400 -0.76616900 -1.13343100

H -2.90779100 -1.70336600 -1.57441500

H -3.05305300 1.23165600 1.39553500

H 5.26482100 1.36126000 -0.64800500

H 6.85458100 -0.52945300 -0.40363700

H 6.05109900 -2.73791800 0.42725900

H 3.64811500 -3.03439700 1.01316400

H 2.06229600 -1.13530600 0.77537900

H 2.86821100 2.01443300 -0.95305100

H 2.74989600 2.08755900 0.81160400

N -2.83610700 -0.21783800 -0.08712800

H -4.02018100 1.54162100 -0.06439200

H -3.95808500 -0.30472800 -1.86631100

C -4.85977500 0.06120800 1.28081700

H -4.48592400 -0.61181100 2.07002100

H -5.48055200 0.82701100 1.76893100

C -4.75844100 -1.72622600 -0.45398600

H -5.30370300 -2.26750100 -1.24133100

H -4.37360600 -2.47405800 0.25898600

C -5.67214000 -0.74371600 0.27221600

H -6.49808800 -1.27158500 0.77151600

H -6.12374000 -0.05730100 -0.46484000

**INT3**

C 0.06932900 1.61802200 -0.68777200

C -0.42229200 0.31303000 -0.42244300

C 0.17967900 -0.89402800 -0.53687800

C -2.59282800 1.59350000 0.12395500

C -1.70278200 -0.12433300 1.65400100

C 1.44023300 2.00364500 -0.76279700

O 1.84858500 3.12434600 -1.04294200

O 2.33973600 1.01266700 -0.47211300

C 4.53988000 0.22421400 -0.01105600

C 5.92672900 0.27256000 -0.20202500

C 6.73902600 -0.77675200 0.22361000

C 6.17363800 -1.89393600 0.84345500

C 4.79340900 -1.94961700 1.03338200

C 3.97944800 -0.89581400 0.61059600

C 3.69681200 1.39457000 -0.44939700

H -0.60498000 2.43373700 -0.92623300

H -0.24617600 -1.81499500 -0.13997100

H 1.16049600 -0.94138500 -1.00390600

H -2.69635200 1.85085300 -0.93645400

H -1.20973400 -1.10105700 1.67708800

H 6.37137600 1.14246400 -0.69251400

H 7.81812500 -0.72553600 0.06623600

H 6.80817600 -2.71849700 1.17344100

H 4.34276200 -2.82017100 1.51423200

H 2.89876100 -0.93888100 0.75488900

H 4.01707800 1.74465400 -1.44389200

H 3.83987100 2.24488500 0.23851800

I -3.01507500 -1.18740200 -0.88020800

N -1.84174700 0.27988700 0.19832500

H -1.00969700 0.62802500 2.06197600

H -1.93239000 2.32164500 0.60788900

C -3.01085800 -0.13076500 2.42361400

H -3.65406100 -0.94983100 2.06348800

H -2.75714000 -0.36771300 3.46689500

C -3.92417600 1.58317500 0.85700300

H -4.33017500 2.60052300 0.76223000

H -4.64504900 0.91100100 0.36766500

C -3.74004300 1.20344900 2.32171800

H -4.71169200 1.14916900 2.83148200

H -3.14579800 1.98260000 2.82635000

**SM-I-Cl**

C 3.90456500 -0.00004900 0.40164400

C 3.20666300 1.25090100 -0.12730600

C 1.70928000 1.20046200 0.17666600

N 1.11378600 0.00001000 -0.39465600

C 1.70924200 -1.20048900 0.17662400

C 3.20662800 -1.25096800 -0.12733600

I -1.00361100 0.00004700 -0.11426700

H 4.97073300 -0.00005500 0.12780200

H 3.85571300 -0.00006100 1.50478200

H 3.64073900 2.16204700 0.31318000

H 3.34144700 1.31477600 -1.22001800

H 1.20935800 2.08471200 -0.24765000

H 1.56712500 1.24584400 1.28126400

H 1.20929200 -2.08470800 -0.24772200

H 1.56707900 -1.24590700 1.28122100

H 3.34142100 -1.31482400 -1.22004800

H 3.64067200 -2.16213700 0.31313300

Cl -3.84511500 -0.00008100 0.23123700

**INT4**

C -0.32488400 -0.56696600 0.91244100

C -0.03971800 0.45118900 -0.06296500

C -0.83139600 0.86581600 -1.09120600

C 1.45319900 1.61712200 1.49347500

C 1.59008500 2.14130900 -0.87917300

C -1.55177000 -1.19754600 1.21129300

O -1.73954500 -2.08499600 2.04497600

O -2.63711200 -0.73628400 0.48732600

C -4.97927500 -0.51502900 0.12692300

C -6.24345000 -1.09690100 -0.02973600

C -7.28413600 -0.38716600 -0.62717800

C -7.07283000 0.91634200 -1.08235800

C -5.81493100 1.50054200 -0.93412200

C -4.77419600 0.79071500 -0.33138200

C -3.88667500 -1.28678900 0.82371600

H 0.49967700 -0.94929700 1.51132300

H -0.57156300 1.67591000 -1.76546200

H -1.77981700 0.35875400 -1.24526100

H 1.29237000 0.80238300 2.20489600

H 1.52343800 1.69614900 -1.87981100

H -6.41020500 -2.11963100 0.31845500

H -8.26335800 -0.85568000 -0.74373100

H -7.88522500 1.47145600 -1.55491800

H -5.63961400 2.51752700 -1.29089700

H -3.78694200 1.24217800 -0.22048900

H -3.93750400 -2.35220400 0.54300100

H -4.03699600 -1.25096400 1.91582700

I 2.88405000 -0.70099200 -0.32171200

N 1.29439800 1.07531400 0.11149300

H 0.79808200 2.90444900 -0.79293700

H 0.64201400 2.35401600 1.63993600

C 2.94153400 2.81512200 -0.67908900

H 3.75680100 2.10084000 -0.87115400

H 3.02540300 3.60664300 -1.43824000

C 2.80095200 2.27920700 1.73841900

H 2.79411600 2.67029800 2.76644400

H 3.60006400 1.52183500 1.68922000

C 3.07194400 3.38549000 0.72728900

H 4.06927500 3.82236000 0.88139300

H 2.33437900 4.19448800 0.86462600

Cl 4.51451200 -2.55827500 -0.84814500

**53-Cl**

C -0.40631500 -1.76992100 -0.81697400

C 0.56498300 -1.30010800 0.04729300

C 0.33254200 -0.09805700 0.90893400

C 2.10603600 -3.06709600 -0.68739100

C 2.61294100 -1.79451000 1.35386000

C -1.66510400 -1.17450700 -1.23635700

O -2.50532900 -1.77751900 -1.87249900

O -1.84363100 0.13231100 -0.91801200

C -4.20309800 0.31123300 -0.31410300

C -5.51788600 0.68270500 -0.62004500

C -6.56439000 0.36043600 0.24190700

C -6.30948800 -0.34250300 1.42192800

C -5.00248800 -0.71761100 1.73013300

C -3.95322700 -0.39058500 0.86793800

C -3.09198700 0.70553100 -1.26043800

H -0.27596800 -2.74011400 -1.28880800

H 0.58122700 -0.29250300 1.95664400

H -0.70022800 0.23594300 0.84307400

H 1.56252300 -2.97734300 -1.63296000

H 2.39480200 -0.85317300 1.86334200

H -5.72046000 1.22880800 -1.54510700

H -7.58481500 0.65576400 -0.00976700

H -7.12852800 -0.59829300 2.09644100

H -4.79354200 -1.26923800 2.64872700

H -2.93289500 -0.68908900 1.11932400

H -2.93430100 1.79243100 -1.21981700

H -3.36548300 0.43280000 -2.28931200

I 1.54858700 1.61963500 0.27974900

N 1.77832600 -1.91914400 0.15478600

H 2.36112500 -2.61876300 2.04904100

H 1.76747700 -3.99915500 -0.19422500

C 4.10165800 -1.84323200 1.03485500

H 4.36757800 -0.95256800 0.44130200

H 4.65881600 -1.78776800 1.98165600

C 3.59705600 -3.15003900 -1.00314500

H 3.77380000 -4.07195200 -1.57643800

H 3.86447000 -2.30231000 -1.65543100

C 4.45235600 -3.10275300 0.25467300

H 5.52213900 -3.12309400 -0.00013600

H 4.24687400 -3.99028900 0.87782900

Cl 3.19306600 4.04829500 -0.56411100

**53**

C 1.91205900 -0.83726100 1.09149700

C 1.39533600 0.45642300 0.53251700

C 0.04450100 0.64775600 0.35943600

C -0.99343700 -0.31127900 0.71066500

O -0.88509800 -1.40231300 1.23359300

N 2.31957100 1.42108400 0.25454000

I 2.20422500 -2.32729200 -0.46236400

C 1.87592900 2.75011000 -0.15988500

C 3.67064600 1.07360100 -0.20202600

O -2.20838800 0.16753300 0.35736900

C -4.58474800 -0.01530900 0.15307900

C -4.58661400 1.19955500 -0.53612300

C -5.79123700 1.76542300 -0.96270100

C -7.00145600 1.12381000 -0.70581200

C -7.00430100 -0.09171000 -0.01627100

C -5.80441400 -0.65533200 0.41057600

C -3.31441700 -0.67550300 0.62280300

H 2.87427500 -0.72925400 1.59744200

H 1.17187000 -1.29820200 1.74549000

H -0.32664900 1.55713300 -0.10408900

H 0.93156400 2.97669800 0.34708800

H 3.96380900 0.09766800 0.19489200

H -3.64149700 1.70362900 -0.73859600

H -5.77968400 2.71557700 -1.50026900

H -7.94102700 1.56738400 -1.04002700

H -7.94704400 -0.60152000 0.19116700

H -5.80871800 -1.60518700 0.95167800

H -3.37087500 -0.89209400 1.70150900

H -3.17553800 -1.64305600 0.11372200

H 3.65134600 0.98266800 -1.30555300

H 1.68108400 2.75348100 -1.25010500

C 4.69818900 2.12272100 0.19814400

H 5.67667900 1.82090900 -0.20294100

H 4.78128700 2.14289300 1.29727300

C 2.89428800 3.82708600 0.20338400

H 2.92696300 3.92103000 1.30129600

H 2.53513800 4.78685500 -0.19568300

C 4.28844200 3.49767200 -0.31445300

H 4.28139000 3.48841300 -1.41792700

H 5.01003400 4.26553300 0.00049300

**TS1**

C 2.09236900 1.77878200 0.33693300

C 1.99485800 1.11711400 1.51015800

C 1.79938200 1.00946300 2.80524100

C 0.68889800 -1.78711500 -0.62669400

C 1.81535800 -2.10897900 -1.60322500

C 2.94253400 -1.09538400 -1.43443400

C 3.41650400 -1.05003800 0.02228000

N 2.37628400 -0.88445100 1.00832300

C 1.21722500 -1.72068400 0.81095100

C 0.97841900 1.88797300 -0.60178700

O 1.05549800 2.34262600 -1.72128500

O -0.18013300 1.42535400 -0.09236200

C -2.37963900 0.54370800 -0.34823500

C -3.50268700 0.21946500 -1.11965500

C -4.52231000 -0.56724500 -0.58820200

C -4.42987700 -1.04406600 0.72195300

C -3.31352600 -0.72499400 1.49347700

C -2.29220300 0.06776400 0.96318900

C -1.30182500 1.40243900 -0.95803400

H 3.05151200 2.16602700 -0.01485500

H 1.88449700 0.05690600 3.32879700

H 1.53843300 1.91112400 3.36792100

H -0.11625900 -2.53555100 -0.67534500

H 0.24385300 -0.81423700 -0.88621600

H 1.44165100 -2.10349600 -2.63796600

H 2.20009700 -3.12364000 -1.40170500

H 3.79897100 -1.33682300 -2.08136400

H 2.58382300 -0.09363600 -1.72574600

H 4.16640600 -0.25780300 0.16897600

H 3.90959000 -2.01444900 0.26110800

H 1.52616600 -2.74278100 1.11528600

H 0.43095700 -1.40532700 1.51375200

H -3.57322200 0.58562300 -2.14710100

H -5.39171700 -0.81416800 -1.20039900

H -5.22640600 -1.66387400 1.13745200

H -3.23310400 -1.09339700 2.51803400

H -1.41925200 0.31801900 1.56775200

H -1.66711600 2.43070800 -1.11785200

H -1.00666700 1.00888500 -1.94309300

TS2

C 0.54753400 2.23226400 0.48491700

C 0.74662500 1.13920400 1.31855500

C 0.66672700 0.80193800 2.59543900

C 0.44070100 -0.49822400 -0.80519600

C 1.36782300 -1.63439400 1.14386100

C -0.70639700 2.49198000 -0.15745800

O -0.96532400 3.40770100 -0.91913000

O -1.66951200 1.57672900 0.17283500

C -3.75527600 0.48730200 -0.17085100

C -4.93430700 0.27646600 -0.89702000

C -5.74142600 -0.82839500 -0.63371300

C -5.37771100 -1.74216600 0.35885700

C -4.20456700 -1.53897200 1.08414500

C -3.39720700 -0.42851000 0.82355600

C -2.91662500 1.70404100 -0.47098800

H 1.37141000 2.90370600 0.23777900

H 0.89004200 -0.17898300 3.01089500

H 0.34522100 1.58029700 3.29163100

H 0.47487300 0.44981600 -1.35749600

H 2.07716300 -1.51880800 1.97296900

H -5.21775100 0.98588400 -1.67883200

H -6.65681300 -0.98082100 -1.20866000

H -6.00725900 -2.61001100 0.56337800

H -3.91249700 -2.24788400 1.86144600

H -2.47998900 -0.26719900 1.39221500

H -3.43237800 2.61714700 -0.12659900

H -2.77857700 1.81386200 -1.55819700

I 3.30300300 0.18663500 -0.24352700

N 1.33977100 -0.36838600 0.36963700

H 0.35074200 -1.72326300 1.56111700

H -0.56642900 -0.58938000 -0.36624100

C 1.68486200 -2.86900800 0.30793200

H 2.72955200 -2.82171500 -0.04062900

H 1.60855600 -3.74475700 0.96846600

C 0.74407100 -1.69605400 -1.69087200

H -0.01427000 -1.72190500 -2.48668600

H 1.72019300 -1.56018900 -2.18539800

C 0.73641200 -2.98953200 -0.88071600

H 1.01779000 -3.84406200 -1.51220900

H -0.28588700 -3.17776200 -0.51179900

**TS3**

C 0.03309300 2.47157800 0.37557600

C 0.23527800 1.49930600 1.30196900

C 0.31047100 1.11995900 2.56022200

C -0.00349900 -0.25441000 -0.96399900

C 0.37213900 -1.54707000 1.03289600

C -1.22494700 2.63597000 -0.32148200

O -1.43293500 3.37714100 -1.26052500

O -2.21260400 1.85755200 0.19370700

C -4.14338300 0.50716500 -0.14515200

C -5.34701000 0.18635900 -0.78588600

C -6.02985600 -0.98469300 -0.46440500

C -5.51731200 -1.85343400 0.50302500

C -4.32174200 -1.53764500 1.14644600

C -3.63792400 -0.36132400 0.82667400

C -3.42214300 1.77563400 -0.52914800

H 0.86889800 3.07015900 0.00639800

H 0.59124300 0.11867300 2.88451300

H 0.08588900 1.86419400 3.33077900

H 0.30369500 0.66229300 -1.48979900

H 0.98764000 -1.61340400 1.94192200

H -5.74745300 0.86161700 -1.54663200

H -6.96549700 -1.22370500 -0.97355500

H -6.05051100 -2.77255500 0.75251300

H -3.91381100 -2.20905500 1.90489100

H -2.70236500 -0.11183000 1.32972700

H -4.05309900 2.65541700 -0.31683900

H -3.21648900 1.78472100 -1.61092700

I 2.88848600 -0.14299100 0.00821600

N 0.73384500 -0.33687400 0.29480300

H -0.68240200 -1.43352800 1.35331000

H -1.07238700 -0.13605100 -0.70390900

C 0.50924500 -2.83656000 0.21914100

H 1.57640600 -3.01449800 0.00425000

H 0.15566300 -3.68247900 0.82843300

C 0.14708400 -1.48457700 -1.85638900

H -0.46834300 -1.35093900 -2.75916600

H 1.19534600 -1.57660500 -2.18660200

C -0.26864200 -2.73980900 -1.09090900

H -0.11403700 -3.64155900 -1.70238100

H -1.34886600 -2.68217300 -0.86715200

Cl 5.60958900 0.17127600 -0.29386900

**TS4**

C -0.44965100 -1.78280200 -1.01437800

C 0.16580900 -1.05903700 0.04460100

C -0.36486800 -0.05753400 0.82815800

C 2.31894700 -2.10036800 -0.67124800

C 1.72426200 -2.01705300 1.67015900

C -1.78732000 -1.70827500 -1.49389100

O -2.25685900 -2.36041300 -2.42231400

O -2.59757300 -0.82194400 -0.82571400

C -4.74731300 0.02542600 -0.25057400

C -5.91173100 0.68785200 -0.65628000

C -6.70436200 1.36451400 0.27076200

C -6.33781800 1.39292500 1.61760200

C -5.17527500 0.73942600 2.02838500

C -4.38580700 0.05748200 1.10103400

C -3.93744500 -0.74987400 -1.25851200

H 0.13764500 -2.51216000 -1.56514400

H 0.20360600 0.32538500 1.67726600

H -1.38020900 0.29746000 0.67911900

H 2.16445400 -1.59329500 -1.63428500

H 1.12966400 -1.46193200 2.40443100

H -6.19657200 0.67540200 -1.71141700

H -7.60860600 1.87825400 -0.06137900

H -6.95397900 1.92685000 2.34337400

H -4.87899000 0.76023800 3.07903900

H -3.47215400 -0.44785900 1.41894800

H -4.00306900 -0.27133400 -2.24970800

H -4.34832900 -1.76599200 -1.37597700

I 2.28125100 1.38976700 -0.09043600

N 1.54606000 -1.40029400 0.35115600

H 1.33510700 -3.05867200 1.64792500

H 1.96896800 -3.14694000 -0.78759300

C 3.18910200 -2.03674400 2.08830500

H 3.53849400 -0.99641200 2.19993000

H 3.27562200 -2.52208800 3.07216600

C 3.80531700 -2.12392900 -0.32429500

H 4.34106700 -2.67845300 -1.10952000

H 4.19441000 -1.09298900 -0.32683900

C 4.03946700 -2.75362900 1.04491500

H 5.10582700 -2.71854400 1.31446300

H 3.74793500 -3.81800700 1.01092300

Cl 3.65634900 3.32671100 -0.69735100
